# Genome-based insights into the resistome and mobilome of multidrug-resistant *Aeromonas* sp. ARM81 isolated from wastewater

**DOI:** 10.1007/s00203-016-1285-6

**Published:** 2016-09-02

**Authors:** Marcin Adamczuk, Lukasz Dziewit

**Affiliations:** Faculty of Biology, Institute of Microbiology, Department of Bacterial Genetics, University of Warsaw, Miecznikowa 1, 02-096 Warsaw, Poland

**Keywords:** *Aeromonas* sp. ARM81, Genome, Antibiotic resistance gene, Plasmid, Transposable element

## Abstract

The draft genome of multidrug-resistant *Aeromonas* sp. ARM81 isolated from a wastewater treatment plant in Warsaw (Poland) was obtained. Sequence analysis revealed multiple genes conferring resistance to aminoglycosides, β-lactams or tetracycline. Three different β-lactamase genes were identified, including an extended-spectrum β-lactamase gene *bla*_PER-1_. The antibiotic susceptibility was experimentally tested. Genome sequencing also allowed us to investigate the plasmidome and transposable mobilome of ARM81. Four plasmids, of which two carry phenotypic modules (i.e., genes encoding a zinc transporter ZitB and a putative glucosyltransferase), and 28 putative transposase genes were identified. The mobility of three insertion sequences (isoforms of previously identified elements IS*As12*, IS*Kpn9* and IS*As26*) was confirmed using trap plasmids.

**Electronic supplementary material:**

The online version of this article (doi:10.1007/s00203-016-1285-6) contains supplementary material, which is available to authorized users.

## Introduction

*Aeromonas* spp. are frequently recognized as pathogens of poikilothermic animals (e.g., *Aeromonas salmonicida*), but also humans (e.g., *Aeromonas hydrophila*) (Igbinosa et al. 2012). The pathogenesis of *Aeromonas* infections is multifactorial, as these bacteria produce a wide variety of virulence factors, e.g., hemolysins, entero- and endotoxins (Tomas 2012). Unfortunately, the increasing resistance to antimicrobial drugs among aeromonads threatens our ability to fight infections caused by these bacteria (Skwor et al. 2014).

Resistance to antibiotics and heavy metals is common in bacteria from wastewater treatment plants (WWTPs) (Li et al. 2015). Moreover, it has been suggested that this particular kind of environment is a specific reservoir of resistance genes (Munck et al. 2015). Aeromonads (which are commonly found in WWTPs) seem to be a good model for studies concerning the effect of water resource recovery facility effluents on the development and dissemination of antibiotic resistance. They can persist in the environment for a long time, are linked to a variety of human infections and carry multiple mobile genetic elements (MGEs) which may contribute to antibiotic resistance dissemination (Picao et al. 2013; Zhang et al. 2015).

As of August 20, 2016, according to the NCBI genome browser (http://www.ncbi.nlm.nih.gov/genome/browse/), 42 plasmids of *Aeromonas* spp. have been fully sequenced and analyzed. These plasmids are of various sizes (between 2524 and 198,307 bp) and some are cryptic (e.g., pAsa1, pAsa2 and pAsa3) (Attere et al. 2015), while others harbor virulence and/or antibiotic resistance genes (e.g., pAsa4 and pAsa5) (Vincent et al. 2014). Several other studies have investigated the transposable mobilome of *Aeromonas* spp., which led to the identification of novel transposons and insertion sequences [e.g., (Reith et al. 2008; Dobiasova et al. 2015)].

In this study, the draft genome of the multidrug-resistant strain *Aeromonas* sp. ARM81 isolated from a WWTP in Poland was investigated. Analyses of this genome provided insights into the resistome and mobilome of the strain.

## Materials and methods

### Standard molecular biology procedures

Plasmid DNA isolation by alkaline lysis and agarose gel electrophoresis was carried out according to the protocols described by Sambrook and Russell (2001). All PCRs were performed with Phusion high-fidelity DNA polymerase (Life Technologies). The active transposable elements were identified by performing PCRs with the trap plasmid insertion derivatives (as template DNAs) and a set of cassette-specific primers (Table S1, Supplementary materials) under the conditions described previously (Bartosik et al. 2003; Szuplewska and Bartosik 2009), followed by Sanger sequencing. Gaps within the plasmids and the Tn*5393*k transposon were closed by Sanger sequencing of amplicons obtained by PCRs performed with the primers listed in Table S1 (Supplementary material), under the following conditions: 35 cycles (denaturation at 96 °C for 30 s, annealing at 50 °C for 30 s, extension at 72 °C for 1 min) preceded by 3-min denaturation at 96 °C, and followed by 5-min extension at 72 °C. Amplified DNA fragments were analyzed by agarose gel electrophoresis and, if necessary, purified using a Gel Out kit (A&A Biotechnology) according to the manufacturer’s instructions.

### Antibiotic susceptibility testing

Minimum inhibitory concentrations (MICs) for *Aeromonas* sp. ARM81 were determined on Mueller–Hinton agar plates (OXOID) using MIC Test Strips (Liofilchem) containing the following antimicrobial agents: ampicillin, ceftazidime, chloramphenicol, ciprofloxacin, erythromycin, gentamicin, kanamycin, meropenem, rifampicin, streptomycin, tetracycline or trimethoprim. MICs were read after incubation at 30 °C for 24 h.

### Identification of active transposable elements (TEs)

For the identification of active TEs, two trap vectors were used: pGBG1 (Schneider et al. 2000) and pMAT1CM (constructed in this work). These vectors contain either the *cI*-*tetA* cassette or a levansucrase-coding gene (*sacB*), both enabling positive selection of transposition events (Dziewit et al. 2012). pMAT1CM was constructed by cloning the *sacB* gene (excised by the *Xba*I/*Pst*I endonucleases) from the plasmid pEBB10 (Bartosik et al. 2008) into the broad host range plasmid pBBR1MCS (Kovach et al. 1994).

Each trap plasmid was introduced into a Rif^r^ derivative of the strain ARM81 by triparental mating as described previously (Bartosik et al. 2001). The identification of functional TEs using each trap plasmid was performed according the scheme described previously (Dziewit et al. 2012).

### DNA sequencing

Genomic DNA of ARM81 was isolated using the CTAB/Lysozyme method (Sambrook and Russell 2001). An Illumina TruSeq library was constructed following manufacturer’s instructions. Sequencing was performed on an Illumina MiSeq instrument using the v3 chemistry kit. Sequence reads were filtered for quality and assembled using Newbler version 3.0 software (Roche). Sanger sequencing of the ‘active’ transposable elements localized within trap plasmids as well as PCR amplicons obtained for the closing of gaps within plasmid and transposon sequences was performed on ABI3730xl Genetic Analyzer (Life Technologies) using BigDye Terminator Mix version 3.1 chemistry (Life Technologies).

### Bioinformatics

The genome of ARM81 was automatically annotated using the RAST server (Aziz et al. 2008). To identify genetic determinants responsible for the resistance phenotype, the genome was screened using the Comprehensive Antibiotic Research Database (CARD) (McArthur et al. 2013)] and BacMet: antibacterial biocide and metal resistance genes database (Pal et al. 2014). Plasmid sequences were manually annotated using Artemis software (Rutherford et al. 2000). Similarity searches were performed using the BLAST programs (Altschul et al. 1997) and Pfam database (Finn et al. 2014). Putative ncRNA and tRNA sequences were identified using the Rfam (Nawrocki et al. 2014), tRNAScan-SE (Lowe and Eddy 1997) and ARAGORN programs (Laslett and Canback 2004). The BLAST and ISfinder (Siguier et al. 2006) database were used for identification and analysis of transposable elements. Plasmid and transposon annotations were visualized and compared using the Easyfig program (Sullivan et al. 2011).

### Nucleotide sequence accession number

The whole-genome shotgun project has been deposited in the NCBI GenBank database under the accession number SAMN04225614.

## Results and discussion

*Aeromonas* sp. ARM81 was isolated from a sample of mechanically pre-treated wastewater collected at a municipal WWTP in Warsaw (Poland). This isolate was randomly picked from bacteria that produced colonies on Luria–Bertani (LB) agar plates supplemented with 50 μg ml^−1^ kanamycin after overnight incubation at 30 °C. ARM81 was classified by 16S rRNA and *rpoB* genes analysis as *Aeromonas* sp., with the highest sequence similarity to *Aeromonas caviae* (*punctata*).

Illumina sequencing and assembly of the genome of *Aeromonas* sp. ARM81 gave 71 scaffolds consisting of 161 contigs with a total length of 4,640,951 bp. The GC content of the ARM81 draft genome was estimated as 61.3 %, which is in agreement with corresponding values for other *Aeromonas* genomes, ranging between 58.17 % (*A. salmonicida* subsp. salmonicida A449) and 62 % (*A. hydrophila* 4AK4). Automatic annotation using the RAST server identified 4240 genes with an average length of 925.8 bp (Table S2, Supplementary material). The total length of the predicted genes was 3,925,446 bp, which covers 84.6 % of the genome. Additionally, 94 tRNA genes were identified (Table S2, Supplementary material).

*Aeromonas* sp. ARM81 was initially identified on the basis of kanamycin resistance. However, antibiotic susceptibility testing performed using MIC Test Strips revealed that the strain also shows decreased susceptibility to other antimicrobials representing different classes, i.e., β-lactams, aminoglycosides and tetracyclines (Table 1).

**Table 1:** Antimicrobial susceptibility of *Aeromonas* sp. ARM81

Antimicrobial agent	AMP	C	CAZ	CIP	CN	E	K	MRP	RD	S	TE	TM
MIC (µg/ml)	>256	1.5	>256	0.75	1	16	128	0.32	4	64	24	1.5

*AMP* ampicillin, *C* chloramphenicol, *CAZ* ceftazidime, *CIP* ciprofloxacin, *CN* gentamicin, *E* erythromycin, *K* kanamycin, *MRP* meropenem, *RD* rifampicin, *S* streptomycin, *TE* tetracycline, *TM* trimethoprim

**Fig. 1:**
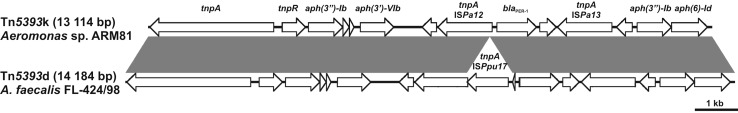
*Linear maps* showing the genetic structure of the Tn*5393*k transposon identified in *Aeromonas* sp. ARM81 and Tn*5393*d of *A. faecalis* FL-424/98 (NCBI accession number: AJ627643). *Arrows* indicate predicted genes and their transcriptional orientation. The *gray-shaded areas* connect DNA regions with 100 % nucleotide sequence identity. The following open reading frames are indicated: *tnpA* transpose-encoding gene, *tnpR* resolvase-encoding gene, *aph(3*′*)*-*VIb*, *aph(3*′′*)*-*Ib* and *aph(6)*-*Id* aminoglycoside resistance genes, and *bla*_PER-1_ β-lactamase-encoding gene

Within the ARM81 genome, seven antibiotic resistance genes were found, namely: (1–3) three different phosphotransferases, *aph(3*′*)*-*VIb*, *aph(3*′′*)*-*Ib and aph(6)*-*Id*, conferring resistance to aminoglycosides (Ramirez and Tolmasky 2010); (4) *tetA*(E) encoding a tetracycline efflux protein (Li and Nikaido 2004); (5) an extended-spectrum β-lactamase gene *bla*_PER-1_, known for its ability to hydrolyze a broad range of β-lactams, excluding carbapenems, cephamycins and oxacillin (Poirel et al. 2005); (6) *bla*_MOX-12_ encoding an AmpC β-lactamase [MOX enzymes are thought to have originated in *Aeromonas* spp. and been mobilized onto plasmids among various bacteria (Philippon et al. 2002)]; and (7) a class D β-lactamase [this class groups OXA-like proteins, which may display an activity toward extended-spectrum cephalosporins and carbapenems (Bush and Fisher 2011)]. It is worth mentioning that *bla*_PER-1_, *aph(3*′*)*-*VIb, aph(3*′′*)*-*Ib and aph(6)*-*Id* are located within a non-composite transposon (named Tn*5393*k), which resembles Tn*5393*d from *Alcaligenes faecalis* FL-424/98 (Mantengoli and Rossolini 2005) and is flanked by 5-bp direct repeats (AAGAA). Tn*5393*k differs from Tn*5393*d only by a lack of one insertion sequence, IS*Ppu17* (Fig. 1). Based on the summarized results of antibiotic susceptibility testing and identification of genes conferring resistance to three different classes of antimicrobial agents, *Aeromonas* sp. ARM81 was defined as a multidrug-resistant strain.

In addition to antibiotic resistance genes, the BacMet database (Pal et al. 2014) predicted seven heavy metal resistance genetic modules, including: (1) the *acr3* gene conferring resistance to arsenic (Bobrowicz et al. 1997); (2–4) two *czcD* genes and one *czcAB* gene cluster conferring resistance to cobalt, zinc and cadmium (Nies 1992); (v) the *zitB* gene for the zinc transporter (Lee et al. 2002); and (6–7) two *zntA* genes, conferring resistance to cadmium, lead and zinc (Rosen 2002).

Analyzing the draft genome of *Aeromonas* sp. ARM81, we also attempted to define its mobilome (i.e., plasmidome and transposable mobilome), since MGEs are frequent vehicles of horizontal transfer of various resistance genes, enabling their dissemination through microbiomes (von Wintersdorff et al. 2016). In *Aeromonas* sp. ARM81 genome, 28 predicted transposase genes were identified. Analysis revealed that they encode transposases of TEs belonging to 10 families described in the ISfinder database (Siguier et al. 2006), namely: IS*As1*, IS*4* (IS*4*, IS*10* and IS*H8* groups), IS*5* (IS*5* and IS*903* groups), IS*21*, IS*200*/IS*605*, IS*66*, IS630, IS1595, IS*1634* and Tn*3*.

To identify active (mobile) TEs, two trap vectors, pGBG1 and pMAT1CM (enabling positive selection of transposition events), were used. The analysis of 100 random insertional clones revealed only three types of insertion sequences. Sequencing showed that these were isoforms (98–99 % nucleotide sequence identity) of previously identified elements, i.e., IS*As12*, IS*Kpn9*, both belonging to IS*As1* family, and IS*As26* grouped within IS*200*/IS*605* family.

**Fig. 2:**
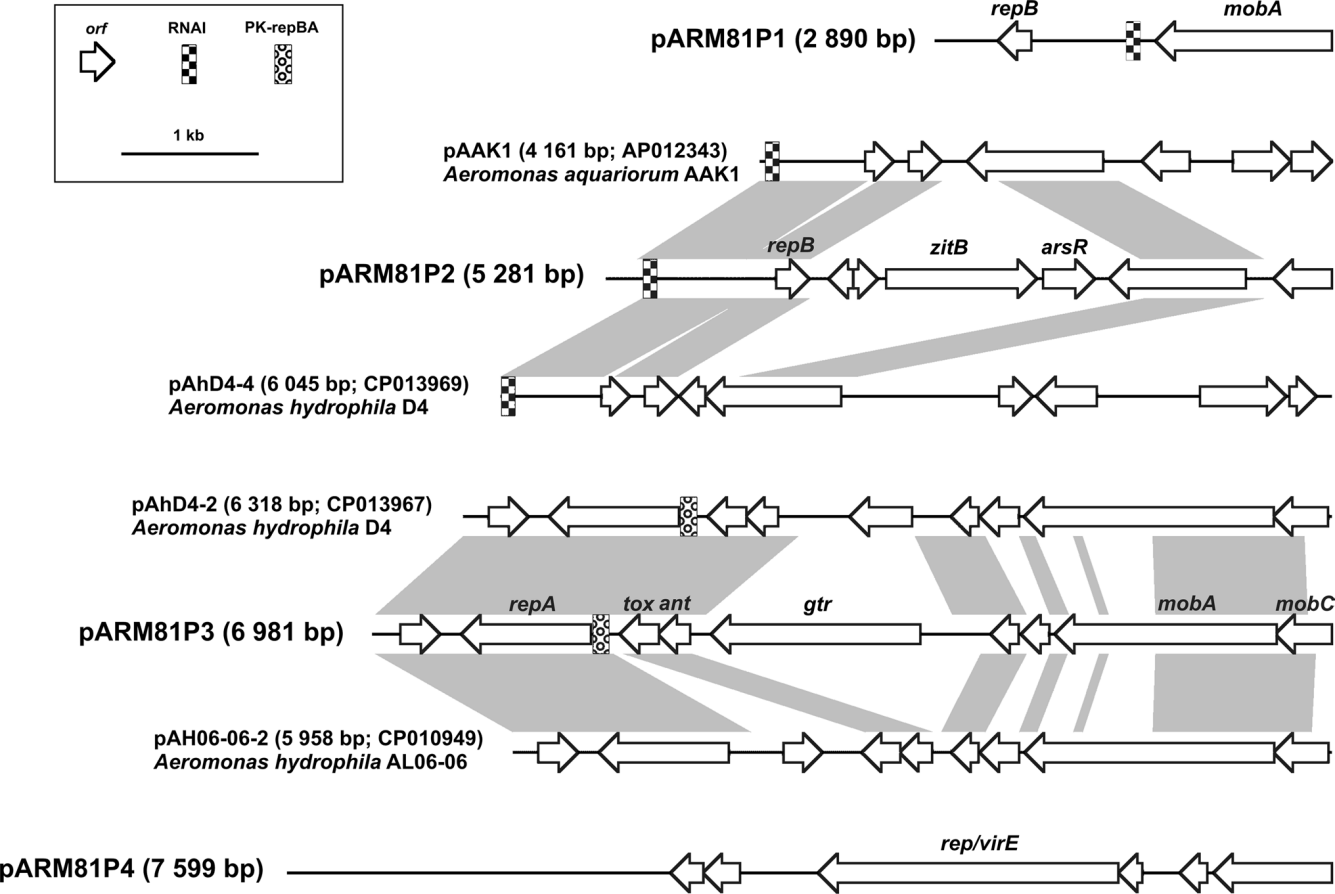
*Linear maps* showing the genetic structure of the circular plasmids pARM81P1-P4 of *Aeromonas* sp. ARM81 and the related plasmids of *Aeromonas* spp. *Arrows* indicate predicted genes and their transcriptional orientation. The *gray-shaded areas* connect DNA regions of different plasmids with at least 66 % nucleotide sequence identity. The following open reading frames are indicated: *repA* replication protein A-encoding gene, *repB* replication protein B-encoding gene, *mobA* mobilization protein A (relaxase)-encoding gene, mobC mobilization protein C-encoding gene, *zitB* zinc transporter ZitB-encoding gene, *arsR* ArsR-family transcriptional regulator-encoding gene, *tox* toxin (of a toxin–antitoxin system)-encoding gene, *ant* antitoxin (of a toxin–antitoxin system)-encoding gene, *gtr* glucosyltransferase-encoding gene and *rep*/*virE* primase/virulence-associated protein-encoding gene

Among the obtained contigs, four had much higher coverage than the average. We assumed that these corresponded to plasmids visible in agarose gel electrophoresis of the ARM81 DNA extracted by alkaline lysis procedure (data not shown). PCRs with primers designed on extremities of the contigs were used to close the plasmids. Linear representations of the plasmid genomes are shown in Fig. 2, and the summary of the genes and ncRNAs identified within the plasmids (including their position, transcriptional orientation, the size of the encoded proteins, and their closest known homologs) is presented in Table S3 (Supplementary Materials).

The smallest plasmid, ColE1-like pARM81P1, is 2890 bp and carries only two genes encoding replication (RepB) and mobilization (MobA) proteins. The second replicon, pARM81P2 (5281 bp), is another ColE1-like plasmid with *repB* and RNAI [which is known to regulate replication of ColE1 (Helmer-Citterich et al. 1988)] and six putative genes, including *zitB* encoding a zinc transporter gene and its putative regulator *arsR.* ZitB is a charge relay system for proton translocation, responsible for Zn(II) efflux, and therefore, it may confer resistance to zinc (Lee et al. 2002).

The third plasmid, pARM81P3 (6981 bp), shows some sequence similarity to plasmids previously identified in *Aeromonas* spp., e.g., pAhD4-2 and pAH06-06-2 (Fig. 1). Apparently, replication of such plasmids depends on a RepA protein whose expression is regulated by the upstream region PK-repBA (Athanasopoulos et al. 1999). Stable maintenance of this type of plasmids is probably ensured by a predicted type II (*parDE*-like) toxin–antitoxin system, while mobilization is apparently mediated by MobA and MobC proteins. Analysis also revealed a gene (*gtr*) encoding a putative glucosyltransferase, which might alter the structure and properties of lipopolysaccharides produced by the ARM81 strain (Lehane et al. 2005).

The largest plasmid, pARM81P4, is 7599 bp. Its nucleotide sequence and predicted genes do not show any significant similarity to sequences deposited in GenBank (NCBI); however, the predicted protein product of the largest gene contains two distinguishable domains: (1) PriCT-2 found in primases and (2) VirE of the virulence-associated proteins, which suggests that it may be a bifunctional protein.

Comparative genomic analyses of the ARM81 plasmids revealed that pARM81P2 and pARM81P3 are similar to several replicons found in *Aeromonas* spp., e.g., *A. aquariorum* AAK1 (Wu et al. 2012), *A. hydrophila* D4 (NCBI accession numbers: CP013967 and CP013969) and *A. hydrophila* AL06-06 (Tekedar et al. 2015), while pARM81P1 and pARM81P4 are unique, as they do not show homology with other known replicons (Fig. 2). The shared similarities between pARM81P2 and pARM81P3 and their homologs cover regions which might be defined as a ‘plasmid backbone,’ as they encode genes responsible for plasmid replication, maintenance and mobilization to conjugal transfer (Fig. 2). This is a common phenomenon observed in other bacterial plasmids harbored by representatives of a specific genus [e.g., plasmids of *Paracoccus* spp. (Maj et al. 2013) or *Psychrobacter* spp. (Dziewit et al. 2013)] or belonging to a particular incompatibility group [e.g., IncL/M plasmids (Adamczuk et al. 2015)], which supports the hypothesis that there is a clear distinction between the permanent members (genes) constituting plasmid backbones and the operative genes (additional load) that can be exchanged between replicating elements in the cell and are not conserved elements of plasmids (Norman et al. 2009).

WWTPs are known to be rich in nutrients and a mixture of toxic compounds/selective agents, including antibiotics and heavy metals (Baker-Austin et al. 2006). We are unable to say whether *Aeromonas* sp. ARM81 is permanently inhabiting this niche or it was just ‘passing through.’ Either way, insights into its genome content suggest that the strain is well adapted to unfavorable conditions as it contains multiple antibiotic and heavy metal resistance genes and a number of efflux transporters extruding a variety of compounds from the cell (data not shown). Identification of plasmids and various TEs (including the Tn*5393*d-like transposon with *bla*_PER-1_) in the ARM81 genome suggests that this strain actively participates in horizontal transfer of various genetic information (including antibiotic resistance genes) in the environment.

## Electronic supplementary material

Below is the link to the electronic supplementary material.

Supplementary material 1 (DOCX 14 kb)

Supplementary material 2 (DOCX 513 kb)

Supplementary material 3 (DOCX 33 kb)
