# Supplementary material for: Genome-based insights into the resistome and mobilome of multidrug-resistant Aeromonas sp. ARM81 isolated from wastewater
Source: Arch Microbiol. 2016 Sep 2;199(1):177–83. doi: 10.1007/s00203-016-1285-6 (PMC5216076; doi:10.1007/s00203-016-1285-6)
Supplement: Supplementary file 1 — Supplementary material 1 (DOCX 14 kb) [file 203_2016_1285_MOESM1_ESM.docx]

Table S1. Primers used in this study.

| **Primer name** | **Sequence** | **Function** |
| --- | --- | --- |
| c80arm81f1 | 5’- GCCCGGCATTAGTTACTCAG -3’ | Gap closing within plasmid pARM81P4 |
| c80arm81r1 | 5’-ACTAAGCCAGCTCGCTCTTC-3’ |  |
| c85arm81f1 | 5’-CAGCCAGCTCAGATCGTAGG-3’ | Gap closing within plasmid pARM81P3 |
| c85arm81r1 | 5’-GCGCGGTGGTCATTGAATCG-3’ |  |
| c88arm81f1 | 5’-ACTGAAGCAGGTACAGAGTG-3’ | Gap closing within plasmid pARM81P2 |
| c88arm81r1 | 5’-CACTTCTGGGCCTCCAAATC-3’ |  |
| c94arm81f1 | 5'-GTCGGTCAGTCGGTCACATC-3' | Gap closing within plasmid pARM81P1 |
| c94arm81r1 | 5'-AACCAGACGGCCCATCATTG-3' |  |
| A81TN1F | 5’-AGCGGGATCTGATCCGAGAG-3’ | Gap closing within Tn*5393*k transposon |
| A81TN1R | 5’-AACATCCGATGGCGACTGAC-3’ |  |
| A81TN2F | 5’-CCGATACTGATTCGCTCAAC-3’ |  |
| A81TN2R | 5’-GCATACAGCTTCGCCATTAG-3’ |  |
| A489SB | 5’-CAGACCGCTAACACAGTACA-3’ | Identification of transposable elements integrated into pMAT1CM trap vector |
| A869SB | 5’-TTAGGATCTCCGGCTAATGC-3’ |  |
| B824SB | 5’-ACTATCACGGCTACCACATC-3’ |  |
| B1253SB | 5’-TTGTCGCCTGAGCTGTAGTT-3’ |  |
| C1225SB | 5’-GATGAAGGCAACTACAGCTC-3’ |  |
| C1639SB | 5’-ACGTAATGCCGTCAATCGTC-3’ |  |
| D1619SB | 5’-TGACGATTGACGGCATTACG-3’ |  |
| D1928SB | 5’-CCTTGTTCAAGGATGCTGTC-3’ |  |
| ALIS | 5’-TTGTAATCAGCTATGCGCCG-3’ | Identification of transposable elements integrated into pGBG1 trap vector |
| ARIS | 5’-TCTGGCTTGAGGTTGAAGGT-3’ |  |
| BLIS | 5’-TGGTGCGGTCATGGAATTAC-3’ |  |
| BRIS | 5’-GTATGCAGCCGTCACTTAGA-3’ |  |
| CLIS | 5’-TCCCTGCCTGAACATGAGAA-3’ |  |
| CRIS | 5’-ACACAAGAGCAGCTTGAGGA-3’ |  |
| DLIS | 5’-TCTTGTCTGCGACAGATTCC-3’ |  |
| DRIS | 5’-TTCATACACGGTGCCTGACT-3’ |  |
| ELIS | 5’-GGTTGCATGTACTAAGGAGG-3’ |  |
| ERIS | 5’-GCAAGACTGGCATGATAAGG-3’ |  |
