# Supplementary material for: Genome-based insights into the resistome and mobilome of multidrug-resistant Aeromonas sp. ARM81 isolated from wastewater
Source: Arch Microbiol. 2016 Sep 2;199(1):177–83. doi: 10.1007/s00203-016-1285-6 (PMC5216076; doi:10.1007/s00203-016-1285-6)
Supplement: Supplementary file 2 — Supplementary material 2 (DOCX 513 kb) [file 203_2016_1285_MOESM2_ESM.docx]

Table S2. Genes and RNAs identified within *Aeromonas* sp. ARM81 genome.

| Type | Contig | Start | Stop | Frame | Strand | Length (bp) | Function |
| --- | --- | --- | --- | --- | --- | --- | --- |
| CDS | contig00001 | 1308 | 43 | -3 | - | 1266 | Endoribonuclease L-PSP |
| CDS | contig00001 | 2378 | 3250 | 2 | + | 873 | hypothetical protein |
| CDS | contig00001 | 3628 | 4029 | 1 | + | 402 | hypothetical protein |
| CDS | contig00001 | 5265 | 4141 | -3 | - | 1125 | Anhydro-N-acetylmuramic acid kinase (EC 2.7.1.-) |
| CDS | contig00001 | 6771 | 5509 | -3 | - | 1263 | Peptidase, M23/M37 family |
| CDS | contig00001 | 6805 | 6918 | 1 | + | 114 | hypothetical protein |
| CDS | contig00001 | 6934 | 8133 | 1 | + | 1200 | Tyrosyl-tRNA synthetase (EC 6.1.1.1) |
| CDS | contig00001 | 10692 | 8212 | -3 | - | 2481 | Putative isomerase |
| CDS | contig00001 | 11688 | 10876 | -3 | - | 813 | Transcriptional regulator, AraC family |
| CDS | contig00001 | 12233 | 11700 | -2 | - | 534 | Phospholipid-binding protein |
| CDS | contig00001 | 13668 | 12412 | -3 | - | 1257 | Adenylosuccinate synthetase (EC 6.3.4.4) |
| CDS | contig00001 | 13873 | 13688 | -1 | - | 186 | hypothetical protein |
| CDS | contig00001 | 13859 | 14761 | 2 | + | 903 | Transcriptional regulator, LysR family |
| CDS | contig00001 | 16180 | 14825 | -1 | - | 1356 | Immunogenic protein |
| CDS | contig00001 | 17468 | 16329 | -2 | - | 1140 | Cystathionine beta-lyase, type II (EC 4.4.1.8) |
| CDS | contig00001 | 17617 | 17444 | -1 | - | 174 | hypothetical protein |
| CDS | contig00001 | 17633 | 18805 | 2 | + | 1173 | Putative transport protein |
| CDS | contig00001 | 19761 | 18877 | -3 | - | 885 | Transcriptional regulator, LysR family |
| CDS | contig00001 | 20606 | 19851 | -2 | - | 756 | FIG00553873: hypothetical protein |
| CDS | contig00001 | 20831 | 21838 | 2 | + | 1008 | Possible protease sohB (EC 3.4.21.-) |
| CDS | contig00001 | 23406 | 22069 | -3 | - | 1338 | Succinylarginine dihydrolase (EC 3.5.3.23) |
| CDS | contig00001 | 23767 | 26388 | 1 | + | 2622 | DNA topoisomerase I (EC 5.99.1.2) |
| CDS | contig00001 | 26504 | 26674 | 2 | + | 171 | FIG00362224: hypothetical protein |
| CDS | contig00001 | 27108 | 26758 | -3 | - | 351 | methylated-DNA--protein-cysteine methyltransferase-related protein |
| CDS | contig00001 | 28728 | 27175 | -3 | - | 1554 | Aerotaxis sensor receptor protein |
| CDS | contig00001 | 30777 | 28921 | -3 | - | 1857 | Beta-hexosaminidase (EC 3.2.1.52) |
| CDS | contig00001 | 31021 | 30845 | -1 | - | 177 | Glucosamine kinase GpsK (EC 2.7.1.8) |
| CDS | contig00001 | 31718 | 31041 | -2 | - | 678 | Glucosamine kinase GpsK (EC 2.7.1.8) |
| CDS | contig00001 | 33550 | 31823 | -1 | - | 1728 | Glucosamine-link cellobiase (EC 3.2.1.21) |
| CDS | contig00001 | 34656 | 33658 | -3 | - | 999 | (GlcNAc)2 ABC transporter, ATP-binding component 2 |
| CDS | contig00001 | 35690 | 34668 | -2 | - | 1023 | (GlcNAc)2 ABC transporter, ATP-binding component 1 |
| CDS | contig00001 | 36592 | 35687 | -1 | - | 906 | (GlcNAc)2 ABC transporter, permease component 2 |
| CDS | contig00001 | 37577 | 36594 | -2 | - | 984 | (GlcNAc)2 ABC transporter, permease component 1 |
| CDS | contig00001 | 39377 | 37695 | -2 | - | 1683 | (GlcNAc)2 ABC transporter, periplasmic substrate-binding protein |
| CDS | contig00001 | 41209 | 39923 | -1 | - | 1287 | Ribosomal protein S12p Asp88 (E. coli) methylthiotransferase |
| CDS | contig00001 | 41518 | 42609 | 1 | + | 1092 | Response regulator |
| CDS | contig00001 | 43002 | 43370 | 3 | + | 369 | Arsenical resistance operon repressor |
| CDS | contig00001 | 43459 | 44523 | 1 | + | 1065 | Arsenical-resistance protein ACR3 |
| CDS | contig00001 | 44575 | 45000 | 1 | + | 426 | Arsenate reductase (EC 1.20.4.1) |
| CDS | contig00001 | 45104 | 45880 | 2 | + | 777 | putative lipoprotein |
| CDS | contig00001 | 45965 | 46129 | 2 | + | 165 | hypothetical protein |
| CDS | contig00001 | 46259 | 46576 | 2 | + | 318 | Flagellar hook-basal body complex protein FliE |
| CDS | contig00001 | 46632 | 48332 | 3 | + | 1701 | Flagellar M-ring protein FliF |
| CDS | contig00001 | 48325 | 49386 | 1 | + | 1062 | Flagellar motor switch protein FliG |
| CDS | contig00001 | 49457 | 50260 | 2 | + | 804 | Flagellar assembly protein FliH |
| CDS | contig00001 | 50335 | 51666 | 1 | + | 1332 | Flagellum-specific ATP synthase FliI |
| CDS | contig00001 | 51683 | 52123 | 2 | + | 441 | Flagellar protein FliJ |
| CDS | contig00001 | 52211 | 54130 | 2 | + | 1920 | Flagellar hook-length control protein FliK |
| CDS | contig00001 | 54188 | 54694 | 2 | + | 507 | Flagellar biosynthesis protein FliL |
| CDS | contig00001 | 54705 | 55778 | 3 | + | 1074 | Flagellar motor switch protein FliM |
| CDS | contig00001 | 55820 | 56215 | 2 | + | 396 | Flagellar motor switch protein FliN |
| CDS | contig00001 | 56230 | 56595 | 1 | + | 366 | Flagellar biosynthesis protein FliQ |
| CDS | contig00001 | 56582 | 57340 | 2 | + | 759 | Flagellar biosynthesis protein FliP |
| CDS | contig00001 | 57464 | 57733 | 2 | + | 270 | Flagellar biosynthesis protein FliQ |
| CDS | contig00001 | 57825 | 58622 | 3 | + | 798 | Flagellar biosynthesis protein FliR |
| CDS | contig00001 | 58697 | 59827 | 2 | + | 1131 | Flagellar biosynthesis protein FlhB |
| CDS | contig00001 | 60098 | 62200 | 2 | + | 2103 | Flagellar biosynthesis protein FlhA |
| CDS | contig00001 | 62217 | 63716 | 3 | + | 1500 | Flagellar biosynthesis protein FlhF |
| CDS | contig00001 | 63718 | 64593 | 1 | + | 876 | Flagellar synthesis regulator FleN |
| CDS | contig00001 | 64628 | 65305 | 2 | + | 678 | RNA polymerase sigma factor for flagellar operon |
| CDS | contig00001 | 65416 | 65799 | 1 | + | 384 | Chemotaxis regulator - transmits chemoreceptor signals to flagelllar motor components CheY |
| CDS | contig00001 | 65812 | 66552 | 1 | + | 741 | Chemotaxis response - phosphatase CheZ |
| CDS | contig00001 | 66564 | 68720 | 3 | + | 2157 | Signal transduction histidine kinase CheA (EC 2.7.3.-) |
| CDS | contig00001 | 68770 | 69867 | 1 | + | 1098 | Chemotaxis response regulator protein-glutamate methylesterase CheB (EC 3.1.1.61) |
| CDS | contig00001 | 69870 | 70607 | 3 | + | 738 | Flagellar motor rotation protein MotA |
| CDS | contig00001 | 70610 | 71518 | 2 | + | 909 | Flagellar motor rotation protein MotB |
| CDS | contig00001 | 71523 | 72317 | 3 | + | 795 | Chromosome (plasmid) partitioning protein ParA |
| CDS | contig00001 | 72314 | 73180 | 2 | + | 867 | CheW domain protein |
| CDS | contig00001 | 73250 | 73738 | 2 | + | 489 | Positive regulator of CheA protein activity (CheW) |
| CDS | contig00001 | 73810 | 74196 | 1 | + | 387 | ATPase involved in DNA repair |
| CDS | contig00001 | 74416 | 75051 | 1 | + | 636 | ABC transporter involved in cytochrome c biogenesis, ATPase component CcmA |
| CDS | contig00001 | 75055 | 75723 | 1 | + | 669 | ABC transporter involved in cytochrome c biogenesis, CcmB subunit |
| CDS | contig00001 | 75890 | 76630 | 2 | + | 741 | Cytochrome c-type biogenesis protein CcmC, putative heme lyase for CcmE |
| CDS | contig00001 | 76633 | 76839 | 1 | + | 207 | Cytochrome c-type biogenesis protein CcmD, interacts with CcmCE |
| CDS | contig00001 | 76836 | 77327 | 3 | + | 492 | Cytochrome c-type biogenesis protein CcmE, heme chaperone |
| CDS | contig00001 | 77407 | 79362 | 1 | + | 1956 | Cytochrome c heme lyase subunit CcmF |
| CDS | contig00001 | 79359 | 79889 | 3 | + | 531 | Cytochrome c-type biogenesis protein CcmG/DsbE, thiol:disulfide oxidoreductase |
| CDS | contig00001 | 79900 | 80382 | 1 | + | 483 | Cytochrome c heme lyase subunit CcmL |
| CDS | contig00001 | 80379 | 81641 | 3 | + | 1263 | Cytochrome c heme lyase subunit CcmH |
| CDS | contig00001 | 81720 | 82532 | 3 | + | 813 | Lipoprotein |
| CDS | contig00001 | 83227 | 82643 | -1 | - | 585 | Superoxide dismutase [Fe] (EC 1.15.1.1) |
| CDS | contig00001 | 83515 | 83859 | 1 | + | 345 | Probable monothiol glutaredoxin GrlA |
| CDS | contig00001 | 85138 | 83927 | -1 | - | 1212 | Sun protein |
| CDS | contig00001 | 85350 | 87317 | 3 | + | 1968 | hypothetical protein |
| CDS | contig00001 | 87314 | 87637 | 2 | + | 324 | FIG00361440: hypothetical protein |
| CDS | contig00001 | 87795 | 88433 | 3 | + | 639 | Pyridoxamine 5&#39;-phosphate oxidase (EC 1.4.3.5) |
| CDS | contig00001 | 88541 | 89323 | 2 | + | 783 | Cys regulon transcriptional activator CysB |
| CDS | contig00001 | 90798 | 89617 | -3 | - | 1182 | N-acetyl-L,L-diaminopimelate deacetylase (EC 3.5.1.47) |
| CDS | contig00001 | 91247 | 90798 | -2 | - | 450 | FIG00947233: hypothetical protein |
| CDS | contig00001 | 92062 | 91244 | -1 | - | 819 | membrane protein, putative |
| CDS | contig00001 | 92323 | 93513 | 1 | + | 1191 | putative acetyltransferase |
| CDS | contig00001 | 93666 | 94172 | 3 | + | 507 | Putative acetyltransferase |
| CDS | contig00001 | 94745 | 94245 | -2 | - | 501 | hypothetical protein |
| CDS | contig00001 | 94928 | 95368 | 2 | + | 441 | acetyltransferase (putative) |
| CDS | contig00001 | 96121 | 95501 | -1 | - | 621 | 4-Hydroxy-2-oxoglutarate aldolase (EC 4.1.3.16) @ 2-dehydro-3-deoxyphosphogluconate aldolase (EC 4.1.2.14) |
| CDS | contig00001 | 97061 | 96114 | -2 | - | 948 | 2-dehydro-3-deoxygluconate kinase (EC 2.7.1.45) |
| CDS | contig00001 | 97631 | 97083 | -2 | - | 549 | Inner membrane protein ygjV |
| CDS | contig00001 | 99115 | 97628 | -1 | - | 1488 | Altronate dehydratase (EC 4.2.1.7) |
| CDS | contig00001 | 100588 | 99125 | -1 | - | 1464 | Altronate oxidoreductase (EC 1.1.1.58) |
| CDS | contig00001 | 102123 | 100711 | -3 | - | 1413 | Uronate isomerase (EC 5.3.1.12) |
| CDS | contig00001 | 102496 | 102290 | -1 | - | 207 | hypothetical protein |
| CDS | contig00001 | 102560 | 103549 | 2 | + | 990 | TRAP-type C4-dicarboxylate transport system, periplasmic component |
| CDS | contig00001 | 103594 | 104115 | 1 | + | 522 | TRAP-type C4-dicarboxylate transport system, small permease component |
| CDS | contig00001 | 104115 | 105416 | 3 | + | 1302 | TRAP-type C4-dicarboxylate transport system, large permease component |
| CDS | contig00001 | 105547 | 106326 | 1 | + | 780 | Hexuronate utilization operon transcriptional repressor ExuR |
| CDS | contig00001 | 107580 | 107092 | -3 | - | 489 | tmRNA-binding protein SmpB |
| CDS | contig00001 | 107829 | 108263 | 3 | + | 435 | Putative oligoketide cyclase/lipid transport protein, similarity with yeast ubiquinone-binding protein YOL008W |
| CDS | contig00001 | 108256 | 108612 | 1 | + | 357 | UPF0125 protein yfjF |
| CDS | contig00001 | 109315 | 108680 | -1 | - | 636 | Nucleoside-diphosphate-sugar epimerases |
| CDS | contig00001 | 109754 | 109416 | -2 | - | 339 | Outer membrane lipoprotein SmpA, a component of the essential YaeT outer-membrane protein assembly complex |
| CDS | contig00001 | 111038 | 109887 | -2 | - | 1152 | Alcohol dehydrogenase (EC 1.1.1.1) |
| CDS | contig00001 | 111431 | 112186 | 2 | + | 756 | Uridine phosphorylase (EC 2.4.2.3) |
| CDS | contig00001 | 113025 | 112294 | -3 | - | 732 | Ribosomal large subunit pseudouridine synthase A (EC 4.2.1.70) |
| CDS | contig00001 | 113220 | 114155 | 3 | + | 936 | Inosine-uridine preferring nucleoside hydrolase (EC 3.2.2.1) |
| CDS | contig00001 | 114218 | 114661 | 2 | + | 444 | Sll1765 protein |
| CDS | contig00001 | 115054 | 114758 | -1 | - | 297 | FIG00361653: hypothetical protein |
| CDS | contig00001 | 115581 | 115051 | -3 | - | 531 | FIG00361612: hypothetical protein |
| CDS | contig00001 | 117382 | 115760 | -1 | - | 1623 | N-acetylglucosamine regulated methyl-accepting chemotaxis protein |
| CDS | contig00001 | 117547 | 118179 | 1 | + | 633 | Putative threonine efflux protein |
| CDS | contig00001 | 119409 | 118306 | -3 | - | 1104 | Hyaluronidase |
| CDS | contig00001 | 121182 | 119455 | -3 | - | 1728 | K+/H+ antiporter |
| CDS | contig00001 | 121596 | 121450 | -3 | - | 147 | hypothetical protein |
| CDS | contig00001 | 122282 | 121596 | -2 | - | 687 | FIG009095: D,D-carboxypeptidase family protein |
| CDS | contig00001 | 123439 | 122312 | -1 | - | 1128 | N-succinyl-L,L-diaminopimelate desuccinylase (EC 3.5.1.18) |
| CDS | contig00001 | 123812 | 123462 | -2 | - | 351 | FIG138056: a glutathione-dependent thiol reductase |
| CDS | contig00001 | 124453 | 124139 | -1 | - | 315 | FIG00361956: hypothetical protein |
| CDS | contig00001 | 124739 | 126115 | 2 | + | 1377 | Anaerobic C4-dicarboxylate transporter DcuC |
| CDS | contig00001 | 126253 | 127392 | 1 | + | 1140 | Acetylornithine deacetylase/Succinyl-diaminopimelate desuccinylase and related deacylases |
| CDS | contig00001 | 128119 | 127463 | -1 | - | 657 | conserved hypothetical protein [Pyrococcus horikoshii]; COG2102: Predicted ATPases of PP-loop superfamily; IPR002761: Domain of unknown function DUF71 |
| CDS | contig00001 | 129589 | 128246 | -1 | - | 1344 | anaerobic C4-dicarboxylate membrane transporter |
| CDS | contig00001 | 131341 | 129941 | -1 | - | 1401 | Asparaginyl-tRNA synthetase (EC 6.1.1.22) |
| CDS | contig00001 | 131579 | 131884 | 2 | + | 306 | hypothetical protein |
| CDS | contig00001 | 132693 | 131872 | -3 | - | 822 | Methyl-accepting chemotaxis protein |
| CDS | contig00001 | 134103 | 132829 | -3 | - | 1275 | Adenosylmethionine-8-amino-7-oxononanoate aminotransferase (EC 2.6.1.62) |
| CDS | contig00001 | 134323 | 134168 | -1 | - | 156 | hypothetical protein |
| CDS | contig00001 | 134297 | 135385 | 2 | + | 1089 | Biotin synthase (EC 2.8.1.6) |
| CDS | contig00001 | 135382 | 136578 | 1 | + | 1197 | 8-amino-7-oxononanoate synthase (EC 2.3.1.47) |
| CDS | contig00001 | 136568 | 137371 | 2 | + | 804 | Biotin synthesis protein BioC |
| CDS | contig00001 | 137480 | 138160 | 2 | + | 681 | Dethiobiotin synthetase (EC 6.3.3.3) |
| CDS | contig00001 | 138274 | 139146 | 1 | + | 873 | Probable protease htpX homolog (EC 3.4.24.-) |
| CDS | contig00001 | 140921 | 139227 | -2 | - | 1695 | Flavodoxin reductases (ferredoxin-NADPH reductases) family 1 |
| CDS | contig00001 | 141358 | 140954 | -1 | - | 405 | hypothetical protein |
| CDS | contig00001 | 141380 | 143026 | 2 | + | 1647 | Pyridine nucleotide-disulphide oxidoreductase family protein associated with PFOR |
| CDS | contig00001 | 143023 | 146598 | 1 | + | 3576 | Pyruvate-flavodoxin oxidoreductase (EC 1.2.7.-) |
| CDS | contig00001 | 146976 | 146761 | -3 | - | 216 | FIG00362307: hypothetical protein |
| CDS | contig00001 | 147843 | 147253 | -3 | - | 591 | FIG00361489: hypothetical protein |
| CDS | contig00001 | 149201 | 147855 | -2 | - | 1347 | Na+ driven multidrug efflux pump |
| CDS | contig00001 | 149184 | 149309 | 3 | + | 126 | hypothetical protein |
| CDS | contig00001 | 151147 | 149324 | -1 | - | 1824 | Putative sulfate permease |
| CDS | contig00001 | 151766 | 151218 | -2 | - | 549 | FIG00361446: hypothetical protein |
| CDS | contig00001 | 152049 | 151792 | -3 | - | 258 | Glutamate synthase [NADPH] large chain (EC 1.4.1.13) |
| CDS | contig00001 | 152204 | 153094 | 2 | + | 891 | Cysteine synthase (EC 2.5.1.47) |
| CDS | contig00001 | 154294 | 153110 | -1 | - | 1185 | Alcohol dehydrogenase (EC 1.1.1.1) |
| CDS | contig00001 | 154838 | 154311 | -2 | - | 528 | hypothetical protein |
| CDS | contig00001 | 155418 | 154828 | -3 | - | 591 | Transcriptional regulator, TetR family |
| CDS | contig00001 | 156857 | 155427 | -2 | - | 1431 | Coniferyl aldehyde dehydrogenase (EC 1.2.1.68) |
| CDS | contig00001 | 157070 | 157363 | 2 | + | 294 | FIG00361208: hypothetical protein |
| CDS | contig00001 | 157900 | 157418 | -1 | - | 483 | Lipoprotein NlpC |
| CDS | contig00001 | 158888 | 157974 | -2 | - | 915 | Hydrogen peroxide-inducible genes activator |
| CDS | contig00001 | 160151 | 159186 | -2 | - | 966 | FIG002781: Alpha-L-glutamate ligase family protein |
| CDS | contig00001 | 161693 | 160155 | -2 | - | 1539 | FIG139976: hypothetical protein |
| CDS | contig00001 | 162485 | 161697 | -2 | - | 789 | FIG008443: hypothetical protein |
| CDS | contig00001 | 163515 | 162538 | -3 | - | 978 | tRNA (5-methoxyuridine) 34 synthase |
| CDS | contig00001 | 164255 | 163512 | -2 | - | 744 | tRNA (uridine-5-oxyacetic acid methyl ester) 34 synthase |
| CDS | contig00001 | 165437 | 164589 | -2 | - | 849 | FIG003003: hypothetical protein |
| CDS | contig00001 | 165866 | 167632 | 2 | + | 1767 | Aspartyl-tRNA synthetase (EC 6.1.1.12) |
| CDS | contig00001 | 167733 | 168473 | 3 | + | 741 | FIG000859: hypothetical protein YebC |
| CDS | contig00001 | 168908 | 170533 | 2 | + | 1626 | Phosphoethanolamine transferase EptA specific for the 1 phosphate group of core-lipid A |
| CDS | contig00001 | 171817 | 170603 | -1 | - | 1215 | N-acetylglucosamine-6P-responsive transcriptional repressor NagC, ROK family |
| CDS | contig00001 | 172967 | 171822 | -2 | - | 1146 | N-acetylglucosamine-6-phosphate deacetylase (EC 3.5.1.25) |
| CDS | contig00001 | 173777 | 172977 | -2 | - | 801 | Glucosamine-6-phosphate deaminase (EC 3.5.99.6) |
| CDS | contig00001 | 174050 | 175504 | 2 | + | 1455 | PTS system, N-acetylglucosamine-specific IIA component/ PTS system, N-acetylglucosamine-specific IIB component/ PTS system, N-acetylglucosamine-specific IIC component |
| CDS | contig00001 | 175627 | 178290 | 1 | + | 2664 | Beta-hexosaminidase (EC 3.2.1.52) |
| CDS | contig00001 | 178629 | 180290 | 3 | + | 1662 | Glutaminyl-tRNA synthetase (EC 6.1.1.18) |
| CDS | contig00001 | 180818 | 180390 | -2 | - | 429 | Ferric uptake regulation protein FUR |
| CDS | contig00001 | 181050 | 181523 | 3 | + | 474 | FIG01200136: hypothetical protein |
| CDS | contig00001 | 182116 | 181592 | -1 | - | 525 | Flavodoxin 1 |
| CDS | contig00001 | 182453 | 182157 | -2 | - | 297 | Uncharacterized protein ybfE |
| CDS | contig00001 | 182695 | 182474 | -1 | - | 222 | FIG01057153: hypothetical protein |
| CDS | contig00001 | 183533 | 182751 | -2 | - | 783 | Esterase ybfF (EC 3.1.-.-) |
| CDS | contig00001 | 183528 | 183767 | 3 | + | 240 | hypothetical protein |
| CDS | contig00001 | 183806 | 184315 | 2 | + | 510 | SeqA protein, negative modulator of initiation of replication |
| CDS | contig00001 | 184336 | 186018 | 1 | + | 1683 | Phosphoglucomutase (EC 5.4.2.2) |
| CDS | contig00001 | 186973 | 186092 | -1 | - | 882 | Permease of the drug/metabolite transporter (DMT) superfamily |
| CDS | contig00001 | 187068 | 187550 | 3 | + | 483 | Transcriptional regulator, MarR family |
| CDS | contig00001 | 188693 | 187596 | -2 | - | 1098 | Membrane-bound lytic murein transglycosylase B precursor (EC 3.2.1.-) |
| CDS | contig00001 | 189390 | 188788 | -3 | - | 603 | Putative phosphatidylglycerophosphate synthase |
| CDS | contig00001 | 190045 | 189383 | -1 | - | 663 | ABC transporter, ATP-binding protein YnjD |
| CDS | contig00001 | 191702 | 190050 | -2 | - | 1653 | ABC transporter, permease protein YnjC |
| CDS | contig00001 | 192878 | 191709 | -2 | - | 1170 | ABC transporter, periplasmic substrate-binding protein YnjB |
| CDS | contig00001 | 195054 | 192898 | -3 | - | 2157 | COG0398: uncharacterized membrane protein / PF00070 family, FAD-dependent NAD(P)-disulphide oxidoreductase |
| CDS | contig00001 | 195626 | 195051 | -2 | - | 576 | hypothetical protein |
| CDS | contig00001 | 196326 | 195616 | -3 | - | 711 | Bll2618 protein |
| CDS | contig00001 | 196732 | 196454 | -1 | - | 279 | Peptidyl-prolyl cis-trans isomerase PpiC (EC 5.2.1.8) |
| CDS | contig00001 | 197187 | 196792 | -3 | - | 396 | hypothetical protein |
| CDS | contig00001 | 197799 | 199499 | 3 | + | 1701 | Uncharacterized protein YtfM precursor |
| CDS | contig00001 | 199610 | 199494 | -2 | - | 117 | hypothetical protein |
| CDS | contig00001 | 199609 | 203349 | 1 | + | 3741 | Uncharacterized protein YtfN |
| CDS | contig00001 | 203929 | 204393 | 1 | + | 465 | hypothetical protein |
| CDS | contig00001 | 204777 | 204457 | -3 | - | 321 | Ribosome hibernation protein YfiA |
| CDS | contig00001 | 205229 | 205086 | -2 | - | 144 | hypothetical protein |
| CDS | contig00001 | 205182 | 207701 | 3 | + | 2520 | diguanylate cyclase/phosphodiesterase (GGDEF & EAL domains) with PAS/PAC sensor(s) |
| CDS | contig00001 | 207731 | 209701 | 2 | + | 1971 | Soluble lytic murein transglycosylase precursor (EC 3.2.1.-) |
| CDS | contig00001 | 209879 | 210877 | 2 | + | 999 | Galactose operon repressor, GalR-LacI family of transcriptional regulators |
| CDS | contig00001 | 211124 | 211936 | 2 | + | 813 | Nucleoside-diphosphate-sugar epimerases |
| CDS | contig00001 | 212076 | 212474 | 3 | + | 399 | Acyl-CoA thioesterase YciA, involved in membrane biogenesis |
| CDS | contig00001 | 212645 | 212481 | -2 | - | 165 | hypothetical protein |
| CDS | contig00001 | 212817 | 213662 | 3 | + | 846 | FIG00613320: hypothetical protein |
| CDS | contig00001 | 215152 | 213758 | -1 | - | 1395 | Membrane-bound lytic murein transglycosylase D precursor (EC 3.2.1.-) |
| CDS | contig00001 | 215162 | 215281 | 2 | + | 120 | hypothetical protein |
| CDS | contig00001 | 216133 | 215369 | -1 | - | 765 | Hydroxyacylglutathione hydrolase (EC 3.1.2.6) |
| CDS | contig00001 | 216213 | 216935 | 3 | + | 723 | FIG005121: SAM-dependent methyltransferase (EC 2.1.1.-) |
| CDS | contig00001 | 217331 | 216942 | -2 | - | 390 | Ribonuclease HI (EC 3.1.26.4) |
| CDS | contig00001 | 217482 | 218216 | 3 | + | 735 | DNA polymerase III epsilon subunit (EC 2.7.7.7) |
| CDS | contig00001 | 218204 | 219463 | 2 | + | 1260 | FIG004599: Hypothetical protein |
| CDS | contig00001 | 220441 | 219539 | -1 | - | 903 | Response regulator |
| CDS | contig00001 | 220433 | 220648 | 2 | + | 216 | hypothetical protein |
| CDS | contig00001 | 221054 | 220614 | -2 | - | 441 | Response regulator |
| CDS | contig00001 | 223685 | 221229 | -2 | - | 2457 | Acyl-CoA dehydrogenase, short-chain specific (EC 1.3.99.2) |
| CDS | contig00001 | 223938 | 224516 | 3 | + | 579 | Phosphoheptose isomerase 1 (EC 5.3.1.-) |
| CDS | contig00001 | 224629 | 225426 | 1 | + | 798 | Predicted glutamine amidotransferase |
| CDS | contig00001 | 225727 | 226062 | 1 | + | 336 | Phosphorelay protein luxU |
| CDS | contig00001 | 227031 | 226075 | -3 | - | 957 | FIG002813: LPPG:FO 2-phospho-L-lactate transferase like, CofD-like |
| CDS | contig00001 | 227217 | 228293 | 3 | + | 1077 | FIG00921036: hypothetical protein |
| CDS | contig00001 | 228312 | 230387 | 3 | + | 2076 | Sensor histidine kinase/response regulator |
| CDS | contig00001 | 230463 | 230341 | -3 | - | 123 | hypothetical protein |
| CDS | contig00001 | 231235 | 230660 | -1 | - | 576 | Uncharacterized protein, similar to the N-terminal domain of Lon protease |
| CDS | contig00001 | 231963 | 231538 | -3 | - | 426 | Putative bacterial haemoglobin |
| CDS | contig00001 | 232748 | 232110 | -2 | - | 639 | Nitrate/nitrite response regulator protein |
| CDS | contig00001 | 234460 | 232748 | -1 | - | 1713 | Nitrate/nitrite sensor protein (EC 2.7.3.-) |
| CDS | contig00001 | 234633 | 235661 | 3 | + | 1029 | Molybdenum cofactor biosynthesis protein MoaA |
| CDS | contig00001 | 235913 | 236422 | 2 | + | 510 | Ferredoxin-type protein NapF (periplasmic nitrate reductase) |
| CDS | contig00001 | 236440 | 236721 | 1 | + | 282 | Periplasmic nitrate reductase component NapD |
| CDS | contig00001 | 236911 | 239400 | 1 | + | 2490 | Periplasmic nitrate reductase precursor (EC 1.7.99.4) |
| CDS | contig00001 | 239417 | 240151 | 2 | + | 735 | Ferredoxin-type protein NapG (periplasmic nitrate reductase) |
| CDS | contig00001 | 240148 | 241023 | 1 | + | 876 | Polyferredoxin NapH (periplasmic nitrate reductase) |
| CDS | contig00001 | 241023 | 241478 | 3 | + | 456 | Nitrate reductase cytochrome c550-type subunit |
| CDS | contig00001 | 241490 | 242080 | 2 | + | 591 | Cytochrome c-type protein NapC |
| CDS | contig00001 | 242274 | 242780 | 3 | + | 507 | Molybdenum cofactor biosynthesis protein MoaB |
| CDS | contig00001 | 242831 | 243325 | 2 | + | 495 | Molybdenum cofactor biosynthesis protein MoaC |
| CDS | contig00001 | 243322 | 243567 | 1 | + | 246 | Molybdenum cofactor biosynthesis protein MoaD |
| CDS | contig00001 | 243571 | 244050 | 1 | + | 480 | Molybdenum cofactor biosynthesis protein MoaE |
| CDS | contig00001 | 244105 | 244833 | 1 | + | 729 | Molybdenum ABC transporter, periplasmic molybdenum-binding protein ModA (TC 3.A.1.8.1) |
| CDS | contig00001 | 244950 | 245630 | 3 | + | 681 | Molybdenum transport system permease protein ModB (TC 3.A.1.8.1) |
| CDS | contig00001 | 245627 | 246682 | 2 | + | 1056 | Molybdenum transport ATP-binding protein ModC (TC 3.A.1.8.1) |
| CDS | contig00001 | 246717 | 247526 | 3 | + | 810 | Beta-ketoadipate enol-lactone hydrolase (EC 3.1.1.24) |
| CDS | contig00001 | 248349 | 247594 | -3 | - | 756 | Molybdopterin biosynthesis protein MoeB |
| CDS | contig00001 | 249578 | 248346 | -2 | - | 1233 | Molybdopterin biosynthesis protein MoeA |
| CDS | contig00001 | 249970 | 250599 | 1 | + | 630 | GTP cyclohydrolase I (EC 3.5.4.16) type 1 |
| CDS | contig00001 | 250787 | 252499 | 2 | + | 1713 | Methyl-accepting chemotaxis protein |
| CDS | contig00001 | 254469 | 252736 | -3 | - | 1734 | Formate--tetrahydrofolate ligase (EC 6.3.4.3) |
| CDS | contig00001 | 254536 | 254937 | 1 | + | 402 | FIG00362184: hypothetical protein |
| CDS | contig00001 | 255848 | 254946 | -2 | - | 903 | Transcriptional regulator, LysR family |
| CDS | contig00001 | 255958 | 256518 | 1 | + | 561 | Protein yceI precursor |
| CDS | contig00001 | 256518 | 257252 | 3 | + | 735 | FIG00361990: hypothetical protein |
| CDS | contig00001 | 257438 | 258559 | 2 | + | 1122 | Adenosylhomocysteinase (EC 3.3.1.1) |
| CDS | contig00001 | 258556 | 258867 | 1 | + | 312 | SelT/selW/selH selenoprotein domain |
| CDS | contig00001 | 260655 | 258931 | -3 | - | 1725 | Putative hemolysin |
| CDS | contig00001 | 261987 | 260761 | -3 | - | 1227 | Tripeptide aminopeptidase (EC 3.4.11.4) |
| CDS | contig00001 | 262021 | 262149 | 1 | + | 129 | hypothetical protein |
| CDS | contig00001 | 263040 | 264914 | 3 | + | 1875 | Biosynthetic arginine decarboxylase (EC 4.1.1.19) |
| CDS | contig00001 | 265171 | 265359 | 1 | + | 189 | FIG00361718: hypothetical protein |
| CDS | contig00001 | 265692 | 265405 | -3 | - | 288 | hypothetical protein |
| CDS | contig00001 | 267964 | 265856 | -1 | - | 2109 | diguanylate cyclase/phosphodiesterase (GGDEF & EAL domains) with PAS/PAC sensor(s) |
| CDS | contig00001 | 268414 | 269001 | 1 | + | 588 | Phosphoglycerate mutase family 4 |
| CDS | contig00001 | 272193 | 269050 | -3 | - | 3144 | helicase |
| CDS | contig00001 | 272347 | 272814 | 1 | + | 468 | hypothetical protein |
| CDS | contig00001 | 272925 | 273293 | 3 | + | 369 | biphenyl-2,3-diol 1,2-dioxygenase III-related protein |
| CDS | contig00001 | 273566 | 273318 | -2 | - | 249 | hypothetical protein |
| CDS | contig00001 | 275008 | 273665 | -1 | - | 1344 | NADP-specific glutamate dehydrogenase (EC 1.4.1.4) |
| CDS | contig00001 | 275579 | 276592 | 2 | + | 1014 | Ribosomal large subunit pseudouridine synthase F (EC 4.2.1.70) |
| CDS | contig00001 | 277911 | 276679 | -3 | - | 1233 | Cyanate transport protein CynX |
| CDS | contig00001 | 278407 | 277904 | -1 | - | 504 | Probable deaminase |
| CDS | contig00001 | 278517 | 279398 | 3 | + | 882 | Transcriptional regulator, LysR family |
| CDS | contig00001 | 279882 | 279415 | -3 | - | 468 | Transcriptional regulator, AraC family |
| CDS | contig00001 | 280058 | 279903 | -2 | - | 156 | hypothetical protein |
| CDS | contig00001 | 280610 | 280143 | -2 | - | 468 | Acyl-CoA hydrolase (EC 3.1.2.20) |
| CDS | contig00001 | 280784 | 281434 | 2 | + | 651 | putative membrane protein |
| CDS | contig00001 | 281594 | 282061 | 2 | + | 468 | acetyltransferase, GNAT family |
| CDS | contig00001 | 283536 | 282088 | -3 | - | 1449 | Transcriptional regulator, GntR family domain / Aspartate aminotransferase (EC 2.6.1.1) |
| CDS | contig00001 | 283657 | 285228 | 1 | + | 1572 | Cytochrome d ubiquinol oxidase subunit I (EC 1.10.3.-) |
| CDS | contig00001 | 285228 | 285881 | 3 | + | 654 | Cytochrome d ubiquinol oxidase subunit II (EC 1.10.3.-) |
| CDS | contig00001 | 285891 | 286355 | 3 | + | 465 | Cytochrome d ubiquinol oxidase subunit II (EC 1.10.3.-) |
| CDS | contig00001 | 286384 | 286497 | 1 | + | 114 | hypothetical protein |
| CDS | contig00001 | 287730 | 286561 | -3 | - | 1170 | Putative drug efflux protein |
| CDS | contig00001 | 288928 | 288125 | -1 | - | 804 | Methylglyoxal reductase, acetol producing (EC 1.1.1.-) / 2,5-diketo-D-gluconic acid reductase B (EC 1.1.1.274) |
| CDS | contig00001 | 289309 | 289019 | -1 | - | 291 | hypothetical protein |
| CDS | contig00001 | 289828 | 289463 | -1 | - | 366 | Glyoxalase family protein |
| CDS | contig00001 | 290241 | 289855 | -3 | - | 387 | hypothetical protein |
| CDS | contig00001 | 290410 | 291312 | 1 | + | 903 | Transcriptional regulator, LysR family |
| CDS | contig00001 | 293023 | 291494 | -1 | - | 1530 | Glycerol kinase (EC 2.7.1.30) |
| CDS | contig00001 | 293899 | 293051 | -1 | - | 849 | Glycerol uptake facilitator protein |
| CDS | contig00001 | 293888 | 294001 | 2 | + | 114 | hypothetical protein |
| CDS | contig00001 | 294390 | 295745 | 3 | + | 1356 | Glycerol-3-phosphate transporter |
| CDS | contig00001 | 295847 | 296605 | 2 | + | 759 | Glycerol-3-phosphate regulon repressor, DeoR family |
| CDS | contig00001 | 297984 | 299471 | 3 | + | 1488 | Aerobic glycerol-3-phosphate dehydrogenase (EC 1.1.5.3) |
| CDS | contig00001 | 299574 | 300653 | 3 | + | 1080 | Glycerophosphoryl diester phosphodiesterase (EC 3.1.4.46) |
| CDS | contig00001 | 301025 | 302098 | 2 | + | 1074 | 2-keto-3-deoxy-D-arabino-heptulosonate-7-phosphate synthase I alpha (EC 2.5.1.54) |
| CDS | contig00001 | 302217 | 303356 | 3 | + | 1140 | Chorismate mutase I/Cyclohexadienyl dehydrogenase |
| CDS | contig00001 | 304346 | 303471 | -2 | - | 876 | Transcriptional regulator, LysR family |
| CDS | contig00001 | 304702 | 304346 | -1 | - | 357 | Uncharacterized protein conserved in bacteria |
| CDS | contig00001 | 305476 | 304994 | -1 | - | 483 | Proteinase inhibitor I11, ecotin precursor |
| CDS | contig00001 | 306228 | 305671 | -3 | - | 558 | Hypothetical protein in cluster with HutR, VCA0067 homolog |
| CDS | contig00001 | 307592 | 306225 | -2 | - | 1368 | Hypothetical protein in cluster with HutR, VCA0066 homolog |
| CDS | contig00001 | 310054 | 307643 | -1 | - | 2412 | Hypothetical with regulatory P domain of a subtilisin-like proprotein convertase |
| CDS | contig00001 | 312126 | 310084 | -3 | - | 2043 | Protease II (EC 3.4.21.83) |
| CDS | contig00001 | 314294 | 312126 | -2 | - | 2169 | TonB-dependent heme receptor HutR |
| CDS | contig00001 | 316391 | 314544 | -2 | - | 1848 | FIG001881: hydrolase of alkaline phosphatase superfamily |
| CDS | contig00001 | 316662 | 316435 | -3 | - | 228 | FIG002927: hypothetical protein |
| CDS | contig00001 | 316736 | 317785 | 2 | + | 1050 | Nucleoid-associated protein NdpA |
| CDS | contig00001 | 318182 | 319363 | 2 | + | 1182 | Maltose/maltodextrin ABC transporter, substrate binding periplasmic protein MalE |
| CDS | contig00001 | 319437 | 321092 | 3 | + | 1656 | Maltose/maltodextrin ABC transporter, permease protein MalF |
| CDS | contig00001 | 321105 | 321995 | 3 | + | 891 | Maltose/maltodextrin ABC transporter, permease protein MalG |
| CDS | contig00001 | 322066 | 323202 | 1 | + | 1137 | Maltose/maltodextrin transport ATP-binding protein MalK (EC 3.6.3.19) |
| CDS | contig00001 | 324160 | 323330 | -1 | - | 831 | FIG01199622: hypothetical protein |
| CDS | contig00001 | 324317 | 325123 | 2 | + | 807 | Beta-ketoadipate enol-lactone hydrolase (EC 3.1.1.24) |
| CDS | contig00001 | 325999 | 325190 | -1 | - | 810 | MltA-interacting protein MipA |
| CDS | contig00001 | 327243 | 326173 | -3 | - | 1071 | Putative permease PerM (= YfgO) |
| CDS | contig00001 | 327535 | 327266 | -1 | - | 270 | FIG028274: hypothetical protein |
| CDS | contig00001 | 327597 | 329117 | 3 | + | 1521 | Exported zinc metalloprotease YfgC precursor |
| CDS | contig00001 | 329706 | 329251 | -3 | - | 456 | Thioredoxin |
| CDS | contig00001 | 329791 | 330141 | 1 | + | 351 | Arsenate reductase (EC 1.20.4.1) |
| CDS | contig00001 | 330138 | 330512 | 3 | + | 375 | FIG00920275: hypothetical protein |
| CDS | contig00001 | 330761 | 331831 | 2 | + | 1071 | Choloylglycine hydrolase (EC 3.5.1.24) |
| CDS | contig00001 | 332481 | 331870 | -3 | - | 612 | Acyl carrier protein phosphodiesterase (EC 3.1.4.14) |
| CDS | contig00001 | 332867 | 333646 | 2 | + | 780 | Lysine-arginine-ornithine-binding periplasmic protein precursor (TC 3.A.1.3.1) |
| CDS | contig00001 | 333705 | 334835 | 3 | + | 1131 | Isoaspartyl dipeptidase (EC 3.4.19.5) @ Asp-X dipeptidase |
| CDS | contig00001 | 335819 | 334917 | -2 | - | 903 | Putative membrane protein |
| CDS | contig00001 | 336352 | 337122 | 1 | + | 771 | Histidine transport ATP-binding protein HisP (TC 3.A.1.3.1) |
| CDS | contig00001 | 337188 | 337961 | 3 | + | 774 | Lysine-arginine-ornithine-binding periplasmic protein precursor (TC 3.A.1.3.1) |
| CDS | contig00001 | 338067 | 338807 | 3 | + | 741 | Arginine/ornithine ABC transporter, permease protein AotQ |
| CDS | contig00001 | 338811 | 339503 | 3 | + | 693 | Histidine ABC transporter, permease protein HisM (TC 3.A.1.3.1) |
| CDS | contig00001 | 340202 | 339561 | -2 | - | 642 | Arginine/ornithine antiporter ArcD |
| CDS | contig00001 | 341142 | 340300 | -3 | - | 843 | Pyruvate formate-lyase activating enzyme (EC 1.97.1.4) |
| CDS | contig00001 | 344157 | 341218 | -3 | - | 2940 | diguanylate cyclase/phosphodiesterase (GGDEF & EAL domains) with PAS/PAC sensor(s) |
| CDS | contig00001 | 344292 | 345980 | 3 | + | 1689 | CoA-disulfide reductase (EC 1.8.1.14) |
| CDS | contig00001 | 347242 | 346148 | -1 | - | 1095 | Putative membrane protein |
| CDS | contig00001 | 347273 | 347407 | 2 | + | 135 | hypothetical protein |
| CDS | contig00001 | 347737 | 352581 | 1 | + | 4845 | Alpha-2-macroglobulin |
| CDS | contig00001 | 352793 | 354955 | 2 | + | 2163 | Penicillin-insensitive transglycosylase (EC 2.4.2.-) & transpeptidase PBP-1C |
| CDS | contig00001 | 356006 | 355044 | -2 | - | 963 | FIG00361258: hypothetical protein |
| CDS | contig00001 | 357275 | 356028 | -2 | - | 1248 | Phage integrase |
| CDS | contig00001 | 357415 | 357275 | -1 | - | 141 | hypothetical protein |
| CDS | contig00001 | 357855 | 357445 | -3 | - | 411 | COG0840: Methyl-accepting chemotaxis protein |
| CDS | contig00001 | 358276 | 357992 | -1 | - | 285 | hypothetical protein |
| CDS | contig00001 | 358831 | 358307 | -1 | - | 525 | hypothetical protein |
| CDS | contig00001 | 360617 | 359985 | -2 | - | 633 | hypothetical protein |
| CDS | contig00001 | 361552 | 360614 | -1 | - | 939 | DNA recombination-dependent growth factor C |
| CDS | contig00001 | 361695 | 361573 | -3 | - | 123 | hypothetical protein |
| CDS | contig00001 | 362390 | 361812 | -2 | - | 579 | putative DNA N-6-adenine methyltransferase |
| CDS | contig00001 | 362598 | 362383 | -3 | - | 216 | Phage EaA protein |
| CDS | contig00001 | 364055 | 362835 | -2 | - | 1221 | hypothetical protein |
| CDS | contig00001 | 364245 | 364069 | -3 | - | 177 | hypothetical protein |
| CDS | contig00001 | 365356 | 364253 | -1 | - | 1104 | TolA protein |
| CDS | contig00001 | 365309 | 365434 | 2 | + | 126 | hypothetical protein |
| CDS | contig00001 | 366383 | 365406 | -2 | - | 978 | FIG01078329: hypothetical protein |
| CDS | contig00001 | 367225 | 366380 | -1 | - | 846 | Exodeoxyribonuclease VIII (EC 3.1.11.-) |
| CDS | contig00001 | 367979 | 367614 | -2 | - | 366 | hypothetical protein |
| CDS | contig00001 | 368173 | 367982 | -1 | - | 192 | hypothetical protein |
| CDS | contig00001 | 368788 | 368543 | -1 | - | 246 | hypothetical protein |
| CDS | contig00001 | 370222 | 369914 | -1 | - | 309 | putative prophage repressor CI |
| CDS | contig00001 | 370934 | 371632 | 2 | + | 699 | Phage DNA binding protein Roi |
| CDS | contig00001 | 371707 | 372063 | 1 | + | 357 | hypothetical protein |
| CDS | contig00001 | 372068 | 372286 | 2 | + | 219 | hypothetical protein |
| CDS | contig00001 | 372352 | 372492 | 1 | + | 141 | hypothetical protein |
| CDS | contig00001 | 372485 | 374182 | 2 | + | 1698 | Modification methylase (EC 2.1.1.37) |
| CDS | contig00001 | 374245 | 375192 | 1 | + | 948 | Origin specific replication initiation factor #replication protein O |
| CDS | contig00001 | 375192 | 375863 | 3 | + | 672 | Replication protein P |
| CDS | contig00001 | 375943 | 376152 | 1 | + | 210 | hypothetical protein |
| CDS | contig00001 | 376291 | 376521 | 1 | + | 231 | hypothetical protein |
| CDS | contig00001 | 376518 | 376697 | 3 | + | 180 | hypothetical protein |
| CDS | contig00001 | 376928 | 377044 | 2 | + | 117 | hypothetical protein |
| CDS | contig00001 | 377041 | 377481 | 1 | + | 441 | Phage NinB DNA recombination |
| CDS | contig00001 | 377733 | 378137 | 3 | + | 405 | hypothetical protein |
| CDS | contig00001 | 378260 | 378451 | 2 | + | 192 | hypothetical protein |
| CDS | contig00001 | 378579 | 379001 | 3 | + | 423 | phage-related hypothetical protein |
| CDS | contig00001 | 379154 | 379696 | 2 | + | 543 | hypothetical protein |
| CDS | contig00001 | 379865 | 380179 | 2 | + | 315 | hypothetical protein |
| CDS | contig00001 | 380188 | 380661 | 1 | + | 474 | FIG00641052: hypothetical protein |
| CDS | contig00001 | 380715 | 380939 | 3 | + | 225 | hypothetical protein |
| CDS | contig00001 | 381019 | 381171 | 1 | + | 153 | hypothetical protein |
| CDS | contig00001 | 381225 | 381404 | 3 | + | 180 | hypothetical protein |
| CDS | contig00001 | 381600 | 382277 | 3 | + | 678 | DNA packaging |
| CDS | contig00001 | 382361 | 383974 | 2 | + | 1614 | Phage protein |
| CDS | contig00001 | 383974 | 386100 | 1 | + | 2127 | FIG01201709: hypothetical protein |
| CDS | contig00001 | 386221 | 386556 | 1 | + | 336 | gene 66 protein |
| CDS | contig00001 | 386688 | 387614 | 3 | + | 927 | Phage protein |
| CDS | contig00001 | 387686 | 388903 | 2 | + | 1218 | conserved phage protein |
| CDS | contig00001 | 388969 | 389358 | 1 | + | 390 | hypothetical protein |
| CDS | contig00001 | 389425 | 389862 | 1 | + | 438 | Phage protein |
| CDS | contig00001 | 389864 | 390445 | 2 | + | 582 | Phage protein |
| CDS | contig00001 | 390458 | 391135 | 2 | + | 678 | Phage protein |
| CDS | contig00001 | 391145 | 392881 | 2 | + | 1737 | hypothetical protein |
| CDS | contig00001 | 392943 | 393287 | 3 | + | 345 | hypothetical protein |
| CDS | contig00001 | 393557 | 394576 | 2 | + | 1020 | Retron-type RNA-directed DNA polymerase (EC 2.7.7.49) |
| CDS | contig00001 | 394624 | 394854 | 1 | + | 231 | Phage protein |
| CDS | contig00001 | 395062 | 396723 | 1 | + | 1662 | FIG00640812: hypothetical protein |
| CDS | contig00001 | 396723 | 400691 | 3 | + | 3969 | Phage tail fiber protein |
| CDS | contig00001 | 400688 | 401050 | 2 | + | 363 | Phage protein |
| CDS | contig00001 | 401059 | 401697 | 1 | + | 639 | Phage protein |
| CDS | contig00001 | 401798 | 402082 | 2 | + | 285 | Phage protein |
| CDS | contig00001 | 402082 | 402339 | 1 | + | 258 | Phage protein |
| CDS | contig00001 | 402364 | 403749 | 1 | + | 1386 | Phage protein |
| CDS | contig00001 | 404060 | 415180 | 2 | + | 11121 | hypothetical protein |
| CDS | contig00001 | 415254 | 415457 | 3 | + | 204 | hypothetical protein |
| CDS | contig00001 | 415616 | 415738 | 2 | + | 123 | hypothetical protein |
| CDS | contig00001 | 416180 | 417628 | 2 | + | 1449 | tRNA S(4)U 4-thiouridine synthase (former ThiI) / Rhodanese-like domain required for thiamine synthesis |
| CDS | contig00001 | 418096 | 417704 | -1 | - | 393 | FIG00361864: hypothetical protein |
| CDS | contig00001 | 418830 | 418198 | -3 | - | 633 | Putative inner membrane protein |
| CDS | contig00001 | 419250 | 420068 | 3 | + | 819 | Flagellin protein FlaA |
| CDS | contig00001 | 420105 | 420560 | 3 | + | 456 | Flagellin protein FlaG |
| CDS | contig00001 | 420589 | 421968 | 1 | + | 1380 | Flagellar hook-associated protein FliD |
| CDS | contig00001 | 421994 | 422419 | 2 | + | 426 | Flagellar biosynthesis protein FliS |
| CDS | contig00001 | 422697 | 422545 | -3 | - | 153 | hypothetical protein |
| CDS | contig00001 | 423743 | 422886 | -2 | - | 858 | Vitamin B12 ABC transporter, B12-binding component BtuF |
| CDS | contig00001 | 424698 | 423736 | -3 | - | 963 | Adenosylcobinamide-phosphate synthase (EC 6.3.1.10) |
| CDS | contig00001 | 425387 | 424695 | -2 | - | 693 | 5&#39;-methylthioadenosine nucleosidase (EC 3.2.2.16) / S-adenosylhomocysteine nucleosidase (EC 3.2.2.9) |
| CDS | contig00001 | 425603 | 425487 | -2 | - | 117 | hypothetical protein |
| CDS | contig00001 | 426546 | 425617 | -3 | - | 930 | Metal transporter, ZIP family |
| CDS | contig00001 | 426842 | 427537 | 2 | + | 696 | Methyl-accepting chemotaxis protein I (serine chemoreceptor protein) |
| CDS | contig00002 | 2566 | 1997 | -1 | - | 570 | Site-specific recombinase, resolvase family |
| CDS | contig00002 | 3444 | 2758 | -3 | - | 687 | hypothetical protein |
| CDS | contig00002 | 5621 | 6970 | 2 | + | 1350 | TldE protein, part of TldE/TldD proteolytic complex |
| CDS | contig00002 | 7303 | 7419 | 1 | + | 117 | hypothetical protein |
| CDS | contig00002 | 7555 | 7421 | -1 | - | 135 | hypothetical protein |
| CDS | contig00002 | 8777 | 7614 | -2 | - | 1164 | Uncharacterized protein EC-HemY, likely associated with heme metabolism based on gene clustering with hemC, hemD in Proteobacteria (unrelated to HemY-type PPO in GramPositives) |
| CDS | contig00002 | 9864 | 8788 | -3 | - | 1077 | Uroporphyrinogen-III methyltransferase (EC 2.1.1.107) |
| CDS | contig00002 | 10595 | 9861 | -2 | - | 735 | Uroporphyrinogen-III synthase (EC 4.2.1.75) |
| CDS | contig00002 | 11589 | 10642 | -3 | - | 948 | Porphobilinogen deaminase (EC 2.5.1.61) |
| CDS | contig00002 | 11620 | 11751 | 1 | + | 132 | hypothetical protein |
| CDS | contig00002 | 12001 | 11867 | -1 | - | 135 | hypothetical protein |
| CDS | contig00002 | 11894 | 14413 | 2 | + | 2520 | Adenylate cyclase (EC 4.6.1.1) |
| CDS | contig00002 | 15143 | 14490 | -2 | - | 654 | Carboxylesterase |
| CDS | contig00002 | 15454 | 15140 | -1 | - | 315 | Frataxin homolog CyaY, facilitates iron supply for heme A synthesis or Fe-S cluster assembly |
| CDS | contig00002 | 15749 | 16999 | 2 | + | 1251 | Diaminopimelate decarboxylase (EC 4.1.1.20) |
| CDS | contig00002 | 17062 | 17892 | 1 | + | 831 | Diaminopimelate epimerase (EC 5.1.1.7) |
| CDS | contig00002 | 17972 | 18652 | 2 | + | 681 | Protein of unknown function DUF484 |
| CDS | contig00002 | 18678 | 19622 | 3 | + | 945 | Tyrosine recombinase XerC |
| CDS | contig00002 | 19718 | 20428 | 2 | + | 711 | Putative FMN hydrolase (EC 3.1.3.-); 5-Amino-6-(5&#39;-phosphoribitylamino)uracil phosphatase |
| CDS | contig00002 | 20491 | 20904 | 1 | + | 414 | FIG00361413: hypothetical protein |
| CDS | contig00002 | 21826 | 21056 | -1 | - | 771 | Para-aminobenzoate synthase, amidotransferase component (EC 2.6.1.85) |
| CDS | contig00002 | 22999 | 22190 | -1 | - | 810 | Predicted hydrolase |
| CDS | contig00002 | 24777 | 23113 | -3 | - | 1665 | hypothetical protein |
| CDS | contig00002 | 25058 | 25687 | 2 | + | 630 | Putative membrane protein |
| CDS | contig00002 | 26859 | 25684 | -3 | - | 1176 | Molybdopterin binding motif, CinA N-terminal domain / C-terminal domain of CinA type E |
| CDS | contig00002 | 28072 | 26888 | -1 | - | 1185 | HD-GYP domain |
| CDS | contig00002 | 28055 | 28186 | 2 | + | 132 | hypothetical protein |
| CDS | contig00002 | 28277 | 29224 | 2 | + | 948 | Homoserine O-succinyltransferase (EC 2.3.1.46) |
| CDS | contig00002 | 29749 | 29231 | -1 | - | 519 | Protein tyrosine phosphatase (EC 3.1.3.48) |
| CDS | contig00002 | 29899 | 30369 | 1 | + | 471 | Transcriptional regulator, AsnC family |
| CDS | contig00002 | 32389 | 30425 | -1 | - | 1965 | Phosphoglycerol transferase I (EC 2.7.8.20) |
| CDS | contig00002 | 32615 | 32926 | 2 | + | 312 | hypothetical protein |
| CDS | contig00002 | 33044 | 34660 | 2 | + | 1617 | Neopullulanase (EC 3.2.1.135) |
| CDS | contig00002 | 35246 | 34716 | -2 | - | 531 | Arginine/ornithine antiporter ArcD |
| CDS | contig00002 | 36713 | 35271 | -2 | - | 1443 | ribosomal protein S6 glutaminyl transferase related protein |
| CDS | contig00002 | 36901 | 37374 | 1 | + | 474 | GNAT family acetyltransferase VC2332 |
| CDS | contig00002 | 37371 | 37607 | 3 | + | 237 | FIG00362435: hypothetical protein |
| CDS | contig00002 | 37650 | 38396 | 3 | + | 747 | 3-hydroxypropionate dehydrogenase (EC 1.1.1.298) |
| CDS | contig00002 | 38620 | 39045 | 1 | + | 426 | hypothetical protein |
| CDS | contig00002 | 39730 | 39146 | -1 | - | 585 | putative exported protein |
| CDS | contig00002 | 39940 | 41091 | 1 | + | 1152 | NADH-dependent butanol dehydrogenase A (EC 1.1.1.-) |
| CDS | contig00002 | 41868 | 41239 | -3 | - | 630 | Glutathione S-transferase (EC 2.5.1.18) |
| CDS | contig00002 | 43245 | 41977 | -3 | - | 1269 | O-acetylhomoserine sulfhydrylase (EC 2.5.1.49) / O-succinylhomoserine sulfhydrylase (EC 2.5.1.48) |
| CDS | contig00002 | 43561 | 43385 | -1 | - | 177 | FIG00361736: hypothetical protein |
| CDS | contig00002 | 43662 | 45416 | 3 | + | 1755 | SgrR, sugar-phosphate stress, transcriptional activator of SgrS small RNA |
| CDS | contig00002 | 45452 | 45847 | 2 | + | 396 | Conserved protein |
| CDS | contig00002 | 45878 | 46591 | 2 | + | 714 | FIG00361954: hypothetical protein |
| CDS | contig00002 | 47386 | 46646 | -1 | - | 741 | Lipase chaperone |
| CDS | contig00002 | 48294 | 47383 | -3 | - | 912 | Lipase precursor (EC 3.1.1.3) |
| CDS | contig00002 | 48561 | 49025 | 3 | + | 465 | FOG: TPR repeat |
| CDS | contig00002 | 49043 | 49663 | 2 | + | 621 | Manganese superoxide dismutase (EC 1.15.1.1) |
| CDS | contig00002 | 50584 | 49712 | -1 | - | 873 | SanA protein |
| CDS | contig00002 | 53533 | 50786 | -1 | - | 2748 | Microbial collagenase, secreted (EC 3.4.24.3) |
| CDS | contig00002 | 55110 | 53710 | -3 | - | 1401 | O-succinylbenzoic acid--CoA ligase (EC 6.2.1.26) |
| CDS | contig00002 | 56043 | 55114 | -3 | - | 930 | O-succinylbenzoate synthase (EC 4.2.1.113) |
| CDS | contig00002 | 56903 | 56043 | -2 | - | 861 | Naphthoate synthase (EC 4.1.3.36) |
| CDS | contig00002 | 57660 | 56896 | -3 | - | 765 | 2-succinyl-6-hydroxy-2,4-cyclohexadiene-1-carboxylate synthase (EC 4.2.99.20) |
| CDS | contig00002 | 59395 | 57650 | -1 | - | 1746 | 2-succinyl-5-enolpyruvyl-6-hydroxy-3-cyclohexene-1-carboxylic-acid synthase (EC 2.2.1.9) |
| CDS | contig00002 | 60760 | 59429 | -1 | - | 1332 | Menaquinone-specific isochorismate synthase (EC 5.4.4.2) |
| CDS | contig00002 | 60967 | 60767 | -1 | - | 201 | hypothetical protein |
| CDS | contig00002 | 60971 | 61876 | 2 | + | 906 | 1,4-dihydroxy-2-naphthoate polyprenyltransferase (EC 2.5.1.74) |
| CDS | contig00002 | 63474 | 62080 | -3 | - | 1395 | Uncharacterized iron-regulated membrane protein; Iron-uptake factor PiuB |
| CDS | contig00002 | 63760 | 64152 | 1 | + | 393 | hypothetical protein |
| CDS | contig00002 | 64452 | 64162 | -3 | - | 291 | FIG00361626: hypothetical protein |
| CDS | contig00002 | 65917 | 64538 | -1 | - | 1380 | ATP-dependent 23S rRNA helicase DbpA |
| CDS | contig00002 | 66038 | 66196 | 2 | + | 159 | hypothetical protein |
| CDS | contig00002 | 66478 | 67650 | 1 | + | 1173 | Nucleoside permease NupC |
| CDS | contig00002 | 68314 | 67757 | -1 | - | 558 | Cytochrome b (EC 1.10.2.2) |
| CDS | contig00002 | 68632 | 68363 | -1 | - | 270 | Probable signal peptide protein |
| CDS | contig00002 | 69206 | 68754 | -2 | - | 453 | FIG00361505: hypothetical protein |
| CDS | contig00002 | 69338 | 70303 | 2 | + | 966 | Magnesium and cobalt transport protein CorA |
| CDS | contig00002 | 70313 | 70831 | 2 | + | 519 | Small-conductance mechanosensitive channel |
| CDS | contig00002 | 71116 | 70868 | -1 | - | 249 | hypothetical protein |
| CDS | contig00002 | 72976 | 71186 | -1 | - | 1791 | Sodium/hydrogen exchanger family protein |
| CDS | contig00002 | 73592 | 73071 | -2 | - | 522 | Mannitol operon repressor |
| CDS | contig00002 | 74835 | 73690 | -3 | - | 1146 | Mannitol-1-phosphate 5-dehydrogenase (EC 1.1.1.17) |
| CDS | contig00002 | 76744 | 74873 | -1 | - | 1872 | PTS system, mannitol-specific IIC component (EC 2.7.1.69) / PTS system, mannitol-specific IIB component (EC 2.7.1.69) / PTS system, mannitol-specific IIA component |
| CDS | contig00002 | 79467 | 77446 | -3 | - | 2022 | Exoribonuclease II (EC 3.1.13.1) |
| CDS | contig00002 | 80453 | 79554 | -2 | - | 900 | Chromosome initiation inhibitor |
| CDS | contig00002 | 81316 | 80687 | -1 | - | 630 | Autoinducer synthesis protein LuxI |
| CDS | contig00002 | 81381 | 82163 | 3 | + | 783 | Transcriptional activator protein LuxR |
| CDS | contig00002 | 82426 | 83046 | 1 | + | 621 | Arginine exporter protein ArgO |
| CDS | contig00002 | 83162 | 85153 | 2 | + | 1992 | 2&#39;,3&#39;-cyclic-nucleotide 2&#39;-phosphodiesterase (EC 3.1.4.16) |
| CDS | contig00002 | 86191 | 85268 | -1 | - | 924 | Lipid A biosynthesis lauroyl acyltransferase (EC 2.3.1.-) |
| CDS | contig00002 | 86440 | 87867 | 1 | + | 1428 | ADP-heptose synthase (EC 2.7.-.-) / D-glycero-beta-D-manno-heptose 7-phosphate kinase |
| CDS | contig00002 | 89347 | 88022 | -1 | - | 1326 | Type I secretion outer membrane protein, TolC precursor |
| CDS | contig00002 | 89654 | 90295 | 2 | + | 642 | ADP-ribose pyrophosphatase (EC 3.6.1.13) |
| CDS | contig00002 | 90395 | 90841 | 2 | + | 447 | FIG074102: hypothetical protein |
| CDS | contig00002 | 90869 | 91687 | 2 | + | 819 | 3&#39;,5&#39;-cyclic-nucleotide phosphodiesterase (EC 3.1.4.17) |
| CDS | contig00002 | 91697 | 92284 | 2 | + | 588 | Putative esterase, FIGfam005057 |
| CDS | contig00002 | 92299 | 92910 | 1 | + | 612 | Mutator MutT protein |
| CDS | contig00002 | 92947 | 93351 | 1 | + | 405 | Gfa-like protein |
| CDS | contig00002 | 93543 | 93764 | 3 | + | 222 | FIG00638170: hypothetical protein |
| CDS | contig00002 | 93757 | 94623 | 1 | + | 867 | Putative membrane protein |
| CDS | contig00002 | 94625 | 96628 | 2 | + | 2004 | FIG01045166: hypothetical protein |
| CDS | contig00002 | 96742 | 98652 | 1 | + | 1911 | Topoisomerase IV subunit B (EC 5.99.1.-) |
| CDS | contig00002 | 98903 | 101197 | 2 | + | 2295 | Topoisomerase IV subunit A (EC 5.99.1.-) |
| CDS | contig00002 | 102639 | 101260 | -3 | - | 1380 | hypothetical protein |
| CDS | contig00002 | 103761 | 102970 | -3 | - | 792 | Predicted metal-dependent membrane protease |
| CDS | contig00002 | 104245 | 103871 | -1 | - | 375 | FIG00361944: hypothetical protein |
| CDS | contig00002 | 104675 | 104394 | -2 | - | 282 | FIG00361696: hypothetical protein |
| CDS | contig00002 | 104892 | 105215 | 3 | + | 324 | FIG00362179: hypothetical protein |
| CDS | contig00002 | 106203 | 105301 | -3 | - | 903 | FIG01057968: hypothetical protein |
| CDS | contig00002 | 106982 | 106215 | -2 | - | 768 | FIG01058386: hypothetical protein |
| CDS | contig00002 | 107540 | 106995 | -2 | - | 546 | FIG01056913: hypothetical protein |
| CDS | contig00002 | 107848 | 107564 | -1 | - | 285 | Chaperone protein DnaJ |
| CDS | contig00002 | 108116 | 108415 | 2 | + | 300 | hypothetical protein |
| CDS | contig00002 | 109127 | 108603 | -2 | - | 525 | Membrane protein, suppressor for copper-sensitivity ScsD |
| CDS | contig00002 | 109871 | 109131 | -2 | - | 741 | Secreted protein, suppressor for copper-sensitivity ScsC |
| CDS | contig00002 | 111924 | 109882 | -3 | - | 2043 | Membrane protein, suppressor for copper-sensitivity ScsB |
| CDS | contig00002 | 112375 | 111980 | -1 | - | 396 | Suppression of copper sensitivity: putative copper binding protein ScsA |
| CDS | contig00002 | 112524 | 113252 | 3 | + | 729 | 1-acyl-sn-glycerol-3-phosphate acyltransferase (EC 2.3.1.51) |
| CDS | contig00002 | 113459 | 114121 | 2 | + | 663 | Phage shock protein A |
| CDS | contig00002 | 114123 | 114329 | 3 | + | 207 | Phage shock domain protein |
| CDS | contig00002 | 114377 | 114595 | 2 | + | 219 | FIG00362046: hypothetical protein |
| CDS | contig00002 | 114981 | 114646 | -3 | - | 336 | FIG014461: putative endonuclease containing a URI domain |
| CDS | contig00002 | 115871 | 114978 | -2 | - | 894 | RarD protein |
| CDS | contig00002 | 115974 | 116564 | 3 | + | 591 | Hypothetical protein VC0266 (sugar utilization related?) |
| CDS | contig00002 | 117418 | 116561 | -1 | - | 858 | FIG00361388: hypothetical protein |
| CDS | contig00002 | 117510 | 118427 | 3 | + | 918 | Glycine cleavage system transcriptional activator |
| CDS | contig00002 | 119311 | 118532 | -1 | - | 780 | Zinc ABC transporter, inner membrane permease protein ZnuB |
| CDS | contig00002 | 120085 | 119321 | -1 | - | 765 | Zinc ABC transporter, ATP-binding protein ZnuC |
| CDS | contig00002 | 120180 | 121118 | 3 | + | 939 | Zinc ABC transporter, periplasmic-binding protein ZnuA |
| CDS | contig00002 | 121195 | 121869 | 1 | + | 675 | FIG00451283: hypothetical protein |
| CDS | contig00002 | 122805 | 121864 | -3 | - | 942 | Immunogenic protein |
| CDS | contig00002 | 124103 | 122802 | -2 | - | 1302 | GGDEF and EAL domain proteins |
| CDS | contig00002 | 124374 | 125711 | 3 | + | 1338 | Cell wall endopeptidase, family M23/M37 |
| CDS | contig00002 | 126719 | 125775 | -2 | - | 945 | D-3-phosphoglycerate dehydrogenase (EC 1.1.1.95) |
| CDS | contig00002 | 126881 | 127462 | 2 | + | 582 | FIG00362383: hypothetical protein |
| CDS | contig00002 | 127689 | 129032 | 3 | + | 1344 | Kef-type K+ transport system, predicted NAD-binding component |
| CDS | contig00002 | 129270 | 129869 | 3 | + | 600 | FIG01345364: inner membrane protein Yip1 |
| CDS | contig00002 | 130316 | 129984 | -2 | - | 333 | hypothetical protein |
| CDS | contig00002 | 130706 | 132193 | 2 | + | 1488 | hypothetical protein |
| CDS | contig00002 | 133122 | 132247 | -3 | - | 876 | Putative DMT superfamily metabolite efflux protein precursor |
| CDS | contig00002 | 133218 | 133676 | 3 | + | 459 | transcriptional regulator, AsnC family |
| CDS | contig00002 | 134384 | 133764 | -2 | - | 621 | Maltose O-acetyltransferase (EC 2.3.1.79) |
| CDS | contig00002 | 136048 | 134384 | -1 | - | 1665 | Trehalose-6-phosphate hydrolase (EC 3.2.1.93) |
| CDS | contig00002 | 137567 | 136137 | -2 | - | 1431 | PTS system, trehalose-specific IIB component (EC 2.7.1.69) / PTS system, trehalose-specific IIC component (EC 2.7.1.69) |
| CDS | contig00002 | 138701 | 137754 | -2 | - | 948 | Trehalose operon transcriptional repressor |
| CDS | contig00002 | 138836 | 139786 | 2 | + | 951 | Transporter |
| CDS | contig00002 | 139898 | 140092 | 2 | + | 195 | FIG00361316: hypothetical protein |
| CDS | contig00002 | 140223 | 141281 | 3 | + | 1059 | L-asparaginase (EC 3.5.1.1) |
| CDS | contig00002 | 142348 | 141362 | -1 | - | 987 | Ca2+/Na+ antiporter |
| CDS | contig00002 | 142734 | 143321 | 3 | + | 588 | FIG00362284: hypothetical protein |
| CDS | contig00002 | 143847 | 143389 | -3 | - | 459 | Methylglyoxal synthase (EC 4.2.3.3) |
| CDS | contig00002 | 144577 | 144098 | -1 | - | 480 | Bacterioferritin |
| CDS | contig00002 | 144915 | 144709 | -3 | - | 207 | Bacterioferritin-associated ferredoxin |
| CDS | contig00002 | 145054 | 145368 | 1 | + | 315 | FIG01199974: hypothetical protein |
| CDS | contig00002 | 145365 | 147425 | 3 | + | 2061 | Predicted P-loop ATPase fused to an acetyltransferase COG1444 |
| CDS | contig00002 | 147794 | 148924 | 2 | + | 1131 | Cytochrome c oxidase polypeptide II (EC 1.9.3.1) |
| CDS | contig00002 | 148934 | 150532 | 2 | + | 1599 | Cytochrome c oxidase polypeptide I (EC 1.9.3.1) |
| CDS | contig00002 | 150542 | 151096 | 2 | + | 555 | Cytochrome oxidase biogenesis protein Cox11-CtaG, copper delivery to Cox1 |
| CDS | contig00002 | 151099 | 151980 | 1 | + | 882 | Cytochrome c oxidase polypeptide III (EC 1.9.3.1) |
| CDS | contig00002 | 152195 | 151989 | -2 | - | 207 | hypothetical protein |
| CDS | contig00002 | 152277 | 152939 | 3 | + | 663 | Cytochrome oxidase biogenesis protein Surf1, facilitates heme A insertion |
| CDS | contig00002 | 152936 | 153418 | 2 | + | 483 | hypothetical protein |
| CDS | contig00002 | 153478 | 154488 | 1 | + | 1011 | Heme A synthase, cytochrome oxidase biogenesis protein Cox15-CtaA |
| CDS | contig00002 | 154485 | 155390 | 3 | + | 906 | Cytochrome oxidase biogenesis protein Sco1/SenC/PrrC, putative copper metallochaperone |
| CDS | contig00002 | 156947 | 155517 | -2 | - | 1431 | 4-aminobutyraldehyde dehydrogenase (EC 1.2.1.19) |
| CDS | contig00002 | 157759 | 156977 | -1 | - | 783 | Spermidine Putrescine ABC transporter permease component potC (TC_3.A.1.11.1) |
| CDS | contig00002 | 158738 | 157800 | -2 | - | 939 | Spermidine Putrescine ABC transporter permease component PotB (TC 3.A.1.11.1) |
| CDS | contig00002 | 159777 | 158764 | -3 | - | 1014 | Putrescine transport ATP-binding protein PotA (TC 3.A.1.11.1) |
| CDS | contig00002 | 160988 | 159843 | -2 | - | 1146 | ABC transporter, periplasmic spermidine putrescine-binding protein PotD (TC 3.A.1.11.1) |
| CDS | contig00002 | 162108 | 161185 | -3 | - | 924 | Transcriptional regulators, LysR family |
| CDS | contig00002 | 163624 | 162215 | -1 | - | 1410 | Na+/H+ antiporter NhaC |
| CDS | contig00002 | 163580 | 163768 | 2 | + | 189 | hypothetical protein |
| CDS | contig00002 | 163924 | 164133 | 1 | + | 210 | hypothetical protein |
| CDS | contig00002 | 164402 | 164130 | -2 | - | 273 | hypothetical protein |
| CDS | contig00002 | 166541 | 165135 | -2 | - | 1407 | Glycogen synthase, ADP-glucose transglucosylase (EC 2.4.1.21) |
| CDS | contig00002 | 167802 | 166585 | -3 | - | 1218 | Glucose-1-phosphate adenylyltransferase (EC 2.7.7.27) |
| CDS | contig00002 | 168981 | 167953 | -3 | - | 1029 | Ribosomal RNA small subunit methyltransferase C (EC 2.1.1.52) |
| CDS | contig00002 | 168934 | 169137 | 1 | + | 204 | hypothetical protein |
| CDS | contig00002 | 169260 | 170132 | 3 | + | 873 | 4-hydroxy-tetrahydrodipicolinate synthase (EC 4.3.3.7) |
| CDS | contig00002 | 170228 | 171874 | 2 | + | 1647 | GGDEF family protein |
| CDS | contig00002 | 172023 | 172613 | 3 | + | 591 | Putative phosphatase YqaB |
| CDS | contig00002 | 173109 | 172750 | -3 | - | 360 | RNA-binding protein Hfq |
| CDS | contig00002 | 173417 | 173298 | -2 | - | 120 | hypothetical protein |
| CDS | contig00002 | 173382 | 174557 | 3 | + | 1176 | Paraquat-inducible protein A |
| CDS | contig00002 | 174550 | 176196 | 1 | + | 1647 | Paraquat-inducible protein B |
| CDS | contig00002 | 176196 | 176765 | 3 | + | 570 | probable lipoprotein protein YPO1422 |
| CDS | contig00002 | 177852 | 176821 | -3 | - | 1032 | Outer membrane protein A precursor |
| CDS | contig00002 | 179256 | 178132 | -3 | - | 1125 | Phospholipase/lecithinase/hemolysin |
| CDS | contig00002 | 179374 | 179754 | 1 | + | 381 | Uncharacterized conserved protein |
| CDS | contig00002 | 180915 | 179812 | -3 | - | 1104 | Membrane-bound lytic murein transglycosylase C precursor (EC 3.2.1.-) |
| CDS | contig00002 | 181307 | 181035 | -2 | - | 273 | FIG001341: Probable Fe(2+)-trafficking protein YggX |
| CDS | contig00002 | 182389 | 181304 | -1 | - | 1086 | A/G-specific adenine glycosylase (EC 3.2.2.-) |
| CDS | contig00002 | 183240 | 182557 | -3 | - | 684 | General secretion pathway protein B |
| CDS | contig00002 | 184883 | 183240 | -2 | - | 1644 | General secretion pathway protein A |
| CDS | contig00002 | 185113 | 186351 | 1 | + | 1239 | tRNA nucleotidyltransferase (EC 2.7.7.21) (EC 2.7.7.25) |
| CDS | contig00002 | 187719 | 186454 | -3 | - | 1266 | Probable low-affinity inorganic phosphate transporter |
| CDS | contig00002 | 188425 | 187745 | -1 | - | 681 | Phosphate transport regulator (distant homolog of PhoU) |
| CDS | contig00002 | 188520 | 189548 | 3 | + | 1029 | Adenylate cyclase (EC 4.6.1.1) |
| CDS | contig00002 | 190098 | 189628 | -3 | - | 471 | Outer membrane lipoprotein PCP |
| CDS | contig00002 | 190682 | 190230 | -2 | - | 453 | N-acetylglutamate synthase (EC 2.3.1.1) |
| CDS | contig00002 | 192622 | 190820 | -1 | - | 1803 | Xaa-Pro aminopeptidase (EC 3.4.11.9) |
| CDS | contig00002 | 193291 | 192656 | -1 | - | 636 | Predicted regulator PutR for proline utilization, GntR family |
| CDS | contig00002 | 195120 | 193495 | -3 | - | 1626 | Phosphoenolpyruvate carboxykinase [ATP] (EC 4.1.1.49) |
| CDS | contig00002 | 196157 | 195270 | -2 | - | 888 | 33 kDa chaperonin (Heat shock protein 33) (HSP33) |
| CDS | contig00002 | 196657 | 196259 | -1 | - | 399 | Ribosome-associated heat shock protein implicated in the recycling of the 50S subunit (S4 paralog) |
| CDS | contig00002 | 196874 | 197722 | 2 | + | 849 | General secretion pathway protein C |
| CDS | contig00002 | 197795 | 199798 | 2 | + | 2004 | General secretion pathway protein D |
| CDS | contig00002 | 199798 | 201303 | 1 | + | 1506 | General secretion pathway protein E |
| CDS | contig00002 | 201305 | 202525 | 2 | + | 1221 | General secretion pathway protein F |
| CDS | contig00002 | 202715 | 203146 | 2 | + | 432 | General secretion pathway protein G |
| CDS | contig00002 | 203194 | 203757 | 1 | + | 564 | General secretion pathway protein H |
| CDS | contig00002 | 203823 | 204176 | 3 | + | 354 | General secretion pathway protein I |
| CDS | contig00002 | 204221 | 204862 | 2 | + | 642 | General secretion pathway protein J |
| CDS | contig00002 | 204928 | 205062 | 1 | + | 135 | hypothetical protein |
| CDS | contig00002 | 205109 | 206077 | 2 | + | 969 | General secretion pathway protein K |
| CDS | contig00002 | 206189 | 207373 | 2 | + | 1185 | General secretion pathway protein L |
| CDS | contig00002 | 207370 | 207861 | 1 | + | 492 | General secretion pathway protein M |
| CDS | contig00002 | 207873 | 208616 | 3 | + | 744 | General secretion pathway protein N |
| CDS | contig00002 | 209474 | 208665 | -2 | - | 810 | transcriptional regulator, LuxR family |
| CDS | contig00002 | 209634 | 209491 | -3 | - | 144 | hypothetical protein |
| CDS | contig00002 | 209587 | 210885 | 1 | + | 1299 | Tyrosyl-tRNA synthetase (EC 6.1.1.1) |
| CDS | contig00002 | 211785 | 210880 | -3 | - | 906 | Transcriptional regulator, LysR family |
| CDS | contig00002 | 211902 | 213008 | 3 | + | 1107 | NADH:flavin oxidoreductase/NADH oxidase |
| CDS | contig00002 | 213756 | 213088 | -3 | - | 669 | FIG001957: putative hydrolase |
| CDS | contig00002 | 213904 | 214512 | 1 | + | 609 | ADP compounds hydrolase NudE (EC 3.6.1.-) |
| CDS | contig00002 | 214557 | 215369 | 3 | + | 813 | 3&#39;(2&#39;),5&#39;-bisphosphate nucleotidase (EC 3.1.3.7) |
| CDS | contig00002 | 215781 | 217049 | 3 | + | 1269 | Serine transporter |
| CDS | contig00002 | 217470 | 217132 | -3 | - | 339 | Protein secretion chaperonin CsaA |
| CDS | contig00002 | 218241 | 217555 | -3 | - | 687 | Transcriptional regulator, AraC family |
| CDS | contig00002 | 218672 | 218349 | -2 | - | 324 | Methionine repressor MetJ |
| CDS | contig00002 | 218896 | 220074 | 1 | + | 1179 | Cystathionine gamma-synthase (EC 2.5.1.48) |
| CDS | contig00002 | 220074 | 222527 | 3 | + | 2454 | Aspartokinase (EC 2.7.2.4) / Homoserine dehydrogenase (EC 1.1.1.3) |
| CDS | contig00002 | 223284 | 222595 | -3 | - | 690 | CidA-associated membrane protein CidB |
| CDS | contig00002 | 223678 | 223277 | -1 | - | 402 | Holin-like protein CidA |
| CDS | contig00002 | 223807 | 224682 | 1 | + | 876 | LysR family regulatory protein CidR |
| CDS | contig00002 | 225474 | 224698 | -3 | - | 777 | ABC-type amino acid transport/signal transduction system |
| CDS | contig00002 | 226345 | 225512 | -1 | - | 834 | hypothetical protein |
| CDS | contig00002 | 227926 | 226499 | -1 | - | 1428 | Transcriptional regulator, GntR family domain / Aspartate aminotransferase (EC 2.6.1.1) |
| CDS | contig00002 | 228179 | 228646 | 2 | + | 468 | Predicted redox protein |
| CDS | contig00002 | 228665 | 229102 | 2 | + | 438 | Rhodanese-related sulfurtransferase |
| CDS | contig00002 | 229086 | 229859 | 3 | + | 774 | Protein export cytoplasm protein SecA ATPase RNA helicase (TC 3.A.5.1.1) |
| CDS | contig00002 | 230458 | 229988 | -1 | - | 471 | Cys-tRNA(Pro) deacylase YbaK |
| CDS | contig00002 | 230640 | 232121 | 3 | + | 1482 | FIG00904536: hypothetical protein |
| CDS | contig00002 | 234921 | 232288 | -3 | - | 2634 | Phosphoenolpyruvate carboxylase (EC 4.1.1.31) |
| CDS | contig00002 | 236280 | 235135 | -3 | - | 1146 | Acetylornithine deacetylase (EC 3.5.1.16) |
| CDS | contig00002 | 236542 | 237549 | 1 | + | 1008 | N-acetyl-gamma-glutamyl-phosphate reductase (EC 1.2.1.38) |
| CDS | contig00002 | 237573 | 238352 | 3 | + | 780 | Acetylglutamate kinase (EC 2.7.2.8) |
| CDS | contig00002 | 238378 | 239295 | 1 | + | 918 | Ornithine carbamoyltransferase (EC 2.1.3.3) |
| CDS | contig00002 | 239352 | 240587 | 3 | + | 1236 | Argininosuccinate synthase (EC 6.3.4.5) |
| CDS | contig00002 | 240790 | 242175 | 1 | + | 1386 | Argininosuccinate lyase (EC 4.3.2.1) |
| CDS | contig00002 | 243175 | 242282 | -1 | - | 894 | Transcriptional regulator |
| CDS | contig00002 | 243272 | 243598 | 2 | + | 327 | 4-carboxymuconolactone decarboxylase (EC 4.1.1.44) |
| CDS | contig00002 | 243589 | 243981 | 1 | + | 393 | probable tautomerase |
| CDS | contig00002 | 243978 | 244502 | 3 | + | 525 | hypothetical protein |
| CDS | contig00002 | 244738 | 245373 | 1 | + | 636 | Transcriptional regulator |
| CDS | contig00002 | 245970 | 245446 | -3 | - | 525 | hypothetical protein |
| CDS | contig00002 | 246320 | 246108 | -2 | - | 213 | FIG00361940: hypothetical protein |
| CDS | contig00002 | 246753 | 246415 | -3 | - | 339 | membrane protein |
| CDS | contig00002 | 247340 | 246750 | -2 | - | 591 | Maltose O-acetyltransferase (EC 2.3.1.79) |
| CDS | contig00002 | 248720 | 247467 | -2 | - | 1254 | FIG00361364: hypothetical protein |
| CDS | contig00002 | 249777 | 248941 | -3 | - | 837 | ABC-type amino acid transport/signal transduction systems, periplasmic component/domain |
| CDS | contig00002 | 250535 | 249774 | -2 | - | 762 | Amino-acid ABC transporter ATP-binding protein |
| CDS | contig00002 | 251194 | 250532 | -1 | - | 663 | ABC-type amino acid transport system, permease component |
| CDS | contig00002 | 251288 | 251175 | -2 | - | 114 | hypothetical protein |
| CDS | contig00002 | 251934 | 251269 | -3 | - | 666 | ABC transporter membrane-spanning permease - glutamine transport |
| CDS | contig00002 | 253142 | 251931 | -2 | - | 1212 | Threonine synthase (EC 4.2.3.1) |
| CDS | contig00002 | 253358 | 255505 | 2 | + | 2148 | Prolyl endopeptidase (EC 3.4.21.26) |
| CDS | contig00002 | 255709 | 257865 | 1 | + | 2157 | A/G-specific adenine glycosylase (EC 3.2.2.-) |
| CDS | contig00002 | 258005 | 258739 | 2 | + | 735 | FIG00553873: hypothetical protein |
| CDS | contig00002 | 261669 | 258799 | -3 | - | 2871 | Glutamate-ammonia-ligase adenylyltransferase (EC 2.7.7.42) |
| CDS | contig00002 | 262245 | 261805 | -3 | - | 441 | Histone acetyltransferase HPA2 and related acetyltransferases |
| CDS | contig00002 | 263636 | 262386 | -2 | - | 1251 | N-acetylglucosamine regulated methyl-accepting chemotaxis protein |
| CDS | contig00002 | 263737 | 264489 | 1 | + | 753 | cAMP-dependent Kef-type K+ transport system |
| CDS | contig00002 | 264642 | 265247 | 3 | + | 606 | SH3 domain protein |
| CDS | contig00002 | 265413 | 266192 | 3 | + | 780 | NADH pyrophosphatase (EC 3.6.1.22) |
| CDS | contig00002 | 266332 | 267399 | 1 | + | 1068 | Uroporphyrinogen III decarboxylase (EC 4.1.1.37) |
| CDS | contig00002 | 267469 | 267356 | -1 | - | 114 | hypothetical protein |
| CDS | contig00002 | 267551 | 268480 | 2 | + | 930 | ROK family Glucokinase with ambiguous substrate specificity |
| CDS | contig00002 | 268531 | 269541 | 1 | + | 1011 | D-erythrose-4-phosphate dehydrogenase (EC 1.2.1.72) |
| CDS | contig00002 | 269608 | 270771 | 1 | + | 1164 | Phosphoglycerate kinase (EC 2.7.2.3) |
| CDS | contig00002 | 270887 | 271966 | 2 | + | 1080 | Fructose-bisphosphate aldolase class II (EC 4.1.2.13) |
| CDS | contig00002 | 272120 | 273421 | 2 | + | 1302 | Gamma-glutamyl-putrescine oxidase (EC1.4.3.-) |
| CDS | contig00002 | 273865 | 273440 | -1 | - | 426 | FIG00361916: hypothetical protein |
| CDS | contig00002 | 275768 | 273990 | -2 | - | 1779 | Cytochrome c-type biogenesis protein DsbD, protein-disulfide reductase (EC 1.8.1.8) |
| CDS | contig00002 | 276082 | 275765 | -1 | - | 318 | Periplasmic divalent cation tolerance protein cutA |
| CDS | contig00002 | 276266 | 276607 | 2 | + | 342 | FIG00362159: hypothetical protein |
| CDS | contig00002 | 277115 | 276678 | -2 | - | 438 | hypothetical protein |
| CDS | contig00002 | 277240 | 278121 | 1 | + | 882 | Transcriptional regulator, LysR family |
| CDS | contig00002 | 279040 | 278129 | -1 | - | 912 | Folate-dependent protein for Fe/S cluster synthesis/repair in oxidative stress |
| CDS | contig00002 | 279154 | 279423 | 1 | + | 270 | YgfY COG2938 |
| CDS | contig00002 | 279488 | 279853 | 2 | + | 366 | hypothetical protein |
| CDS | contig00002 | 281579 | 279972 | -2 | - | 1608 | L-aspartate oxidase (EC 1.4.3.16) |
| CDS | contig00002 | 281761 | 282342 | 1 | + | 582 | RNA polymerase sigma factor RpoE |
| CDS | contig00002 | 282365 | 282952 | 2 | + | 588 | Sigma factor RpoE negative regulatory protein RseA |
| CDS | contig00002 | 282974 | 283954 | 2 | + | 981 | Sigma factor RpoE negative regulatory protein RseB precursor |
| CDS | contig00002 | 284036 | 284506 | 2 | + | 471 | Sigma factor RpoE regulatory protein RseC |
| CDS | contig00002 | 284666 | 286459 | 2 | + | 1794 | Translation elongation factor LepA |
| CDS | contig00002 | 286463 | 287386 | 2 | + | 924 | Signal peptidase I (EC 3.4.21.89) |
| CDS | contig00002 | 287387 | 288058 | 2 | + | 672 | Ribonuclease III (EC 3.1.26.3) |
| CDS | contig00002 | 288120 | 289019 | 3 | + | 900 | GTP-binding protein Era |
| CDS | contig00002 | 289020 | 289730 | 3 | + | 711 | DNA recombination and repair protein RecO |
| CDS | contig00002 | 289888 | 290625 | 1 | + | 738 | Pyridoxine 5&#39;-phosphate synthase (EC 2.6.99.2) |
| CDS | contig00002 | 291654 | 290767 | -3 | - | 888 | N-acetylmuramoyl-L-alanine amidase (EC 3.5.1.28) |
| CDS | contig00002 | 292172 | 291687 | -2 | - | 486 | FIG00362153: hypothetical protein |
| CDS | contig00002 | 294957 | 292237 | -3 | - | 2721 | BarA sensory histidine kinase (= VarS = GacS) |
| CDS | contig00002 | 295227 | 296549 | 3 | + | 1323 | 23S rRNA (Uracil-5-) -methyltransferase RumA (EC 2.1.1.-) |
| CDS | contig00002 | 296899 | 299109 | 1 | + | 2211 | GTP pyrophosphokinase (EC 2.7.6.5) / Guanosine-3&#39;,5&#39;-bis(diphosphate) 3&#39;-pyrophosphohydrolase (EC 3.1.7.2) |
| CDS | contig00002 | 299281 | 300078 | 1 | + | 798 | Nucleoside triphosphate pyrophosphohydrolase MazG (EC 3.6.1.8) |
| CDS | contig00002 | 300220 | 301857 | 1 | + | 1638 | CTP synthase (EC 6.3.4.2) |
| CDS | contig00002 | 301947 | 303248 | 3 | + | 1302 | Enolase (EC 4.2.1.11) |
| CDS | contig00002 | 303426 | 303743 | 3 | + | 318 | Cell division protein DivIC (FtsB), stabilizes FtsL against RasP cleavage |
| CDS | contig00002 | 303736 | 304467 | 1 | + | 732 | 2-C-methyl-D-erythritol 4-phosphate cytidylyltransferase (EC 2.7.7.60) |
| CDS | contig00002 | 304526 | 305002 | 2 | + | 477 | 2-C-methyl-D-erythritol 2,4-cyclodiphosphate synthase (EC 4.6.1.12) |
| CDS | contig00002 | 304999 | 306060 | 1 | + | 1062 | tRNA pseudouridine 13 synthase (EC 4.2.1.-) |
| CDS | contig00002 | 306041 | 306802 | 2 | + | 762 | 5-nucleotidase SurE (EC 3.1.3.5) @ Exopolyphosphatase (EC 3.6.1.11) |
| CDS | contig00002 | 306807 | 307424 | 3 | + | 618 | Protein-L-isoaspartate O-methyltransferase (EC 2.1.1.77) |
| CDS | contig00002 | 307421 | 308002 | 2 | + | 582 | FIG139438: lipoprotein B |
| CDS | contig00002 | 308048 | 309082 | 2 | + | 1035 | Lipoprotein NlpD |
| CDS | contig00002 | 309129 | 310112 | 3 | + | 984 | RNA polymerase sigma factor RpoS |
| CDS | contig00002 | 311199 | 310186 | -3 | - | 1014 | TsaD/Kae1/Qri7 protein, required for threonylcarbamoyladenosine t(6)A37 formation in tRNA |
| CDS | contig00002 | 311379 | 311594 | 3 | + | 216 | SSU ribosomal protein S21p |
| CDS | contig00002 | 311610 | 312053 | 3 | + | 444 | Transamidase GatB domain protein |
| CDS | contig00002 | 312143 | 313930 | 2 | + | 1788 | DNA primase (EC 2.7.7.-) |
| CDS | contig00002 | 314153 | 316009 | 2 | + | 1857 | RNA polymerase sigma factor RpoD |
| CDS | contig00002 | 316798 | 316328 | -1 | - | 471 | FOG: TPR repeat protein |
| CDS | contig00002 | 318853 | 316946 | -1 | - | 1908 | Alpha-amylase precursor (EC 3.2.1.1) (1,4-alpha-D-glucan glucanohydrolase) |
| CDS | contig00002 | 320338 | 319784 | -1 | - | 555 | Cytochrome b (EC 1.10.2.2) |
| CDS | contig00002 | 320693 | 320319 | -2 | - | 375 | Soluble cytochrome b562 |
| CDS | contig00002 | 322388 | 321099 | -2 | - | 1290 | Phosphoribosylamine--glycine ligase (EC 6.3.4.13) |
| CDS | contig00002 | 322350 | 322499 | 3 | + | 150 | hypothetical protein |
| CDS | contig00002 | 324085 | 322493 | -1 | - | 1593 | IMP cyclohydrolase (EC 3.5.4.10) / Phosphoribosylaminoimidazolecarboxamide formyltransferase (EC 2.1.2.3) |
| CDS | contig00002 | 324518 | 324955 | 2 | + | 438 | transcriptional regulator, MerR family |
| CDS | contig00002 | 324945 | 325481 | 3 | + | 537 | Dihydrofolate reductase (EC 1.5.1.3) |
| CDS | contig00002 | 325589 | 325807 | 2 | + | 219 | hypothetical protein |
| CDS | contig00002 | 327069 | 325924 | -3 | - | 1146 | Radical SAM family enzyme, similar to coproporphyrinogen III oxidase, oxygen-independent, clustered with nucleoside-triphosphatase RdgB |
| CDS | contig00002 | 327798 | 327202 | -3 | - | 597 | Nucleoside 5-triphosphatase RdgB (dHAPTP, dITP, XTP-specific) (EC 3.6.1.15) |
| CDS | contig00002 | 328033 | 329196 | 1 | + | 1164 | Multidrug resistance transporter, Bcr/CflA family |
| CDS | contig00002 | 329300 | 330349 | 2 | + | 1050 | Uncharacterized oxidoreductase ydgJ (EC 1.-.-.-) |
| CDS | contig00002 | 330656 | 330901 | 2 | + | 246 | Outer membrane protein assembly factor YaeT precursor |
| CDS | contig00003 | 26 | 472 | 2 | + | 447 | MSHA pilin protein MshD |
| CDS | contig00003 | 601 | 1335 | 1 | + | 735 | MSHA biogenesis protein MshO |
| CDS | contig00003 | 1325 | 1825 | 2 | + | 501 | MSHA biogenesis protein MshP |
| CDS | contig00003 | 1933 | 5325 | 1 | + | 3393 | MSHA biogenesis protein MshQ |
| CDS | contig00003 | 5542 | 6582 | 1 | + | 1041 | Rod shape-determining protein MreB |
| CDS | contig00003 | 6624 | 7532 | 3 | + | 909 | Rod shape-determining protein MreC |
| CDS | contig00003 | 7532 | 8017 | 2 | + | 486 | Rod shape-determining protein MreD |
| CDS | contig00003 | 8070 | 8657 | 3 | + | 588 | Septum formation protein Maf |
| CDS | contig00003 | 8737 | 10206 | 1 | + | 1470 | Cytoplasmic axial filament protein CafA and Ribonuclease G (EC 3.1.4.-) |
| CDS | contig00003 | 10207 | 14106 | 1 | + | 3900 | FIG005080: Possible exported protein |
| CDS | contig00003 | 14358 | 15164 | 3 | + | 807 | FIG003879: Predicted amidohydrolase |
| CDS | contig00003 | 15231 | 16670 | 3 | + | 1440 | TldD protein, part of TldE/TldD proteolytic complex |
| CDS | contig00003 | 16703 | 17566 | 2 | + | 864 | Sialic acid utilization regulator, RpiR family |
| CDS | contig00003 | 18260 | 17655 | -2 | - | 606 | hypothetical protein |
| CDS | contig00003 | 19383 | 18310 | -3 | - | 1074 | Arginine/ornithine antiporter ArcD |
| CDS | contig00003 | 20940 | 19486 | -3 | - | 1455 | PTS system, N-acetylmuramic acid-specific IIB component (EC 2.7.1.69) / PTS system, N-acetylmuramic acid-specific IIC component |
| CDS | contig00003 | 21930 | 21034 | -3 | - | 897 | N-acetylmuramic acid 6-phosphate etherase |
| CDS | contig00003 | 22400 | 22197 | -2 | - | 204 | FIG00362255: hypothetical protein |
| CDS | contig00003 | 23282 | 22560 | -2 | - | 723 | Multiple antibiotic resistance protein MarC |
| CDS | contig00003 | 24719 | 23403 | -2 | - | 1317 | L-Proline/Glycine betaine transporter ProP |
| CDS | contig00003 | 24874 | 25551 | 1 | + | 678 | conserved hypothetical protein [Pyrococcus horikoshii]; COG2102: Predicted ATPases of PP-loop superfamily; IPR002761: Domain of unknown function DUF71 |
| CDS | contig00003 | 26479 | 25625 | -1 | - | 855 | YpfJ protein, zinc metalloprotease superfamily |
| CDS | contig00003 | 26817 | 26554 | -3 | - | 264 | FIG00361559: hypothetical protein |
| CDS | contig00003 | 26958 | 27479 | 3 | + | 522 | Protein export cytoplasm protein SecA ATPase RNA helicase (TC 3.A.5.1.1) |
| CDS | contig00003 | 27757 | 27581 | -1 | - | 177 | FIG00361742: hypothetical protein |
| CDS | contig00003 | 29506 | 28229 | -1 | - | 1278 | Na+/H+ antiporter NhaP |
| CDS | contig00003 | 29711 | 33406 | 2 | + | 3696 | 5-methyltetrahydrofolate--homocysteine methyltransferase (EC 2.1.1.13) |
| CDS | contig00003 | 33474 | 34541 | 3 | + | 1068 | FIG00732001: hypothetical protein |
| CDS | contig00003 | 34746 | 35642 | 3 | + | 897 | Ribosomal protein S6 glutaminyl transferase |
| CDS | contig00003 | 37053 | 35797 | -3 | - | 1257 | two-component system sensor protein |
| CDS | contig00003 | 37718 | 37050 | -2 | - | 669 | Putative two-component response regulator |
| CDS | contig00003 | 37809 | 38237 | 3 | + | 429 | Lipoprotein, putative |
| CDS | contig00003 | 38316 | 38804 | 3 | + | 489 | hypothetical protein |
| CDS | contig00003 | 39180 | 38842 | -3 | - | 339 | hypothetical protein |
| CDS | contig00003 | 39244 | 41235 | 1 | + | 1992 | Hydroxymethylpyrimidine phosphate synthase ThiC (EC 4.1.99.17) |
| CDS | contig00003 | 41232 | 42839 | 3 | + | 1608 | Hydroxymethylpyrimidine phosphate kinase ThiD (EC 2.7.4.7) / Thiamin-phosphate pyrophosphorylase (EC 2.5.1.3) |
| CDS | contig00003 | 42829 | 43608 | 1 | + | 780 | Sulfur carrier protein adenylyltransferase ThiF |
| CDS | contig00003 | 43611 | 43811 | 3 | + | 201 | Sulfur carrier protein ThiS |
| CDS | contig00003 | 43897 | 44664 | 1 | + | 768 | Thiazole biosynthesis protein ThiG |
| CDS | contig00003 | 44661 | 45932 | 3 | + | 1272 | 2-iminoacetate synthase (ThiH) (EC 4.1.99.19) |
| CDS | contig00003 | 48463 | 47828 | -1 | - | 636 | Chloramphenicol acetyltransferase (EC 2.3.1.28) |
| CDS | contig00003 | 48687 | 49271 | 3 | + | 585 | FIG00545237: hypothetical protein |
| CDS | contig00003 | 49600 | 50490 | 1 | + | 891 | putative GGDEF family protein |
| CDS | contig00003 | 51192 | 50554 | -3 | - | 639 | exopolysaccharide synthesis protein ExoD-related protein |
| CDS | contig00003 | 51412 | 52008 | 1 | + | 597 | hypothetical protein |
| CDS | contig00003 | 52054 | 52584 | 1 | + | 531 | GCN5-related N-acetyltransferase |
| CDS | contig00003 | 52870 | 52565 | -1 | - | 306 | Protein ydhR precursor |
| CDS | contig00003 | 52992 | 53348 | 3 | + | 357 | Transcriptional regulator, HxlR family |
| CDS | contig00003 | 54357 | 53320 | -3 | - | 1038 | Fucose 4-O-acetylase |
| CDS | contig00003 | 55154 | 54534 | -2 | - | 621 | Probable transmembrane protein |
| CDS | contig00003 | 57262 | 55220 | -1 | - | 2043 | Ferrichrome-iron receptor |
| CDS | contig00003 | 57535 | 58014 | 1 | + | 480 | Acetyltransferase, GNAT family |
| CDS | contig00003 | 59155 | 58097 | -1 | - | 1059 | Outer membrane protein C precursor |
| CDS | contig00003 | 59374 | 60384 | 1 | + | 1011 | Fructose-1,6-bisphosphatase, GlpX type (EC 3.1.3.11) |
| CDS | contig00003 | 60526 | 60777 | 1 | + | 252 | Proteinase inhibitor |
| CDS | contig00003 | 62197 | 60941 | -1 | - | 1257 | UDP-N-acetylglucosamine 1-carboxyvinyltransferase (EC 2.5.1.7) |
| CDS | contig00003 | 62474 | 62217 | -2 | - | 258 | YrbA protein |
| CDS | contig00003 | 62746 | 62474 | -1 | - | 273 | Uncharacterized protein YrbB |
| CDS | contig00003 | 63375 | 62743 | -3 | - | 633 | Uncharacterized ABC transporter, auxiliary component YrbC |
| CDS | contig00003 | 63875 | 63378 | -2 | - | 498 | Uncharacterized ABC transporter, periplasmic component YrbD |
| CDS | contig00003 | 64662 | 63883 | -3 | - | 780 | Uncharacterized ABC transporter, permease component YrbE |
| CDS | contig00003 | 65450 | 64662 | -2 | - | 789 | Uncharacterized ABC transporter, ATP-binding protein YrbF |
| CDS | contig00003 | 65669 | 66664 | 2 | + | 996 | Arabinose 5-phosphate isomerase (EC 5.3.1.13) |
| CDS | contig00003 | 66664 | 67218 | 1 | + | 555 | 3-deoxy-D-manno-octulosonate 8-phosphate phosphatase (EC 3.1.3.45) |
| CDS | contig00003 | 67215 | 67775 | 3 | + | 561 | Uncharacterized protein YrbK clustered with lipopolysaccharide transporters |
| CDS | contig00003 | 67747 | 68280 | 1 | + | 534 | LptA, protein essential for LPS transport across the periplasm |
| CDS | contig00003 | 68293 | 69018 | 1 | + | 726 | Lipopolysaccharide ABC transporter, ATP-binding protein LptB |
| CDS | contig00003 | 69084 | 70523 | 3 | + | 1440 | RNA polymerase sigma-54 factor RpoN |
| CDS | contig00003 | 70544 | 70831 | 2 | + | 288 | Ribosome hibernation protein YhbH |
| CDS | contig00003 | 70834 | 71280 | 1 | + | 447 | PTS system nitrogen-specific IIA component, PtsN |
| CDS | contig00003 | 71321 | 72187 | 2 | + | 867 | Hypothetical ATP-binding protein UPF0042, contains P-loop |
| CDS | contig00003 | 72208 | 72480 | 1 | + | 273 | Phosphocarrier protein, nitrogen regulation associated |
| CDS | contig00003 | 74400 | 72496 | -3 | - | 1905 | Putative BglB-family transcriptional antiterminator |
| CDS | contig00003 | 75243 | 74503 | -3 | - | 741 | 2-dehydro-3-deoxyphosphogluconate aldolase (EC 4.1.2.14) in D-glucosaminate utilization operon |
| CDS | contig00003 | 76358 | 75243 | -2 | - | 1116 | D-Glucosaminate-6-phosphate ammonia-lyase (EC 4.3.1.-) |
| CDS | contig00003 | 77481 | 76342 | -3 | - | 1140 | Metallo-dependent hydrolases, subgroup B |
| CDS | contig00003 | 78342 | 77683 | -3 | - | 660 | Putative inner membrane protein |
| CDS | contig00003 | 79121 | 78345 | -2 | - | 777 | Putative inner membrane protein |
| CDS | contig00003 | 79443 | 79147 | -3 | - | 297 | FIG074102: hypothetical protein |
| CDS | contig00003 | 79808 | 79443 | -2 | - | 366 | FIG00628496: hypothetical protein |
| CDS | contig00003 | 80171 | 79809 | -2 | - | 363 | FIG01045571: hypothetical protein |
| CDS | contig00003 | 81579 | 80479 | -3 | - | 1101 | Outer membrane stress sensor protease DegS |
| CDS | contig00003 | 83054 | 81696 | -2 | - | 1359 | Outer membrane stress sensor protease DegQ, serine protease |
| CDS | contig00003 | 83516 | 83136 | -2 | - | 381 | Putative cytochrome d ubiquinol oxidase subunit III (EC 1.10.3.-) (Cytochrome bd-I oxidase subunit III) |
| CDS | contig00003 | 83656 | 84750 | 1 | + | 1095 | ATPase, AFG1 family |
| CDS | contig00003 | 85017 | 85445 | 3 | + | 429 | LSU ribosomal protein L13p (L13Ae) |
| CDS | contig00003 | 85460 | 85852 | 2 | + | 393 | SSU ribosomal protein S9p (S16e) |
| CDS | contig00003 | 86243 | 86743 | 2 | + | 501 | Ubiquinol-cytochrome C reductase iron-sulfur subunit (EC 1.10.2.2) |
| CDS | contig00003 | 86746 | 87963 | 1 | + | 1218 | Ubiquinol--cytochrome c reductase, cytochrome B subunit (EC 1.10.2.2) |
| CDS | contig00003 | 87960 | 88694 | 3 | + | 735 | ubiquinol cytochrome C oxidoreductase, cytochrome C1 subunit |
| CDS | contig00003 | 88782 | 89411 | 3 | + | 630 | Stringent starvation protein A |
| CDS | contig00003 | 89426 | 89839 | 2 | + | 414 | Stringent starvation protein B |
| CDS | contig00003 | 90461 | 89883 | -2 | - | 579 | 21 kDa hemolysin precursor |
| CDS | contig00003 | 91056 | 90466 | -3 | - | 591 | Phosphoheptose isomerase (EC 5.3.1.-) |
| CDS | contig00003 | 91359 | 91066 | -3 | - | 294 | Endonuclease (EC 3.1.-.-) |
| CDS | contig00003 | 93169 | 91379 | -1 | - | 1791 | LppC putative lipoprotein |
| CDS | contig00003 | 93369 | 94202 | 3 | + | 834 | rRNA small subunit methyltransferase I |
| CDS | contig00003 | 95101 | 95556 | 1 | + | 456 | Cell division protein MraZ |
| CDS | contig00003 | 95557 | 96495 | 1 | + | 939 | rRNA small subunit methyltransferase H |
| CDS | contig00003 | 96499 | 96813 | 1 | + | 315 | Cell division protein FtsL |
| CDS | contig00003 | 96810 | 98576 | 3 | + | 1767 | Cell division protein FtsI [Peptidoglycan synthetase] (EC 2.4.1.129) |
| CDS | contig00003 | 98672 | 100156 | 2 | + | 1485 | UDP-N-acetylmuramoylalanyl-D-glutamate--2,6-diaminopimelate ligase (EC 6.3.2.13) |
| CDS | contig00003 | 100153 | 101508 | 1 | + | 1356 | UDP-N-acetylmuramoylalanyl-D-glutamyl-2,6-diaminopimelate--D-alanyl-D-alanine ligase (EC 6.3.2.10) |
| CDS | contig00003 | 101502 | 102584 | 3 | + | 1083 | Phospho-N-acetylmuramoyl-pentapeptide-transferase (EC 2.7.8.13) |
| CDS | contig00003 | 102588 | 103871 | 3 | + | 1284 | UDP-N-acetylmuramoylalanine--D-glutamate ligase (EC 6.3.2.9) |
| CDS | contig00003 | 103868 | 105052 | 2 | + | 1185 | Cell division protein FtsW |
| CDS | contig00003 | 105049 | 106116 | 1 | + | 1068 | UDP-N-acetylglucosamine--N-acetylmuramyl-(pentapeptide) pyrophosphoryl-undecaprenol N-acetylglucosamine transferase (EC 2.4.1.227) |
| CDS | contig00003 | 106227 | 107681 | 3 | + | 1455 | UDP-N-acetylmuramate--alanine ligase (EC 6.3.2.8) |
| CDS | contig00003 | 107743 | 108531 | 1 | + | 789 | Cell division protein FtsQ |
| CDS | contig00003 | 108512 | 109771 | 2 | + | 1260 | Cell division protein FtsA |
| CDS | contig00003 | 109811 | 110962 | 2 | + | 1152 | Cell division protein FtsZ (EC 3.4.24.-) |
| CDS | contig00003 | 111182 | 111988 | 2 | + | 807 | UDP-3-O-[3-hydroxymyristoyl] N-acetylglucosamine deacetylase (EC 3.5.1.108) |
| CDS | contig00003 | 112129 | 113034 | 1 | + | 906 | Peptidase, M23/M37 family |
| CDS | contig00003 | 113092 | 115812 | 1 | + | 2721 | Protein export cytoplasm protein SecA ATPase RNA helicase (TC 3.A.5.1.1) |
| CDS | contig00003 | 115878 | 117062 | 3 | + | 1185 | TPR repeat |
| CDS | contig00003 | 117071 | 117475 | 2 | + | 405 | Mutator mutT protein (7,8-dihydro-8-oxoguanine-triphosphatase) (EC 3.6.1.-) |
| CDS | contig00003 | 117727 | 117533 | -1 | - | 195 | FIG003276: zinc-binding protein |
| CDS | contig00003 | 118460 | 117738 | -2 | - | 723 | FIG002842: hypothetical protein |
| CDS | contig00003 | 119112 | 118498 | -3 | - | 615 | Dephospho-CoA kinase (EC 2.7.1.24) |
| CDS | contig00003 | 119997 | 119131 | -3 | - | 867 | Leader peptidase (Prepilin peptidase) (EC 3.4.23.43) / N-methyltransferase (EC 2.1.1.-) |
| CDS | contig00003 | 121325 | 120084 | -2 | - | 1242 | Type IV fimbrial assembly protein PilC |
| CDS | contig00003 | 123159 | 121453 | -3 | - | 1707 | Type IV fimbrial assembly, ATPase PilB |
| CDS | contig00003 | 124799 | 123936 | -2 | - | 864 | Quinolinate phosphoribosyltransferase [decarboxylating] (EC 2.4.2.19) |
| CDS | contig00003 | 125273 | 124803 | -2 | - | 471 | FIG00536384: hypothetical protein |
| CDS | contig00003 | 125402 | 125974 | 2 | + | 573 | N-acetylmuramoyl-L-alanine amidase (EC 3.5.1.28) AmpD |
| CDS | contig00003 | 126251 | 127015 | 2 | + | 765 | Transcriptional repressor for pyruvate dehydrogenase complex |
| CDS | contig00003 | 127099 | 129759 | 1 | + | 2661 | Pyruvate dehydrogenase E1 component (EC 1.2.4.1) |
| CDS | contig00003 | 129880 | 131775 | 1 | + | 1896 | Dihydrolipoamide acetyltransferase component of pyruvate dehydrogenase complex (EC 2.3.1.12) |
| CDS | contig00003 | 131921 | 133351 | 2 | + | 1431 | Dihydrolipoamide dehydrogenase of pyruvate dehydrogenase complex (EC 1.8.1.4) |
| CDS | contig00003 | 133607 | 133425 | -2 | - | 183 | FIG00361776: hypothetical protein |
| CDS | contig00003 | 135398 | 133752 | -2 | - | 1647 | Methyl-accepting chemotaxis protein |
| CDS | contig00003 | 137431 | 135521 | -1 | - | 1911 | Methyl-accepting chemotaxis protein |
| CDS | contig00003 | 137705 | 140008 | 2 | + | 2304 | putative patatin-like phospholipase |
| CDS | contig00003 | 140238 | 142835 | 3 | + | 2598 | Aconitate hydratase 2 (EC 4.2.1.3) @ 2-methylisocitrate dehydratase (EC 4.2.1.99) |
| CDS | contig00003 | 143067 | 144638 | 3 | + | 1572 | Rtn protein |
| CDS | contig00003 | 145552 | 144635 | -1 | - | 918 | Transcriptional regulator, LysR family |
| CDS | contig00003 | 145629 | 146246 | 3 | + | 618 | Threonine efflux protein |
| CDS | contig00003 | 146362 | 147768 | 1 | + | 1407 | Putative metal chaperone, involved in Zn homeostasis, GTPase of COG0523 family |
| CDS | contig00003 | 147849 | 148967 | 3 | + | 1119 | Putative metal chaperone, involved in Zn homeostasis, GTPase of COG0523 family |
| CDS | contig00003 | 149029 | 150417 | 1 | + | 1389 | Drug resistance transporter, EmrB/QacA family |
| CDS | contig00003 | 150909 | 150427 | -3 | - | 483 | HigA protein (antitoxin to HigB) |
| CDS | contig00003 | 151536 | 151027 | -3 | - | 510 | 2-amino-4-hydroxy-6-hydroxymethyldihydropteridine pyrophosphokinase (EC 2.7.6.3) |
| CDS | contig00003 | 151907 | 151536 | -2 | - | 372 | Dihydroneopterin aldolase (EC 4.1.2.25) |
| CDS | contig00003 | 152100 | 152741 | 3 | + | 642 | Acyl-phosphate:glycerol-3-phosphate O-acyltransferase PlsY |
| CDS | contig00003 | 153829 | 154197 | 1 | + | 369 | Mobile element protein |
| CDS | contig00003 | 154944 | 154465 | -3 | - | 480 | Transcriptional regulator, MarR family |
| CDS | contig00003 | 155108 | 155635 | 2 | + | 528 | FIG00361875: hypothetical protein |
| CDS | contig00003 | 155645 | 157243 | 2 | + | 1599 | Glycine betaine transporter OpuD |
| CDS | contig00003 | 159851 | 157314 | -2 | - | 2538 | DNA mismatch repair protein MutS |
| CDS | contig00003 | 160555 | 160148 | -1 | - | 408 | Probable secreted protein |
| CDS | contig00003 | 160752 | 161246 | 3 | + | 495 | C-terminal domain of CinA type S; Protein Implicated in DNA repair function with RecA and MutS |
| CDS | contig00003 | 161327 | 162391 | 2 | + | 1065 | RecA protein |
| CDS | contig00003 | 162448 | 162939 | 1 | + | 492 | Regulatory protein RecX |
| CDS | contig00003 | 163219 | 165843 | 1 | + | 2625 | Alanyl-tRNA synthetase (EC 6.1.1.7) |
| CDS | contig00003 | 165860 | 167107 | 2 | + | 1248 | Aspartokinase (EC 2.7.2.4) |
| CDS | contig00003 | 167201 | 167389 | 2 | + | 189 | Carbon storage regulator |
| CDS | contig00003 | 169371 | 168586 | -3 | - | 786 | Murein-DD-endopeptidase (EC 3.4.99.-) |
| CDS | contig00003 | 170215 | 169550 | -1 | - | 666 | DedA protein |
| CDS | contig00003 | 171526 | 170234 | -1 | - | 1293 | Magnesium and cobalt efflux protein CorC |
| CDS | contig00003 | 174586 | 171734 | -1 | - | 2853 | Valyl-tRNA synthetase (EC 6.1.1.9) |
| CDS | contig00003 | 175098 | 174652 | -3 | - | 447 | DNA polymerase III chi subunit (EC 2.7.7.7) |
| CDS | contig00003 | 176788 | 175277 | -1 | - | 1512 | Cytosol aminopeptidase PepA (EC 3.4.11.1) |
| CDS | contig00003 | 176985 | 178073 | 3 | + | 1089 | FIG000988: Predicted permease |
| CDS | contig00003 | 178099 | 179169 | 1 | + | 1071 | FIG000906: Predicted Permease |
| CDS | contig00003 | 179826 | 179314 | -3 | - | 513 | FIG023103: Predicted transmembrane protein |
| CDS | contig00004 | 507 | 139 | -3 | - | 369 | hypothetical protein |
| CDS | contig00004 | 1028 | 912 | -2 | - | 117 | hypothetical protein |
| CDS | contig00004 | 1041 | 2468 | 3 | + | 1428 | Ammonium transporter |
| CDS | contig00004 | 2700 | 4052 | 3 | + | 1353 | 23S rRNA (Uracil-5-) -methyltransferase RumA (EC 2.1.1.-) |
| CDS | contig00004 | 4240 | 4749 | 1 | + | 510 | acetyltransferase, putative |
| CDS | contig00004 | 4831 | 5001 | 1 | + | 171 | hypothetical protein |
| CDS | contig00004 | 5584 | 4970 | -1 | - | 615 | MSHA pilin protein MshB |
| CDS | contig00004 | 5625 | 6413 | 3 | + | 789 | Putative deoxyribonuclease YjjV |
| CDS | contig00004 | 6627 | 7901 | 3 | + | 1275 | Nucleoside permease NupC |
| CDS | contig00004 | 8046 | 8195 | 3 | + | 150 | hypothetical protein |
| CDS | contig00004 | 8583 | 9356 | 3 | + | 774 | Deoxyribose-phosphate aldolase (EC 4.1.2.4) |
| CDS | contig00004 | 9439 | 10770 | 1 | + | 1332 | Thymidine phosphorylase (EC 2.4.2.4) |
| CDS | contig00004 | 10785 | 11993 | 3 | + | 1209 | Phosphopentomutase (EC 5.4.2.7) |
| CDS | contig00004 | 12127 | 12843 | 1 | + | 717 | Purine nucleoside phosphorylase (EC 2.4.2.1) |
| CDS | contig00004 | 15154 | 12908 | -1 | - | 2247 | Chemotaxis protein methyltransferase CheR (EC 2.1.1.80) |
| CDS | contig00004 | 15997 | 15221 | -1 | - | 777 | Smp-like protein |
| CDS | contig00004 | 16023 | 17027 | 3 | + | 1005 | Phosphoserine phosphatase (EC 3.1.3.3) |
| CDS | contig00004 | 17056 | 17961 | 1 | + | 906 | Esterase/lipase/thioesterase family protein |
| CDS | contig00004 | 20394 | 18019 | -3 | - | 2376 | FIG00921683: hypothetical protein |
| CDS | contig00004 | 20495 | 21859 | 2 | + | 1365 | DNA repair protein RadA |
| CDS | contig00004 | 22299 | 21913 | -3 | - | 387 | FIG01056990: hypothetical protein |
| CDS | contig00004 | 22884 | 22402 | -3 | - | 483 | Regulator of sigma D |
| CDS | contig00004 | 23223 | 23495 | 3 | + | 273 | DNA-binding protein HU-alpha |
| CDS | contig00004 | 23588 | 24034 | 2 | + | 447 | FIG00362312: hypothetical protein |
| CDS | contig00004 | 24267 | 24118 | -3 | - | 150 | hypothetical protein |
| CDS | contig00004 | 25256 | 24270 | -2 | - | 987 | tRNA dihydrouridine synthase A (EC 1.-.-.-) |
| CDS | contig00004 | 25387 | 25851 | 1 | + | 465 | Zinc uptake regulation protein ZUR |
| CDS | contig00004 | 25841 | 26302 | 2 | + | 462 | Chemotaxis protein CheX |
| CDS | contig00004 | 26308 | 26535 | 1 | + | 228 | hypothetical protein |
| CDS | contig00004 | 27888 | 26626 | -3 | - | 1263 | Branched-chain amino acid transport system carrier protein |
| CDS | contig00004 | 28834 | 28124 | -1 | - | 711 | tRNA (adenine37-N(6))-methyltransferase TrmN6 (EC 2.1.1.223) |
| CDS | contig00004 | 28962 | 30182 | 3 | + | 1221 | ATP-dependent RNA helicase SrmB |
| CDS | contig00004 | 31143 | 30370 | -3 | - | 774 | UPF0246 protein YaaA |
| CDS | contig00004 | 32285 | 31176 | -2 | - | 1110 | Twitching motility protein PilT |
| CDS | contig00004 | 33265 | 32309 | -1 | - | 957 | Twitching motility protein PilT |
| CDS | contig00004 | 33383 | 34084 | 2 | + | 702 | Hypothetical protein YggS, proline synthase co-transcribed bacterial homolog PROSC |
| CDS | contig00004 | 34207 | 35031 | 1 | + | 825 | Pyrroline-5-carboxylate reductase (EC 1.5.1.2) |
| CDS | contig00004 | 35055 | 35606 | 3 | + | 552 | Integral membrane protein YggT, involved in response to extracytoplasmic stress (osmotic shock) |
| CDS | contig00004 | 35606 | 35905 | 2 | + | 300 | COG1872 |
| CDS | contig00004 | 35924 | 36343 | 2 | + | 420 | FIG003551: hypothetical protein |
| CDS | contig00004 | 37249 | 36419 | -1 | - | 831 | Putative membrane protein precursor |
| CDS | contig00004 | 37701 | 37411 | -3 | - | 291 | FIG00361987: hypothetical protein |
| CDS | contig00004 | 38125 | 37751 | -1 | - | 375 | hypothetical protein |
| CDS | contig00004 | 38332 | 39447 | 1 | + | 1116 | Sugar diacid utilization regulator SdaR |
| CDS | contig00004 | 39545 | 39420 | -2 | - | 126 | hypothetical protein |
| CDS | contig00004 | 39567 | 40829 | 3 | + | 1263 | D-glycerate transporter (predicted) |
| CDS | contig00004 | 40839 | 41972 | 3 | + | 1134 | Glycerate kinase (EC 2.7.1.31) |
| CDS | contig00004 | 42234 | 42797 | 3 | + | 564 | NnrU family protein in cluster with Mesaconyl-CoA hydratase |
| CDS | contig00004 | 42857 | 43393 | 2 | + | 537 | Glutathione peroxidase family protein |
| CDS | contig00004 | 43476 | 44411 | 3 | + | 936 | Transcriptional regulator, LysR family |
| CDS | contig00004 | 44594 | 45790 | 2 | + | 1197 | Nucleoside permease NupC |
| CDS | contig00004 | 46873 | 46379 | -1 | - | 495 | Methylated-DNA--protein-cysteine methyltransferase (EC 2.1.1.63) |
| CDS | contig00004 | 47316 | 46900 | -3 | - | 417 | Hypothetical protein YaeJ with similarity to translation release factor |
| CDS | contig00004 | 47518 | 47856 | 1 | + | 339 | Nitrogen regulatory protein P-II |
| CDS | contig00004 | 48082 | 49092 | 1 | + | 1011 | Ferric iron ABC transporter, iron-binding protein |
| CDS | contig00004 | 49132 | 50757 | 1 | + | 1626 | Ferric iron ABC transporter, permease protein |
| CDS | contig00004 | 50798 | 51832 | 2 | + | 1035 | Ferric iron ABC transporter, ATP-binding protein |
| CDS | contig00004 | 51922 | 52827 | 1 | + | 906 | Predicted dye-decolorizing peroxidase (DyP), YfeX-like subgroup |
| CDS | contig00004 | 53377 | 52907 | -1 | - | 471 | Arginine pathway regulatory protein ArgR, repressor of arg regulon |
| CDS | contig00004 | 53578 | 54318 | 1 | + | 741 | Arginine ABC transporter, ATP-binding protein ArtP |
| CDS | contig00004 | 54343 | 55080 | 1 | + | 738 | Arginine ABC transporter, periplasmic arginine-binding protein ArtI |
| CDS | contig00004 | 55114 | 55734 | 1 | + | 621 | Arginine ABC transporter, permease protein ArtQ |
| CDS | contig00004 | 55737 | 56399 | 3 | + | 663 | Arginine ABC transporter, permease protein ArtM |
| CDS | contig00004 | 56642 | 57577 | 2 | + | 936 | Malate dehydrogenase (EC 1.1.1.37) |
| CDS | contig00004 | 58094 | 58633 | 2 | + | 540 | Sodium-type flagellar protein MotX |
| CDS | contig00004 | 59909 | 58716 | -2 | - | 1194 | Flavohemoprotein (Hemoglobin-like protein) (Flavohemoglobin) (Nitric oxide dioxygenase) (EC 1.14.12.17) |
| CDS | contig00004 | 60021 | 60488 | 3 | + | 468 | Nitrite-sensitive transcriptional repressor NsrR |
| CDS | contig00004 | 61775 | 60519 | -2 | - | 1257 | Hemolysins and related proteins containing CBS domains |
| CDS | contig00004 | 62630 | 61839 | -2 | - | 792 | Glutamate synthase [NADPH] small chain (EC 1.4.1.13) |
| CDS | contig00004 | 62916 | 64295 | 3 | + | 1380 | Signal recognition particle, subunit Ffh SRP54 (TC 3.A.5.1.1) |
| CDS | contig00004 | 64475 | 64723 | 2 | + | 249 | SSU ribosomal protein S16p |
| CDS | contig00004 | 64748 | 65269 | 2 | + | 522 | 16S rRNA processing protein RimM |
| CDS | contig00004 | 65304 | 66053 | 3 | + | 750 | tRNA (Guanine37-N1) -methyltransferase (EC 2.1.1.31) |
| CDS | contig00004 | 66086 | 66433 | 2 | + | 348 | LSU ribosomal protein L19p |
| CDS | contig00004 | 66865 | 66536 | -1 | - | 330 | 1,4-alpha-glucan branching enzyme (EC 2.4.1.18) |
| CDS | contig00004 | 67623 | 66949 | -3 | - | 675 | DNA mismatch repair endonuclease MutH |
| CDS | contig00004 | 68355 | 68882 | 3 | + | 528 | Adenosine (5&#39;)-pentaphospho-(5&#39;&#39;)-adenosine pyrophosphohydrolase (EC 3.6.1.-) |
| CDS | contig00004 | 68896 | 71175 | 1 | + | 2280 | FIG001592: Phosphocarrier protein kinase/phosphorylase, nitrogen regulation associated |
| CDS | contig00004 | 71329 | 72066 | 1 | + | 738 | Prolipoprotein diacylglyceryl transferase (EC 2.4.99.-) |
| CDS | contig00004 | 72066 | 72860 | 3 | + | 795 | Thymidylate synthase (EC 2.1.1.45) |
| CDS | contig00004 | 73139 | 74329 | 2 | + | 1191 | Na+/H+ antiporter NhaA type |
| CDS | contig00004 | 74350 | 74730 | 1 | + | 381 | FIG00361984: hypothetical protein |
| CDS | contig00004 | 74717 | 75649 | 2 | + | 933 | Transcriptional activator NhaR |
| CDS | contig00004 | 75698 | 76000 | 2 | + | 303 | Transcriptional activator HlyU |
| CDS | contig00004 | 76234 | 76049 | -1 | - | 186 | SSU ribosomal protein S20p |
| CDS | contig00004 | 76470 | 76231 | -3 | - | 240 | hypothetical protein |
| CDS | contig00004 | 76516 | 78066 | 1 | + | 1551 | Proposed peptidoglycan lipid II flippase MurJ |
| CDS | contig00004 | 78154 | 79251 | 1 | + | 1098 | Riboflavin kinase (EC 2.7.1.26) / FMN adenylyltransferase (EC 2.7.7.2) |
| CDS | contig00004 | 79251 | 82112 | 3 | + | 2862 | Isoleucyl-tRNA synthetase (EC 6.1.1.5) |
| CDS | contig00004 | 82112 | 82615 | 2 | + | 504 | Lipoprotein signal peptidase (EC 3.4.23.36) |
| CDS | contig00004 | 82612 | 83064 | 1 | + | 453 | FKBP-type peptidyl-prolyl cis-trans isomerase SlpA (EC 5.2.1.8) |
| CDS | contig00004 | 83064 | 83999 | 3 | + | 936 | 4-hydroxy-3-methylbut-2-enyl diphosphate reductase (EC 1.17.1.2) |
| CDS | contig00004 | 84110 | 84514 | 2 | + | 405 | Type IV pilus biogenesis protein PilE |
| CDS | contig00004 | 86523 | 90185 | 3 | + | 3663 | Type IV fimbrial biogenesis protein PilY1 |
| CDS | contig00004 | 90182 | 90463 | 2 | + | 282 | hypothetical protein |
| CDS | contig00004 | 91782 | 93404 | 3 | + | 1623 | NAD synthetase (EC 6.3.1.5) / Glutamine amidotransferase chain of NAD synthetase |
| CDS | contig00004 | 93414 | 93752 | 3 | + | 339 | Nitrogen regulatory protein P-II |
| CDS | contig00004 | 93754 | 94401 | 1 | + | 648 | putative lipoprotein |
| CDS | contig00004 | 94420 | 94929 | 1 | + | 510 | S-ribosylhomocysteine lyase (EC 4.4.1.21) / Autoinducer-2 production protein LuxS |
| CDS | contig00004 | 96013 | 94991 | -1 | - | 1023 | GGDEF family protein |
| CDS | contig00004 | 96147 | 98540 | 3 | + | 2394 | 3&#39;-to-5&#39; exoribonuclease RNase R |
| CDS | contig00004 | 98537 | 99286 | 2 | + | 750 | 23S rRNA (guanosine-2&#39;-O-) -methyltransferase rlmB (EC 2.1.1.-) |
| CDS | contig00004 | 99294 | 99863 | 3 | + | 570 | cAMP-binding proteins - catabolite gene activator and regulatory subunit of cAMP-dependent protein kinases |
| CDS | contig00004 | 100015 | 100470 | 1 | + | 456 | ElaA protein |
| CDS | contig00004 | 101506 | 100658 | -1 | - | 849 | Predicted metal-dependent hydrolase |
| CDS | contig00004 | 102830 | 101607 | -2 | - | 1224 | Manganese transport protein MntH |
| CDS | contig00004 | 103067 | 103453 | 2 | + | 387 | SSU ribosomal protein S6p |
| CDS | contig00004 | 103486 | 103716 | 1 | + | 231 | SSU ribosomal protein S18p @ SSU ribosomal protein S18p, zinc-independent |
| CDS | contig00004 | 103769 | 104215 | 2 | + | 447 | LSU ribosomal protein L9p |
| CDS | contig00004 | 104317 | 105060 | 1 | + | 744 | Ferredoxin--NADP(+) reductase (EC 1.18.1.2) |
| CDS | contig00004 | 105459 | 105115 | -3 | - | 345 | hypothetical protein |
| CDS | contig00004 | 105742 | 105485 | -1 | - | 258 | hypothetical protein |
| CDS | contig00004 | 106034 | 107005 | 2 | + | 972 | FIG00362222: hypothetical protein |
| CDS | contig00004 | 107414 | 107013 | -2 | - | 402 | Fosfomycin resistance protein FosA |
| CDS | contig00004 | 107602 | 107444 | -1 | - | 159 | FIG00362022: hypothetical protein |
| CDS | contig00004 | 107668 | 108642 | 1 | + | 975 | NADPH:quinone reductase |
| CDS | contig00004 | 108639 | 109076 | 3 | + | 438 | Putative membrane protein |
| CDS | contig00004 | 109102 | 111915 | 1 | + | 2814 | Protease, insulinase family/protease, insulinase family |
| CDS | contig00004 | 111974 | 113572 | 2 | + | 1599 | Glutamate--cysteine ligase (EC 6.3.2.2) |
| CDS | contig00004 | 115010 | 113691 | -2 | - | 1320 | C4-dicarboxylate transporter DcuA |
| CDS | contig00004 | 116583 | 115141 | -3 | - | 1443 | Aspartate ammonia-lyase (EC 4.3.1.1) |
| CDS | contig00004 | 117033 | 117512 | 3 | + | 480 | FxsA protein |
| CDS | contig00004 | 118869 | 117535 | -3 | - | 1335 | Multi antimicrobial extrusion protein (Na(+)/drug antiporter), MATE family of MDR efflux pumps |
| CDS | contig00004 | 119112 | 119405 | 3 | + | 294 | Heat shock protein 60 family co-chaperone GroES |
| CDS | contig00004 | 119449 | 121083 | 1 | + | 1635 | Heat shock protein 60 family chaperone GroEL |
| CDS | contig00004 | 121229 | 121675 | 2 | + | 447 | FIG00361250: hypothetical protein |
| CDS | contig00004 | 121758 | 123632 | 3 | + | 1875 | diguanylate cyclase/phosphodiesterase (GGDEF & EAL domains) with PAS/PAC sensor(s) |
| CDS | contig00004 | 123691 | 123879 | 1 | + | 189 | FIG00361320: hypothetical protein |
| CDS | contig00004 | 124600 | 123944 | -1 | - | 657 | Ribosomal large subunit pseudouridine synthase A (EC 4.2.1.70) |
| CDS | contig00004 | 127581 | 124714 | -3 | - | 2868 | RNA polymerase associated protein RapA (EC 3.6.1.-) |
| CDS | contig00005 | 1935 | 1138 | -3 | - | 798 | FIG008480: hypothetical protein |
| CDS | contig00005 | 3474 | 2005 | -3 | - | 1470 | Succinylglutamic semialdehyde dehydrogenase (EC 1.2.1.71) |
| CDS | contig00005 | 4561 | 3542 | -1 | - | 1020 | Arginine N-succinyltransferase (EC 2.3.1.109) |
| CDS | contig00005 | 5854 | 4637 | -1 | - | 1218 | Acetylornithine aminotransferase (EC 2.6.1.11) / N-succinyl-L,L-diaminopimelate aminotransferase (EC 2.6.1.17) / Succinylornithine transaminase (EC 2.6.1.81) |
| CDS | contig00005 | 6715 | 6134 | -1 | - | 582 | Para-aminobenzoate synthase, amidotransferase component (EC 2.6.1.85) |
| CDS | contig00005 | 7252 | 7037 | -1 | - | 216 | FIG00361834: hypothetical protein |
| CDS | contig00005 | 8376 | 7372 | -3 | - | 1005 | Tryptophanyl-tRNA synthetase (EC 6.1.1.2) |
| CDS | contig00005 | 9081 | 8416 | -3 | - | 666 | Phosphoglycolate phosphatase (EC 3.1.3.18) |
| CDS | contig00005 | 9742 | 9068 | -1 | - | 675 | Ribulose-phosphate 3-epimerase (EC 5.1.3.1) |
| CDS | contig00005 | 10805 | 9933 | -2 | - | 873 | Methyl-directed repair DNA adenine methylase (EC 2.1.1.72) |
| CDS | contig00005 | 12432 | 10876 | -3 | - | 1557 | DamX, an inner membrane protein involved in bile resistance |
| CDS | contig00005 | 13504 | 12422 | -1 | - | 1083 | 3-dehydroquinate synthase (EC 4.2.3.4) |
| CDS | contig00005 | 14040 | 13522 | -3 | - | 519 | Shikimate kinase I (EC 2.7.1.71) |
| CDS | contig00005 | 16457 | 14232 | -2 | - | 2226 | Type IV pilus biogenesis protein PilQ |
| CDS | contig00005 | 16938 | 16474 | -3 | - | 465 | Type IV pilus biogenesis protein PilP |
| CDS | contig00005 | 17600 | 17004 | -2 | - | 597 | Type IV pilus biogenesis protein PilO |
| CDS | contig00005 | 18166 | 17597 | -1 | - | 570 | Type IV pilus biogenesis protein PilN |
| CDS | contig00005 | 19179 | 18154 | -3 | - | 1026 | Type IV pilus biogenesis protein PilM |
| CDS | contig00005 | 19514 | 22003 | 2 | + | 2490 | Multimodular transpeptidase-transglycosylase (EC 2.4.1.129) (EC 3.4.-.-) |
| CDS | contig00005 | 22970 | 22074 | -2 | - | 897 | Hydrogen peroxide-inducible genes activator |
| CDS | contig00005 | 23744 | 23100 | -2 | - | 645 | Thioredoxin-like protein clustered with PA0057 |
| CDS | contig00005 | 24676 | 23819 | -1 | - | 858 | Metallo-beta-lactamase superfamily protein PA0057 |
| CDS | contig00005 | 24815 | 25711 | 2 | + | 897 | LysR-family transcriptional regulator clustered with PA0057 |
| CDS | contig00005 | 26558 | 25773 | -2 | - | 786 | Possible transmembrane protein |
| CDS | contig00005 | 26916 | 26596 | -3 | - | 321 | hypothetical protein |
| CDS | contig00005 | 27054 | 27881 | 3 | + | 828 | Membrane proteins related to metalloendopeptidases |
| CDS | contig00005 | 28014 | 27892 | -3 | - | 123 | hypothetical protein |
| CDS | contig00005 | 29303 | 27969 | -2 | - | 1335 | Multi antimicrobial extrusion protein (Na(+)/drug antiporter), MATE family of MDR efflux pumps |
| CDS | contig00005 | 29457 | 32732 | 3 | + | 3276 | Potassium efflux system KefA protein / Small-conductance mechanosensitive channel |
| CDS | contig00005 | 32762 | 33439 | 2 | + | 678 | Glycerophosphoryl diester phosphodiesterase (EC 3.1.4.46) |
| CDS | contig00005 | 33596 | 35473 | 2 | + | 1878 | Methyl-accepting chemotaxis protein |
| CDS | contig00005 | 35989 | 37773 | 1 | + | 1785 | Succinate dehydrogenase flavoprotein subunit (EC 1.3.99.1) |
| CDS | contig00005 | 37775 | 38500 | 2 | + | 726 | Succinate dehydrogenase iron-sulfur protein (EC 1.3.99.1) |
| CDS | contig00005 | 38517 | 38915 | 3 | + | 399 | Fumarate reductase subunit C |
| CDS | contig00005 | 39028 | 39384 | 1 | + | 357 | Fumarate reductase subunit D |
| CDS | contig00005 | 40192 | 39494 | -1 | - | 699 | FIG01111792: hypothetical protein |
| CDS | contig00005 | 42158 | 40938 | -2 | - | 1221 | Putative cytoplasmic protein clustered with trehalase |
| CDS | contig00005 | 42874 | 42233 | -1 | - | 642 | Putative methyltransferase associated with DUF414 |
| CDS | contig00005 | 43062 | 43727 | 3 | + | 666 | Thermostable hemolysin delta-VPH |
| CDS | contig00005 | 43724 | 45205 | 2 | + | 1482 | Long-chain-fatty-acid--CoA ligase (EC 6.2.1.3) / long-chain acyl-CoA synthetase |
| CDS | contig00005 | 45202 | 45858 | 1 | + | 657 | long-chain acyl-CoA synthetase |
| CDS | contig00005 | 45855 | 46703 | 3 | + | 849 | Short-chain dehydrogenase/reductase SDR |
| CDS | contig00005 | 46705 | 47352 | 1 | + | 648 | FIG01200150: hypothetical protein |
| CDS | contig00005 | 47434 | 48126 | 1 | + | 693 | Two-component system response regulator QseB |
| CDS | contig00005 | 48123 | 49562 | 3 | + | 1440 | Sensor protein basS/pmrB (EC 2.7.3.-) |
| CDS | contig00005 | 50319 | 49630 | -3 | - | 690 | FIG00361333: hypothetical protein |
| CDS | contig00005 | 50501 | 51544 | 2 | + | 1044 | GMP reductase (EC 1.7.1.7) |
| CDS | contig00005 | 51640 | 52836 | 1 | + | 1197 | FIG00361581: hypothetical protein |
| CDS | contig00005 | 52882 | 53448 | 1 | + | 567 | Translation elongation factor P |
| CDS | contig00005 | 54214 | 53543 | -1 | - | 672 | Methionine ABC transporter permease protein |
| CDS | contig00005 | 54993 | 54199 | -3 | - | 795 | Methionine ABC transporter ATP-binding protein |
| CDS | contig00005 | 55802 | 54990 | -2 | - | 813 | Methionine ABC transporter substrate-binding protein |
| CDS | contig00005 | 56864 | 55824 | -2 | - | 1041 | 2-Oxobutyrate oxidase, putative |
| CDS | contig00005 | 57175 | 57945 | 1 | + | 771 | FIG00362087: hypothetical protein |
| CDS | contig00005 | 58062 | 59018 | 3 | + | 957 | D-3-phosphoglycerate dehydrogenase (EC 1.1.1.95) |
| CDS | contig00005 | 59578 | 59105 | -1 | - | 474 | Glycine-rich cell wall structural protein precursor |
| CDS | contig00005 | 59600 | 59722 | 2 | + | 123 | hypothetical protein |
| CDS | contig00005 | 61364 | 59820 | -2 | - | 1545 | diguanylate cyclase/phosphodiesterase (GGDEF & EAL domains) with PAS/PAC sensor(s) |
| CDS | contig00005 | 61655 | 62257 | 2 | + | 603 | Nitroreductase family protein |
| CDS | contig00005 | 62422 | 63825 | 1 | + | 1404 | FOG: GGDEF domain |
| CDS | contig00005 | 64518 | 63847 | -3 | - | 672 | Putative phosphatase YfbT |
| CDS | contig00005 | 64739 | 64515 | -2 | - | 225 | FIG00361701: hypothetical protein |
| CDS | contig00005 | 64854 | 65741 | 3 | + | 888 | Transcriptional regulators, LysR family |
| CDS | contig00005 | 66422 | 65787 | -2 | - | 636 | Cold shock domain family protein |
| CDS | contig00005 | 67092 | 66526 | -3 | - | 567 | Isochorismatase (EC 3.3.2.1) |
| CDS | contig00005 | 68396 | 67233 | -2 | - | 1164 | 2-octaprenyl-3-methyl-6-methoxy-1,4-benzoquinol hydroxylase (EC 1.14.13.-) |
| CDS | contig00005 | 68621 | 70054 | 2 | + | 1434 | tRNA-i(6)A37 methylthiotransferase |
| CDS | contig00005 | 70105 | 71163 | 1 | + | 1059 | Phosphate starvation-inducible protein PhoH, predicted ATPase |
| CDS | contig00005 | 71160 | 71624 | 3 | + | 465 | Metal-dependent hydrolase YbeY, involved in rRNA and/or ribosome maturation and assembly |
| CDS | contig00005 | 71720 | 72601 | 2 | + | 882 | Magnesium and cobalt efflux protein CorC |
| CDS | contig00005 | 72782 | 74311 | 2 | + | 1530 | Apolipoprotein N-acyltransferase (EC 2.3.1.-) / Copper homeostasis protein CutE |
| CDS | contig00005 | 74885 | 74397 | -2 | - | 489 | FIG002095: hypothetical protein |
| CDS | contig00005 | 75167 | 77704 | 2 | + | 2538 | Leucyl-tRNA synthetase (EC 6.1.1.4) |
| CDS | contig00005 | 77817 | 78299 | 3 | + | 483 | LPS-assembly lipoprotein RlpB precursor (Rare lipoprotein B) |
| CDS | contig00005 | 78327 | 78470 | 3 | + | 144 | DNA polymerase III delta subunit (EC 2.7.7.7) |
| CDS | contig00005 | 78470 | 79504 | 2 | + | 1035 | DNA polymerase III delta subunit (EC 2.7.7.7) |
| CDS | contig00005 | 79498 | 80151 | 1 | + | 654 | Nicotinate-nucleotide adenylyltransferase (EC 2.7.7.18) |
| CDS | contig00005 | 80364 | 80705 | 3 | + | 342 | Iojap protein |
| CDS | contig00005 | 80727 | 81194 | 3 | + | 468 | LSU m3Psi1915 methyltransferase RlmH |
| CDS | contig00005 | 81259 | 83172 | 1 | + | 1914 | Penicillin-binding protein 2 (PBP-2) |
| CDS | contig00005 | 83169 | 84272 | 3 | + | 1104 | Rod shape-determining protein RodA |
| CDS | contig00005 | 84272 | 85270 | 2 | + | 999 | Membrane-bound lytic murein transglycosylase B precursor (EC 3.2.1.-) |
| CDS | contig00005 | 85251 | 86141 | 3 | + | 891 | Rare lipoprotein A precursor |
| CDS | contig00005 | 86307 | 87536 | 3 | + | 1230 | D-alanyl-D-alanine carboxypeptidase (EC 3.4.16.4) |
| CDS | contig00005 | 87816 | 88079 | 3 | + | 264 | Proposed lipoate regulatory protein YbeD |
| CDS | contig00005 | 88173 | 88865 | 3 | + | 693 | Octanoate-[acyl-carrier-protein]-protein-N-octanoyltransferase |
| CDS | contig00005 | 88969 | 89916 | 1 | + | 948 | Lipoate synthase |
| CDS | contig00005 | 90061 | 90549 | 1 | + | 489 | Histone acetyltransferase HPA2 and related acetyltransferases |
| CDS | contig00005 | 91182 | 90583 | -3 | - | 600 | Na(+)-translocating NADH-quinone reductase subunit E (EC 1.6.5.-) |
| CDS | contig00005 | 91678 | 91385 | -1 | - | 294 | hypothetical protein |
| CDS | contig00005 | 91764 | 91901 | 3 | + | 138 | hypothetical protein |
| CDS | contig00005 | 92488 | 91985 | -1 | - | 504 | PhnO protein |
| CDS | contig00005 | 93719 | 92529 | -2 | - | 1191 | Transcriptional regulator, MerR family |
| CDS | contig00005 | 95591 | 93861 | -2 | - | 1731 | Single-stranded-DNA-specific exonuclease RecJ (EC 3.1.-.-) |
| CDS | contig00005 | 96237 | 95557 | -3 | - | 681 | Thiol:disulfide interchange protein DsbC |
| CDS | contig00005 | 96521 | 96931 | 2 | + | 411 | Large-conductance mechanosensitive channel |
| CDS | contig00005 | 97903 | 97004 | -1 | - | 900 | Site-specific recombinase XerD |
| CDS | contig00005 | 98087 | 98605 | 2 | + | 519 | Flavodoxin 2 |
| CDS | contig00005 | 99116 | 100516 | 2 | + | 1401 | TldD protein, part of TldE/TldD proteolytic complex |
| CDS | contig00005 | 100598 | 100479 | -2 | - | 120 | hypothetical protein |
| CDS | contig00005 | 100623 | 101963 | 3 | + | 1341 | TldE protein, part of TldE/TldD proteolytic complex |
| CDS | contig00005 | 102105 | 102482 | 3 | + | 378 | Glyoxalase family protein |
| CDS | contig00005 | 104010 | 103084 | -3 | - | 927 | L,D-transpeptidase YbiS |
| CDS | contig00005 | 104454 | 105818 | 3 | + | 1365 | Putative protease |
| CDS | contig00005 | 105820 | 106068 | 1 | + | 249 | Ferredoxin |
| CDS | contig00005 | 106769 | 106377 | -2 | - | 393 | Protein yqjC precursor |
| CDS | contig00005 | 106930 | 107283 | 1 | + | 354 | Putative ACR protein |
| CDS | contig00005 | 108498 | 107287 | -3 | - | 1212 | Permease of the major facilitator superfamily |
| CDS | contig00005 | 110779 | 108641 | -1 | - | 2139 | Polyribonucleotide nucleotidyltransferase (EC 2.7.7.8) |
| CDS | contig00005 | 111229 | 110960 | -1 | - | 270 | SSU ribosomal protein S15p (S13e) |
| CDS | contig00005 | 112315 | 111365 | -1 | - | 951 | tRNA pseudouridine synthase B (EC 4.2.1.70) |
| CDS | contig00005 | 112751 | 112317 | -2 | - | 435 | Ribosome-binding factor A |
| CDS | contig00005 | 115509 | 112807 | -3 | - | 2703 | Translation initiation factor 2 |
| CDS | contig00005 | 117032 | 115530 | -2 | - | 1503 | Transcription termination protein NusA |
| CDS | contig00005 | 117484 | 117047 | -1 | - | 438 | FIG000325: clustered with transcription termination protein NusA |
| CDS | contig00005 | 118258 | 117932 | -1 | - | 327 | Preprotein translocase subunit SecG (TC 3.A.5.1.1) |
| CDS | contig00005 | 118777 | 118259 | -1 | - | 519 | Triosephosphate isomerase (EC 5.3.1.1) |
| CDS | contig00005 | 120489 | 119155 | -3 | - | 1335 | Phosphoglucosamine mutase (EC 5.4.2.10) |
| CDS | contig00005 | 121353 | 120508 | -3 | - | 846 | Dihydropteroate synthase (EC 2.5.1.15) |
| CDS | contig00005 | 123381 | 121438 | -3 | - | 1944 | Cell division protein FtsH (EC 3.4.24.-) |
| CDS | contig00005 | 124065 | 123436 | -3 | - | 630 | Heat shock protein FtsJ/RrmJ @ Ribosomal RNA large subunit methyltransferase E (EC 2.1.1.-) |
| CDS | contig00005 | 124163 | 124459 | 2 | + | 297 | FIG004454: RNA binding protein |
| CDS | contig00005 | 124565 | 125158 | 2 | + | 594 | hypothetical protein |
| CDS | contig00005 | 125667 | 125206 | -3 | - | 462 | Transcription elongation factor GreA |
| CDS | contig00005 | 126721 | 125828 | -1 | - | 894 | Flagellar motor rotation protein MotB |
| CDS | contig00005 | 127490 | 126732 | -2 | - | 759 | Flagellar motor rotation protein MotA |
| CDS | contig00005 | 127709 | 127524 | -2 | - | 186 | hypothetical protein |
| CDS | contig00005 | 127725 | 127976 | 3 | + | 252 | Exodeoxyribonuclease VII small subunit (EC 3.1.11.6) |
| CDS | contig00005 | 127977 | 128867 | 3 | + | 891 | Octaprenyl-diphosphate synthase (EC 2.5.1.-) / Dimethylallyltransferase (EC 2.5.1.1) / Geranyltranstransferase (farnesyldiphosphate synthase) (EC 2.5.1.10) / Geranylgeranyl pyrophosphate synthetase (EC 2.5.1.29) |
| CDS | contig00005 | 128889 | 130754 | 3 | + | 1866 | 1-deoxy-D-xylulose 5-phosphate synthase (EC 2.2.1.7) |
| CDS | contig00005 | 131917 | 130976 | -1 | - | 942 | Dipeptide transport system permease protein DppC (TC 3.A.1.5.2) |
| CDS | contig00005 | 132895 | 131921 | -1 | - | 975 | Peptide ABC transporter, permease component |
| CDS | contig00005 | 134548 | 132992 | -1 | - | 1557 | Dipeptide-binding ABC transporter, periplasmic substrate-binding component (TC 3.A.1.5.2) |
| CDS | contig00005 | 136331 | 134607 | -2 | - | 1725 | Dipeptide transport ATP-binding protein DppD (TC 3.A.1.5.2) |
| CDS | contig00005 | 137466 | 136978 | -3 | - | 489 | Phosphatidylglycerophosphatase A (EC 3.1.3.27) |
| CDS | contig00005 | 138440 | 137463 | -2 | - | 978 | Thiamine-monophosphate kinase (EC 2.7.4.16) |
| CDS | contig00005 | 138893 | 138480 | -2 | - | 414 | Transcription termination protein NusB |
| CDS | contig00005 | 139378 | 138908 | -1 | - | 471 | 6,7-dimethyl-8-ribityllumazine synthase (EC 2.5.1.78) |
| CDS | contig00005 | 140621 | 139512 | -2 | - | 1110 | 3,4-dihydroxy-2-butanone 4-phosphate synthase (EC 4.1.99.12) / GTP cyclohydrolase II (EC 3.5.4.25) |
| CDS | contig00005 | 141460 | 140759 | -1 | - | 702 | Riboflavin synthase eubacterial/eukaryotic (EC 2.5.1.9) |
| CDS | contig00005 | 142577 | 141468 | -2 | - | 1110 | Diaminohydroxyphosphoribosylaminopyrimidine deaminase (EC 3.5.4.26) / 5-amino-6-(5-phosphoribosylamino)uracil reductase (EC 1.1.1.193) |
| CDS | contig00005 | 142566 | 142793 | 3 | + | 228 | hypothetical protein |
| CDS | contig00005 | 143304 | 142855 | -3 | - | 450 | Ribonucleotide reductase transcriptional regulator NrdR |
| CDS | contig00005 | 143879 | 143439 | -2 | - | 441 | hypothetical protein |
| CDS | contig00005 | 145214 | 143961 | -2 | - | 1254 | Serine hydroxymethyltransferase (EC 2.1.2.1) |
| CDS | contig00005 | 145556 | 145843 | 2 | + | 288 | hypothetical protein |
| CDS | contig00005 | 145994 | 146662 | 2 | + | 669 | Chaperone protein TorD |
| CDS | contig00005 | 146670 | 147038 | 3 | + | 369 | FIG00361216: hypothetical protein |
| CDS | contig00005 | 147145 | 148935 | 1 | + | 1791 | diguanylate cyclase/phosphodiesterase (GGDEF & EAL domains) with PAS/PAC sensor(s) |
| CDS | contig00005 | 149111 | 151057 | 2 | + | 1947 | Acetyl-coenzyme A synthetase (EC 6.2.1.1) |
| CDS | contig00005 | 151341 | 151790 | 3 | + | 450 | 3-dehydroquinate dehydratase II (EC 4.2.1.10) |
| CDS | contig00005 | 151827 | 152288 | 3 | + | 462 | Biotin carboxyl carrier protein of acetyl-CoA carboxylase |
| CDS | contig00005 | 152304 | 153644 | 3 | + | 1341 | Biotin carboxylase of acetyl-CoA carboxylase (EC 6.3.4.14) |
| CDS | contig00005 | 153914 | 154162 | 2 | + | 249 | FIG003021: Membrane protein |
| CDS | contig00005 | 154152 | 155609 | 3 | + | 1458 | Pantothenate:Na+ symporter (TC 2.A.21.1.1) |
| CDS | contig00005 | 155697 | 157121 | 3 | + | 1425 | Phosphotransferase system IIC components, glucose/maltose/N- acetylglucosamine-specific |
| CDS | contig00005 | 157994 | 157122 | -2 | - | 873 | Transcriptional regulator, LysR family |
| CDS | contig00005 | 158095 | 159111 | 1 | + | 1017 | Bifunctional protein: zinc-containing alcohol dehydrogenase; quinone oxidoreductase ( NADPH:quinone reductase) (EC 1.1.1.-); Similar to arginate lyase |
| CDS | contig00005 | 159643 | 160521 | 1 | + | 879 | Ribosomal protein L11 methyltransferase (EC 2.1.1.-) |
| CDS | contig00005 | 160781 | 161329 | 2 | + | 549 | Nodulation protein L |
| CDS | contig00005 | 162429 | 161350 | -3 | - | 1080 | Putative methyl-accepting chemotaxis protein |
| CDS | contig00005 | 163516 | 162968 | -1 | - | 549 | FIG002003: Protein YdjA |
| CDS | contig00005 | 165681 | 163624 | -3 | - | 2058 | 2,4-dienoyl-CoA reductase [NADPH] (EC 1.3.1.34) |
| CDS | contig00005 | 165676 | 165798 | 1 | + | 123 | hypothetical protein |
| CDS | contig00005 | 165974 | 167818 | 2 | + | 1845 | Signal peptide peptidase SppA (EC 3.4.21.-) |
| CDS | contig00005 | 168438 | 167881 | -3 | - | 558 | Nudix dNTPase DR0274 (EC 3.6.1.-) |
| CDS | contig00005 | 169238 | 168435 | -2 | - | 804 | FIG00920727: hypothetical protein |
| CDS | contig00005 | 169450 | 170883 | 1 | + | 1434 | NADPH-dependent glyceraldehyde-3-phosphate dehydrogenase (EC 1.2.1.13) |
| CDS | contig00005 | 170944 | 171237 | 1 | + | 294 | FIG074102: hypothetical protein |
| CDS | contig00005 | 172388 | 171489 | -2 | - | 900 | L-asparaginase I, cytoplasmic (EC 3.5.1.1) |
| CDS | contig00005 | 172350 | 172655 | 3 | + | 306 | hypothetical protein |
| CDS | contig00005 | 172986 | 174185 | 3 | + | 1200 | Sodium/glutamate symport protein |
| CDS | contig00005 | 176315 | 174444 | -2 | - | 1872 | Methyl-accepting chemotaxis protein |
| CDS | contig00005 | 176812 | 177666 | 1 | + | 855 | Peptide chain release factor 2; programmed frameshift-containing |
| CDS | contig00005 | 177687 | 179222 | 3 | + | 1536 | Lysyl-tRNA synthetase (class II) (EC 6.1.1.6) |
| CDS | contig00005 | 180151 | 179354 | -1 | - | 798 | Phenazine biosynthesis protein PhzF like |
| CDS | contig00005 | 180350 | 182137 | 2 | + | 1788 | Sulfite reductase [NADPH] flavoprotein alpha-component (EC 1.8.1.2) |
| CDS | contig00005 | 182137 | 183849 | 1 | + | 1713 | Sulfite reductase [NADPH] hemoprotein beta-component (EC 1.8.1.2) |
| CDS | contig00005 | 183839 | 184645 | 2 | + | 807 | Phosphoadenylyl-sulfate reductase [thioredoxin] (EC 1.8.4.8) |
| CDS | contig00005 | 185740 | 184739 | -1 | - | 1002 | L-proline glycine betaine binding ABC transporter protein ProX (TC 3.A.1.12.1) |
| CDS | contig00005 | 186885 | 185737 | -3 | - | 1149 | L-proline glycine betaine ABC transport system permease protein ProW (TC 3.A.1.12.1) |
| CDS | contig00005 | 188078 | 186882 | -2 | - | 1197 | L-proline glycine betaine ABC transport system permease protein ProV (TC 3.A.1.12.1) |
| CDS | contig00005 | 189043 | 188552 | -1 | - | 492 | Dihydrofolate reductase (EC 1.5.1.3) |
| CDS | contig00005 | 189535 | 189068 | -1 | - | 468 | FIG001826: putative inner membrane protein |
| CDS | contig00005 | 190305 | 189532 | -3 | - | 774 | FIG023911: putative membrane protein |
| CDS | contig00005 | 190900 | 190562 | -1 | - | 339 | hypothetical protein |
| CDS | contig00005 | 191263 | 191838 | 1 | + | 576 | Molybdopterin biosynthesis molybdochelatase MogA |
| CDS | contig00005 | 192283 | 191870 | -1 | - | 414 | hypothetical protein |
| CDS | contig00005 | 193180 | 192425 | -1 | - | 756 | DNA/RNA endonuclease G |
| CDS | contig00005 | 193826 | 194551 | 2 | + | 726 | Carbonic anhydrase (EC 4.2.1.1) |
| CDS | contig00005 | 195749 | 194841 | -2 | - | 909 | Pseudouridine 5&#39;-phosphate glycosidase |
| CDS | contig00005 | 196834 | 195743 | -1 | - | 1092 | Pseudouridine kinase (EC 2.7.1.83) |
| CDS | contig00005 | 197383 | 196979 | -1 | - | 405 | Nitrite reductase [NAD(P)H] small subunit (EC 1.7.1.4) |
| CDS | contig00005 | 199962 | 197380 | -3 | - | 2583 | Nitrite reductase [NAD(P)H] large subunit (EC 1.7.1.4) |
| CDS | contig00005 | 200810 | 199989 | -2 | - | 822 | Nitrate ABC transporter, ATP-binding protein |
| CDS | contig00005 | 201819 | 200842 | -3 | - | 978 | Cyanate ABC transporter, permease protein |
| CDS | contig00005 | 203171 | 201816 | -2 | - | 1356 | Nitrate ABC transporter, nitrate-binding protein |
| CDS | contig00005 | 203157 | 203480 | 3 | + | 324 | hypothetical protein |
| CDS | contig00005 | 204101 | 203517 | -2 | - | 585 | Response regulator NasT |
| CDS | contig00005 | 204985 | 204227 | -1 | - | 759 | Uroporphyrinogen-III methyltransferase (EC 2.1.1.107) |
| CDS | contig00005 | 207638 | 205014 | -2 | - | 2625 | Assimilatory nitrate reductase large subunit (EC:1.7.99.4) |
| CDS | contig00005 | 207657 | 207926 | 3 | + | 270 | hypothetical protein |
| CDS | contig00005 | 207923 | 208426 | 2 | + | 504 | tRNA (guanosine(18)-2&#39;-O)-methyltransferase (EC 2.1.1.34) |
| CDS | contig00005 | 209213 | 208470 | -2 | - | 744 | hypothetical protein |
| CDS | contig00005 | 209408 | 209839 | 2 | + | 432 | Universal stress protein family |
| CDS | contig00005 | 210022 | 209909 | -1 | - | 114 | hypothetical protein |
| CDS | contig00005 | 210644 | 210009 | -2 | - | 636 | hypothetical protein |
| CDS | contig00005 | 210837 | 210712 | -3 | - | 126 | hypothetical protein |
| CDS | contig00005 | 212054 | 210801 | -2 | - | 1254 | Gamma-glutamyl phosphate reductase (EC 1.2.1.41) |
| CDS | contig00005 | 213061 | 212090 | -1 | - | 972 | Glutamate 5-kinase (EC 2.7.2.11) / RNA-binding C-terminal domain PUA |
| CDS | contig00005 | 213080 | 213217 | 2 | + | 138 | hypothetical protein |
| CDS | contig00005 | 213731 | 213339 | -2 | - | 393 | Curlin genes transcriptional activator |
| CDS | contig00005 | 215062 | 213794 | -1 | - | 1269 | Fermentation/respiration switch protein |
| CDS | contig00005 | 215704 | 215237 | -1 | - | 468 | Xanthine-guanine phosphoribosyltransferase (EC 2.4.2.22) |
| CDS | contig00005 | 216261 | 216148 | -3 | - | 114 | hypothetical protein |
| CDS | contig00005 | 216672 | 216283 | -3 | - | 390 | hypothetical protein |
| CDS | contig00005 | 216631 | 218082 | 1 | + | 1452 | Aminoacyl-histidine dipeptidase (Peptidase D) (EC 3.4.13.3) |
| CDS | contig00005 | 218347 | 218466 | 1 | + | 120 | hypothetical protein |
| CDS | contig00005 | 219722 | 218712 | -2 | - | 1011 | Oligopeptide transport ATP-binding protein OppF (TC 3.A.1.5.1) |
| CDS | contig00005 | 220842 | 219805 | -3 | - | 1038 | Oligopeptide transport ATP-binding protein OppD (TC 3.A.1.5.1) |
| CDS | contig00005 | 222685 | 220868 | -1 | - | 1818 | Oligopeptide ABC transporter, periplasmic oligopeptide-binding protein OppA (TC 3.A.1.5.1) |
| CDS | contig00005 | 223811 | 222768 | -2 | - | 1044 | Oligopeptide transport system permease protein OppC (TC 3.A.1.5.1) |
| CDS | contig00005 | 224809 | 223814 | -1 | - | 996 | Oligopeptide transport system permease protein OppB (TC 3.A.1.5.1) |
| CDS | contig00005 | 225636 | 228182 | 3 | + | 2547 | TonB-dependent receptor |
| CDS | contig00005 | 228285 | 229049 | 3 | + | 765 | TonB system biopolymer transport component; Chromosome segregation ATPase |
| CDS | contig00005 | 229046 | 230419 | 2 | + | 1374 | MotA/TolQ/ExbB proton channel family protein |
| CDS | contig00005 | 230430 | 230957 | 3 | + | 528 | Ferric siderophore transport system, biopolymer transport protein ExbB |
| CDS | contig00005 | 231024 | 231431 | 3 | + | 408 | Biopolymer transport protein ExbD/TolR |
| CDS | contig00005 | 231448 | 232083 | 1 | + | 636 | Ferric siderophore transport system, periplasmic binding protein TonB |
| CDS | contig00005 | 232095 | 233330 | 3 | + | 1236 | TPR domain protein, putative component of TonB system |
| CDS | contig00005 | 234793 | 234005 | -1 | - | 789 | Chitodextrinase precursor (EC 3.2.1.14) |
| CDS | contig00006 | 23 | 2938 | 2 | + | 2916 | Alkaline phosphatase (EC 3.1.3.1) |
| CDS | contig00006 | 3026 | 3583 | 2 | + | 558 | hypothetical protein |
| CDS | contig00006 | 3638 | 4339 | 2 | + | 702 | Protein of unknown function DUF541 |
| CDS | contig00006 | 5877 | 4501 | -3 | - | 1377 | Aromatic amino acid transport protein AroP |
| CDS | contig00006 | 6234 | 6509 | 3 | + | 276 | hypothetical protein |
| CDS | contig00006 | 7452 | 6640 | -3 | - | 813 | FIG137360: hypothetical protein |
| CDS | contig00006 | 7760 | 10132 | 2 | + | 2373 | Phosphoenolpyruvate synthase (EC 2.7.9.2) |
| CDS | contig00006 | 10251 | 11705 | 3 | + | 1455 | Putative molybdenum transport ATP-binding protein modF |
| CDS | contig00006 | 11924 | 11724 | -2 | - | 201 | hypothetical protein |
| CDS | contig00006 | 12460 | 11921 | -1 | - | 540 | Isochorismatase (EC 3.3.2.1) |
| CDS | contig00006 | 12681 | 12878 | 3 | + | 198 | hypothetical protein |
| CDS | contig00006 | 13345 | 12893 | -1 | - | 453 | Lactoylglutathione lyase and related lyases |
| CDS | contig00006 | 13687 | 14508 | 1 | + | 822 | Methionine ABC transporter substrate-binding protein |
| CDS | contig00006 | 15284 | 14562 | -2 | - | 723 | Phosphatidylglycerophosphatase B (EC 3.1.3.27) |
| CDS | contig00006 | 15535 | 16656 | 1 | + | 1122 | Erythronate-4-phosphate dehydrogenase (EC 1.1.1.290) |
| CDS | contig00006 | 16803 | 17819 | 3 | + | 1017 | Aspartate-semialdehyde dehydrogenase (EC 1.2.1.11) |
| CDS | contig00006 | 18152 | 20239 | 2 | + | 2088 | Probable type IV pilus assembly FimV-related transmembrane protein |
| CDS | contig00006 | 20406 | 21215 | 3 | + | 810 | tRNA pseudouridine synthase A (EC 4.2.1.70) |
| CDS | contig00006 | 21335 | 22198 | 2 | + | 864 | Acetyl-coenzyme A carboxyl transferase beta chain (EC 6.4.1.2) |
| CDS | contig00006 | 22213 | 23460 | 1 | + | 1248 | Dihydrofolate synthase (EC 6.3.2.12) @ Folylpolyglutamate synthase (EC 6.3.2.17) |
| CDS | contig00006 | 23667 | 24443 | 3 | + | 777 | DedD protein |
| CDS | contig00006 | 24901 | 24557 | -1 | - | 345 | tRNA 2-thiouridine synthesizing protein E (EC 2.8.1.-) |
| CDS | contig00006 | 27164 | 25014 | -2 | - | 2151 | Putative efflux (PET) family inner membrane protein YccS |
| CDS | contig00006 | 28000 | 27344 | -1 | - | 657 | Putative TEGT family carrier/transport protein |
| CDS | contig00006 | 29117 | 28164 | -2 | - | 954 | Transcriptional regulator, VCA0231 ortholog |
| CDS | contig00006 | 29004 | 29129 | 3 | + | 126 | hypothetical protein |
| CDS | contig00006 | 29131 | 31389 | 1 | + | 2259 | TonB-dependent siderophore receptor |
| CDS | contig00006 | 31487 | 32533 | 2 | + | 1047 | putative esterase |
| CDS | contig00006 | 34234 | 33035 | -1 | - | 1200 | 2-methylaconitate cis-trans isomerase |
| CDS | contig00006 | 37010 | 34392 | -2 | - | 2619 | 2-methylcitrate dehydratase FeS dependent (EC 4.2.1.79) |
| CDS | contig00006 | 38167 | 37010 | -1 | - | 1158 | 2-methylcitrate synthase (EC 2.3.3.5) |
| CDS | contig00006 | 39131 | 38232 | -2 | - | 900 | Methylisocitrate lyase (EC 4.1.3.30) |
| CDS | contig00006 | 39514 | 40089 | 1 | + | 576 | Aerotaxis sensor receptor protein |
| CDS | contig00006 | 40043 | 41068 | 2 | + | 1026 | Methyl-accepting chemotaxis protein |
| CDS | contig00006 | 42764 | 41172 | -2 | - | 1593 | diguanylate cyclase (GGDEF domain) with PAS/PAC sensor |
| CDS | contig00006 | 42853 | 43497 | 1 | + | 645 | Ribosomal large subunit pseudouridine synthase A (EC 4.2.1.70) |
| CDS | contig00006 | 43494 | 44147 | 3 | + | 654 | Hypothetical protein, similar to phosphoserine phosphatase |
| CDS | contig00006 | 44387 | 45541 | 2 | + | 1155 | 4-hydroxyphenylpyruvate dioxygenase (EC 1.13.11.27) |
| CDS | contig00006 | 45534 | 46682 | 3 | + | 1149 | Homogentisate 1,2-dioxygenase (EC 1.13.11.5) |
| CDS | contig00006 | 46693 | 47691 | 1 | + | 999 | Fumarylacetoacetase (EC 3.7.1.2) |
| CDS | contig00006 | 47761 | 48399 | 1 | + | 639 | Maleylacetoacetate isomerase (EC 5.2.1.2) @ Glutathione S-transferase, zeta (EC 2.5.1.18) |
| CDS | contig00006 | 49095 | 48472 | -3 | - | 624 | Uncharacterized glutathione S-transferase-like protein |
| CDS | contig00006 | 49724 | 49224 | -2 | - | 501 | FIG00361184: hypothetical protein |
| CDS | contig00006 | 51556 | 49892 | -1 | - | 1665 | Asparagine synthetase [glutamine-hydrolyzing] (EC 6.3.5.4) |
| CDS | contig00006 | 52130 | 51762 | -2 | - | 369 | FIG00361201: hypothetical protein |
| CDS | contig00006 | 53021 | 52272 | -2 | - | 750 | Phosphatase NagD predicted to act in N-acetylglucosamine utilization subsystem |
| CDS | contig00006 | 53231 | 53085 | -2 | - | 147 | hypothetical protein |
| CDS | contig00006 | 53223 | 54242 | 3 | + | 1020 | Adenosine deaminase (EC 3.5.4.4) |
| CDS | contig00006 | 55303 | 54317 | -1 | - | 987 | Bifunctional protein: zinc-containing alcohol dehydrogenase; quinone oxidoreductase ( NADPH:quinone reductase) (EC 1.1.1.-); Similar to arginate lyase |
| CDS | contig00006 | 56557 | 55448 | -1 | - | 1110 | N-ethylmaleimide reductase |
| CDS | contig00006 | 58275 | 56734 | -3 | - | 1542 | Magnesium and cobalt efflux protein CorC |
| CDS | contig00006 | 59442 | 58915 | -3 | - | 528 | hypothetical protein |
| CDS | contig00006 | 60726 | 59800 | -3 | - | 927 | Cobalt-zinc-cadmium resistance protein |
| CDS | contig00006 | 61213 | 60968 | -1 | - | 246 | hypothetical protein |
| CDS | contig00006 | 61214 | 63952 | 2 | + | 2739 | Glycolate dehydrogenase (EC 1.1.99.14), subunit GlcD |
| CDS | contig00006 | 63952 | 64440 | 1 | + | 489 | FIG00361393: hypothetical protein |
| CDS | contig00006 | 64567 | 65028 | 1 | + | 462 | Uncharacterized protein |
| CDS | contig00006 | 65209 | 67107 | 1 | + | 1899 | DEAD-box ATP-dependent RNA helicase CshA (EC 3.6.4.13) |
| CDS | contig00006 | 67437 | 67919 | 3 | + | 483 | hypothetical protein |
| CDS | contig00006 | 67921 | 68409 | 1 | + | 489 | diguanylate cyclase/phosphodiesterase (GGDEF & EAL domains) with PAS/PAC sensor(s) |
| CDS | contig00006 | 68366 | 69862 | 2 | + | 1497 | diguanylate cyclase/phosphodiesterase (GGDEF & EAL domains) with PAS/PAC sensor(s) |
| CDS | contig00006 | 70071 | 70976 | 3 | + | 906 | Transcriptional regulator, LysR family |
| CDS | contig00006 | 71141 | 72274 | 2 | + | 1134 | MFS permease |
| CDS | contig00006 | 73660 | 72338 | -1 | - | 1323 | Histidine permease YuiF |
| CDS | contig00006 | 74549 | 73887 | -2 | - | 663 | Ribonuclease T (EC 3.1.13.-) |
| CDS | contig00006 | 74695 | 76668 | 1 | + | 1974 | Methyl-accepting chemotaxis protein |
| CDS | contig00006 | 76779 | 77612 | 3 | + | 834 | Sodium-type flagellar protein MotY |
| CDS | contig00006 | 78507 | 77713 | -3 | - | 795 | Inositol-1-monophosphatase (EC 3.1.3.25) |
| CDS | contig00006 | 78771 | 79172 | 3 | + | 402 | hypothetical protein |
| CDS | contig00006 | 79292 | 79900 | 2 | + | 609 | Glutathione S-transferase (EC 2.5.1.18) |
| CDS | contig00006 | 79932 | 80594 | 3 | + | 663 | Glutathione S-transferase (EC 2.5.1.18) |
| CDS | contig00006 | 80790 | 82316 | 3 | + | 1527 | Oligopeptide ABC transporter, periplasmic oligopeptide-binding protein OppA (TC 3.A.1.5.1) |
| CDS | contig00006 | 82313 | 83275 | 2 | + | 963 | Oligopeptide transport system permease protein OppB (TC 3.A.1.5.1) |
| CDS | contig00006 | 83278 | 84084 | 1 | + | 807 | Dipeptide transport system permease protein DppC (TC 3.A.1.5.2) |
| CDS | contig00006 | 84081 | 85511 | 3 | + | 1431 | ABC transporter, ATP-binding protein |
| CDS | contig00006 | 85782 | 86981 | 3 | + | 1200 | Multidrug resistance protein D |
| CDS | contig00006 | 86936 | 87250 | 2 | + | 315 | hypothetical protein |
| CDS | contig00006 | 87646 | 87314 | -1 | - | 333 | Z-ring-associated protein ZapA |
| CDS | contig00006 | 87820 | 88389 | 1 | + | 570 | FIG001590: Putative conserved exported protein precursor |
| CDS | contig00006 | 88577 | 89800 | 2 | + | 1224 | 2-octaprenyl-6-methoxyphenol hydroxylase (EC 1.14.13.-) |
| CDS | contig00006 | 89901 | 91121 | 3 | + | 1221 | 2-octaprenyl-3-methyl-6-methoxy-1,4-benzoquinol hydroxylase (EC 1.14.13.-) |
| CDS | contig00006 | 91503 | 92600 | 3 | + | 1098 | Aminomethyltransferase (glycine cleavage system T protein) (EC 2.1.2.10) |
| CDS | contig00006 | 92677 | 93066 | 1 | + | 390 | Glycine cleavage system H protein |
| CDS | contig00006 | 93299 | 96175 | 2 | + | 2877 | Glycine dehydrogenase [decarboxylating] (glycine cleavage system P protein) (EC 1.4.4.2) |
| CDS | contig00006 | 96748 | 96263 | -1 | - | 486 | 3-demethylubiquinone-9 3-methyltransferase |
| CDS | contig00006 | 96914 | 97726 | 2 | + | 813 | Methyltransferase (EC 2.1.1.-) |
| CDS | contig00006 | 98087 | 97851 | -2 | - | 237 | hypothetical protein |
| CDS | contig00006 | 98213 | 99532 | 2 | + | 1320 | L-Proline/Glycine betaine transporter ProP |
| CDS | contig00006 | 101187 | 99601 | -3 | - | 1587 | Alkyl hydroperoxide reductase protein F (EC 1.6.4.-) |
| CDS | contig00006 | 101896 | 101330 | -1 | - | 567 | Alkyl hydroperoxide reductase protein C (EC 1.6.4.-) |
| CDS | contig00006 | 102972 | 102526 | -3 | - | 447 | Putative acetoin utilization protein AcuB |
| CDS | contig00006 | 103184 | 104251 | 2 | + | 1068 | S-adenosylmethionine:tRNA ribosyltransferase-isomerase (EC 5.-.-.-) |
| CDS | contig00006 | 104370 | 105506 | 3 | + | 1137 | tRNA-guanine transglycosylase (EC 2.4.2.29) |
| CDS | contig00006 | 105557 | 105892 | 2 | + | 336 | Preprotein translocase subunit YajC (TC 3.A.5.1.1) |
| CDS | contig00006 | 105911 | 107764 | 2 | + | 1854 | Protein-export membrane protein SecD (TC 3.A.5.1.1) |
| CDS | contig00006 | 107774 | 108721 | 2 | + | 948 | Protein-export membrane protein SecF (TC 3.A.5.1.1) |
| CDS | contig00006 | 108822 | 109190 | 3 | + | 369 | Rhodanese domain protein |
| CDS | contig00006 | 109419 | 110213 | 3 | + | 795 | Lipoprotein nlpI precursor |
| CDS | contig00006 | 111161 | 110289 | -2 | - | 873 | FIG139928: Putative protease |
| CDS | contig00006 | 112169 | 111174 | -2 | - | 996 | FIG139552: Putative protease |
| CDS | contig00006 | 112369 | 112253 | -1 | - | 117 | hypothetical protein |
| CDS | contig00006 | 112358 | 112870 | 2 | + | 513 | FIG138517: Putative lipid carrier protein |
| CDS | contig00006 | 113061 | 113795 | 3 | + | 735 | tRNA:Cm32/Um32 methyltransferase |
| CDS | contig00006 | 113920 | 114393 | 1 | + | 474 | Iron-sulfur cluster regulator IscR |
| CDS | contig00006 | 114446 | 115660 | 2 | + | 1215 | Cysteine desulfurase (EC 2.8.1.7), IscS subfamily |
| CDS | contig00006 | 115715 | 116098 | 2 | + | 384 | Iron-sulfur cluster assembly scaffold protein IscU |
| CDS | contig00006 | 116114 | 116437 | 2 | + | 324 | Iron binding protein IscA for iron-sulfur cluster assembly |
| CDS | contig00006 | 116628 | 117146 | 3 | + | 519 | Chaperone protein HscB |
| CDS | contig00006 | 117175 | 119022 | 1 | + | 1848 | Chaperone protein HscA |
| CDS | contig00006 | 119024 | 119362 | 2 | + | 339 | Ferredoxin, 2Fe-2S |
| CDS | contig00006 | 119513 | 119370 | -2 | - | 144 | hypothetical protein |
| CDS | contig00006 | 119520 | 120821 | 3 | + | 1302 | Peptidase B (EC 3.4.11.23) |
| CDS | contig00006 | 120818 | 122101 | 2 | + | 1284 | Peptidase B (EC 3.4.11.23) |
| CDS | contig00006 | 122379 | 122807 | 3 | + | 429 | Nucleoside diphosphate kinase (EC 2.7.4.6) |
| CDS | contig00006 | 122807 | 124078 | 2 | + | 1272 | Ribosomal RNA large subunit methyltransferase N (EC 2.1.1.-) |
| CDS | contig00006 | 124120 | 124881 | 1 | + | 762 | Type IV pilus biogenesis protein PilF |
| CDS | contig00006 | 124871 | 125791 | 2 | + | 921 | FIG021952: putative membrane protein |
| CDS | contig00006 | 125802 | 126932 | 3 | + | 1131 | 1-hydroxy-2-methyl-2-(E)-butenyl 4-diphosphate synthase (EC 1.17.7.1) |
| CDS | contig00006 | 127088 | 126966 | -2 | - | 123 | hypothetical protein |
| CDS | contig00006 | 127101 | 128348 | 3 | + | 1248 | Histidyl-tRNA synthetase (EC 6.1.1.21) |
| CDS | contig00006 | 128352 | 129002 | 3 | + | 651 | Mlr7403 protein |
| CDS | contig00006 | 128995 | 130179 | 1 | + | 1185 | Outer membrane protein YfgL, lipoprotein component of the protein assembly complex (forms a complex with YaeT, YfiO, and NlpB) |
| CDS | contig00006 | 130254 | 131750 | 3 | + | 1497 | GTP-binding protein EngA |
| CDS | contig00006 | 131874 | 132104 | 3 | + | 231 | FIG00613201: hypothetical protein |
| CDS | contig00006 | 132249 | 132127 | -3 | - | 123 | hypothetical protein |
| CDS | contig00006 | 133920 | 132427 | -3 | - | 1494 | Transglycosylase, Slt family |
| CDS | contig00006 | 134117 | 133965 | -2 | - | 153 | hypothetical protein |
| CDS | contig00006 | 134070 | 137963 | 3 | + | 3894 | Phosphoribosylformylglycinamidine synthase, synthetase subunit (EC 6.3.5.3) / Phosphoribosylformylglycinamidine synthase, glutamine amidotransferase subunit (EC 6.3.5.3) |
| CDS | contig00006 | 139696 | 138233 | -1 | - | 1464 | NADH-ubiquinone oxidoreductase chain N (EC 1.6.5.3) |
| CDS | contig00006 | 141257 | 139740 | -2 | - | 1518 | NADH-ubiquinone oxidoreductase chain M (EC 1.6.5.3) |
| CDS | contig00006 | 143174 | 141327 | -2 | - | 1848 | NADH-ubiquinone oxidoreductase chain L (EC 1.6.5.3) |
| CDS | contig00006 | 143464 | 143171 | -1 | - | 294 | NADH-ubiquinone oxidoreductase chain K (EC 1.6.5.3) |
| CDS | contig00006 | 144098 | 143559 | -2 | - | 540 | NADH-ubiquinone oxidoreductase chain J (EC 1.6.5.3) |
| CDS | contig00006 | 144650 | 144108 | -2 | - | 543 | NADH-ubiquinone oxidoreductase chain I (EC 1.6.5.3) |
| CDS | contig00006 | 145706 | 144741 | -2 | - | 966 | NADH-ubiquinone oxidoreductase chain H (EC 1.6.5.3) |
| CDS | contig00006 | 148432 | 145703 | -1 | - | 2730 | NADH-ubiquinone oxidoreductase chain G (EC 1.6.5.3) |
| CDS | contig00006 | 149875 | 148490 | -1 | - | 1386 | NADH-ubiquinone oxidoreductase chain F (EC 1.6.5.3) |
| CDS | contig00006 | 150411 | 149872 | -3 | - | 540 | NADH-ubiquinone oxidoreductase chain E (EC 1.6.5.3) |
| CDS | contig00006 | 152289 | 150484 | -3 | - | 1806 | NADH-ubiquinone oxidoreductase chain C (EC 1.6.5.3) / NADH-ubiquinone oxidoreductase chain D (EC 1.6.5.3) |
| CDS | contig00006 | 152966 | 152292 | -2 | - | 675 | NADH-ubiquinone oxidoreductase chain B (EC 1.6.5.3) |
| CDS | contig00006 | 153400 | 152996 | -1 | - | 405 | NADH ubiquinone oxidoreductase chain A (EC 1.6.5.3) |
| CDS | contig00006 | 154532 | 153657 | -2 | - | 876 | Flagellar motor rotation protein MotB |
| CDS | contig00006 | 155268 | 154510 | -3 | - | 759 | Flagellar motor rotation protein MotA |
| CDS | contig00006 | 157133 | 156462 | -2 | - | 672 | FIG004694: Hypothetical protein |
| CDS | contig00006 | 157836 | 157102 | -3 | - | 735 | Beta-propeller domains of methanol dehydrogenase type |
| CDS | contig00006 | 158438 | 157833 | -2 | - | 606 | LemA family protein |
| CDS | contig00006 | 159224 | 158481 | -2 | - | 744 | Murein endopeptidase |
| CDS | contig00006 | 159399 | 159941 | 3 | + | 543 | hypothetical protein |
| CDS | contig00006 | 160154 | 160432 | 2 | + | 279 | RNA-binding protein |
| CDS | contig00006 | 161401 | 160502 | -1 | - | 900 | Putative oxidoreductase YeaE, aldo/keto reductase family |
| CDS | contig00006 | 161568 | 161975 | 3 | + | 408 | FIG01202129: hypothetical protein |
| CDS | contig00006 | 162051 | 162758 | 3 | + | 708 | D-beta-hydroxybutyrate dehydrogenase (EC 1.1.1.30) |
| CDS | contig00006 | 162845 | 163861 | 2 | + | 1017 | Low-specificity L-threonine aldolase (EC 4.1.2.48) |
| CDS | contig00006 | 164356 | 163925 | -1 | - | 432 | diguanylate cyclase (GGDEF domain) with PAS/PAC sensor |
| CDS | contig00006 | 165411 | 164389 | -3 | - | 1023 | Chemotaxis protein CheC -- inhibitor of MCP methylation |
| CDS | contig00006 | 166402 | 165458 | -1 | - | 945 | Predicted lactate-responsive transcriptional regulator of ykgEFG LDH gene cluster, LysR-type |
| CDS | contig00006 | 167208 | 166486 | -3 | - | 723 | FIG00362163: hypothetical protein |
| CDS | contig00006 | 167294 | 169297 | 2 | + | 2004 | diguanylate cyclase/phosphodiesterase (GGDEF & EAL domains) with PAS/PAC sensor(s) |
| CDS | contig00006 | 169467 | 171014 | 3 | + | 1548 | Aminobenzoyl-glutamate transport protein |
| CDS | contig00006 | 171082 | 171807 | 1 | + | 726 | Oxygen-insensitive NADPH nitroreductase (EC 1.-.-.-) |
| CDS | contig00006 | 171961 | 172725 | 1 | + | 765 | ATPase involved in DNA repair |
| CDS | contig00006 | 172806 | 173273 | 3 | + | 468 | Protein yecM |
| CDS | contig00006 | 174511 | 173258 | -1 | - | 1254 | Possible efflux permease |
| CDS | contig00006 | 174605 | 175507 | 2 | + | 903 | Transcriptional regulator, LysR family |
| CDS | contig00006 | 176167 | 175574 | -1 | - | 594 | GTP cyclohydrolase II (EC 3.5.4.25) |
| CDS | contig00006 | 176431 | 177123 | 1 | + | 693 | Cytidylate kinase (EC 2.7.4.25) |
| CDS | contig00006 | 177217 | 178887 | 1 | + | 1671 | SSU ribosomal protein S1p |
| CDS | contig00006 | 178980 | 179264 | 3 | + | 285 | Integration host factor beta subunit |
| CDS | contig00006 | 179421 | 179705 | 3 | + | 285 | Inner membrane protein yciS |
| CDS | contig00006 | 179715 | 180881 | 3 | + | 1167 | Heat shock (predicted periplasmic) protein YciM, precursor |
| CDS | contig00006 | 181003 | 181701 | 1 | + | 699 | Orotidine 5&#39;-phosphate decarboxylase (EC 4.1.1.23) |
| CDS | contig00006 | 182309 | 181788 | -2 | - | 522 | Rhombosortase |
| CDS | contig00006 | 182332 | 182985 | 1 | + | 654 | hypothetical protein |
| CDS | contig00006 | 183008 | 183715 | 2 | + | 708 | Queuosine Biosynthesis QueC ATPase |
| CDS | contig00006 | 183768 | 184448 | 3 | + | 681 | Queuosine Biosynthesis QueE Radical SAM |
| CDS | contig00006 | 184450 | 184680 | 1 | + | 231 | FIG074102: hypothetical protein |
| CDS | contig00006 | 184677 | 185240 | 3 | + | 564 | ATP:Cob(I)alamin adenosyltransferase (EC 2.5.1.17) |
| CDS | contig00006 | 185716 | 185318 | -1 | - | 399 | DNA-binding protein H-NS |
| CDS | contig00006 | 186307 | 186185 | -1 | - | 123 | hypothetical protein |
| CDS | contig00006 | 186293 | 187873 | 2 | + | 1581 | Predicted lysine transporter, NhaC family |
| CDS | contig00006 | 187959 | 188540 | 3 | + | 582 | Thymidine kinase (EC 2.7.1.21) |
| CDS | contig00006 | 189948 | 188611 | -3 | - | 1338 | FIG00487266: hypothetical protein |
| CDS | contig00006 | 190143 | 190559 | 3 | + | 417 | putative sensory transduction regulator |
| CDS | contig00006 | 191325 | 191203 | -3 | - | 123 | hypothetical protein |
| CDS | contig00006 | 191782 | 192540 | 1 | + | 759 | Phosphosugar-binding transcriptional regulator, RpiR family |
| CDS | contig00006 | 192659 | 194167 | 2 | + | 1509 | PTS system, beta-glucoside-specific IIB component (EC 2.7.1.69) / PTS system, beta-glucoside-specific IIC component / PTS system, beta-glucoside-specific IIA component |
| CDS | contig00006 | 194181 | 195614 | 3 | + | 1434 | 6-phospho-beta-glucosidase (EC 3.2.1.86) |
| CDS | contig00006 | 197187 | 195937 | -3 | - | 1251 | putative exported protein |
| CDS | contig00006 | 198771 | 197254 | -3 | - | 1518 | FIG312471: hypothetical protein |
| CDS | contig00006 | 201276 | 199102 | -3 | - | 2175 | Catalase (EC 1.11.1.6) / Peroxidase (EC 1.11.1.7) |
| CDS | contig00006 | 201298 | 201414 | 1 | + | 117 | hypothetical protein |
| CDS | contig00006 | 202313 | 203170 | 2 | + | 858 | Methylenetetrahydrofolate dehydrogenase (NADP+) (EC 1.5.1.5) / Methenyltetrahydrofolate cyclohydrolase (EC 3.5.4.9) |
| CDS | contig00006 | 204419 | 203256 | -2 | - | 1164 | Methionine gamma-lyase (EC 4.4.1.11) |
| CDS | contig00006 | 205536 | 204514 | -3 | - | 1023 | 2-Oxobutyrate oxidase, putative |
| CDS | contig00006 | 206522 | 205884 | -2 | - | 639 | Rrf2-linked NADH-flavin reductase |
| CDS | contig00006 | 206623 | 207522 | 1 | + | 900 | Transcriptional regulator PtxR |
| CDS | contig00006 | 207714 | 208538 | 3 | + | 825 | Transcriptional regulator, AraC family |
| CDS | contig00006 | 209467 | 208535 | -1 | - | 933 | diguanylate cyclase/phosphodiesterase (GGDEF & EAL domains) with PAS/PAC sensor(s) |
| CDS | contig00006 | 209480 | 209659 | 2 | + | 180 | Methyl-accepting chemotaxis protein I (serine chemoreceptor protein) |
| CDS | contig00007 | 1138 | 926 | -1 | - | 213 | hypothetical protein |
| CDS | contig00007 | 1321 | 2406 | 1 | + | 1086 | hypothetical protein |
| CDS | contig00007 | 2588 | 2944 | 2 | + | 357 | hypothetical protein |
| CDS | contig00007 | 2984 | 3154 | 2 | + | 171 | hypothetical protein |
| CDS | contig00007 | 3201 | 3407 | 3 | + | 207 | hypothetical protein |
| CDS | contig00007 | 4974 | 3475 | -3 | - | 1500 | diguanylate cyclase/phosphodiesterase (GGDEF & EAL domains) with PAS/PAC sensor(s) |
| CDS | contig00007 | 7290 | 6976 | -3 | - | 315 | hypothetical protein |
| CDS | contig00007 | 8095 | 7358 | -1 | - | 738 | hypothetical protein |
| CDS | contig00007 | 9218 | 8760 | -2 | - | 459 | hypothetical protein |
| CDS | contig00007 | 9671 | 9237 | -2 | - | 435 | hypothetical protein |
| CDS | contig00007 | 10303 | 10494 | 1 | + | 192 | hypothetical protein |
| CDS | contig00007 | 11865 | 10963 | -3 | - | 903 | hypothetical protein |
| CDS | contig00007 | 12905 | 12591 | -2 | - | 315 | hypothetical protein |
| CDS | contig00007 | 13877 | 13623 | -2 | - | 255 | hypothetical protein |
| CDS | contig00007 | 16681 | 15449 | -1 | - | 1233 | Chromosome (plasmid) partitioning protein ParA |
| CDS | contig00007 | 17112 | 17321 | 3 | + | 210 | hypothetical protein |
| CDS | contig00007 | 20923 | 18710 | -1 | - | 2214 | hypothetical protein |
| CDS | contig00007 | 22672 | 23013 | 1 | + | 342 | hypothetical protein |
| CDS | contig00007 | 23406 | 23825 | 3 | + | 420 | hypothetical protein |
| CDS | contig00007 | 23837 | 24454 | 2 | + | 618 | Single-stranded DNA-binding protein |
| CDS | contig00007 | 24571 | 25221 | 1 | + | 651 | FIG01049668: hypothetical protein |
| CDS | contig00007 | 26018 | 26578 | 2 | + | 561 | Potential queD like |
| CDS | contig00007 | 28404 | 27082 | -3 | - | 1323 | Phage exonuclease |
| CDS | contig00007 | 29296 | 28478 | -1 | - | 819 | hypothetical protein |
| CDS | contig00007 | 30325 | 29600 | -1 | - | 726 | Thiol:disulfide interchange protein DsbC |
| CDS | contig00007 | 30843 | 30322 | -3 | - | 522 | hypothetical protein |
| CDS | contig00007 | 31245 | 31003 | -3 | - | 243 | hypothetical protein |
| CDS | contig00007 | 31813 | 32439 | 1 | + | 627 | hypothetical protein |
| CDS | contig00007 | 35025 | 33946 | -3 | - | 1080 | Signal recognition particle receptor protein FtsY (=alpha subunit) (TC 3.A.5.1.1) |
| CDS | contig00007 | 35208 | 36878 | 3 | + | 1671 | hypothetical protein |
| CDS | contig00007 | 39681 | 37138 | -3 | - | 2544 | IncF plasmid conjugative transfer pilus assembly protein TraC |
| CDS | contig00007 | 40399 | 39764 | -1 | - | 636 | Conjugative transfer protein TraV |
| CDS | contig00007 | 41090 | 40410 | -2 | - | 681 | Thiol:disulfide involved in conjugative transfer |
| CDS | contig00007 | 42975 | 41218 | -3 | - | 1758 | hypothetical protein |
| CDS | contig00007 | 43832 | 43119 | -2 | - | 714 | hypothetical protein |
| CDS | contig00007 | 44551 | 43943 | -1 | - | 609 | BfpH protein (involved in biogenesis of type IV pili) |
| CDS | contig00007 | 44781 | 44551 | -3 | - | 231 | hypothetical protein |
| CDS | contig00007 | 46272 | 45049 | -3 | - | 1224 | IncF plasmid conjugative transfer pilus assembly protein TraB |
| CDS | contig00007 | 47492 | 46449 | -2 | - | 1044 | IncF plasmid conjugative transfer pilus assembly protein TraK |
| CDS | contig00007 | 48283 | 47621 | -1 | - | 663 | IncF plasmid conjugative transfer pilus assembly protein TraE |
| CDS | contig00007 | 50589 | 48619 | -3 | - | 1971 | ATP-dependent DNA helicase RecG (EC 3.6.1.-) |
| CDS | contig00007 | 50748 | 52589 | 3 | + | 1842 | IncF plasmid conjugative transfer protein TraD |
| CDS | contig00007 | 52591 | 53259 | 1 | + | 669 | hypothetical protein |
| CDS | contig00007 | 53374 | 53706 | 1 | + | 333 | hypothetical protein |
| CDS | contig00007 | 53911 | 54060 | 1 | + | 150 | hypothetical protein |
| CDS | contig00007 | 54199 | 54873 | 1 | + | 675 | hypothetical protein |
| CDS | contig00007 | 55670 | 58066 | 2 | + | 2397 | T1SS secreted agglutinin RTX |
| CDS | contig00007 | 58077 | 60443 | 3 | + | 2367 | T1SS secreted agglutinin RTX |
| CDS | contig00007 | 60651 | 62858 | 3 | + | 2208 | hypothetical protein |
| CDS | contig00007 | 62870 | 67075 | 2 | + | 4206 | hypothetical protein |
| CDS | contig00007 | 67538 | 67927 | 2 | + | 390 | hypothetical protein |
| CDS | contig00007 | 68693 | 68875 | 2 | + | 183 | Phage protein |
| CDS | contig00007 | 69084 | 69209 | 3 | + | 126 | hypothetical protein |
| CDS | contig00007 | 69441 | 69683 | 3 | + | 243 | hypothetical protein |
| CDS | contig00007 | 70041 | 70418 | 3 | + | 378 | Signal peptidase I (EC 3.4.21.89) |
| CDS | contig00007 | 70453 | 71676 | 1 | + | 1224 | IncF plasmid conjugative transfer pilus assembly protein TraW |
| CDS | contig00007 | 71673 | 71801 | 3 | + | 129 | hypothetical protein |
| CDS | contig00007 | 71840 | 72949 | 2 | + | 1110 | IncF plasmid conjugative transfer pilus assembly protein TraU |
| CDS | contig00007 | 73038 | 76166 | 3 | + | 3129 | IncF plasmid conjugative transfer protein TraN |
| CDS | contig00007 | 76568 | 77047 | 2 | + | 480 | hypothetical protein |
| CDS | contig00008 | 1664 | 21 | -2 | - | 1644 | Mobile element protein |
| CDS | contig00008 | 2078 | 1713 | -2 | - | 366 | Mobile element protein |
| CDS | contig00008 | 2380 | 2078 | -1 | - | 303 | hypothetical protein |
| CDS | contig00010 | 67 | 267 | 1 | + | 201 | Mobile element protein |
| CDS | contig00011 | 2247 | 514 | -3 | - | 1734 | hypothetical protein |
| CDS | contig00011 | 5598 | 3088 | -3 | - | 2511 | Putative membrane protein |
| CDS | contig00011 | 7038 | 5632 | -3 | - | 1407 | Phage integrase |
| CDS | contig00011 | 7695 | 7390 | -3 | - | 306 | YebG, DNA damage-inducible gene in SOS regulon, expressed in stationary phase |
| CDS | contig00011 | 7916 | 8560 | 2 | + | 645 | Paraquat-inducible protein A |
| CDS | contig00011 | 8538 | 9146 | 3 | + | 609 | Paraquat-inducible protein A |
| CDS | contig00011 | 9198 | 11750 | 3 | + | 2553 | Paraquat-inducible protein B |
| CDS | contig00011 | 11726 | 11842 | 2 | + | 117 | hypothetical protein |
| CDS | contig00011 | 11945 | 11817 | -2 | - | 129 | hypothetical protein |
| CDS | contig00011 | 11880 | 13310 | 3 | + | 1431 | Ribosomal RNA small subunit methyltransferase F (EC 2.1.1.-) |
| CDS | contig00011 | 13829 | 13545 | -2 | - | 285 | hypothetical protein |
| CDS | contig00011 | 15021 | 13993 | -3 | - | 1029 | Integrase/recombinase xerD homolog |
| CDS | contig00011 | 16102 | 15323 | -1 | - | 780 | Putative deoxyribonuclease YcfH |
| CDS | contig00011 | 17062 | 16115 | -1 | - | 948 | DNA polymerase III delta prime subunit (EC 2.7.7.7) |
| CDS | contig00011 | 17688 | 17047 | -3 | - | 642 | Thymidylate kinase (EC 2.7.4.9) |
| CDS | contig00011 | 18689 | 17688 | -2 | - | 1002 | FIG004453: protein YceG like |
| CDS | contig00011 | 19485 | 18679 | -3 | - | 807 | Aminodeoxychorismate lyase (EC 4.1.3.38) |
| CDS | contig00011 | 20757 | 19552 | -3 | - | 1206 | 3-oxoacyl-[acyl-carrier-protein] synthase, KASII (EC 2.3.1.179) |
| CDS | contig00011 | 21111 | 20875 | -3 | - | 237 | Acyl carrier protein |
| CDS | contig00011 | 22003 | 21269 | -1 | - | 735 | 3-oxoacyl-[acyl-carrier protein] reductase (EC 1.1.1.100) |
| CDS | contig00011 | 22952 | 22017 | -2 | - | 936 | Malonyl CoA-acyl carrier protein transacylase (EC 2.3.1.39) |
| CDS | contig00011 | 23978 | 23019 | -2 | - | 960 | 3-oxoacyl-[acyl-carrier-protein] synthase, KASIII (EC 2.3.1.180) |
| CDS | contig00011 | 24794 | 23985 | -2 | - | 810 | Phosphate:acyl-ACP acyltransferase PlsX |
| CDS | contig00011 | 25181 | 25014 | -2 | - | 168 | LSU ribosomal protein L32p |
| CDS | contig00011 | 25633 | 25202 | -1 | - | 432 | COG1399 protein, clustered with ribosomal protein L32p |
| CDS | contig00011 | 26423 | 25920 | -2 | - | 504 | Collagenase and related proteases |
| CDS | contig00011 | 26674 | 27255 | 1 | + | 582 | FIG146278: Maf/YceF/YhdE family protein |
| CDS | contig00011 | 28290 | 27307 | -3 | - | 984 | Ribosomal large subunit pseudouridine synthase C (EC 4.2.1.70) |
| CDS | contig00011 | 28666 | 31812 | 1 | + | 3147 | Ribonuclease E (EC 3.1.26.12) |
| CDS | contig00011 | 33941 | 32469 | -2 | - | 1473 | Low-affinity inorganic phosphate transporter |
| CDS | contig00011 | 34843 | 34262 | -1 | - | 582 | Deoxycytidine triphosphate deaminase (EC 3.5.4.13) |
| CDS | contig00011 | 35551 | 34907 | -1 | - | 645 | Uridine kinase (EC 2.7.1.48) [C1] |
| CDS | contig00011 | 36742 | 35660 | -1 | - | 1083 | Scaffold protein for [4Fe-4S] cluster assembly ApbC, MRP-like |
| CDS | contig00011 | 36949 | 38961 | 1 | + | 2013 | Methionyl-tRNA synthetase (EC 6.1.1.10) |
| CDS | contig00011 | 39767 | 39036 | -2 | - | 732 | hypothetical protein |
| CDS | contig00011 | 40052 | 39780 | -2 | - | 273 | Acylphosphate phosphohydrolase (EC 3.6.1.7), putative |
| CDS | contig00011 | 40138 | 41568 | 1 | + | 1431 | N-acetylglucosamine regulated methyl-accepting chemotaxis protein |
| CDS | contig00011 | 41752 | 42945 | 1 | + | 1194 | LSU m5C1962 methyltransferase RlmI |
| CDS | contig00011 | 44056 | 43412 | -1 | - | 645 | Maleylacetoacetate isomerase (EC 5.2.1.2) / Glutathione S-transferase |
| CDS | contig00011 | 44316 | 45500 | 3 | + | 1185 | putative acyltransferase |
| CDS | contig00011 | 45849 | 46886 | 3 | + | 1038 | Selenide,water dikinase (EC 2.7.9.3) |
| CDS | contig00011 | 46883 | 48013 | 2 | + | 1131 | Selenophosphate-dependent tRNA 2-selenouridine synthase |
| CDS | contig00011 | 48007 | 48651 | 1 | + | 645 | Hypothetical metal-binding enzyme, YcbL homolog |
| CDS | contig00011 | 48851 | 48627 | -2 | - | 225 | Response regulator |
| CDS | contig00011 | 49627 | 48821 | -1 | - | 807 | Response regulator |
| CDS | contig00011 | 49989 | 49639 | -3 | - | 351 | Two-component system sensor protein |
| CDS | contig00011 | 52808 | 49986 | -2 | - | 2823 | Two-component system sensor protein |
| CDS | contig00011 | 53186 | 53013 | -2 | - | 174 | LSU ribosomal protein L25p |
| CDS | contig00011 | 54297 | 53464 | -3 | - | 834 | hypothetical protein |
| CDS | contig00011 | 58268 | 54369 | -2 | - | 3900 | ATP-dependent RNA helicase HrpA (EC 3.6.4.13) |
| CDS | contig00011 | 58880 | 58458 | -2 | - | 423 | Inosine monophosphate dehydrogenase-related protein |
| CDS | contig00011 | 60034 | 58964 | -1 | - | 1071 | FIG000557: hypothetical protein co-occurring with RecR |
| CDS | contig00011 | 60441 | 61763 | 3 | + | 1323 | Predicted histidine uptake transporter |
| CDS | contig00011 | 62702 | 61821 | -2 | - | 882 | Pirin |
| CDS | contig00011 | 62821 | 63747 | 1 | + | 927 | Transcriptional regulator, LysR family |
| CDS | contig00011 | 64188 | 63859 | -3 | - | 330 | FIG000557: hypothetical protein co-occurring with RecR |
| CDS | contig00011 | 66790 | 64313 | -1 | - | 2478 | DNA polymerase III subunits gamma and tau (EC 2.7.7.7) |
| CDS | contig00011 | 67555 | 66968 | -1 | - | 588 | Adenine phosphoribosyltransferase (EC 2.4.2.7) |
| CDS | contig00011 | 68023 | 67646 | -1 | - | 378 | Hypothetical protein DUF454 |
| CDS | contig00011 | 68200 | 69144 | 1 | + | 945 | tRNA-dihydrouridine synthase C (EC 1.-.-.-) |
| CDS | contig00011 | 69201 | 70028 | 3 | + | 828 | L-rhamnose operon transcriptional activator RhaR |
| CDS | contig00011 | 70103 | 71068 | 2 | + | 966 | FIG006442: Integral membrane protein |
| CDS | contig00011 | 71165 | 71518 | 2 | + | 354 | DksA-type zinc finger protein |
| CDS | contig00011 | 71518 | 71634 | 1 | + | 117 | hypothetical protein |
| CDS | contig00011 | 72882 | 71677 | -3 | - | 1206 | Ribonuclease D (EC 3.1.26.3) |
| CDS | contig00011 | 74579 | 72888 | -2 | - | 1692 | Long-chain-fatty-acid--CoA ligase (EC 6.2.1.3) |
| CDS | contig00011 | 75612 | 74761 | -3 | - | 852 | Predicted hydrolase/acyltransferase |
| CDS | contig00011 | 76137 | 75622 | -3 | - | 516 | Starvation lipoprotein Slp paralog |
| CDS | contig00011 | 76888 | 76187 | -1 | - | 702 | TsaB protein, required for threonylcarbamoyladenosine (t(6)A) formation in tRNA |
| CDS | contig00011 | 78899 | 76986 | -2 | - | 1914 | DinG family ATP-dependent helicase YoaA |
| CDS | contig00011 | 79212 | 78967 | -3 | - | 246 | hypothetical protein |
| CDS | contig00011 | 79556 | 80452 | 2 | + | 897 | ATP phosphoribosyltransferase (EC 2.4.2.17) |
| CDS | contig00011 | 80484 | 81809 | 3 | + | 1326 | Histidinol dehydrogenase (EC 1.1.1.23) |
| CDS | contig00011 | 81806 | 82876 | 2 | + | 1071 | Histidinol-phosphate aminotransferase (EC 2.6.1.9) |
| CDS | contig00011 | 82896 | 84029 | 3 | + | 1134 | Histidinol-phosphatase (EC 3.1.3.15) / Imidazoleglycerol-phosphate dehydratase (EC 4.2.1.19) |
| CDS | contig00011 | 84029 | 84652 | 2 | + | 624 | Imidazole glycerol phosphate synthase amidotransferase subunit (EC 2.4.2.-) |
| CDS | contig00011 | 84658 | 85404 | 1 | + | 747 | Phosphoribosylformimino-5-aminoimidazole carboxamide ribotide isomerase (EC 5.3.1.16) |
| CDS | contig00011 | 85537 | 86646 | 1 | + | 1110 | Response regulator |
| CDS | contig00011 | 86669 | 88777 | 2 | + | 2109 | Sensory box/GGDEF family protein |
| CDS | contig00011 | 88774 | 92745 | 1 | + | 3972 | diguanylate cyclase/phosphodiesterase (GGDEF & EAL domains) with PAS/PAC sensor(s) |
| CDS | contig00011 | 92924 | 93697 | 2 | + | 774 | Imidazole glycerol phosphate synthase cyclase subunit (EC 4.1.3.-) |
| CDS | contig00011 | 93690 | 94325 | 3 | + | 636 | Phosphoribosyl-AMP cyclohydrolase (EC 3.5.4.19) / Phosphoribosyl-ATP pyrophosphatase (EC 3.6.1.31) |
| CDS | contig00012 | 6 | 1328 | 3 | + | 1323 | ADA regulatory protein / Methylated-DNA--protein-cysteine methyltransferase (EC 2.1.1.63) |
| CDS | contig00012 | 1325 | 1801 | 2 | + | 477 | Methylated-DNA--protein-cysteine methyltransferase (EC 2.1.1.63) |
| CDS | contig00012 | 2489 | 1824 | -2 | - | 666 | SAM-dependent methyltransferases |
| CDS | contig00012 | 2858 | 2601 | -2 | - | 258 | hypothetical protein |
| CDS | contig00012 | 3232 | 2903 | -1 | - | 330 | FIG00361972: hypothetical protein |
| CDS | contig00012 | 5212 | 3350 | -1 | - | 1863 | Probable collagenase (EC 3.4.24.3) |
| CDS | contig00012 | 6001 | 5390 | -1 | - | 612 | Lysine exporter protein (LYSE/YGGA) precursor |
| CDS | contig00012 | 7083 | 6130 | -3 | - | 954 | Transcriptional regulator, LysR family |
| CDS | contig00012 | 7078 | 7287 | 1 | + | 210 | hypothetical protein |
| CDS | contig00012 | 7284 | 8372 | 3 | + | 1089 | 3-oxoacyl-[acyl-carrier-protein] synthase, KASIII (EC 2.3.1.180) |
| CDS | contig00012 | 9058 | 9330 | 1 | + | 273 | hypothetical protein |
| CDS | contig00012 | 9422 | 10174 | 2 | + | 753 | Sensory box/GGDEF family protein |
| CDS | contig00012 | 10297 | 11562 | 1 | + | 1266 | Succinyl-CoA synthetase, alpha subunit |
| CDS | contig00012 | 14726 | 11619 | -2 | - | 3108 | Tetrathionate reductase subunit A |
| CDS | contig00012 | 15832 | 14726 | -1 | - | 1107 | Tetrathionate reductase subunit C |
| CDS | contig00012 | 16610 | 15822 | -2 | - | 789 | Tetrathionate reductase subunit B |
| CDS | contig00012 | 16869 | 18776 | 3 | + | 1908 | Tetrathionate reductase sensory transduction histidine kinase |
| CDS | contig00012 | 18778 | 19389 | 1 | + | 612 | Tetrathionate reductase two-component response regulator |
| CDS | contig00012 | 19510 | 20238 | 1 | + | 729 | Alpha-aspartyl dipeptidase Peptidase E (EC 3.4.13.21) |
| CDS | contig00012 | 21843 | 20305 | -3 | - | 1539 | Multidrug resistance protein B |
| CDS | contig00012 | 22912 | 21845 | -1 | - | 1068 | Membrane fusion component of tripartite multidrug resistance system |
| CDS | contig00012 | 23378 | 22950 | -2 | - | 429 | Transcriptional regulator, MarR family |
| CDS | contig00012 | 23501 | 24892 | 2 | + | 1392 | Fumarate hydratase class II (EC 4.2.1.2) |
| CDS | contig00012 | 25461 | 25024 | -3 | - | 438 | PhnB protein; putative DNA binding 3-demethylubiquinone-9 3-methyltransferase domain protein |
| CDS | contig00012 | 25860 | 29807 | 3 | + | 3948 | Transcriptional repressor of PutA and PutP / Proline dehydrogenase (EC 1.5.99.8) (Proline oxidase) / Delta-1-pyrroline-5-carboxylate dehydrogenase (EC 1.5.1.12) |
| CDS | contig00012 | 31038 | 29965 | -3 | - | 1074 | Alanine racemase (EC 5.1.1.1) |
| CDS | contig00012 | 32567 | 31173 | -2 | - | 1395 | Replicative DNA helicase (EC 3.6.1.-) |
| CDS | contig00012 | 32988 | 33842 | 3 | + | 855 | FIG00361548: hypothetical protein |
| CDS | contig00012 | 35706 | 34036 | -3 | - | 1671 | UDP-sugar hydrolase (EC 3.6.1.45); 5&#39;-nucleotidase (EC 3.1.3.5) |
| CDS | contig00012 | 35955 | 36293 | 3 | + | 339 | FIG00922492: hypothetical protein |
| CDS | contig00012 | 36409 | 36744 | 1 | + | 336 | FIG00361615: hypothetical protein |
| CDS | contig00012 | 36843 | 37709 | 3 | + | 867 | FIG00361583: hypothetical protein |
| CDS | contig00012 | 39182 | 37779 | -2 | - | 1404 | Putative LuxO repressor protein |
| CDS | contig00012 | 40140 | 39331 | -3 | - | 810 | FKBP-type peptidyl-prolyl cis-trans isomerase FkpA precursor (EC 5.2.1.8) |
| CDS | contig00012 | 40260 | 41228 | 3 | + | 969 | FOG: WD40 repeat |
| CDS | contig00012 | 41243 | 41458 | 2 | + | 216 | Protein SlyX |
| CDS | contig00012 | 42161 | 41523 | -2 | - | 639 | FKBP-type peptidyl-prolyl cis-trans isomerase SlyD (EC 5.2.1.8) |
| CDS | contig00012 | 42514 | 42308 | -1 | - | 207 | Putative cytoplasmic protein ,probably associated with Glutathione-regulated potassium-efflux |
| CDS | contig00012 | 42554 | 43240 | 2 | + | 687 | hypothetical protein |
| CDS | contig00012 | 43353 | 43219 | -3 | - | 135 | hypothetical protein |
| CDS | contig00012 | 43379 | 44278 | 2 | + | 900 | Chemotaxis protein CheV (EC 2.7.3.-) |
| CDS | contig00012 | 44339 | 45322 | 2 | + | 984 | Hydrolase, alpha/beta fold family functionally coupled to Phosphoribulokinase |
| CDS | contig00012 | 46452 | 45304 | -3 | - | 1149 | diguanylate cyclase/phosphodiesterase (GGDEF & EAL domains) with PAS/PAC sensor(s) |
| CDS | contig00012 | 46542 | 46787 | 3 | + | 246 | hypothetical protein |
| CDS | contig00012 | 47023 | 47892 | 1 | + | 870 | Phosphoribulokinase (EC 2.7.1.19) homolog, function unknown |
| CDS | contig00012 | 47909 | 48442 | 2 | + | 534 | hypothetical protein |
| CDS | contig00012 | 48811 | 48512 | -1 | - | 300 | FIG00545237: hypothetical protein |
| CDS | contig00012 | 49264 | 48860 | -1 | - | 405 | OsmC/Ohr family protein |
| CDS | contig00012 | 49639 | 50277 | 1 | + | 639 | Cyclic AMP receptor protein |
| CDS | contig00012 | 50330 | 51205 | 2 | + | 876 | Ribosomal RNA large subunit methyltransferase A (EC 2.1.1.51) |
| CDS | contig00012 | 53943 | 51346 | -3 | - | 2598 | Chitinase (EC 3.2.1.14) |
| CDS | contig00012 | 57804 | 54343 | -3 | - | 3462 | Sensor histidine kinase |
| CDS | contig00012 | 59010 | 57868 | -3 | - | 1143 | Probable 3-phenylpropionic acid transporter |
| CDS | contig00012 | 61365 | 59272 | -3 | - | 2094 | TonB-dependent heme receptor HutR |
| CDS | contig00012 | 61562 | 62530 | 2 | + | 969 | Uncharacterized iron-regulated protein |
| CDS | contig00012 | 62545 | 63078 | 1 | + | 534 | Isochorismatase (EC 3.3.2.1) |
| CDS | contig00012 | 63136 | 63543 | 1 | + | 408 | FIG00362141: hypothetical protein |
| CDS | contig00012 | 64165 | 63608 | -1 | - | 558 | Pyridoxamine 5&#39;-phosphate oxidase-related putative heme iron utilization protein |
| CDS | contig00012 | 64674 | 64162 | -3 | - | 513 | Putative heme iron utilization protein |
| CDS | contig00012 | 64842 | 65687 | 3 | + | 846 | Periplasmic hemin-binding protein |
| CDS | contig00012 | 65687 | 66715 | 2 | + | 1029 | Hemin ABC transporter, permease protein |
| CDS | contig00012 | 66731 | 67522 | 2 | + | 792 | ABC-type hemin transport system, ATPase component |
| CDS | contig00012 | 68227 | 67619 | -1 | - | 609 | Arginine/ornithine antiporter ArcD |
| CDS | contig00012 | 68520 | 69869 | 3 | + | 1350 | UDP-glucose dehydrogenase (EC 1.1.1.22) |
| CDS | contig00012 | 69866 | 70864 | 2 | + | 999 | dTDP-glucose 4,6-dehydratase (EC 4.2.1.46) |
| CDS | contig00012 | 72594 | 70906 | -3 | - | 1689 | Polymyxin resistance protein ArnT, undecaprenyl phosphate-alpha-L-Ara4N transferase; Melittin resistance protein PqaB |
| CDS | contig00012 | 72946 | 72584 | -1 | - | 363 | FIG01217776: hypothetical protein |
| CDS | contig00012 | 73683 | 72943 | -3 | - | 741 | Dolichol-phosphate mannosyltransferase |
| CDS | contig00012 | 73996 | 74838 | 1 | + | 843 | Membrane-bound lytic murein transglycosylase D precursor (EC 3.2.1.-) |
| CDS | contig00012 | 75037 | 76704 | 1 | + | 1668 | ABC transporter, ATP-binding protein |
| CDS | contig00012 | 76816 | 77145 | 1 | + | 330 | hypothetical protein |
| CDS | contig00012 | 77347 | 78756 | 1 | + | 1410 | Xanthine/uracil/thiamine/ascorbate permease family protein |
| CDS | contig00012 | 79664 | 78840 | -2 | - | 825 | DnaJ-like protein DjlA |
| CDS | contig00012 | 80352 | 79684 | -3 | - | 669 | Glucose-1-phosphate thymidylyltransferase (EC 2.7.7.24) |
| CDS | contig00012 | 81359 | 80349 | -2 | - | 1011 | COG3178: Predicted phosphotransferase related to Ser/Thr protein kinases |
| CDS | contig00012 | 81483 | 83966 | 3 | + | 2484 | Outer membrane protein Imp, required for envelope biogenesis / Organic solvent tolerance protein precursor |
| CDS | contig00012 | 83991 | 85289 | 3 | + | 1299 | Survival protein SurA precursor (Peptidyl-prolyl cis-trans isomerase SurA) (EC 5.2.1.8) |
| CDS | contig00012 | 85289 | 86335 | 2 | + | 1047 | 4-hydroxythreonine-4-phosphate dehydrogenase (EC 1.1.1.262) |
| CDS | contig00012 | 86343 | 87137 | 3 | + | 795 | SSU rRNA (adenine(1518)-N(6)/adenine(1519)-N(6))-dimethyltransferase (EC 2.1.1.182) |
| CDS | contig00012 | 87142 | 87504 | 1 | + | 363 | ApaG protein |
| CDS | contig00012 | 87511 | 88332 | 1 | + | 822 | Bis(5&#39;-nucleosyl)-tetraphosphatase, symmetrical (EC 3.6.1.41) |
| CDS | contig00012 | 88944 | 88459 | -3 | - | 486 | FIG00361726: hypothetical protein |
| CDS | contig00012 | 90118 | 89129 | -1 | - | 990 | FIG00361586: hypothetical protein |
| CDS | contig00012 | 90335 | 93220 | 2 | + | 2886 | Chemotactic transducer-related protein |
| CDS | contig00012 | 94661 | 93291 | -2 | - | 1371 | Predicted arginine uptake transporter |
| CDS | contig00012 | 96230 | 95028 | -2 | - | 1203 | GTP-binding protein Obg |
| CDS | contig00012 | 96677 | 96420 | -2 | - | 258 | LSU ribosomal protein L27p |
| CDS | contig00012 | 97006 | 96695 | -1 | - | 312 | LSU ribosomal protein L21p |
| CDS | contig00012 | 97249 | 98220 | 1 | + | 972 | Octaprenyl diphosphate synthase (EC 2.5.1.90) / Dimethylallyltransferase (EC 2.5.1.1) / (2E,6E)-farnesyl diphosphate synthase (EC 2.5.1.10) / Geranylgeranyl pyrophosphate synthetase (EC 2.5.1.29) |
| CDS | contig00012 | 98497 | 98309 | -1 | - | 189 | Putative inner membrane protein YjeT (clustered with HflC) |
| CDS | contig00012 | 99473 | 98589 | -2 | - | 885 | HflC protein |
| CDS | contig00012 | 100628 | 99477 | -2 | - | 1152 | HflK protein |
| CDS | contig00012 | 102007 | 100721 | -1 | - | 1287 | GTP-binding protein HflX |
| CDS | contig00012 | 102334 | 102071 | -1 | - | 264 | RNA-binding protein Hfq |
| CDS | contig00012 | 103333 | 102407 | -1 | - | 927 | tRNA dimethylallyltransferase (EC 2.5.1.75) |
| CDS | contig00012 | 105362 | 103422 | -2 | - | 1941 | DNA mismatch repair protein MutL |
| CDS | contig00012 | 106962 | 105418 | -3 | - | 1545 | N-acetylmuramoyl-L-alanine amidase (EC 3.5.1.28) |
| CDS | contig00012 | 107432 | 106959 | -2 | - | 474 | TsaE protein, required for threonylcarbamoyladenosine t(6)A37 formation in tRNA |
| CDS | contig00012 | 108964 | 107450 | -1 | - | 1515 | NAD(P)HX epimerase / NAD(P)HX dehydratase |
| CDS | contig00012 | 109052 | 110206 | 2 | + | 1155 | Epoxyqueuosine (oQ) reductase QueG |
| CDS | contig00012 | 110290 | 111033 | 1 | + | 744 | Polyphosphate glucokinase (EC 2.7.1.63) |
| CDS | contig00012 | 111541 | 111086 | -1 | - | 456 | hypothetical protein |
| CDS | contig00012 | 112023 | 113057 | 3 | + | 1035 | Methionine ABC transporter ATP-binding protein |
| CDS | contig00012 | 113038 | 113691 | 1 | + | 654 | Methionine ABC transporter permease protein |
| CDS | contig00012 | 113718 | 114518 | 3 | + | 801 | Methionine ABC transporter substrate-binding protein |
| CDS | contig00012 | 114616 | 115116 | 1 | + | 501 | Transcriptional activator RfaH |
| CDS | contig00012 | 117260 | 115284 | -2 | - | 1977 | Methyl-accepting chemotaxis protein |
| CDS | contig00012 | 118479 | 117298 | -3 | - | 1182 | regulatory protein for cyclic-di-GMP, EAL domain |
| CDS | contig00012 | 122776 | 118502 | -1 | - | 4275 | sensor histidine kinase |
| CDS | contig00012 | 123124 | 122921 | -1 | - | 204 | FIG00361219: hypothetical protein |
| CDS | contig00012 | 124794 | 123418 | -3 | - | 1377 | Deoxyguanosinetriphosphate triphosphohydrolase (EC 3.1.5.1) |
| CDS | contig00012 | 127166 | 124824 | -2 | - | 2343 | Aerobic respiration control sensor protein arcB (EC 2.7.3.-) |
| CDS | contig00012 | 127339 | 128337 | 1 | + | 999 | FIG00361673: hypothetical protein |
| CDS | contig00012 | 129091 | 128399 | -1 | - | 693 | FIG00361685: hypothetical protein |
| CDS | contig00012 | 129695 | 129210 | -2 | - | 486 | Histidine triad family protein |
| CDS | contig00012 | 130353 | 129730 | -3 | - | 624 | putative secreted protein |
| CDS | contig00012 | 131333 | 130428 | -2 | - | 906 | Transcriptional regulator, LysR family |
| CDS | contig00012 | 131428 | 132030 | 1 | + | 603 | Short chain dehydrogenase |
| CDS | contig00012 | 133257 | 132154 | -3 | - | 1104 | Phosphoribosylaminoimidazole-succinocarboxamide synthase (EC 6.3.2.6) |
| CDS | contig00012 | 134545 | 133427 | -1 | - | 1119 | Outer membrane protein NlpB, lipoprotein component of the protein assembly complex (forms a complex with YaeT, YfiO, and YfgL); Lipoprotein-34 precursor |
| CDS | contig00012 | 135425 | 134538 | -2 | - | 888 | 4-hydroxy-tetrahydrodipicolinate synthase (EC 4.3.3.7) |
| CDS | contig00012 | 135614 | 136147 | 2 | + | 534 | Glycine cleavage system transcriptional antiactivator GcvR |
| CDS | contig00012 | 136171 | 136635 | 1 | + | 465 | Thiol peroxidase, Bcp-type (EC 1.11.1.15) |
| CDS | contig00012 | 136831 | 137331 | 1 | + | 501 | FIG00361523: hypothetical protein |
| CDS | contig00012 | 137722 | 137384 | -1 | - | 339 | FIG00638298: membrane protein YfbV |
| CDS | contig00012 | 138126 | 139328 | 3 | + | 1203 | Acetate kinase (EC 2.7.2.1) |
| CDS | contig00012 | 139488 | 139330 | -3 | - | 159 | hypothetical protein |
| CDS | contig00012 | 139477 | 141552 | 1 | + | 2076 | Phosphate acetyltransferase (EC 2.3.1.8) |
| CDS | contig00012 | 142551 | 142030 | -3 | - | 522 | Acetolactate synthase small subunit (EC 2.2.1.6) |
| CDS | contig00012 | 144242 | 142524 | -2 | - | 1719 | Acetolactate synthase large subunit (EC 2.2.1.6) |
| CDS | contig00012 | 144785 | 144633 | -2 | - | 153 | hypothetical protein |
| CDS | contig00012 | 145930 | 144821 | -1 | - | 1110 | Glycosyltransferase (EC 2.4.1.-) |
| CDS | contig00012 | 146430 | 145927 | -3 | - | 504 | Membrane-associated phospholipid phosphatase |
| CDS | contig00012 | 147179 | 146556 | -2 | - | 624 | Transcriptional regulator, TetR family |
| CDS | contig00012 | 147585 | 149147 | 3 | + | 1563 | 2-isopropylmalate synthase (EC 2.3.3.13) |
| CDS | contig00012 | 149259 | 150344 | 3 | + | 1086 | 3-isopropylmalate dehydrogenase (EC 1.1.1.85) |
| CDS | contig00012 | 150377 | 151774 | 2 | + | 1398 | 3-isopropylmalate dehydratase large subunit (EC 4.2.1.33) |
| CDS | contig00012 | 151787 | 152386 | 2 | + | 600 | 3-isopropylmalate dehydratase small subunit (EC 4.2.1.33) |
| CDS | contig00012 | 152499 | 153428 | 3 | + | 930 | uncharacterized secreted protein |
| CDS | contig00012 | 153770 | 153480 | -2 | - | 291 | FIG00361573: hypothetical protein |
| CDS | contig00012 | 154036 | 155016 | 1 | + | 981 | Thiamin ABC transporter, substrate-binding component |
| CDS | contig00012 | 154992 | 156620 | 3 | + | 1629 | Thiamin ABC transporter, transmembrane component |
| CDS | contig00012 | 156762 | 157472 | 3 | + | 711 | Thiamin ABC transporter, ATPase component |
| CDS | contig00012 | 157488 | 157649 | 3 | + | 162 | Thiamin ABC transporter, ATPase component |
| CDS | contig00012 | 159086 | 157716 | -2 | - | 1371 | Predicted ATPase related to phosphate starvation-inducible protein PhoH |
| CDS | contig00012 | 159975 | 159292 | -3 | - | 684 | Ribonucleotide reductase of class II (coenzyme B12-dependent), alpha subunit (EC 1.17.4.1) |
| CDS | contig00012 | 162132 | 159985 | -3 | - | 2148 | Ribonucleotide reductase of class II (coenzyme B12-dependent) (EC 1.17.4.1) |
| CDS | contig00012 | 162771 | 162340 | -3 | - | 432 | hypothetical protein |
| CDS | contig00013 | 1604 | 39 | -2 | - | 1566 | ATPase |
| CDS | contig00013 | 2006 | 2386 | 2 | + | 381 | hypothetical protein |
| CDS | contig00013 | 4571 | 2547 | -2 | - | 2025 | High-affinity choline uptake protein BetT |
| CDS | contig00013 | 4581 | 4865 | 3 | + | 285 | hypothetical protein |
| CDS | contig00013 | 4870 | 5133 | 1 | + | 264 | Putative signal peptide protein |
| CDS | contig00013 | 5193 | 6035 | 3 | + | 843 | Uncharacterized protein conserved in bacteria, NMA0228-like |
| CDS | contig00013 | 6089 | 6841 | 2 | + | 753 | Conserved domain protein |
| CDS | contig00013 | 6838 | 7293 | 1 | + | 456 | INTEGRAL MEMBRANE PROTEIN (Rhomboid family) |
| CDS | contig00013 | 7354 | 7818 | 1 | + | 465 | Histone acetyltransferase HPA2 and related acetyltransferases |
| CDS | contig00013 | 8019 | 9314 | 3 | + | 1296 | Methyl-accepting chemotaxis protein |
| CDS | contig00013 | 9459 | 10016 | 3 | + | 558 | FIG01057338: hypothetical protein |
| CDS | contig00013 | 10101 | 11474 | 3 | + | 1374 | Response regulator |
| CDS | contig00013 | 12623 | 11544 | -2 | - | 1080 | Putative inner membrane protein |
| CDS | contig00013 | 13454 | 12705 | -2 | - | 750 | Molybdopterin-guanine dinucleotide biosynthesis protein MobB |
| CDS | contig00013 | 14065 | 13451 | -1 | - | 615 | Molybdopterin-guanine dinucleotide biosynthesis protein MobA |
| CDS | contig00013 | 14287 | 15996 | 1 | + | 1710 | NAD-dependent malic enzyme (EC 1.1.1.38) |
| CDS | contig00013 | 17340 | 16114 | -3 | - | 1227 | LPXTG-motif cell wall anchor domain protein |
| CDS | contig00013 | 17658 | 18896 | 3 | + | 1239 | Uracil permease |
| CDS | contig00013 | 19042 | 19782 | 1 | + | 741 | Dienelactone hydrolase family |
| CDS | contig00013 | 20844 | 19846 | -3 | - | 999 | 6-phosphogluconolactonase (EC 3.1.1.31) |
| CDS | contig00013 | 21510 | 20902 | -3 | - | 609 | Transporter, LysE family |
| CDS | contig00013 | 24948 | 21631 | -3 | - | 3318 | Potassium efflux system KefA protein / Small-conductance mechanosensitive channel |
| CDS | contig00013 | 26522 | 25098 | -2 | - | 1425 | Pyruvate kinase (EC 2.7.1.40) |
| CDS | contig00013 | 28045 | 26915 | -1 | - | 1131 | FIG002776: hypothetical protein |
| CDS | contig00013 | 29568 | 28198 | -3 | - | 1371 | Adenylosuccinate lyase (EC 4.3.2.2) |
| CDS | contig00013 | 30252 | 29599 | -3 | - | 654 | FIG002903: a protein of unknown function perhaps involved in purine metabolism |
| CDS | contig00013 | 31664 | 30558 | -2 | - | 1107 | tRNA-specific 2-thiouridylase MnmA |
| CDS | contig00013 | 32435 | 31968 | -2 | - | 468 | Nudix-like NDP and NTP phosphohydrolase YmfB |
| CDS | contig00013 | 33336 | 32500 | -3 | - | 837 | Ribosomal large subunit pseudouridine synthase E (EC 4.2.1.70) |
| CDS | contig00013 | 33958 | 33491 | -1 | - | 468 | acetyltransferase, GNAT family |
| CDS | contig00013 | 34149 | 33955 | -3 | - | 195 | hypothetical protein |
| CDS | contig00013 | 34918 | 34157 | -1 | - | 762 | hypothetical protein |
| CDS | contig00013 | 37042 | 34922 | -1 | - | 2121 | hypothetical protein |
| CDS | contig00013 | 37025 | 37156 | 2 | + | 132 | hypothetical protein |
| CDS | contig00013 | 37582 | 37289 | -1 | - | 294 | hypothetical protein |
| CDS | contig00013 | 38141 | 38290 | 2 | + | 150 | hypothetical protein |
| CDS | contig00013 | 39466 | 38375 | -1 | - | 1092 | hypothetical protein |
| CDS | contig00013 | 39877 | 41130 | 1 | + | 1254 | Isocitrate dehydrogenase [NADP] (EC 1.1.1.42) |
| CDS | contig00013 | 41373 | 41197 | -3 | - | 177 | hypothetical protein |
| CDS | contig00013 | 43044 | 41713 | -3 | - | 1332 | D-serine dehydratase (EC 4.3.1.18) |
| CDS | contig00013 | 43262 | 44206 | 2 | + | 945 | D-serine dehydratase transcriptional activator |
| CDS | contig00013 | 44556 | 44963 | 3 | + | 408 | DNA-binding protein H-NS |
| CDS | contig00013 | 46540 | 45434 | -1 | - | 1107 | 2-keto-3-deoxy-D-arabino-heptulosonate-7-phosphate synthase I alpha (EC 2.5.1.54) |
| CDS | contig00013 | 47596 | 46790 | -1 | - | 807 | iron-chelator utilization protein |
| CDS | contig00013 | 49096 | 47729 | -1 | - | 1368 | RND efflux system, outer membrane lipoprotein CmeC |
| CDS | contig00013 | 51123 | 49099 | -3 | - | 2025 | Macrolide export ATP-binding/permease protein MacB (EC 3.6.3.-) |
| CDS | contig00013 | 52283 | 51120 | -2 | - | 1164 | Macrolide-specific efflux protein MacA |
| CDS | contig00013 | 52918 | 52478 | -1 | - | 441 | FIG00361518: hypothetical protein |
| CDS | contig00013 | 55054 | 53390 | -1 | - | 1665 | DNA repair protein RecN |
| CDS | contig00013 | 55088 | 55240 | 2 | + | 153 | hypothetical protein |
| CDS | contig00013 | 55361 | 56518 | 2 | + | 1158 | diguanylate cyclase/phosphodiesterase (GGDEF & EAL domains) with PAS/PAC sensor(s) |
| CDS | contig00013 | 57427 | 56543 | -1 | - | 885 | NAD kinase (EC 2.7.1.23) |
| CDS | contig00013 | 57691 | 58266 | 1 | + | 576 | Heat shock protein GrpE |
| CDS | contig00013 | 58550 | 60478 | 2 | + | 1929 | Chaperone protein DnaK |
| CDS | contig00013 | 60666 | 60529 | -3 | - | 138 | Uroporphyrinogen-III methyltransferase (EC 2.1.1.107) |
| CDS | contig00013 | 60733 | 61869 | 1 | + | 1137 | Chaperone protein DnaJ |
| CDS | contig00013 | 62140 | 63624 | 1 | + | 1485 | Maltoporin (maltose/maltodextrin high-affinity receptor, phage lambda receptor protein) |
| CDS | contig00013 | 63772 | 64695 | 1 | + | 924 | Fructokinase (EC 2.7.1.4) |
| CDS | contig00013 | 64779 | 66143 | 3 | + | 1365 | PTS system, sucrose-specific IIB component (EC 2.7.1.69) / PTS system, sucrose-specific IIC component (EC 2.7.1.69) |
| CDS | contig00013 | 66143 | 67558 | 2 | + | 1416 | Sucrose-6-phosphate hydrolase (EC 3.2.1.B3) |
| CDS | contig00013 | 67586 | 68596 | 2 | + | 1011 | Sucrose operon repressor ScrR, LacI family |
| CDS | contig00013 | 69402 | 68674 | -3 | - | 729 | Mg(2+) transport ATPase protein C |
| CDS | contig00013 | 69958 | 69584 | -1 | - | 375 | hypothetical protein |
| CDS | contig00013 | 71601 | 70330 | -3 | - | 1272 | FAD-dependent pyridine nucleotide-disulphide oxidoreductase |
| CDS | contig00013 | 71839 | 71654 | -1 | - | 186 | Rhodanese-related sulfurtransferase |
| CDS | contig00013 | 71925 | 72239 | 3 | + | 315 | Transcriptional regulator, ArsR family |
| CDS | contig00013 | 72236 | 72649 | 2 | + | 414 | Probable transmembrane protein |
| CDS | contig00013 | 72663 | 73109 | 3 | + | 447 | Predicted transporter component |
| CDS | contig00013 | 73146 | 74168 | 3 | + | 1023 | Efflux transporter, RND family, MFP subunit, AcrA/E family |
| CDS | contig00013 | 74161 | 77319 | 1 | + | 3159 | Acriflavin resistance protein |
| CDS | contig00013 | 79797 | 77353 | -3 | - | 2445 | diguanylate cyclase/phosphodiesterase (GGDEF & EAL domains) with PAS/PAC sensor(s) |
| CDS | contig00013 | 80208 | 81221 | 3 | + | 1014 | Lipoate-protein ligase A |
| CDS | contig00013 | 81407 | 83092 | 2 | + | 1686 | Glutamate decarboxylase |
| CDS | contig00013 | 83543 | 83166 | -2 | - | 378 | hypothetical protein |
| CDS | contig00013 | 86712 | 83590 | -3 | - | 3123 | Cobalt-zinc-cadmium resistance protein CzcA; Cation efflux system protein CusA |
| CDS | contig00013 | 88318 | 86786 | -1 | - | 1533 | Cobalt/zinc/cadmium efflux RND transporter, membrane fusion protein, CzcB family |
| CDS | contig00013 | 89111 | 88620 | -2 | - | 492 | hypothetical protein |
| CDS | contig00013 | 90101 | 89295 | -2 | - | 807 | Exodeoxyribonuclease III (EC 3.1.11.2) |
| CDS | contig00013 | 91316 | 90354 | -2 | - | 963 | Kef-type K+ transport system, predicted NAD-binding component |
| CDS | contig00013 | 93021 | 91375 | -3 | - | 1647 | Response regulator with TPR repeat |
| CDS | contig00013 | 93292 | 93717 | 1 | + | 426 | Small heat shock protein |
| CDS | contig00013 | 94802 | 93759 | -2 | - | 1044 | Thiamin biosynthesis lipoprotein ApbE |
| CDS | contig00013 | 95102 | 95542 | 2 | + | 441 | Azurin |
| CDS | contig00013 | 95724 | 95927 | 3 | + | 204 | putative secreted protein |
| CDS | contig00013 | 96493 | 95924 | -1 | - | 570 | Conserved hypothetical protein (perhaps related to histidine degradation) |
| CDS | contig00013 | 97045 | 96623 | -1 | - | 423 | Transcriptional regulator, MarR family |
| CDS | contig00013 | 97204 | 97085 | -1 | - | 120 | hypothetical protein |
| CDS | contig00013 | 97181 | 97426 | 2 | + | 246 | FIG00905263: hypothetical protein |
| CDS | contig00013 | 97559 | 98530 | 2 | + | 972 | Inosine-uridine preferring nucleoside hydrolase (EC 3.2.2.1) |
| CDS | contig00013 | 98626 | 98955 | 1 | + | 330 | FIG00362388: hypothetical protein |
| CDS | contig00013 | 99954 | 99046 | -3 | - | 909 | Transcriptional regulator, LysR family |
| CDS | contig00013 | 100074 | 100517 | 3 | + | 444 | 4-carboxymuconolactone decarboxylase domain/alkylhydroperoxidase AhpD family core domain protein |
| CDS | contig00013 | 101025 | 102113 | 3 | + | 1089 | Cytochrome O ubiquinol oxidase subunit II (EC 1.10.3.-) |
| CDS | contig00013 | 102117 | 104093 | 3 | + | 1977 | Cytochrome O ubiquinol oxidase subunit I (EC 1.10.3.-) |
| CDS | contig00013 | 104098 | 104709 | 1 | + | 612 | Cytochrome O ubiquinol oxidase subunit III (EC 1.10.3.-) |
| CDS | contig00013 | 104710 | 105039 | 1 | + | 330 | Cytochrome O ubiquinol oxidase subunit IV (EC 1.10.3.-) |
| CDS | contig00013 | 105048 | 105941 | 3 | + | 894 | Heme O synthase, protoheme IX farnesyltransferase (EC 2.5.1.-) COX10-CtaB |
| CDS | contig00013 | 106106 | 107467 | 2 | + | 1362 | Putative transport protein |
| CDS | contig00013 | 107541 | 107909 | 3 | + | 369 | Protein of unknown function DUF1428 |
| CDS | contig00013 | 108903 | 108052 | -3 | - | 852 | Alpha-ketoglutarate-dependent taurine dioxygenase (EC 1.14.11.17) |
| CDS | contig00013 | 109813 | 108983 | -1 | - | 831 | Taurine transport system permease protein TauC |
| CDS | contig00013 | 110577 | 109810 | -3 | - | 768 | Taurine transport ATP-binding protein TauB |
| CDS | contig00013 | 111568 | 110594 | -1 | - | 975 | Taurine-binding periplasmic protein TauA |
| CDS | contig00013 | 112963 | 111857 | -1 | - | 1107 | Uncharacterized conserved protein |
| CDS | contig00013 | 114036 | 113110 | -3 | - | 927 | Transcriptional regulator, LysR family |
| CDS | contig00013 | 114104 | 114592 | 2 | + | 489 | FIG073159: hypothetical protein |
| CDS | contig00013 | 114691 | 115125 | 1 | + | 435 | FIG123062: hypothetical protein |
| CDS | contig00013 | 115640 | 115398 | -2 | - | 243 | hypothetical protein |
| CDS | contig00013 | 115922 | 115743 | -2 | - | 180 | hypothetical protein |
| CDS | contig00013 | 116907 | 116545 | -3 | - | 363 | hypothetical protein |
| CDS | contig00013 | 117821 | 117693 | -2 | - | 129 | hypothetical protein |
| CDS | contig00013 | 119731 | 119907 | 1 | + | 177 | hypothetical protein |
| CDS | contig00013 | 122079 | 121273 | -3 | - | 807 | Tryptophan synthase alpha chain (EC 4.2.1.20) |
| CDS | contig00013 | 123269 | 122076 | -2 | - | 1194 | Tryptophan synthase beta chain (EC 4.2.1.20) |
| CDS | contig00013 | 124879 | 123458 | -1 | - | 1422 | Indole-3-glycerol phosphate synthase (EC 4.1.1.48) / Phosphoribosylanthranilate isomerase (EC 5.3.1.24) |
| CDS | contig00013 | 125976 | 124963 | -3 | - | 1014 | Anthranilate phosphoribosyltransferase (EC 2.4.2.18) |
| CDS | contig00013 | 126586 | 125987 | -1 | - | 600 | Anthranilate synthase, amidotransferase component (EC 4.1.3.27) |
| CDS | contig00013 | 128301 | 126667 | -3 | - | 1635 | Anthranilate synthase, aminase component (EC 4.1.3.27) |
| CDS | contig00013 | 128317 | 128631 | 1 | + | 315 | hypothetical protein |
| CDS | contig00013 | 128726 | 129607 | 2 | + | 882 | COG0613, Predicted metal-dependent phosphoesterases (PHP family) |
| CDS | contig00013 | 129734 | 130354 | 2 | + | 621 | Hypothetical YciO protein, TsaC/YrdC paralog |
| CDS | contig00013 | 130434 | 131228 | 3 | + | 795 | Segregation and condensation protein A |
| CDS | contig00013 | 131270 | 131962 | 2 | + | 693 | Segregation and condensation protein B |
| CDS | contig00013 | 131955 | 132878 | 3 | + | 924 | Ribosomal large subunit pseudouridine synthase B (EC 4.2.1.70) |
| CDS | contig00013 | 133284 | 133463 | 3 | + | 180 | DNA methylase, putative |
| CDS | contig00013 | 133645 | 134508 | 1 | + | 864 | Acyl-CoA thioesterase II (EC 3.1.2.-) |
| CDS | contig00013 | 135747 | 134572 | -3 | - | 1176 | Proton/glutamate symport protein @ Sodium/glutamate symport protein |
| CDS | contig00013 | 136428 | 135916 | -3 | - | 513 | Tellurite resistance protein-related protein |
| CDS | contig00013 | 137206 | 136571 | -1 | - | 636 | Transcription repressor of multidrug efflux pump acrAB operon, TetR (AcrR) family |
| CDS | contig00013 | 137361 | 138542 | 3 | + | 1182 | Membrane fusion protein of RND family multidrug efflux pump |
| CDS | contig00013 | 138560 | 141709 | 2 | + | 3150 | RND efflux system, inner membrane transporter CmeB |
| CDS | contig00013 | 141702 | 143117 | 3 | + | 1416 | RND efflux system, outer membrane lipoprotein CmeC |
| CDS | contig00013 | 143444 | 144439 | 2 | + | 996 | Sulfate-binding protein Sbp |
| CDS | contig00013 | 145583 | 144843 | -2 | - | 741 | putative histidinol phosphatase and related hydrolases of the PHP family |
| CDS | contig00013 | 146052 | 146705 | 3 | + | 654 | Predicted transcriptional regulator for fatty acid degradation FadQ, TetR family |
| CDS | contig00013 | 146753 | 149077 | 2 | + | 2325 | Butyryl-CoA dehydrogenase (EC 1.3.8.1) |
| CDS | contig00013 | 149239 | 152301 | 1 | + | 3063 | Acriflavin resistance protein |
| CDS | contig00013 | 152317 | 153360 | 1 | + | 1044 | Probable Co/Zn/Cd efflux system membrane fusion protein |
| CDS | contig00013 | 154866 | 153427 | -3 | - | 1440 | Pyruvate kinase (EC 2.7.1.40) |
| CDS | contig00013 | 155884 | 155027 | -1 | - | 858 | Phosphogluconate repressor HexR, RpiR family |
| CDS | contig00013 | 156238 | 156026 | -1 | - | 213 | hypothetical protein |
| CDS | contig00013 | 156230 | 157198 | 2 | + | 969 | Glucokinase (EC 2.7.1.2) |
| CDS | contig00013 | 158950 | 157568 | -1 | - | 1383 | Sensor protein PhoQ (EC 2.7.13.3) |
| CDS | contig00013 | 159396 | 158950 | -3 | - | 447 | Transcriptional regulatory protein PhoP |
| CDS | contig00014 | 469 | 80 | -1 | - | 390 | Glycoprotein/polysaccharide metabolism |
| CDS | contig00014 | 636 | 1112 | 3 | + | 477 | hypothetical protein |
| CDS | contig00014 | 1595 | 1116 | -2 | - | 480 | Ribonuclease E inhibitor RraA |
| CDS | contig00014 | 1927 | 1703 | -1 | - | 225 | hypothetical protein |
| CDS | contig00014 | 2487 | 3206 | 3 | + | 720 | Aerobic respiration control protein arcA |
| CDS | contig00014 | 3798 | 5573 | 3 | + | 1776 | Transport ATP-binding protein CydD |
| CDS | contig00014 | 5566 | 7311 | 1 | + | 1746 | Transport ATP-binding protein CydC |
| CDS | contig00014 | 7419 | 7790 | 3 | + | 372 | lipoprotein, putative |
| CDS | contig00014 | 7799 | 8185 | 2 | + | 387 | Uncharacterized conserved protein |
| CDS | contig00014 | 9251 | 8256 | -2 | - | 996 | Isoflavone reductase |
| CDS | contig00014 | 9557 | 10261 | 2 | + | 705 | COG1720: Uncharacterized conserved protein |
| CDS | contig00014 | 11454 | 10333 | -3 | - | 1122 | 3-oxoacyl-[acyl-carrier-protein] synthase, KASIII (EC 2.3.1.41) |
| CDS | contig00014 | 12673 | 11510 | -1 | - | 1164 | FIG00362067: hypothetical protein |
| CDS | contig00014 | 13519 | 12677 | -1 | - | 843 | ATPase associated with various cellular activities, AAA_5 |
| CDS | contig00014 | 13683 | 14651 | 3 | + | 969 | Cysteine synthase (EC 2.5.1.47) |
| CDS | contig00014 | 15095 | 15352 | 2 | + | 258 | Phosphocarrier protein of PTS system |
| CDS | contig00014 | 15397 | 17124 | 1 | + | 1728 | Phosphoenolpyruvate-protein phosphotransferase of PTS system (EC 2.7.3.9) |
| CDS | contig00014 | 17168 | 17677 | 2 | + | 510 | PTS system, glucose-specific IIA component |
| CDS | contig00014 | 17933 | 18478 | 2 | + | 546 | Cytochrome b561 |
| CDS | contig00014 | 22315 | 18560 | -1 | - | 3756 | Exonuclease SbcC |
| CDS | contig00014 | 23538 | 22312 | -3 | - | 1227 | Exonuclease SbcD |
| CDS | contig00014 | 24077 | 23649 | -2 | - | 429 | Probable acetyltransferase |
| CDS | contig00014 | 24257 | 25168 | 2 | + | 912 | DNA recombination-dependent growth factor C |
| CDS | contig00014 | 25953 | 25336 | -3 | - | 618 | Arylesterase precursor (EC 3.1.1.2) |
| CDS | contig00014 | 26066 | 27841 | 2 | + | 1776 | Gamma-glutamyltranspeptidase (EC 2.3.2.2) |
| CDS | contig00014 | 28216 | 29847 | 1 | + | 1632 | POTASSIUM/PROTON ANTIPORTER ROSB |
| CDS | contig00014 | 31247 | 29946 | -2 | - | 1302 | Nitrogen regulation protein NR(I) |
| CDS | contig00014 | 31600 | 32178 | 1 | + | 579 | Fap amyloid fibril minor component |
| CDS | contig00014 | 32366 | 33325 | 2 | + | 960 | Fap amyloid fibril major component |
| CDS | contig00014 | 33378 | 34055 | 3 | + | 678 | Fap protein with C39 domain |
| CDS | contig00014 | 34063 | 34722 | 1 | + | 660 | hypothetical protein |
| CDS | contig00014 | 34785 | 35990 | 3 | + | 1206 | Fap amyloid fiber secretin |
| CDS | contig00014 | 37368 | 36091 | -3 | - | 1278 | Similar to gamma-glutamyl-putrescine oxidase |
| CDS | contig00014 | 38026 | 37418 | -1 | - | 609 | Putrescine utilization regulator |
| CDS | contig00014 | 38336 | 39799 | 2 | + | 1464 | Succinate-semialdehyde dehydrogenase [NAD(P)+] (EC 1.2.1.16) |
| CDS | contig00014 | 40032 | 41321 | 3 | + | 1290 | Gamma-aminobutyrate:alpha-ketoglutarate aminotransferase (EC 2.6.1.19) |
| CDS | contig00014 | 41555 | 41692 | 2 | + | 138 | hypothetical protein |
| CDS | contig00014 | 41692 | 43035 | 1 | + | 1344 | Putrescine importer |
| CDS | contig00014 | 43507 | 44850 | 1 | + | 1344 | Putrescine importer |
| CDS | contig00014 | 45321 | 44926 | -3 | - | 396 | hypothetical protein |
| CDS | contig00014 | 45569 | 46723 | 2 | + | 1155 | Sodium-dependent phosphate transporter |
| CDS | contig00014 | 46861 | 47331 | 1 | + | 471 | FKBP-type peptidyl-prolyl cis-trans isomerase FkpA precursor (EC 5.2.1.8) |
| CDS | contig00014 | 47630 | 48397 | 2 | + | 768 | FIG074102: hypothetical protein |
| CDS | contig00014 | 48541 | 51762 | 1 | + | 3222 | Hybrid sensory histidine kinase in two-component regulatory system with EvgA |
| CDS | contig00014 | 52230 | 52030 | -3 | - | 201 | probable exported protein STY0748 |
| CDS | contig00014 | 52445 | 52732 | 2 | + | 288 | hypothetical protein |
| CDS | contig00014 | 53292 | 56687 | 3 | + | 3396 | Chitin catabolic cascade sensor histidine kinase ChiS |
| CDS | contig00014 | 57498 | 58415 | 3 | + | 918 | FIG00362183: hypothetical protein |
| CDS | contig00014 | 58486 | 59421 | 1 | + | 936 | Peptide chain release factor RF-3 |
| CDS | contig00014 | 60210 | 59485 | -3 | - | 726 | Acyl dehydratase |
| CDS | contig00014 | 61394 | 60447 | -2 | - | 948 | Ribosomal RNA large subunit methyltransferase F (EC 2.1.1.51) |
| CDS | contig00014 | 62034 | 61546 | -3 | - | 489 | Lipoprotein NlpC |
| CDS | contig00014 | 62311 | 63033 | 1 | + | 723 | 4&#39;-phosphopantetheinyl transferase (EC 2.7.8.-) |
| CDS | contig00014 | 63030 | 63878 | 3 | + | 849 | FIG000875: Thioredoxin domain-containing protein EC-YbbN |
| CDS | contig00014 | 64261 | 64019 | -1 | - | 243 | outer membrane lipoprotein |
| CDS | contig00014 | 65140 | 64487 | -1 | - | 654 | 3,4-dihydroxy-2-butanone 4-phosphate synthase (EC 4.1.99.12) |
| CDS | contig00014 | 65606 | 66442 | 2 | + | 837 | Formyltetrahydrofolate deformylase (EC 3.5.1.10) |
| CDS | contig00014 | 67769 | 66501 | -2 | - | 1269 | hypothetical protein |
| CDS | contig00014 | 69360 | 67879 | -3 | - | 1482 | Cardiolipin synthetase (EC 2.7.8.-) |
| CDS | contig00014 | 69472 | 69999 | 1 | + | 528 | Putative lipoprotein yceB precursor |
| CDS | contig00014 | 70444 | 70004 | -1 | - | 441 | FIG00362068: hypothetical protein |
| CDS | contig00014 | 70775 | 71527 | 2 | + | 753 | Soluble lytic murein transglycosylase |
| CDS | contig00014 | 72437 | 71751 | -2 | - | 687 | Aquaporin Z |
| CDS | contig00014 | 73259 | 72738 | -2 | - | 522 | Crossover junction endodeoxyribonuclease RuvC (EC 3.1.22.4) |
| CDS | contig00014 | 74401 | 73493 | -1 | - | 909 | Oxidoreductase, Gfo/Idh/MocA family |
| CDS | contig00014 | 74913 | 74686 | -3 | - | 228 | hypothetical protein |
| CDS | contig00014 | 75548 | 77086 | 2 | + | 1539 | Outer membrane protein |
| CDS | contig00014 | 77104 | 78699 | 1 | + | 1596 | hypothetical protein |
| CDS | contig00014 | 78702 | 79535 | 3 | + | 834 | hypothetical protein |
| CDS | contig00014 | 80019 | 79870 | -3 | - | 150 | hypothetical protein |
| CDS | contig00014 | 81102 | 80152 | -3 | - | 951 | Mobile element protein |
| CDS | contig00014 | 81652 | 81867 | 1 | + | 216 | Probable transcription regulator protein of MDR efflux pump cluster |
| CDS | contig00014 | 84372 | 81955 | -3 | - | 2418 | Probable Fe-S oxidoreductase family 2 |
| CDS | contig00014 | 87721 | 84569 | -1 | - | 3153 | RND multidrug efflux transporter; Acriflavin resistance protein |
| CDS | contig00014 | 88761 | 87721 | -3 | - | 1041 | Probable RND efflux membrane fusion protein |
| CDS | contig00014 | 88984 | 90540 | 1 | + | 1557 | diguanylate cyclase/phosphodiesterase (GGDEF & EAL domains) with PAS/PAC sensor(s) |
| CDS | contig00014 | 90575 | 91444 | 2 | + | 870 | UPF0028 protein YchK |
| CDS | contig00014 | 92871 | 91510 | -3 | - | 1362 | Probable sodium-dependent transporter |
| CDS | contig00014 | 93333 | 93067 | -3 | - | 267 | C protein |
| CDS | contig00014 | 93592 | 94179 | 1 | + | 588 | hypothetical protein |
| CDS | contig00014 | 94466 | 94176 | -2 | - | 291 | hypothetical protein |
| CDS | contig00014 | 94485 | 95504 | 3 | + | 1020 | Outer membrane protein A precursor |
| CDS | contig00014 | 95850 | 95737 | -3 | - | 114 | hypothetical protein |
| CDS | contig00014 | 95917 | 96918 | 1 | + | 1002 | Outer membrane protein A precursor |
| CDS | contig00014 | 97138 | 98196 | 1 | + | 1059 | Outer membrane protein A precursor |
| CDS | contig00014 | 99965 | 98271 | -2 | - | 1695 | NAD-dependent malic enzyme (EC 1.1.1.38) |
| CDS | contig00014 | 100878 | 100021 | -3 | - | 858 | AraC-type DNA-binding domain-containing protein |
| CDS | contig00014 | 100999 | 102378 | 1 | + | 1380 | Adhesin |
| CDS | contig00014 | 102530 | 102778 | 2 | + | 249 | hypothetical protein |
| CDS | contig00014 | 103948 | 102809 | -1 | - | 1140 | diguanylate cyclase (GGDEF domain) with PAS/PAC sensor |
| CDS | contig00014 | 104233 | 104114 | -1 | - | 120 | hypothetical protein |
| CDS | contig00014 | 104192 | 105589 | 2 | + | 1398 | Xanthine-uracil permease |
| CDS | contig00014 | 105690 | 106403 | 3 | + | 714 | Lipoate-protein ligase A |
| CDS | contig00014 | 107981 | 106605 | -2 | - | 1377 | Sodium/glycine symporter GlyP |
| CDS | contig00014 | 108699 | 108574 | -3 | - | 126 | hypothetical protein |
| CDS | contig00014 | 108706 | 109854 | 1 | + | 1149 | Ornithine decarboxylase (EC 4.1.1.17) / Arginine decarboxylase (EC 4.1.1.19) |
| CDS | contig00014 | 110888 | 109920 | -2 | - | 969 | Inner membrane protein YrbG, predicted calcium/sodium:proton antiporter |
| CDS | contig00014 | 111483 | 111013 | -3 | - | 471 | Antioxidant, putative |
| CDS | contig00014 | 112108 | 111596 | -1 | - | 513 | Putative Nudix hydrolase YfcD (EC 3.6.-.-) |
| CDS | contig00014 | 112376 | 112519 | 2 | + | 144 | hypothetical protein |
| CDS | contig00014 | 113358 | 112615 | -3 | - | 744 | ABC-type polar amino acid transport system, ATPase component |
| CDS | contig00014 | 114466 | 113372 | -1 | - | 1095 | Glutamate Aspartate transport system permease protein GltK (TC 3.A.1.3.4) |
| CDS | contig00014 | 115663 | 114476 | -1 | - | 1188 | Glutamate Aspartate transport system permease protein GltJ (TC 3.A.1.3.4) |
| CDS | contig00014 | 116745 | 115726 | -3 | - | 1020 | Glutamate Aspartate periplasmic binding protein precursor GltI (TC 3.A.1.3.4) |
| CDS | contig00014 | 117016 | 116747 | -1 | - | 270 | hypothetical protein |
| CDS | contig00014 | 118026 | 117013 | -3 | - | 1014 | ABC transporter, permease protein |
| CDS | contig00014 | 118987 | 118106 | -1 | - | 882 | ABC transporter, ATP-binding protein |
| CDS | contig00014 | 119736 | 119125 | -3 | - | 612 | FKBP-type peptidyl-prolyl cis-trans isomerase FklB (EC 5.2.1.8) |
| CDS | contig00014 | 120047 | 119928 | -2 | - | 120 | hypothetical protein |
| CDS | contig00014 | 120175 | 120056 | -1 | - | 120 | hypothetical protein |
| CDS | contig00014 | 120303 | 120184 | -3 | - | 120 | hypothetical protein |
| CDS | contig00014 | 121037 | 120312 | -2 | - | 726 | Cytoplasmic copper homeostasis protein CutC |
| CDS | contig00014 | 121981 | 121079 | -1 | - | 903 | N-acetyl-D-glucosamine kinase (EC 2.7.1.59) |
| CDS | contig00014 | 122597 | 122091 | -2 | - | 507 | GCN5-related N-acetyltransferase |
| CDS | contig00014 | 123400 | 122585 | -1 | - | 816 | Zn-dependent protease with chaperone function PA4632 |
| CDS | contig00014 | 124527 | 123553 | -3 | - | 975 | Phosphate ABC transporter, periplasmic phosphate-binding protein PstS (TC 3.A.1.7.1) |
| CDS | contig00014 | 126096 | 124801 | -3 | - | 1296 | Phosphate regulon sensor protein PhoR (SphS) (EC 2.7.13.3) |
| CDS | contig00014 | 126858 | 126169 | -3 | - | 690 | Phosphate regulon transcriptional regulatory protein PhoB (SphR) |
| CDS | contig00014 | 127371 | 126961 | -3 | - | 411 | Glyoxalase family protein |
| CDS | contig00014 | 128726 | 127440 | -2 | - | 1287 | GGDEF family protein |
| CDS | contig00014 | 130706 | 129030 | -2 | - | 1677 | Sigma-54 dependent transcriptional regulator |
| CDS | contig00014 | 130689 | 131000 | 3 | + | 312 | hypothetical protein |
| CDS | contig00014 | 130997 | 132514 | 2 | + | 1518 | Aldehyde dehydrogenase (EC 1.2.1.3) |
| CDS | contig00014 | 133360 | 132566 | -1 | - | 795 | Transcriptional regulator, AraC family |
| CDS | contig00014 | 133457 | 134344 | 2 | + | 888 | Permease of the drug/metabolite transporter (DMT) superfamily |
| CDS | contig00014 | 134844 | 134440 | -3 | - | 405 | 4-carboxymuconolactone decarboxylase (EC 4.1.1.44) |
| CDS | contig00014 | 135824 | 134841 | -2 | - | 984 | Aldo-keto reductase |
| CDS | contig00014 | 135924 | 136832 | 3 | + | 909 | Transcriptional regulator, LysR family |
| CDS | contig00014 | 137640 | 136948 | -3 | - | 693 | Fumarate respiration transcriptional regulator DcuR |
| CDS | contig00014 | 139209 | 137644 | -3 | - | 1566 | Sensor kinase CitA, DpiB (EC 2.7.3.-) |
| CDS | contig00014 | 139468 | 139941 | 1 | + | 474 | Transcriptional regulator SlyA |
| CDS | contig00014 | 139943 | 140998 | 2 | + | 1056 | HlyD family secretion protein |
| CDS | contig00014 | 141003 | 142037 | 3 | + | 1035 | Permease of the major facilitator superfamily |
| CDS | contig00014 | 142221 | 142580 | 3 | + | 360 | FIG00545237: hypothetical protein |
| CDS | contig00014 | 143019 | 142639 | -3 | - | 381 | putative lipoprotein |
| CDS | contig00014 | 143335 | 143165 | -1 | - | 171 | protein of unknown function UPF0057 |
| CDS | contig00014 | 143902 | 143393 | -1 | - | 510 | Ribonucleotide reductase of class III (anaerobic), activating protein (EC 1.97.1.4) |
| CDS | contig00014 | 146130 | 144013 | -3 | - | 2118 | Ribonucleotide reductase of class III (anaerobic), large subunit (EC 1.17.4.2) |
| CDS | contig00014 | 146569 | 146399 | -1 | - | 171 | hypothetical protein |
| CDS | contig00014 | 146570 | 147538 | 2 | + | 969 | ABC-type sugar transport system, ATPase component |
| CDS | contig00014 | 149602 | 147596 | -1 | - | 2007 | DNA ligase (EC 6.5.1.2) |
| CDS | contig00014 | 150832 | 149702 | -1 | - | 1131 | Cell division protein ZipA |
| CDS | contig00014 | 154222 | 150848 | -1 | - | 3375 | Chromosome partition protein smc |
| CDS | contig00014 | 154333 | 155097 | 1 | + | 765 | Sulfate transporter, CysZ-type |
| CDS | contig00014 | 155232 | 155846 | 3 | + | 615 | Putative inner membrane protein |
| CDS | contig00014 | 156192 | 155941 | -3 | - | 252 | Phosphonate ABC transporter phosphate-binding periplasmic component (TC 3.A.1.9.1) |
| CDS | contig00014 | 156841 | 156296 | -1 | - | 546 | FIG001587: exported protein |
| CDS | contig00015 | 225 | 43 | -3 | - | 183 | hypothetical protein |
| CDS | contig00015 | 1605 | 280 | -3 | - | 1326 | Sensory histidine kinase in two-component regulatory system with RstA |
| CDS | contig00015 | 2541 | 1834 | -3 | - | 708 | Transcriptional regulatory protein RstA |
| CDS | contig00015 | 3938 | 2805 | -2 | - | 1134 | tRNA (Uracil54-C5-)-methyltransferase (EC 2.1.1.35) |
| CDS | contig00015 | 4105 | 5229 | 1 | + | 1125 | hypothetical protein |
| CDS | contig00015 | 5324 | 5857 | 2 | + | 534 | Uncharacterized conserved protein |
| CDS | contig00015 | 7892 | 6012 | -2 | - | 1881 | Maltodextrin glucosidase (EC 3.2.1.20) |
| CDS | contig00015 | 8917 | 8087 | -1 | - | 831 | Glutamate synthase [NADPH] large chain (EC 1.4.1.13) |
| CDS | contig00015 | 9424 | 9005 | -1 | - | 420 | heat shock protein HslJ |
| CDS | contig00015 | 10964 | 9603 | -2 | - | 1362 | Putative heat shock protein YegD |
| CDS | contig00015 | 12058 | 11069 | -1 | - | 990 | D-lactate dehydrogenase (EC 1.1.1.28) |
| CDS | contig00015 | 12615 | 12136 | -3 | - | 480 | Conserved uncharacterized protein CreA |
| CDS | contig00015 | 13083 | 12637 | -3 | - | 447 | Putative PRS2 protein |
| CDS | contig00015 | 14135 | 13275 | -2 | - | 861 | FIG00361730: hypothetical protein |
| CDS | contig00015 | 14678 | 14292 | -2 | - | 387 | C4-type zinc finger protein, DksA/TraR family |
| CDS | contig00015 | 15141 | 16472 | 3 | + | 1332 | glutamine synthetase family protein |
| CDS | contig00015 | 16760 | 16617 | -2 | - | 144 | hypothetical protein |
| CDS | contig00015 | 16764 | 18125 | 3 | + | 1362 | Omega-amino acid--pyruvate aminotransferase (EC 2.6.1.18) |
| CDS | contig00015 | 18180 | 19277 | 3 | + | 1098 | Putrescine ABC transporter putrescine-binding protein PotF (TC 3.A.1.11.2) |
| CDS | contig00015 | 19395 | 20480 | 3 | + | 1086 | Putrescine ABC transporter putrescine-binding protein PotF (TC 3.A.1.11.2) |
| CDS | contig00015 | 20557 | 21711 | 1 | + | 1155 | Putrescine transport ATP-binding protein PotG (TC 3.A.1.11.2) |
| CDS | contig00015 | 21708 | 22613 | 3 | + | 906 | Putrescine transport system permease protein PotH (TC 3.A.1.11.2) |
| CDS | contig00015 | 22610 | 23452 | 2 | + | 843 | Putrescine transport system permease protein PotI (TC 3.A.1.11.2) |
| CDS | contig00015 | 23501 | 24226 | 2 | + | 726 | FIG060329: MOSC domain protein |
| CDS | contig00015 | 25210 | 24683 | -1 | - | 528 | FIG00362117: hypothetical protein |
| CDS | contig00015 | 25789 | 25334 | -1 | - | 456 | putative protein PaaI, possibly involved in aromatic compounds catabolism |
| CDS | contig00015 | 25930 | 27765 | 1 | + | 1836 | ATP-dependent DNA helicase RecQ |
| CDS | contig00015 | 28184 | 27882 | -2 | - | 303 | Cytochrome c4 |
| CDS | contig00015 | 28370 | 29716 | 2 | + | 1347 | Selenoprotein O and cysteine-containing homologs |
| CDS | contig00015 | 29727 | 30014 | 3 | + | 288 | Aminopeptidase N |
| CDS | contig00015 | 30159 | 30833 | 3 | + | 675 | Putative preQ0 transporter |
| CDS | contig00015 | 31591 | 31106 | -1 | - | 486 | Ribonuclease E inhibitor RraA |
| CDS | contig00015 | 33170 | 31746 | -2 | - | 1425 | Magnesium transporter |
| CDS | contig00015 | 34709 | 33303 | -2 | - | 1407 | Siroheme synthase / Precorrin-2 oxidase (EC 1.3.1.76) / Sirohydrochlorin ferrochelatase (EC 4.99.1.4) / Uroporphyrinogen-III methyltransferase (EC 2.1.1.107) |
| CDS | contig00015 | 35534 | 34722 | -2 | - | 813 | Nitrite transporter NirC |
| CDS | contig00015 | 35988 | 35671 | -3 | - | 318 | Nitrite reductase [NAD(P)H] small subunit (EC 1.7.1.4) |
| CDS | contig00015 | 38635 | 36089 | -1 | - | 2547 | Nitrite reductase [NAD(P)H] large subunit (EC 1.7.1.4) |
| CDS | contig00015 | 40020 | 38932 | -3 | - | 1089 | ABC transport system, permease component YbhR |
| CDS | contig00015 | 41133 | 40024 | -3 | - | 1110 | ABC transport system, permease component YbhS |
| CDS | contig00015 | 42899 | 41130 | -2 | - | 1770 | ABC transporter multidrug efflux pump, fused ATP-binding domains |
| CDS | contig00015 | 43864 | 42896 | -1 | - | 969 | Predicted membrane fusion protein (MFP) component of efflux pump, membrane anchor protein YbhG |
| CDS | contig00015 | 44600 | 43959 | -2 | - | 642 | hypothetical protein |
| CDS | contig00015 | 45793 | 44873 | -1 | - | 921 | Transcriptional regulator, LysR family |
| CDS | contig00015 | 45962 | 47386 | 2 | + | 1425 | Multidrug resistance protein B |
| CDS | contig00015 | 48803 | 47475 | -2 | - | 1329 | ATP-dependent hsl protease ATP-binding subunit HslU |
| CDS | contig00015 | 48771 | 48911 | 3 | + | 141 | hypothetical protein |
| CDS | contig00015 | 49429 | 48896 | -1 | - | 534 | ATP-dependent protease HslV (EC 3.4.25.-) |
| CDS | contig00015 | 50464 | 49616 | -1 | - | 849 | Cell division protein FtsN |
| CDS | contig00015 | 52213 | 50468 | -1 | - | 1746 | Arginyl-tRNA synthetase (EC 6.1.1.19) |
| CDS | contig00015 | 52519 | 53094 | 1 | + | 576 | Uncharacterized protein, similar to the N-terminal domain of Lon protease |
| CDS | contig00015 | 53219 | 53788 | 2 | + | 570 | RNA polymerase sigma-70 factor, ECF subfamily |
| CDS | contig00015 | 53781 | 54446 | 3 | + | 666 | Transcriptional activator ChrR |
| CDS | contig00015 | 55374 | 54559 | -3 | - | 816 | Undecaprenyl-diphosphatase (EC 3.6.1.27) |
| CDS | contig00015 | 56624 | 55629 | -2 | - | 996 | Putative ion-channel protein |
| CDS | contig00015 | 56646 | 56789 | 3 | + | 144 | hypothetical protein |
| CDS | contig00015 | 57780 | 56773 | -3 | - | 1008 | Aldose 1-epimerase (EC 5.1.3.3) |
| CDS | contig00015 | 59016 | 57868 | -3 | - | 1149 | Galactokinase (EC 2.7.1.6) |
| CDS | contig00015 | 60071 | 59013 | -2 | - | 1059 | Galactose-1-phosphate uridylyltransferase (EC 2.7.7.10) |
| CDS | contig00015 | 61203 | 60178 | -3 | - | 1026 | UDP-glucose 4-epimerase (EC 5.1.3.2) |
| CDS | contig00015 | 61195 | 61356 | 1 | + | 162 | hypothetical protein |
| CDS | contig00015 | 61381 | 62412 | 1 | + | 1032 | Galactose operon repressor, GalR-LacI family of transcriptional regulators |
| CDS | contig00015 | 63078 | 66152 | 3 | + | 3075 | Beta-galactosidase (EC 3.2.1.23) |
| CDS | contig00015 | 66372 | 67358 | 3 | + | 987 | Galactose/methyl galactoside ABC transport system, D-galactose-binding periplasmic protein MglB (TC 3.A.1.2.3) |
| CDS | contig00015 | 67388 | 68932 | 2 | + | 1545 | Galactose/methyl galactoside ABC transport system, ATP-binding protein MglA (EC 3.6.3.17) |
| CDS | contig00015 | 68948 | 69958 | 2 | + | 1011 | Galactose/methyl galactoside ABC transport system, permease protein MglC (TC 3.A.1.2.3) |
| CDS | contig00015 | 70016 | 71749 | 2 | + | 1734 | Methyl-accepting chemotaxis protein II (mcp-II) (aspartate chemoreceptor protein) |
| CDS | contig00015 | 72668 | 72492 | -2 | - | 177 | FIG00361819: hypothetical protein |
| CDS | contig00015 | 73415 | 74635 | 2 | + | 1221 | Arginine deiminase (EC 3.5.3.6) |
| CDS | contig00015 | 74698 | 75624 | 1 | + | 927 | Carbamate kinase (EC 2.7.2.2) |
| CDS | contig00015 | 75703 | 76707 | 1 | + | 1005 | Ornithine carbamoyltransferase (EC 2.1.3.3) |
| CDS | contig00015 | 76829 | 78235 | 2 | + | 1407 | Arginine/ornithine antiporter ArcD |
| CDS | contig00015 | 78881 | 78390 | -2 | - | 492 | Arginine pathway regulatory protein ArgR, repressor of arg regulon |
| CDS | contig00015 | 79564 | 79100 | -1 | - | 465 | Aspartate carbamoyltransferase regulatory chain (PyrI) |
| CDS | contig00015 | 80501 | 79581 | -2 | - | 921 | Aspartate carbamoyltransferase (EC 2.1.3.2) |
| CDS | contig00015 | 81318 | 80776 | -3 | - | 543 | putative; ORF located using Glimmer/Genemark |
| CDS | contig00015 | 81807 | 83177 | 3 | + | 1371 | Phosphatidylserine decarboxylase (EC 4.1.1.65) |
| CDS | contig00015 | 84104 | 83232 | -2 | - | 873 | Transcriptional regulator, LysR family |
| CDS | contig00015 | 84205 | 84702 | 1 | + | 498 | Glyoxalase family protein |
| CDS | contig00015 | 84903 | 86792 | 3 | + | 1890 | Methyl-accepting chemotaxis protein |
| CDS | contig00015 | 87364 | 86921 | -1 | - | 444 | acetyltransferase, GNAT family |
| CDS | contig00015 | 87882 | 87361 | -3 | - | 522 | Outer membrane lipoprotein Blc |
| CDS | contig00015 | 88807 | 87884 | -1 | - | 924 | Hypothetical transcriptional regulator YqhC |
| CDS | contig00015 | 88926 | 90086 | 3 | + | 1161 | Hypothetical oxidoreductase YqhD (EC 1.1.-.-) |
| CDS | contig00015 | 90593 | 92119 | 2 | + | 1527 | NAD(P) transhydrogenase alpha subunit (EC 1.6.1.2) |
| CDS | contig00015 | 92131 | 93615 | 1 | + | 1485 | NAD(P) transhydrogenase subunit beta (EC 1.6.1.2) |
| CDS | contig00015 | 93939 | 94610 | 3 | + | 672 | YheO-like PAS domain |
| CDS | contig00015 | 94652 | 95944 | 2 | + | 1293 | Inner membrane protein |
| CDS | contig00015 | 96781 | 96017 | -1 | - | 765 | Probable component of the lipoprotein assembly complex (forms a complex with YaeT, YfgL, and NlpB) |
| CDS | contig00015 | 96889 | 97869 | 1 | + | 981 | Ribosomal large subunit pseudouridine synthase D (EC 4.2.1.70) |
| CDS | contig00015 | 97989 | 98726 | 3 | + | 738 | COG1496: Uncharacterized conserved protein |
| CDS | contig00015 | 98977 | 101553 | 1 | + | 2577 | ClpB protein |
| CDS | contig00015 | 102552 | 101647 | -3 | - | 906 | Transcriptional regulator, LysR family |
| CDS | contig00015 | 102724 | 103350 | 1 | + | 627 | Lysine exporter protein (LYSE/YGGA) |
| CDS | contig00015 | 103377 | 103883 | 3 | + | 507 | FIG00948579: hypothetical protein |
| CDS | contig00015 | 105130 | 104138 | -1 | - | 993 | Aspartate--ammonia ligase (EC 6.3.1.1) |
| CDS | contig00015 | 105285 | 105746 | 3 | + | 462 | Regulatory protein AsnC |
| CDS | contig00015 | 106588 | 105848 | -1 | - | 741 | Putative amino acid ABC transporter, periplasmic amino acid-binding protein |
| CDS | contig00015 | 107265 | 106861 | -3 | - | 405 | Inosine monophosphate dehydrogenase-related protein |
| CDS | contig00015 | 107425 | 108129 | 1 | + | 705 | FolM Alternative dihydrofolate reductase 1 |
| CDS | contig00015 | 108733 | 108188 | -1 | - | 546 | 2-amino-4-hydroxy-6-hydroxymethyldihydropteridine pyrophosphokinase (EC 2.7.6.3) |
| CDS | contig00015 | 109096 | 110466 | 1 | + | 1371 | Putative sensor-like histidine kinase YfhK |
| CDS | contig00015 | 110560 | 111081 | 1 | + | 522 | FIG00361780: hypothetical protein |
| CDS | contig00015 | 111136 | 112416 | 1 | + | 1281 | Putative sensory histidine kinase YfhA |
| CDS | contig00015 | 112950 | 112477 | -3 | - | 474 | FIG00361396: hypothetical protein |
| CDS | contig00015 | 113348 | 113235 | -2 | - | 114 | hypothetical protein |
| CDS | contig00015 | 113322 | 114332 | 3 | + | 1011 | Putative sulfite oxidase subunit YedY |
| CDS | contig00015 | 114424 | 115077 | 1 | + | 654 | FIG001196: Membrane protein YedZ |
| CDS | contig00015 | 115336 | 115088 | -1 | - | 249 | FIG00361608: hypothetical protein |
| CDS | contig00015 | 115400 | 115732 | 2 | + | 333 | Arginine/ornithine antiporter ArcD |
| CDS | contig00015 | 115820 | 116476 | 2 | + | 657 | Two-component system response regulator QseB |
| CDS | contig00015 | 116508 | 117854 | 3 | + | 1347 | Signal transduction histidine kinase |
| CDS | contig00015 | 117957 | 118418 | 3 | + | 462 | FIG00362259: hypothetical protein |
| CDS | contig00015 | 120966 | 118486 | -3 | - | 2481 | Trimethylamine-N-oxide reductase (EC 1.6.6.9) |
| CDS | contig00015 | 121372 | 120989 | -1 | - | 384 | Cytochrome c-type protein TorY |
| CDS | contig00015 | 122119 | 121385 | -1 | - | 735 | Cytochrome c-type protein TorY |
| CDS | contig00015 | 122418 | 124427 | 3 | + | 2010 | DNA topoisomerase III (EC 5.99.1.2) |
| CDS | contig00015 | 125856 | 124501 | -3 | - | 1356 | Potassium uptake protein, integral membrane component, KtrB |
| CDS | contig00015 | 126506 | 125853 | -2 | - | 654 | Trk system potassium uptake protein TrkA |
| CDS | contig00015 | 126632 | 127669 | 2 | + | 1038 | UDP-N-acetylenolpyruvoylglucosamine reductase (EC 1.1.1.158) |
| CDS | contig00015 | 127666 | 128631 | 1 | + | 966 | Biotin--protein ligase (EC 6.3.4.9, EC 6.3.4.10, EC 6.3.4.11, EC 6.3.4.15) / Biotin operon repressor |
| CDS | contig00015 | 129646 | 128708 | -1 | - | 939 | Pantothenate kinase (EC 2.7.1.33) |
| CDS | contig00017 | 13 | 273 | 1 | + | 261 | Translation elongation factor Tu |
| CDS | contig00017 | 505 | 834 | 1 | + | 330 | Preprotein translocase subunit SecE (TC 3.A.5.1.1) |
| CDS | contig00017 | 844 | 1395 | 1 | + | 552 | Transcription antitermination protein NusG |
| CDS | contig00017 | 1530 | 1958 | 3 | + | 429 | LSU ribosomal protein L11p (L12e) |
| CDS | contig00017 | 1963 | 2664 | 1 | + | 702 | LSU ribosomal protein L1p (L10Ae) |
| CDS | contig00017 | 2921 | 3421 | 2 | + | 501 | LSU ribosomal protein L10p (P0) |
| CDS | contig00017 | 3473 | 3838 | 2 | + | 366 | LSU ribosomal protein L7/L12 (P1/P2) |
| CDS | contig00017 | 4062 | 8090 | 3 | + | 4029 | DNA-directed RNA polymerase beta subunit (EC 2.7.7.6) |
| CDS | contig00017 | 8182 | 12486 | 1 | + | 4305 | DNA-directed RNA polymerase beta&#39; subunit (EC 2.7.7.6) |
| CDS | contig00017 | 12684 | 13400 | 3 | + | 717 | YheO-like PAS domain |
| CDS | contig00017 | 13427 | 13807 | 2 | + | 381 | Endoribonuclease L-PSP |
| CDS | contig00017 | 13894 | 14286 | 1 | + | 393 | tRNA 5-methylaminomethyl-2-thiouridine synthase TusD |
| CDS | contig00017 | 14428 | 14652 | 1 | + | 225 | tRNA 5-methylaminomethyl-2-thiouridine synthase TusC |
| CDS | contig00017 | 14690 | 14938 | 2 | + | 249 | tRNA 5-methylaminomethyl-2-thiouridine synthase TusB |
| CDS | contig00017 | 15120 | 15494 | 3 | + | 375 | SSU ribosomal protein S12p (S23e) |
| CDS | contig00017 | 15588 | 16058 | 3 | + | 471 | SSU ribosomal protein S7p (S5e) |
| CDS | contig00017 | 16134 | 18239 | 3 | + | 2106 | Translation elongation factor G |
| CDS | contig00018 | 19 | 273 | 1 | + | 255 | Translation elongation factor Tu |
| CDS | contig00018 | 387 | 929 | 3 | + | 543 | D-glycero-D-manno-heptose 1,7-bisphosphate phosphatase (EC 3.1.1.-) |
| CDS | contig00019 | 1112 | 285 | -2 | - | 828 | ADA regulatory protein / Methylated-DNA--protein-cysteine methyltransferase (EC 2.1.1.63) |
| CDS | contig00019 | 1927 | 1163 | -1 | - | 765 | 3-oxoacyl-[acyl-carrier protein] reductase (EC 1.1.1.100) |
| CDS | contig00019 | 2709 | 2338 | -3 | - | 372 | hypothetical protein |
| CDS | contig00019 | 3488 | 2706 | -2 | - | 783 | DNA alkylation repair enzyme |
| CDS | contig00019 | 3922 | 3485 | -1 | - | 438 | Transcriptional regulator, MarR family |
| CDS | contig00019 | 4274 | 5644 | 2 | + | 1371 | serine/threonine protein kinase |
| CDS | contig00019 | 5755 | 6402 | 1 | + | 648 | Thiopurine S-methyltransferase (EC 2.1.1.67) |
| CDS | contig00019 | 9462 | 6688 | -3 | - | 2775 | Protease III precursor (EC 3.4.24.55) |
| CDS | contig00019 | 9625 | 10092 | 1 | + | 468 | Phosphohistidine phosphatase SixA |
| CDS | contig00019 | 10226 | 10432 | 2 | + | 207 | FIG00362274: hypothetical protein |
| CDS | contig00019 | 10618 | 11538 | 1 | + | 921 | Permease of the drug/metabolite transporter (DMT) superfamily |
| CDS | contig00019 | 13202 | 11586 | -2 | - | 1617 | Oligopeptide ABC transporter, periplasmic oligopeptide-binding protein OppA (TC 3.A.1.5.1) |
| CDS | contig00019 | 13576 | 14610 | 1 | + | 1035 | Fructose-1,6-bisphosphatase, type I (EC 3.1.3.11) |
| CDS | contig00019 | 18633 | 15178 | -3 | - | 3456 | Transcription-repair coupling factor |
| CDS | contig00019 | 19260 | 18685 | -3 | - | 576 | FIG01200287: hypothetical protein |
| CDS | contig00019 | 19337 | 20572 | 2 | + | 1236 | Lipoprotein releasing system transmembrane protein LolC |
| CDS | contig00019 | 20613 | 21311 | 3 | + | 699 | Lipoprotein releasing system ATP-binding protein LolD |
| CDS | contig00019 | 21311 | 22552 | 2 | + | 1242 | Lipoprotein releasing system transmembrane protein LolE |
| CDS | contig00019 | 23273 | 22683 | -2 | - | 591 | Nucleotidase YfbR, HD superfamily |
| CDS | contig00019 | 23792 | 23358 | -2 | - | 435 | Inner membrane protein YccF |
| CDS | contig00019 | 24228 | 24097 | -3 | - | 132 | hypothetical protein |
| CDS | contig00019 | 24223 | 25533 | 1 | + | 1311 | Cell division trigger factor (EC 5.2.1.8) |
| CDS | contig00019 | 25620 | 26243 | 3 | + | 624 | ATP-dependent Clp protease proteolytic subunit (EC 3.4.21.92) |
| CDS | contig00019 | 26314 | 27588 | 1 | + | 1275 | ATP-dependent Clp protease ATP-binding subunit ClpX |
| CDS | contig00019 | 27728 | 30082 | 2 | + | 2355 | ATP-dependent protease La (EC 3.4.21.53) Type I |
| CDS | contig00019 | 30323 | 30595 | 2 | + | 273 | DNA-binding protein HU-beta |
| CDS | contig00019 | 30745 | 32658 | 1 | + | 1914 | Peptidyl-prolyl cis-trans isomerase PpiD (EC 5.2.1.8) |
| CDS | contig00019 | 34533 | 32941 | -3 | - | 1593 | Putative ATPase component of ABC transporter with duplicated ATPase domain |
| CDS | contig00019 | 36399 | 34744 | -3 | - | 1656 | Acetate permease ActP (cation/acetate symporter) |
| CDS | contig00019 | 36707 | 36396 | -2 | - | 312 | Putative membrane protein, clustering with ActP |
| CDS | contig00019 | 37441 | 36815 | -1 | - | 627 | DNA polymerase III epsilon subunit (EC 2.7.7.7) |
| CDS | contig00019 | 39231 | 37444 | -3 | - | 1788 | Predicted signal-transduction protein containing cAMP-binding and CBS domains |
| CDS | contig00019 | 39389 | 40195 | 2 | + | 807 | Enoyl-CoA hydratase (EC 4.2.1.17) |
| CDS | contig00019 | 41762 | 40263 | -2 | - | 1500 | Sodium/alanine symporter family protein |
| CDS | contig00019 | 42026 | 41910 | -2 | - | 117 | hypothetical protein |
| CDS | contig00019 | 42069 | 42506 | 3 | + | 438 | PhnO protein |
| CDS | contig00019 | 42525 | 43334 | 3 | + | 810 | Inner membrane protein |
| CDS | contig00019 | 44009 | 43413 | -2 | - | 597 | FIG00361445: hypothetical protein |
| CDS | contig00019 | 48002 | 45108 | -2 | - | 2895 | putative exported protein |
| CDS | contig00019 | 48699 | 48286 | -3 | - | 414 | Predicted transcriptional regulator LiuR of leucine degradation pathway, MerR family |
| CDS | contig00019 | 49725 | 48754 | -3 | - | 972 | Hydroxymethylglutaryl-CoA lyase (EC 4.1.3.4) |
| CDS | contig00019 | 51686 | 49722 | -2 | - | 1965 | Methylcrotonyl-CoA carboxylase biotin-containing subunit (EC 6.4.1.4) |
| CDS | contig00019 | 52644 | 51796 | -3 | - | 849 | Methylglutaconyl-CoA hydratase (EC 4.2.1.18) |
| CDS | contig00019 | 54270 | 52654 | -3 | - | 1617 | Methylcrotonyl-CoA carboxylase carboxyl transferase subunit (EC 6.4.1.4) |
| CDS | contig00019 | 55415 | 54267 | -2 | - | 1149 | Isovaleryl-CoA dehydrogenase (EC 1.3.8.4) |
| CDS | contig00019 | 55684 | 55565 | -1 | - | 120 | hypothetical protein |
| CDS | contig00019 | 55673 | 57184 | 2 | + | 1512 | Methylmalonate-semialdehyde dehydrogenase (EC 1.2.1.27) |
| CDS | contig00019 | 57266 | 58423 | 2 | + | 1158 | Branched-chain acyl-CoA dehydrogenase (EC 1.3.99.12) |
| CDS | contig00019 | 58542 | 59333 | 3 | + | 792 | 3-hydroxyisobutyryl-CoA hydrolase (EC 3.1.2.4) |
| CDS | contig00019 | 59424 | 60512 | 3 | + | 1089 | 3-hydroxyisobutyryl-CoA hydrolase (EC 3.1.2.4) |
| CDS | contig00019 | 60540 | 61460 | 3 | + | 921 | 3-hydroxyisobutyrate dehydrogenase (EC 1.1.1.31) |
| CDS | contig00019 | 61532 | 62293 | 2 | + | 762 | 3-oxoacyl-[acyl-carrier protein] reductase (EC 1.1.1.100) |
| CDS | contig00019 | 62659 | 64095 | 1 | + | 1437 | Arginine/ornithine antiporter ArcD |
| CDS | contig00019 | 64188 | 64976 | 3 | + | 789 | Protein of unknown function DUF81 |
| CDS | contig00019 | 65273 | 66421 | 2 | + | 1149 | site-specific recombinase, phage integrase family |
| CDS | contig00019 | 69586 | 69699 | 1 | + | 114 | hypothetical protein |
| CDS | contig00019 | 71954 | 71835 | -2 | - | 120 | hypothetical protein |
| CDS | contig00019 | 72138 | 72260 | 3 | + | 123 | hypothetical protein |
| CDS | contig00019 | 72673 | 72557 | -1 | - | 117 | hypothetical protein |
| CDS | contig00019 | 72763 | 73347 | 1 | + | 585 | hypothetical protein |
| CDS | contig00019 | 73853 | 74992 | 2 | + | 1140 | site-specific recombinase, phage integrase family |
| CDS | contig00019 | 77852 | 75189 | -2 | - | 2664 | putative DNA primase/helicase |
| CDS | contig00019 | 78445 | 80406 | 1 | + | 1962 | Type I restriction-modification system, DNA-methyltransferase subunit M (EC 2.1.1.72) |
| CDS | contig00019 | 80403 | 81758 | 3 | + | 1356 | Type I restriction-modification system, specificity subunit S (EC 3.1.21.3) |
| CDS | contig00019 | 81770 | 82063 | 2 | + | 294 | hypothetical protein |
| CDS | contig00019 | 82073 | 82462 | 2 | + | 390 | Nucleotidyltransferase (EC 2.7.7.-) |
| CDS | contig00019 | 82459 | 85656 | 1 | + | 3198 | Type I restriction-modification system, restriction subunit R (EC 3.1.21.3) |
| CDS | contig00019 | 86560 | 87027 | 1 | + | 468 | DNA repair protein RadC |
| CDS | contig00019 | 87024 | 87392 | 3 | + | 369 | hypothetical protein |
| CDS | contig00019 | 87398 | 87538 | 2 | + | 141 | hypothetical protein |
| CDS | contig00019 | 87535 | 87672 | 1 | + | 138 | hypothetical protein |
| CDS | contig00019 | 87912 | 88457 | 3 | + | 546 | Site-specific recombinase, resolvase family |
| CDS | contig00019 | 88740 | 90050 | 3 | + | 1311 | FIG01206370: hypothetical protein |
| CDS | contig00019 | 91745 | 92845 | 2 | + | 1101 | Outer membrane protein romA |
| CDS | contig00019 | 94035 | 93028 | -3 | - | 1008 | Ribose ABC transport system, permease protein RbsC (TC 3.A.1.2.1) |
| CDS | contig00019 | 95047 | 94028 | -1 | - | 1020 | Ribose ABC transport system, permease protein RbsC (TC 3.A.1.2.1) |
| CDS | contig00019 | 96563 | 95049 | -2 | - | 1515 | Putative sugar ABC transport system, ATP-binding protein YtfR (EC 3.6.3.17) |
| CDS | contig00019 | 97602 | 96643 | -3 | - | 960 | Putative sugar ABC transport system, periplasmic binding protein YtfQ precursor |
| CDS | contig00019 | 98839 | 97886 | -1 | - | 954 | Transcriptional regulator, LysR family |
| CDS | contig00019 | 98969 | 99820 | 2 | + | 852 | ThiJ/PfpI family protein |
| CDS | contig00019 | 100761 | 99883 | -3 | - | 879 | Hydrogen peroxide-inducible genes activator |
| CDS | contig00019 | 100923 | 101708 | 3 | + | 786 | Formate dehydrogenase chain D (EC 1.2.1.2) |
| CDS | contig00019 | 101692 | 103989 | 1 | + | 2298 | Putative formate dehydrogenase oxidoreductase protein |
| CDS | contig00019 | 105647 | 104073 | -2 | - | 1575 | FOG: GGDEF domain |
| CDS | contig00019 | 105612 | 105725 | 3 | + | 114 | hypothetical protein |
| CDS | contig00019 | 106666 | 105749 | -1 | - | 918 | Transcriptional regulator |
| CDS | contig00019 | 106830 | 108263 | 3 | + | 1434 | Beta-glucosidase (EC 3.2.1.21); 6-phospho-beta-glucosidase (EC 3.2.1.86) |
| CDS | contig00019 | 109117 | 108359 | -1 | - | 759 | Reductase |
| CDS | contig00019 | 109248 | 110183 | 3 | + | 936 | Transcriptional regulator |
| CDS | contig00019 | 111173 | 110163 | -2 | - | 1011 | Transcriptional regulator, AraC family |
| CDS | contig00019 | 111416 | 111300 | -2 | - | 117 | hypothetical protein |
| CDS | contig00019 | 111405 | 113441 | 3 | + | 2037 | Beta-hexosaminidase (EC 3.2.1.52) |
| CDS | contig00019 | 113611 | 114468 | 1 | + | 858 | Endonuclease IV (EC 3.1.21.2) |
| CDS | contig00019 | 114504 | 115481 | 3 | + | 978 | MGC89088 protein |
| CDS | contig00019 | 116451 | 115567 | -3 | - | 885 | Transcriptional regulator, LysR family |
| CDS | contig00019 | 116522 | 117535 | 2 | + | 1014 | Tellurite resistance protein |
| CDS | contig00019 | 117756 | 118745 | 3 | + | 990 | Lipid A biosynthesis (KDO) 2-(lauroyl)-lipid IVA acyltransferase (EC 2.3.1.-) |
| CDS | contig00019 | 118947 | 118813 | -3 | - | 135 | hypothetical protein |
| CDS | contig00019 | 118876 | 119895 | 1 | + | 1020 | Lipid A biosynthesis lauroyl acyltransferase (EC 2.3.1.-) |
| CDS | contig00019 | 119990 | 120850 | 2 | + | 861 | FIG00361278: hypothetical protein |
| CDS | contig00019 | 121788 | 120910 | -3 | - | 879 | Pirin-related protein |
| CDS | contig00019 | 122026 | 124428 | 1 | + | 2403 | DNA polymerase II (EC 2.7.7.7) |
| CDS | contig00019 | 126796 | 125336 | -1 | - | 1461 | Multi antimicrobial extrusion protein (Na(+)/drug antiporter), MATE family of MDR efflux pumps |
| CDS | contig00019 | 127073 | 126867 | -2 | - | 207 | FIG00361744: hypothetical protein |
| CDS | contig00019 | 127207 | 127818 | 1 | + | 612 | Riboflavin synthase eubacterial/eukaryotic (EC 2.5.1.9) |
| CDS | contig00019 | 127921 | 128211 | 1 | + | 291 | hypothetical protein |
| CDS | contig00019 | 128492 | 128289 | -2 | - | 204 | FIG00362276: hypothetical protein |
| CDS | contig00019 | 129282 | 128623 | -3 | - | 660 | 2-deoxyglucose-6-phosphate hydrolase YniC |
| CDS | contig00019 | 130589 | 129411 | -2 | - | 1179 | Phosphoribosylglycinamide formyltransferase 2 (EC 2.1.2.-) |
| CDS | contig00019 | 131557 | 130613 | -1 | - | 945 | Putative transporter, DME family |
| CDS | contig00019 | 131676 | 132371 | 3 | + | 696 | FMN-dependent NADH-azoreductase |
| CDS | contig00019 | 132646 | 132467 | -1 | - | 180 | hypothetical protein |
| CDS | contig00019 | 132996 | 132808 | -3 | - | 189 | FIG00362240: hypothetical protein |
| CDS | contig00019 | 133463 | 132993 | -2 | - | 471 | Transcriptional regulator, MarR family |
| CDS | contig00019 | 134783 | 133614 | -2 | - | 1170 | hypothetical protein |
| CDS | contig00020 | 92 | 544 | 2 | + | 453 | FIG106692: Outer membrane lipoprotein |
| CDS | contig00020 | 716 | 1369 | 2 | + | 654 | Protein of unknown function YceH |
| CDS | contig00020 | 1588 | 5424 | 1 | + | 3837 | COG0553: Superfamily II DNA/RNA helicases, SNF2 family |
| CDS | contig00020 | 6396 | 5503 | -3 | - | 894 | DNA replication terminus site-binding protein |
| CDS | contig00020 | 6463 | 6594 | 1 | + | 132 | hypothetical protein |
| CDS | contig00020 | 6927 | 7136 | 3 | + | 210 | FIG00361806: hypothetical protein |
| CDS | contig00020 | 7420 | 7292 | -1 | - | 129 | hypothetical protein |
| CDS | contig00020 | 7406 | 8425 | 2 | + | 1020 | Succinylglutamate desuccinylase (EC 3.5.1.96) |
| CDS | contig00020 | 8697 | 9785 | 3 | + | 1089 | Branched-chain alpha-keto acid dehydrogenase, E1 component, alpha subunit (EC 1.2.4.4) |
| CDS | contig00020 | 9782 | 10768 | 2 | + | 987 | Branched-chain alpha-keto acid dehydrogenase, E1 component, alpha subunit (EC 1.2.4.4) / Branched-chain alpha-keto acid dehydrogenase, E1 component, beta subunit (EC 1.2.4.4) |
| CDS | contig00020 | 10827 | 11963 | 3 | + | 1137 | Dihydrolipoamide acyltransferase component of branched-chain alpha-keto acid dehydrogenase complex (EC 2.3.1.168) |
| CDS | contig00020 | 12179 | 12379 | 2 | + | 201 | Cold shock protein CspE |
| CDS | contig00020 | 12456 | 12767 | 3 | + | 312 | hypothetical protein |
| CDS | contig00020 | 13002 | 12754 | -3 | - | 249 | DNA-binding protein inhibitor Id-2-related protein |
| CDS | contig00020 | 13249 | 13797 | 1 | + | 549 | hypothetical protein |
| CDS | contig00020 | 13892 | 14284 | 2 | + | 393 | FIG00361547: hypothetical protein |
| CDS | contig00020 | 16224 | 14374 | -3 | - | 1851 | Oligopeptide ABC transporter, periplasmic oligopeptide-binding protein OppA (TC 3.A.1.5.1) |
| CDS | contig00020 | 17208 | 16315 | -3 | - | 894 | Permease of the drug/metabolite transporter (DMT) superfamily |
| CDS | contig00020 | 17387 | 17575 | 2 | + | 189 | FIG00361194: hypothetical protein |
| CDS | contig00020 | 18762 | 17572 | -3 | - | 1191 | HD domain protein |
| CDS | contig00020 | 18935 | 19627 | 2 | + | 693 | Ribosomal small subunit pseudouridine synthase A (EC 4.2.1.70) |
| CDS | contig00020 | 20627 | 19725 | -2 | - | 903 | Permease of the drug/metabolite transporter (DMT) superfamily |
| CDS | contig00020 | 20918 | 22555 | 2 | + | 1638 | Oligopeptide ABC transporter, periplasmic oligopeptide-binding protein OppA (TC 3.A.1.5.1) |
| CDS | contig00020 | 22566 | 23108 | 3 | + | 543 | FIG002292: Phosphodiesterase yfcE (EC 3.1.4.-) |
| CDS | contig00020 | 23568 | 23152 | -3 | - | 417 | Flagellar biosynthesis protein FlgN |
| CDS | contig00020 | 23885 | 23565 | -2 | - | 321 | Negative regulator of flagellin synthesis FlgM |
| CDS | contig00020 | 24609 | 23980 | -3 | - | 630 | Flagellar basal-body P-ring formation protein FlgA |
| CDS | contig00020 | 24823 | 25734 | 1 | + | 912 | Chemotaxis protein CheV (EC 2.7.3.-) |
| CDS | contig00020 | 25752 | 26576 | 3 | + | 825 | Chemotaxis protein methyltransferase CheR (EC 2.1.1.80) |
| CDS | contig00020 | 26638 | 27039 | 1 | + | 402 | Flagellar basal-body rod protein FlgB |
| CDS | contig00020 | 27036 | 27455 | 3 | + | 420 | Flagellar basal-body rod protein FlgC |
| CDS | contig00020 | 27546 | 28283 | 3 | + | 738 | Flagellar basal-body rod modification protein FlgD |
| CDS | contig00020 | 28294 | 29640 | 1 | + | 1347 | Flagellar hook protein FlgE |
| CDS | contig00020 | 29787 | 30533 | 3 | + | 747 | Flagellar basal-body rod protein FlgF |
| CDS | contig00020 | 30548 | 31336 | 2 | + | 789 | Flagellar basal-body rod protein FlgG |
| CDS | contig00020 | 31350 | 32024 | 3 | + | 675 | Flagellar L-ring protein FlgH |
| CDS | contig00020 | 32085 | 33182 | 3 | + | 1098 | Flagellar P-ring protein FlgI |
| CDS | contig00020 | 33229 | 34329 | 1 | + | 1101 | Flagellar protein FlgJ [peptidoglycan hydrolase] (EC 3.2.1.-) |
| CDS | contig00020 | 34333 | 36330 | 1 | + | 1998 | Flagellar hook-associated protein FlgK |
| CDS | contig00020 | 36334 | 37764 | 1 | + | 1431 | Flagellar hook-associated protein FlgL |
| CDS | contig00020 | 38012 | 39355 | 2 | + | 1344 | Magnesium transporter |
| CDS | contig00020 | 40854 | 39415 | -3 | - | 1440 | Exopolyphosphatase (EC 3.6.1.11) |
| CDS | contig00020 | 42964 | 40895 | -1 | - | 2070 | Polyphosphate kinase (EC 2.7.4.1) |
| CDS | contig00020 | 43553 | 43041 | -2 | - | 513 | ACT domain protein |
| CDS | contig00020 | 43812 | 44651 | 3 | + | 840 | Potential queD like |
| CDS | contig00020 | 44897 | 45682 | 2 | + | 786 | Flagellar regulatory protein FleQ |
| CDS | contig00020 | 45673 | 46308 | 1 | + | 636 | Flagellar regulatory protein FleQ |
| CDS | contig00020 | 46414 | 47448 | 1 | + | 1035 | Flagellar sensor histidine kinase FleS |
| CDS | contig00020 | 47519 | 48853 | 2 | + | 1335 | Flagellar regulatory protein FleQ |
| CDS | contig00020 | 48961 | 49722 | 1 | + | 762 | FIG137478: Hypothetical protein YbgI |
| CDS | contig00020 | 50508 | 49837 | -3 | - | 672 | Phosphoribosylglycinamide formyltransferase (EC 2.1.2.2) |
| CDS | contig00020 | 51542 | 50505 | -2 | - | 1038 | Phosphoribosylformylglycinamidine cyclo-ligase (EC 6.3.3.1) |
| CDS | contig00020 | 51719 | 52345 | 2 | + | 627 | Uracil phosphoribosyltransferase (EC 2.4.2.9) |
| CDS | contig00020 | 52531 | 53514 | 1 | + | 984 | FIG00921138: hypothetical protein |
| CDS | contig00020 | 53584 | 54318 | 1 | + | 735 | Chromosomal replication initiator protein DnaA |
| CDS | contig00020 | 55864 | 54533 | -1 | - | 1332 | Isocitrate lyase (EC 4.1.3.1) |
| CDS | contig00020 | 57523 | 55916 | -1 | - | 1608 | Malate synthase (EC 2.3.3.9) |
| CDS | contig00020 | 58355 | 58215 | -2 | - | 141 | hypothetical protein |
| CDS | contig00020 | 59068 | 59616 | 1 | + | 549 | tRNA-specific adenosine-34 deaminase (EC 3.5.4.-) |
| CDS | contig00020 | 59778 | 62015 | 3 | + | 2238 | Phosphate transport system permease protein PstC (TC 3.A.1.7.1) |
| CDS | contig00020 | 62033 | 63682 | 2 | + | 1650 | Phosphate transport system permease protein PstA (TC 3.A.1.7.1) |
| CDS | contig00020 | 63819 | 63691 | -3 | - | 129 | hypothetical protein |
| CDS | contig00020 | 63787 | 64605 | 1 | + | 819 | Phosphate transport ATP-binding protein PstB (TC 3.A.1.7.1) |
| CDS | contig00020 | 64705 | 65415 | 1 | + | 711 | Phosphate transport system regulatory protein PhoU |
| CDS | contig00020 | 65657 | 66976 | 2 | + | 1320 | Putative hemolysin |
| CDS | contig00020 | 66995 | 67537 | 2 | + | 543 | FIG00361380: hypothetical protein |
| CDS | contig00020 | 67830 | 68732 | 3 | + | 903 | Homocysteine S-methyltransferase (EC 2.1.1.10) |
| CDS | contig00020 | 68742 | 70151 | 3 | + | 1410 | Arginine/ornithine antiporter ArcD |
| CDS | contig00020 | 71253 | 70219 | -3 | - | 1035 | Sterol desaturase |
| CDS | contig00020 | 72003 | 71260 | -3 | - | 744 | Glutamine transport ATP-binding protein GlnQ (TC 3.A.1.3.2) |
| CDS | contig00020 | 72109 | 71987 | -1 | - | 123 | hypothetical protein |
| CDS | contig00020 | 73064 | 72093 | -2 | - | 972 | Amino acid ABC transporter, permease protein |
| CDS | contig00020 | 73925 | 73137 | -2 | - | 789 | Lysine-arginine-ornithine-binding periplasmic protein precursor (TC 3.A.1.3.1) |
| CDS | contig00020 | 74387 | 75520 | 2 | + | 1134 | diguanylate cyclase (GGDEF domain) with PAS/PAC sensor |
| CDS | contig00020 | 76024 | 75629 | -1 | - | 396 | Regulator of nucleoside diphosphate kinase |
| CDS | contig00020 | 76992 | 76258 | -3 | - | 735 | ABC-type amino acid transport, signal transduction systems, periplasmic component/domain |
| CDS | contig00020 | 77757 | 77074 | -3 | - | 684 | FIG00361344: hypothetical protein |
| CDS | contig00020 | 79187 | 77808 | -2 | - | 1380 | Alkaline phosphatase (EC 3.1.3.1) |
| CDS | contig00020 | 80591 | 79200 | -2 | - | 1392 | Alkaline phosphatase (EC 3.1.3.1) |
| CDS | contig00020 | 80911 | 80732 | -1 | - | 180 | hypothetical protein |
| CDS | contig00020 | 80933 | 81883 | 2 | + | 951 | 50S ribosomal subunit maturation GTPase RbgA (B. subtilis YlqF) |
| CDS | contig00020 | 82230 | 82343 | 3 | + | 114 | hypothetical protein |
| CDS | contig00020 | 82403 | 83551 | 2 | + | 1149 | Hypothetical MFS-type transporter protein ycaD |
| CDS | contig00020 | 84537 | 83641 | -3 | - | 897 | Histone deacetylase/AcuC/AphA family protein |
| CDS | contig00020 | 85267 | 84608 | -1 | - | 660 | putative secreted protein |
| CDS | contig00020 | 87553 | 85481 | -1 | - | 2073 | ATP-dependent helicase DinG/Rad3 |
| CDS | contig00020 | 87936 | 89021 | 3 | + | 1086 | Outer membrane protein C precursor |
| CDS | contig00020 | 89252 | 90193 | 2 | + | 942 | hypothetical protein |
| CDS | contig00020 | 90329 | 91519 | 2 | + | 1191 | Biosynthetic Aromatic amino acid aminotransferase alpha (EC 2.6.1.57) @ Aspartate aminotransferase (EC 2.6.1.1) |
| CDS | contig00020 | 93104 | 91599 | -2 | - | 1506 | ATPase component BioM of energizing module of biotin ECF transporter |
| CDS | contig00020 | 93714 | 93220 | -3 | - | 495 | FIG00361382: hypothetical protein |
| CDS | contig00020 | 94226 | 93708 | -2 | - | 519 | FIG01199889: hypothetical protein |
| CDS | contig00020 | 94342 | 96483 | 1 | + | 2142 | DNA internalization-related competence protein ComEC/Rec2 |
| CDS | contig00020 | 96618 | 98387 | 3 | + | 1770 | Lipid A export ATP-binding/permease protein MsbA |
| CDS | contig00020 | 98387 | 99382 | 2 | + | 996 | Tetraacyldisaccharide 4&#39;-kinase (EC 2.7.1.130) |
| CDS | contig00020 | 99495 | 99692 | 3 | + | 198 | FIG002473: Protein YcaR in KDO2-Lipid A biosynthesis cluster |
| CDS | contig00020 | 99689 | 100468 | 2 | + | 780 | 3-deoxy-manno-octulosonate cytidylyltransferase (EC 2.7.7.38) |
| CDS | contig00020 | 100738 | 101322 | 1 | + | 585 | BarA-associated response regulator UvrY (= GacA = SirA) |
| CDS | contig00020 | 101433 | 103286 | 3 | + | 1854 | Excinuclease ABC subunit C |
| CDS | contig00020 | 103500 | 104054 | 3 | + | 555 | CDP-diacylglycerol--glycerol-3-phosphate 3-phosphatidyltransferase (EC 2.7.8.5) |
| CDS | contig00020 | 105119 | 104859 | -2 | - | 261 | Probable transcription regulator protein of MDR efflux pump cluster |
| CDS | contig00020 | 106129 | 107307 | 1 | + | 1179 | hypothetical protein |
| CDS | contig00020 | 107313 | 107795 | 3 | + | 483 | Integral membrane protein |
| CDS | contig00020 | 108941 | 107958 | -2 | - | 984 | Alcohol dehydrogenase (EC 1.1.1.1) |
| CDS | contig00020 | 109629 | 108994 | -3 | - | 636 | Transcriptional regulator, TetR family |
| CDS | contig00020 | 110035 | 109787 | -1 | - | 249 | Transglycosylase associated protein |
| CDS | contig00020 | 110889 | 110164 | -3 | - | 726 | UDP-2,3-diacylglucosamine diphosphatase (EC 3.6.1.54) |
| CDS | contig00020 | 111589 | 110903 | -1 | - | 687 | hypothetical protein |
| CDS | contig00020 | 112076 | 111579 | -2 | - | 498 | Peptidyl-prolyl cis-trans isomerase PpiB (EC 5.2.1.8) |
| CDS | contig00020 | 112268 | 113644 | 2 | + | 1377 | Cysteinyl-tRNA synthetase (EC 6.1.1.16) |
| CDS | contig00020 | 114237 | 113878 | -3 | - | 360 | Putative periplasmic protein |
| CDS | contig00020 | 114333 | 114947 | 3 | + | 615 | hypothetical protein |
| CDS | contig00021 | 537 | 154 | -3 | - | 384 | LSU ribosomal protein L17p |
| CDS | contig00021 | 1570 | 581 | -1 | - | 990 | DNA-directed RNA polymerase alpha subunit (EC 2.7.7.6) |
| CDS | contig00021 | 2060 | 1596 | -2 | - | 465 | SSU ribosomal protein S4p (S9e) |
| CDS | contig00021 | 2411 | 2256 | -2 | - | 156 | SSU ribosomal protein S11p (S14e) |
| CDS | contig00021 | 3016 | 2660 | -1 | - | 357 | SSU ribosomal protein S13p (S18e) |
| CDS | contig00021 | 4625 | 3297 | -2 | - | 1329 | Preprotein translocase secY subunit (TC 3.A.5.1.1) |
| CDS | contig00021 | 5071 | 4634 | -1 | - | 438 | LSU ribosomal protein L15p (L27Ae) |
| CDS | contig00021 | 5253 | 5074 | -3 | - | 180 | LSU ribosomal protein L30p (L7e) |
| CDS | contig00021 | 5760 | 5260 | -3 | - | 501 | SSU ribosomal protein S5p (S2e) |
| CDS | contig00021 | 6077 | 5775 | -2 | - | 303 | LSU ribosomal protein L18p (L5e) |
| CDS | contig00021 | 6671 | 6138 | -2 | - | 534 | LSU ribosomal protein L6p (L9e) |
| CDS | contig00021 | 7077 | 6685 | -3 | - | 393 | SSU ribosomal protein S8p (S15Ae) |
| CDS | contig00021 | 7402 | 7097 | -1 | - | 306 | SSU ribosomal protein S14p (S29e) @ SSU ribosomal protein S14p (S29e), zinc-independent |
| CDS | contig00021 | 7952 | 7413 | -2 | - | 540 | LSU ribosomal protein L5p (L11e) |
| CDS | contig00021 | 8283 | 7966 | -3 | - | 318 | LSU ribosomal protein L24p (L26e) |
| CDS | contig00021 | 8663 | 8295 | -2 | - | 369 | LSU ribosomal protein L14p (L23e) |
| CDS | contig00021 | 9060 | 8812 | -3 | - | 249 | SSU ribosomal protein S17p (S11e) |
| CDS | contig00021 | 9251 | 9060 | -2 | - | 192 | LSU ribosomal protein L29p (L35e) |
| CDS | contig00021 | 9664 | 9251 | -1 | - | 414 | LSU ribosomal protein L16p (L10e) |
| CDS | contig00021 | 10391 | 9678 | -2 | - | 714 | SSU ribosomal protein S3p (S3e) |
| CDS | contig00021 | 10739 | 10395 | -2 | - | 345 | LSU ribosomal protein L22p (L17e) |
| CDS | contig00021 | 11028 | 10750 | -3 | - | 279 | SSU ribosomal protein S19p (S15e) |
| CDS | contig00021 | 11872 | 11051 | -1 | - | 822 | LSU ribosomal protein L2p (L8e) |
| CDS | contig00021 | 12190 | 11888 | -1 | - | 303 | LSU ribosomal protein L23p (L23Ae) |
| CDS | contig00021 | 12792 | 12187 | -3 | - | 606 | LSU ribosomal protein L4p (L1e) |
| CDS | contig00021 | 13442 | 12810 | -2 | - | 633 | LSU ribosomal protein L3p (L3e) |
| CDS | contig00021 | 13776 | 13465 | -3 | - | 312 | SSU ribosomal protein S10p (S20e) |
| CDS | contig00021 | 14131 | 15045 | 1 | + | 915 | Permease of the drug/metabolite transporter (DMT) superfamily |
| CDS | contig00021 | 16392 | 15082 | -3 | - | 1311 | Osmolarity sensory histidine kinase EnvZ |
| CDS | contig00021 | 17108 | 16389 | -2 | - | 720 | Two-component system response regulator OmpR |
| CDS | contig00021 | 17311 | 17784 | 1 | + | 474 | Transcription elongation factor GreB |
| CDS | contig00021 | 18076 | 17768 | -1 | - | 309 | hypothetical protein |
| CDS | contig00021 | 18053 | 20359 | 2 | + | 2307 | Transcription accessory protein (S1 RNA-binding domain) |
| CDS | contig00021 | 21182 | 20418 | -2 | - | 765 | SrpA-related protein |
| CDS | contig00021 | 21417 | 21184 | -3 | - | 234 | FIG00361911: hypothetical protein |
| CDS | contig00021 | 22254 | 21490 | -3 | - | 765 | Biotin synthesis protein BioH |
| CDS | contig00021 | 22293 | 23021 | 3 | + | 729 | Competence protein F homolog, phosphoribosyltransferase domain; protein YhgH required for utilization of DNA as sole source of carbon and energy |
| CDS | contig00021 | 23170 | 23748 | 1 | + | 579 | NfuA Fe-S protein maturation |
| CDS | contig00021 | 24822 | 23818 | -3 | - | 1005 | Glycerol-3-phosphate dehydrogenase [NAD(P)+] (EC 1.1.1.94) |
| CDS | contig00021 | 25295 | 24825 | -2 | - | 471 | Protein export cytoplasm chaperone protein (SecB, maintains protein to be exported in unfolded state) |
| CDS | contig00021 | 25774 | 25343 | -1 | - | 432 | FIG136845: Rhodanese-related sulfurtransferase |
| CDS | contig00021 | 26123 | 27652 | 2 | + | 1530 | 2,3-bisphosphoglycerate-independent phosphoglycerate mutase (EC 5.4.2.1) |
| CDS | contig00021 | 27710 | 29065 | 2 | + | 1356 | Lipoprotein NlpD |
| CDS | contig00021 | 30042 | 29356 | -3 | - | 687 | FIG00361592: hypothetical protein |
| CDS | contig00021 | 30095 | 31102 | 2 | + | 1008 | Gluconate utilization system Gnt-I transcriptional repressor |
| CDS | contig00021 | 31213 | 31602 | 1 | + | 390 | Uncharacterized protein C11D3.04c |
| CDS | contig00021 | 32350 | 31658 | -1 | - | 693 | Transcriptional regulator, AraC family |
| CDS | contig00021 | 33130 | 32489 | -1 | - | 642 | 4-hydroxy-2-oxoglutarate aldolase (EC 4.1.3.16) @ 2-dehydro-3-deoxyphosphogluconate aldolase (EC 4.1.2.14) |
| CDS | contig00021 | 34936 | 33140 | -1 | - | 1797 | Phosphogluconate dehydratase (EC 4.2.1.12) |
| CDS | contig00021 | 35506 | 34997 | -1 | - | 510 | Gluconokinase (EC 2.7.1.12) |
| CDS | contig00021 | 35660 | 37033 | 2 | + | 1374 | Low-affinity gluconate/H+ symporter GntU |
| CDS | contig00021 | 37186 | 38535 | 1 | + | 1350 | CDP-diacylglycerol--serine O-phosphatidyltransferase (EC 2.7.8.8) |
| CDS | contig00021 | 38712 | 40781 | 3 | + | 2070 | ATP-dependent DNA helicase RecG (EC 3.6.1.-) |
| CDS | contig00021 | 40924 | 42984 | 1 | + | 2061 | Putative exported protein |
| CDS | contig00021 | 43939 | 43031 | -1 | - | 909 | GNAT family acetyltransferase YiiD potentially involved in tRNA processing |
| CDS | contig00021 | 44188 | 44793 | 1 | + | 606 | Spermidine synthase (EC 2.5.1.16) |
| CDS | contig00021 | 45297 | 44860 | -3 | - | 438 | D-tyrosyl-tRNA(Tyr) deacylase (EC 3.6.1.n1) |
| CDS | contig00021 | 46296 | 45424 | -3 | - | 873 | Inner membrane protein YihY, formerly thought to be RNase BN |
| CDS | contig00021 | 48284 | 46473 | -2 | - | 1812 | GTP-binding protein TypA/BipA |
| CDS | contig00021 | 48635 | 50044 | 2 | + | 1410 | Glutamine synthetase type I (EC 6.3.1.2) |
| CDS | contig00021 | 50237 | 50806 | 2 | + | 570 | FIG01200260: hypothetical protein |
| CDS | contig00021 | 50893 | 51948 | 1 | + | 1056 | Nitrogen regulation protein NtrB (EC 2.7.13.3) |
| CDS | contig00021 | 51958 | 53376 | 1 | + | 1419 | Nitrogen regulation protein NtrC |
| CDS | contig00021 | 53518 | 54519 | 1 | + | 1002 | Adenosine deaminase (EC 3.5.4.4) |
| CDS | contig00021 | 55961 | 54588 | -2 | - | 1374 | Coproporphyrinogen III oxidase, oxygen-independent (EC 1.3.99.22) |
| CDS | contig00021 | 56430 | 55990 | -3 | - | 441 | Periplasmic/membrane protein associated with DUF414 |
| CDS | contig00021 | 57023 | 56442 | -2 | - | 582 | Protein of unknown function DUF414 |
| CDS | contig00021 | 58393 | 57824 | -1 | - | 570 | Cytochrome c4 |
| CDS | contig00021 | 58585 | 59238 | 1 | + | 654 | GTP-binding protein EngB |
| CDS | contig00021 | 61040 | 60219 | -2 | - | 822 | Shikimate 5-dehydrogenase I alpha (EC 1.1.1.25) |
| CDS | contig00021 | 62082 | 61144 | -3 | - | 939 | Coproporphyrinogen III oxidase, aerobic (EC 1.3.3.3) |
| CDS | contig00021 | 62713 | 62153 | -1 | - | 561 | TsaC protein (YrdC domain) required for threonylcarbamoyladenosine t(6)A37 modification in tRNA |
| CDS | contig00021 | 63549 | 62992 | -3 | - | 558 | Similar to C-terminal Zn-finger domain of DNA topoisomerase I |
| CDS | contig00021 | 64078 | 63605 | -1 | - | 474 | Protein of unknown function Smg |
| CDS | contig00021 | 65165 | 64071 | -2 | - | 1095 | Rossmann fold nucleotide-binding protein Smf possibly involved in DNA uptake |
| CDS | contig00021 | 66276 | 65188 | -3 | - | 1089 | Uncharacterized protein with LysM domain, COG1652 |
| CDS | contig00021 | 66385 | 66897 | 1 | + | 513 | Peptide deformylase (EC 3.5.1.88) |
| CDS | contig00021 | 66910 | 67854 | 1 | + | 945 | Methionyl-tRNA formyltransferase (EC 2.1.2.9) |
| CDS | contig00021 | 67915 | 69207 | 1 | + | 1293 | Ribosomal RNA small subunit methyltransferase B (EC 2.1.1.-) |
| CDS | contig00021 | 69282 | 70658 | 3 | + | 1377 | Trk system potassium uptake protein TrkA |
| CDS | contig00021 | 70752 | 72194 | 3 | + | 1443 | Potassium uptake protein TrkH |
| CDS | contig00021 | 73608 | 72277 | -3 | - | 1332 | DNA recombination protein RmuC |
| CDS | contig00021 | 73820 | 73605 | -2 | - | 216 | FIG00361587: hypothetical protein |
| CDS | contig00021 | 73958 | 74470 | 2 | + | 513 | Ferredoxin-type protein NapF (periplasmic nitrate reductase) |
| CDS | contig00021 | 74975 | 74523 | -2 | - | 453 | Nitrite-sensitive transcriptional repressor NsrR |
| CDS | contig00021 | 76090 | 75251 | -1 | - | 840 | Carbohydrate kinase, PfkB family |
| CDS | contig00021 | 77122 | 76151 | -1 | - | 972 | Cys regulon transcriptional activator CysB |
| CDS | contig00021 | 78004 | 77276 | -1 | - | 729 | GMP synthase (EC 6.3.5.2) |
| CDS | contig00021 | 79605 | 78088 | -3 | - | 1518 | Methylmalonate-semialdehyde dehydrogenase [inositol] (EC 1.2.1.27) |
| CDS | contig00021 | 81010 | 79682 | -1 | - | 1329 | Omega-amino acid--pyruvate aminotransferase (EC 2.6.1.18) |
| CDS | contig00021 | 81182 | 81724 | 2 | + | 543 | Transcriptional regulator, MerR family |
| CDS | contig00021 | 81976 | 83472 | 1 | + | 1497 | Aldehyde dehydrogenase (EC 1.2.1.3) |
| CDS | contig00021 | 83597 | 83770 | 2 | + | 174 | hypothetical protein |
| CDS | contig00021 | 83893 | 84789 | 1 | + | 897 | Protein rarD |
| CDS | contig00021 | 84846 | 85649 | 3 | + | 804 | Transcriptional regulator, AraC family |
| CDS | contig00021 | 85735 | 86373 | 1 | + | 639 | Threonine efflux protein |
| CDS | contig00021 | 87092 | 86427 | -2 | - | 666 | hypothetical protein |
| CDS | contig00021 | 87291 | 87734 | 3 | + | 444 | Uncharacterized conserved protein |
| CDS | contig00021 | 87910 | 88221 | 1 | + | 312 | hypothetical protein |
| CDS | contig00021 | 89000 | 88500 | -2 | - | 501 | hypothetical protein |
| CDS | contig00021 | 89157 | 89354 | 3 | + | 198 | FIG00362285: hypothetical protein |
| CDS | contig00021 | 89801 | 89460 | -2 | - | 342 | hypothetical protein |
| CDS | contig00021 | 92321 | 90150 | -2 | - | 2172 | ATP-dependent DNA helicase UvrD/PcrA |
| CDS | contig00021 | 92734 | 93732 | 1 | + | 999 | Predicted nucleoside ABC transporter, substrate-binding component |
| CDS | contig00021 | 93774 | 95411 | 3 | + | 1638 | Predicted nucleoside ABC transporter, ATP-binding component |
| CDS | contig00021 | 95491 | 96609 | 1 | + | 1119 | Predicted nucleoside ABC transporter, permease 1 component |
| CDS | contig00021 | 96612 | 97577 | 3 | + | 966 | Predicted nucleoside ABC transporter, permease 2 component |
| CDS | contig00021 | 98546 | 97641 | -2 | - | 906 | Transcriptional regulator, LysR family, in formaldehyde detoxification operon |
| CDS | contig00021 | 98665 | 99792 | 1 | + | 1128 | S-(hydroxymethyl)glutathione dehydrogenase (EC 1.1.1.284) |
| CDS | contig00021 | 99841 | 100689 | 1 | + | 849 | S-formylglutathione hydrolase (EC 3.1.2.12) |
| CDS | contig00021 | 100753 | 101259 | 1 | + | 507 | hypothetical protein |
| CDS | contig00021 | 102054 | 101332 | -3 | - | 723 | hypothetical protein |
| CDS | contig00021 | 102353 | 104500 | 2 | + | 2148 | Glutamate synthase [NADPH] large chain (EC 1.4.1.13) |
| CDS | contig00021 | 105529 | 104558 | -1 | - | 972 | Putative membrane protein precursor |
| CDS | contig00021 | 106585 | 105803 | -1 | - | 783 | FIG00553873: hypothetical protein |
| CDS | contig00021 | 107260 | 106628 | -1 | - | 633 | FIG00553873: hypothetical protein |
| CDS | contig00021 | 107753 | 107415 | -2 | - | 339 | tRNA-binding protein YgjH |
| CDS | contig00021 | 109261 | 107816 | -1 | - | 1446 | FIG00553873: hypothetical protein |
| CDS | contig00021 | 109818 | 109363 | -3 | - | 456 | Evolved beta-D-galactosidase, beta subunit |
| CDS | contig00021 | 112901 | 109815 | -2 | - | 3087 | Evolved beta-D-galactosidase, alpha subunit |
| CDS | contig00021 | 114179 | 113223 | -2 | - | 957 | Evolved beta-D-galactosidase transcriptional repressor |
| CDS | contig00022 | 678 | 1280 | 3 | + | 603 | Recombination protein RecR |
| CDS | contig00022 | 1820 | 3733 | 2 | + | 1914 | Chaperone protein HtpG |
| CDS | contig00022 | 3973 | 4617 | 1 | + | 645 | Adenylate kinase (EC 2.7.4.3) |
| CDS | contig00022 | 4737 | 5711 | 3 | + | 975 | Ferrochelatase, protoheme ferro-lyase (EC 4.99.1.1) |
| CDS | contig00022 | 5776 | 6168 | 1 | + | 393 | probable membrane protein STY2112 |
| CDS | contig00022 | 6376 | 6260 | -1 | - | 117 | hypothetical protein |
| CDS | contig00022 | 6344 | 7648 | 2 | + | 1305 | Inosine-guanosine kinase (EC 2.7.1.73) |
| CDS | contig00022 | 7811 | 9439 | 2 | + | 1629 | Methyl-accepting chemotaxis protein |
| CDS | contig00022 | 9562 | 9924 | 1 | + | 363 | FIG00361671: hypothetical protein |
| CDS | contig00022 | 10071 | 10565 | 3 | + | 495 | hypothetical protein |
| CDS | contig00022 | 12189 | 10528 | -3 | - | 1662 | GGDEF domain protein |
| CDS | contig00022 | 12408 | 13628 | 3 | + | 1221 | Glucose-1-phosphate adenylyltransferase (EC 2.7.7.27) |
| CDS | contig00022 | 15687 | 13687 | -3 | - | 2001 | FIG00919923: hypothetical protein |
| CDS | contig00022 | 16385 | 15834 | -2 | - | 552 | Uncharacterized lipoprotein yceB precursor |
| CDS | contig00022 | 16721 | 17893 | 2 | + | 1173 | Isochorismate synthase (EC 5.4.4.2) of siderophore biosynthesis |
| CDS | contig00022 | 17890 | 19557 | 1 | + | 1668 | 2,3-dihydroxybenzoate-AMP ligase (EC 2.7.7.58) of siderophore biosynthesis |
| CDS | contig00022 | 19581 | 20489 | 3 | + | 909 | Isochorismatase (EC 3.3.2.1) of siderophore biosynthesis |
| CDS | contig00022 | 20486 | 23587 | 2 | + | 3102 | Siderophore biosynthesis non-ribosomal peptide synthetase modules |
| CDS | contig00022 | 23608 | 24357 | 1 | + | 750 | 2,3-dihydro-2,3-dihydroxybenzoate dehydrogenase (EC 1.3.1.28) of siderophore biosynthesis |
| CDS | contig00022 | 24372 | 30635 | 3 | + | 6264 | Siderophore biosynthesis non-ribosomal peptide synthetase modules |
| CDS | contig00022 | 30632 | 32176 | 2 | + | 1545 | Peptide synthetase |
| CDS | contig00022 | 33200 | 32262 | -2 | - | 939 | Iron(III) dicitrate transport system, periplasmic iron-binding protein FecB (TC 3.A.1.14.1) |
| CDS | contig00022 | 33408 | 34139 | 3 | + | 732 | 4&#39;-phosphopantetheinyl transferase entD (EC 2.7.8.-) |
| CDS | contig00022 | 35048 | 34230 | -2 | - | 819 | Iron(III) dicitrate transport ATP-binding protein FecE (TC 3.A.1.14.1) |
| CDS | contig00022 | 36141 | 35092 | -3 | - | 1050 | ABC-type Fe3+-siderophore transport system, permease component |
| CDS | contig00022 | 37170 | 36154 | -3 | - | 1017 | ABC-type Fe3+-siderophore transport system, permease component |
| CDS | contig00022 | 39222 | 37249 | -3 | - | 1974 | Colicin I receptor precursor |
| CDS | contig00022 | 40518 | 39283 | -3 | - | 1236 | FIG00904545: hypothetical protein |
| CDS | contig00022 | 40734 | 41483 | 3 | + | 750 | Type II and III secretion system family protein |
| CDS | contig00022 | 41954 | 41493 | -2 | - | 462 | Arginine/ornithine antiporter ArcD |
| CDS | contig00022 | 43046 | 42099 | -2 | - | 948 | Magnesium and cobalt transport protein CorA |
| CDS | contig00022 | 45090 | 43462 | -3 | - | 1629 | Electron transfer flavoprotein-ubiquinone oxidoreductase (EC 1.5.5.1) |
| CDS | contig00022 | 45232 | 45981 | 1 | + | 750 | Electron transfer flavoprotein, beta subunit |
| CDS | contig00022 | 46072 | 45956 | -1 | - | 117 | hypothetical protein |
| CDS | contig00022 | 46097 | 47014 | 2 | + | 918 | Electron transfer flavoprotein, alpha subunit |
| CDS | contig00022 | 47239 | 48327 | 1 | + | 1089 | Phosphoserine aminotransferase (EC 2.6.1.52) |
| CDS | contig00022 | 48498 | 49778 | 3 | + | 1281 | 5-Enolpyruvylshikimate-3-phosphate synthase (EC 2.5.1.19) |
| CDS | contig00022 | 49975 | 51030 | 1 | + | 1056 | 2-keto-3-deoxy-D-arabino-heptulosonate-7-phosphate synthase I alpha (EC 2.5.1.54) |
| CDS | contig00022 | 51839 | 51117 | -2 | - | 723 | Integral membrane protein TerC |
| CDS | contig00022 | 51956 | 53071 | 2 | + | 1116 | ATP-NAD kinase (EC 2.7.1.23) |
| CDS | contig00022 | 53179 | 53457 | 1 | + | 279 | FIG00361411: hypothetical protein |
| CDS | contig00022 | 53557 | 55221 | 1 | + | 1665 | Long-chain-fatty-acid--CoA ligase (EC 6.2.1.3) |
| CDS | contig00022 | 56708 | 55827 | -2 | - | 882 | Cytidine deaminase (EC 3.5.4.5) |
| CDS | contig00022 | 56703 | 56888 | 3 | + | 186 | hypothetical protein |
| CDS | contig00022 | 57621 | 56935 | -3 | - | 687 | LrgA-associated membrane protein LrgB |
| CDS | contig00022 | 57977 | 57618 | -2 | - | 360 | Antiholin-like protein LrgA |
| CDS | contig00022 | 58108 | 59823 | 1 | + | 1716 | putative alpha-1,6-galactosidase |
| CDS | contig00022 | 60388 | 59882 | -1 | - | 507 | hypothetical protein |
| CDS | contig00022 | 61964 | 60537 | -2 | - | 1428 | Exodeoxyribonuclease I (EC 3.1.11.1) |
| CDS | contig00022 | 62853 | 62143 | -3 | - | 711 | Serine/threonine protein phosphatase (EC 3.1.3.16) |
| CDS | contig00022 | 63073 | 65628 | 1 | + | 2556 | Conserved protein |
| CDS | contig00022 | 65639 | 66352 | 2 | + | 714 | virulence factor family protein |
| CDS | contig00022 | 67214 | 66444 | -2 | - | 771 | Lysine-arginine-ornithine-binding periplasmic protein precursor (TC 3.A.1.3.1) |
| CDS | contig00022 | 68053 | 67268 | -1 | - | 786 | FIG003879: Predicted amidohydrolase |
| CDS | contig00022 | 69243 | 68092 | -3 | - | 1152 | Aspartate aminotransferase (EC 2.6.1.1) |
| CDS | contig00022 | 69359 | 70249 | 2 | + | 891 | Glycine cleavage system transcriptional activator |
| CDS | contig00022 | 71820 | 70321 | -3 | - | 1500 | L-arabinose isomerase (EC 5.3.1.4) |
| CDS | contig00022 | 72533 | 71835 | -2 | - | 699 | L-ribulose-5-phosphate 4-epimerase (EC 5.1.3.4) |
| CDS | contig00022 | 74206 | 72530 | -1 | - | 1677 | Ribulokinase (EC 2.7.1.16) |
| CDS | contig00022 | 74567 | 75550 | 2 | + | 984 | L-arabinose-binding periplasmic protein precursor AraF (TC 3.A.1.2.2) |
| CDS | contig00022 | 75617 | 77113 | 2 | + | 1497 | L-arabinose transport ATP-binding protein AraG (TC 3.A.1.2.2) |
| CDS | contig00022 | 77162 | 78163 | 2 | + | 1002 | L-arabinose transport system permease protein (TC 3.A.1.2.2) |
| CDS | contig00022 | 78296 | 79207 | 2 | + | 912 | Arabinose operon regulatory protein |
| CDS | contig00022 | 79401 | 79276 | -3 | - | 126 | LSU ribosomal protein L36p |
| CDS | contig00022 | 79667 | 79398 | -2 | - | 270 | LSU ribosomal protein L31p |
| CDS | contig00022 | 79893 | 80147 | 3 | + | 255 | DNA-damage-inducible protein I |
| CDS | contig00022 | 81127 | 80378 | -1 | - | 750 | FIG00786704: hypothetical protein |
| CDS | contig00022 | 81714 | 81211 | -3 | - | 504 | COG1451: Predicted metal-dependent hydrolase |
| CDS | contig00022 | 83489 | 81846 | -2 | - | 1644 | FIG00362448: hypothetical protein |
| CDS | contig00022 | 85418 | 83769 | -2 | - | 1650 | 5&#39;-nucleotidase (EC 3.1.3.5) |
| CDS | contig00022 | 86861 | 85509 | -2 | - | 1353 | C4-dicarboxylate transporter DcuC (TC 2.A.61.1.1) |
| CDS | contig00022 | 87185 | 87790 | 2 | + | 606 | Sll1761 protein |
| CDS | contig00022 | 89675 | 87909 | -2 | - | 1767 | 5-methylaminomethyl-2-thiouridine-forming enzyme mnmC |
| CDS | contig00022 | 90027 | 91226 | 3 | + | 1200 | 3-oxoacyl-[acyl-carrier-protein] synthase, KASI (EC 2.3.1.41) |
| CDS | contig00022 | 91820 | 91296 | -2 | - | 525 | GCN5-related N-acetyltransferase |
| CDS | contig00022 | 92232 | 91918 | -3 | - | 315 | putative lipoprotein |
| CDS | contig00022 | 93612 | 92326 | -3 | - | 1287 | Citrate synthase (si) (EC 2.3.3.1) |
| CDS | contig00022 | 94153 | 94425 | 1 | + | 273 | Succinate dehydrogenase cytochrome b-556 subunit |
| CDS | contig00022 | 94419 | 94763 | 3 | + | 345 | Succinate dehydrogenase hydrophobic membrane anchor protein |
| CDS | contig00022 | 94765 | 96531 | 1 | + | 1767 | Succinate dehydrogenase flavoprotein subunit (EC 1.3.99.1) |
| CDS | contig00022 | 96606 | 97265 | 3 | + | 660 | Succinate dehydrogenase iron-sulfur protein (EC 1.3.99.1) |
| CDS | contig00022 | 97380 | 100190 | 3 | + | 2811 | 2-oxoglutarate dehydrogenase E1 component (EC 1.2.4.2) |
| CDS | contig00022 | 100279 | 101472 | 1 | + | 1194 | Dihydrolipoamide succinyltransferase component (E2) of 2-oxoglutarate dehydrogenase complex (EC 2.3.1.61) |
| CDS | contig00022 | 101581 | 102747 | 1 | + | 1167 | Succinyl-CoA ligase [ADP-forming] beta chain (EC 6.2.1.5) |
| CDS | contig00022 | 102747 | 103619 | 3 | + | 873 | Succinyl-CoA ligase [ADP-forming] alpha chain (EC 6.2.1.5) |
| CDS | contig00024 | 185 | 340 | 2 | + | 156 | Mobile element protein |
| CDS | contig00025 | 586 | 1779 | 1 | + | 1194 | Short-chain alcohol dehydrogenase family |
| CDS | contig00025 | 1942 | 2619 | 1 | + | 678 | Arginine/ornithine antiporter ArcD |
| CDS | contig00025 | 2616 | 3416 | 3 | + | 801 | Arginine/ornithine antiporter ArcD |
| CDS | contig00025 | 4696 | 3482 | -1 | - | 1215 | Aspartate aminotransferase (EC 2.6.1.1) |
| CDS | contig00025 | 4673 | 4786 | 2 | + | 114 | hypothetical protein |
| CDS | contig00025 | 5053 | 5262 | 1 | + | 210 | FIG00361672: hypothetical protein |
| CDS | contig00025 | 5579 | 5322 | -2 | - | 258 | Glutaredoxin 1 |
| CDS | contig00025 | 7230 | 5797 | -3 | - | 1434 | D-alanyl-D-alanine carboxypeptidase (EC 3.4.16.4) |
| CDS | contig00025 | 9078 | 7318 | -3 | - | 1761 | ATP-dependent RNA helicase YejH |
| CDS | contig00025 | 10236 | 9199 | -3 | - | 1038 | Probable Co/Zn/Cd efflux system membrane fusion protein |
| CDS | contig00025 | 10579 | 10247 | -1 | - | 333 | GTPase (EC 3.6.1.-) |
| CDS | contig00025 | 10787 | 12439 | 2 | + | 1653 | FIG00732166: hypothetical protein |
| CDS | contig00025 | 13923 | 12502 | -3 | - | 1422 | Carbon starvation protein A |
| CDS | contig00025 | 14772 | 14068 | -3 | - | 705 | FIG001014_Response regulator of the LytR/AlgR family |
| CDS | contig00025 | 16460 | 14769 | -2 | - | 1692 | Autolysis histidine kinase LytS |
| CDS | contig00025 | 18048 | 16813 | -3 | - | 1236 | HD-GYP domain |
| CDS | contig00025 | 20834 | 18159 | -2 | - | 2676 | Alcohol dehydrogenase (EC 1.1.1.1); Acetaldehyde dehydrogenase (EC 1.2.1.10) |
| CDS | contig00025 | 21666 | 22301 | 3 | + | 636 | Putative membrane protein |
| CDS | contig00025 | 22555 | 24201 | 1 | + | 1647 | Oligopeptide ABC transporter, periplasmic oligopeptide-binding protein OppA (TC 3.A.1.5.1) |
| CDS | contig00025 | 24299 | 25219 | 2 | + | 921 | Oligopeptide transport system permease protein OppB (TC 3.A.1.5.1) |
| CDS | contig00025 | 25234 | 26145 | 1 | + | 912 | Oligopeptide transport system permease protein OppC (TC 3.A.1.5.1) |
| CDS | contig00025 | 26254 | 27234 | 1 | + | 981 | Oligopeptide transport ATP-binding protein OppD (TC 3.A.1.5.1) |
| CDS | contig00025 | 27235 | 28230 | 1 | + | 996 | Oligopeptide transport ATP-binding protein OppF (TC 3.A.1.5.1) |
| CDS | contig00025 | 28480 | 28367 | -1 | - | 114 | hypothetical protein |
| CDS | contig00025 | 28523 | 29467 | 2 | + | 945 | Integral membrane protein TerC |
| CDS | contig00025 | 30751 | 29525 | -1 | - | 1227 | Arginine racemase (EC 5.1.1.9) @ Lysine racemase (EC 5.1.1.5) @ Ornithine racemase (EC 5.1.1.12) |
| CDS | contig00025 | 30931 | 31377 | 1 | + | 447 | Putative protein-S-isoprenylcysteine methyltransferase |
| CDS | contig00025 | 32710 | 31451 | -1 | - | 1260 | Sodium:dicarboxylate symporter |
| CDS | contig00025 | 33390 | 32914 | -3 | - | 477 | hypothetical protein |
| CDS | contig00025 | 35268 | 33925 | -3 | - | 1344 | Dihydroorotase (EC 3.5.2.3) |
| CDS | contig00025 | 35348 | 36232 | 2 | + | 885 | NADPH dependent preQ0 reductase (EC 1.7.1.13) |
| CDS | contig00025 | 38303 | 36303 | -2 | - | 2001 | Methyl-accepting chemotaxis protein I (serine chemoreceptor protein) |
| CDS | contig00025 | 38572 | 38922 | 1 | + | 351 | YcfF/hinT protein: a purine nucleoside phosphoramidase |
| CDS | contig00025 | 38922 | 40343 | 3 | + | 1422 | hypothetical protein |
| CDS | contig00025 | 40412 | 40810 | 2 | + | 399 | YcfL protein: an outer membrane lipoprotein that is part of a salvage cluster |
| CDS | contig00025 | 40810 | 41406 | 1 | + | 597 | Lipoprotein YcfM, part of a salvage pathway of unknown substrate |
| CDS | contig00025 | 41396 | 42259 | 2 | + | 864 | Thiamine kinase (EC 2.7.1.89) |
| CDS | contig00025 | 42311 | 43171 | 2 | + | 861 | hypothetical protein |
| CDS | contig00025 | 43279 | 43746 | 1 | + | 468 | hypothetical protein |
| CDS | contig00025 | 43826 | 45697 | 2 | + | 1872 | Protein-export membrane protein SecD (TC 3.A.5.1.1) |
| CDS | contig00025 | 45807 | 46721 | 3 | + | 915 | Protein-export membrane protein SecF (TC 3.A.5.1.1) |
| CDS | contig00025 | 46972 | 48591 | 1 | + | 1620 | methyl-accepting chemotaxis protein |
| CDS | contig00025 | 49248 | 48646 | -3 | - | 603 | FIG027190: Putative transmembrane protein |
| CDS | contig00025 | 49455 | 50366 | 3 | + | 912 | Membrane protein, putative |
| CDS | contig00025 | 50536 | 52326 | 1 | + | 1791 | Oligoendopeptidase F (EC 3.4.24.-) |
| CDS | contig00025 | 52405 | 54213 | 1 | + | 1809 | Oligoendopeptidase F (EC 3.4.24.-) |
| CDS | contig00025 | 54398 | 54937 | 2 | + | 540 | YcfP protein: probably an esterase that is part of a salvage cluster |
| CDS | contig00025 | 55012 | 56304 | 1 | + | 1293 | NADH dehydrogenase (EC 1.6.99.3) |
| CDS | contig00025 | 56684 | 57001 | 2 | + | 318 | FIG00361931: hypothetical protein |
| CDS | contig00025 | 59751 | 57481 | -3 | - | 2271 | Putative cyclic beta-1,2-glucan modification protein |
| CDS | contig00025 | 60003 | 61097 | 3 | + | 1095 | FIG00361789: hypothetical protein |
| CDS | contig00025 | 62565 | 61213 | -3 | - | 1353 | Aspartokinase (EC 2.7.2.4) |
| CDS | contig00025 | 64315 | 62987 | -1 | - | 1329 | N-acetylglutamate synthase (EC 2.3.1.1) |
| CDS | contig00025 | 64568 | 65200 | 2 | + | 633 | Peptide methionine sulfoxide reductase MsrA (EC 1.8.4.11) |
| CDS | contig00025 | 65839 | 65504 | -1 | - | 336 | Type IIA topoisomerase, B subunit |
| CDS | contig00025 | 66379 | 65900 | -1 | - | 480 | acetyltransferase, GNAT family |
| CDS | contig00025 | 66754 | 66383 | -1 | - | 372 | MmcQ |
| CDS | contig00025 | 66861 | 67655 | 3 | + | 795 | Beta-ketoadipate enol-lactone hydrolase, putative |
| CDS | contig00025 | 68576 | 67671 | -2 | - | 906 | Predicted Lactate-responsive regulator, LysR family |
| CDS | contig00025 | 68707 | 70407 | 1 | + | 1701 | L-lactate permease |
| CDS | contig00025 | 70420 | 71241 | 1 | + | 822 | Predicted L-lactate dehydrogenase, Fe-S oxidoreductase subunit YkgE |
| CDS | contig00025 | 71234 | 72670 | 2 | + | 1437 | Predicted L-lactate dehydrogenase, Iron-sulfur cluster-binding subunit YkgF |
| CDS | contig00025 | 72672 | 73316 | 3 | + | 645 | Predicted L-lactate dehydrogenase, hypothetical protein subunit YkgG |
| CDS | contig00025 | 73316 | 76108 | 2 | + | 2793 | Predicted D-lactate dehydrogenase, Fe-S protein, FAD/FMN-containing |
| CDS | contig00025 | 77893 | 76805 | -1 | - | 1089 | AttH component of AttEFGH ABC transport system |
| CDS | contig00025 | 80457 | 78031 | -3 | - | 2427 | AttF component of AttEFGH ABC transport system / AttG component of AttEFGH ABC transport system |
| CDS | contig00025 | 81159 | 80479 | -3 | - | 681 | AttE component of AttEFGH ABC transport system |
| CDS | contig00025 | 81312 | 81785 | 3 | + | 474 | FIG00362043: hypothetical protein |
| CDS | contig00025 | 81921 | 83345 | 3 | + | 1425 | Na+/H+ antiporter NhaD type |
| CDS | contig00025 | 83379 | 83945 | 3 | + | 567 | FIG01199753: hypothetical protein |
| CDS | contig00025 | 85201 | 84005 | -1 | - | 1197 | MFS family multidrug transport protein, bicyclomycin resistance protein |
| CDS | contig00025 | 86942 | 85254 | -2 | - | 1689 | membrane protein, putative |
| CDS | contig00025 | 88280 | 87012 | -2 | - | 1269 | FIG00921296: hypothetical protein |
| CDS | contig00025 | 89035 | 88475 | -1 | - | 561 | FIG00921362: hypothetical protein |
| CDS | contig00025 | 90507 | 89083 | -3 | - | 1425 | FIG00919982: hypothetical protein |
| CDS | contig00025 | 91422 | 90583 | -3 | - | 840 | Cytochrome C553 (soluble cytochrome f) |
| CDS | contig00025 | 94382 | 91422 | -2 | - | 2961 | FIG00919891: hypothetical protein |
| CDS | contig00025 | 94836 | 94387 | -3 | - | 450 | FIG00920227: hypothetical protein |
| CDS | contig00025 | 95440 | 94955 | -1 | - | 486 | Putative membrane protein |
| CDS | contig00025 | 96272 | 95523 | -2 | - | 750 | FIG00441227: hypothetical protein |
| CDS | contig00025 | 97056 | 96256 | -3 | - | 801 | Protein containing transglutaminase-like domain, putative cysteine protease |
| CDS | contig00025 | 97979 | 97053 | -2 | - | 927 | Protein containing domains DUF403 |
| CDS | contig00025 | 99409 | 97973 | -1 | - | 1437 | Protein containing domains DUF404, DUF407 |
| CDS | contig00025 | 100347 | 102056 | 3 | + | 1710 | Putative sulfate permease |
| CDS | contig00026 | 425 | 634 | 2 | + | 210 | CFA/I fimbrial minor adhesin |
| CDS | contig00026 | 649 | 1323 | 1 | + | 675 | CFA/I fimbrial chaperone |
| CDS | contig00026 | 1996 | 1358 | -1 | - | 639 | Putative transport protein |
| CDS | contig00026 | 2208 | 3428 | 3 | + | 1221 | Fosmidomycin resistance protein |
| CDS | contig00026 | 4838 | 3654 | -2 | - | 1185 | NAD(FAD)-utilizing dehydrogenases |
| CDS | contig00026 | 5525 | 5001 | -2 | - | 525 | Ferritin-like protein 2 |
| CDS | contig00026 | 5735 | 7519 | 2 | + | 1785 | hypothetical protein |
| CDS | contig00026 | 8724 | 7537 | -3 | - | 1188 | Uncharacterized paraquat-inducible protein B |
| CDS | contig00026 | 9177 | 9611 | 3 | + | 435 | Universal stress protein A |
| CDS | contig00026 | 9803 | 10816 | 2 | + | 1014 | Tryptophanyl-tRNA synthetase (EC 6.1.1.2) |
| CDS | contig00026 | 11769 | 10795 | -3 | - | 975 | Transcriptional regulator, LysR family |
| CDS | contig00026 | 11861 | 12760 | 2 | + | 900 | Permease of the drug/metabolite transporter (DMT) superfamily |
| CDS | contig00026 | 12745 | 12906 | 1 | + | 162 | FIG00362267: hypothetical protein |
| CDS | contig00026 | 13485 | 13012 | -3 | - | 474 | hypothetical protein |
| CDS | contig00026 | 13525 | 14379 | 1 | + | 855 | Putative acetate efflux pump, MadN |
| CDS | contig00026 | 15249 | 14443 | -3 | - | 807 | diguanylate cyclase/phosphodiesterase (GGDEF & EAL domains) with PAS/PAC sensor(s) |
| CDS | contig00026 | 16190 | 15477 | -2 | - | 714 | 3-deoxy-D-manno-octulosonic acid kinase (EC 2.7.1.-) |
| CDS | contig00026 | 16329 | 17429 | 3 | + | 1101 | ADP-heptose--lipooligosaccharide heptosyltransferase II (EC 2.4.1.-) |
| CDS | contig00026 | 17686 | 18312 | 1 | + | 627 | Guanylate kinase (EC 2.7.4.8) |
| CDS | contig00026 | 18392 | 18667 | 2 | + | 276 | DNA-directed RNA polymerase omega subunit (EC 2.7.7.6) |
| CDS | contig00026 | 18803 | 20920 | 2 | + | 2118 | GTP pyrophosphokinase (EC 2.7.6.5) / Guanosine-3&#39;,5&#39;-bis(diphosphate) 3&#39;-pyrophosphohydrolase (EC 3.1.7.2) |
| CDS | contig00026 | 21028 | 21759 | 1 | + | 732 | tRNA (guanosine(18)-2&#39;-O)-methyltransferase (EC 2.1.1.34) |
| CDS | contig00026 | 21759 | 22424 | 3 | + | 666 | N-acetylglutamate synthase (EC 2.3.1.1) |
| CDS | contig00026 | 22421 | 23461 | 2 | + | 1041 | Lysophospholipase L2 (EC 3.1.1.5) |
| CDS | contig00026 | 23565 | 24368 | 3 | + | 804 | HMP-PP hydrolase (pyridoxal phosphatase) Cof, detected in genetic screen for thiamin metabolic genes (PMID:15292217) |
| CDS | contig00026 | 24957 | 24439 | -3 | - | 519 | hypothetical protein |
| CDS | contig00026 | 25835 | 26425 | 2 | + | 591 | Transcriptional regulator, TetR family |
| CDS | contig00026 | 26422 | 27528 | 1 | + | 1107 | Probable Co/Zn/Cd efflux system membrane fusion protein |
| CDS | contig00026 | 27544 | 30624 | 1 | + | 3081 | RND multidrug efflux transporter; Acriflavin resistance protein |
| CDS | contig00026 | 30746 | 32704 | 2 | + | 1959 | Signal transduction histidine kinase |
| CDS | contig00026 | 32781 | 34130 | 3 | + | 1350 | Glutathione reductase (EC 1.8.1.7) |
| CDS | contig00026 | 34480 | 36162 | 1 | + | 1683 | Potassium-transporting ATPase A chain (EC 3.6.3.12) (TC 3.A.3.7.1) |
| CDS | contig00026 | 36236 | 38275 | 2 | + | 2040 | Potassium-transporting ATPase B chain (EC 3.6.3.12) (TC 3.A.3.7.1) |
| CDS | contig00026 | 38336 | 38863 | 2 | + | 528 | Potassium-transporting ATPase C chain (EC 3.6.3.12) (TC 3.A.3.7.1) |
| CDS | contig00026 | 38966 | 41554 | 2 | + | 2589 | Osmosensitive K+ channel histidine kinase KdpD (EC 2.7.3.-) |
| CDS | contig00026 | 41593 | 42276 | 1 | + | 684 | sensor histidine kinase |
| CDS | contig00026 | 43408 | 42281 | -1 | - | 1128 | Multidrug resistance protein D |
| CDS | contig00026 | 43501 | 44325 | 1 | + | 825 | Transcriptional regulator, AraC family |
| CDS | contig00026 | 44772 | 46022 | 3 | + | 1251 | Valine--pyruvate aminotransferase (EC 2.6.1.66) |
| CDS | contig00026 | 46535 | 46095 | -2 | - | 441 | 16 kDa heat shock protein A |
| CDS | contig00026 | 47041 | 46628 | -1 | - | 414 | 16 kDa heat shock protein A |
| CDS | contig00026 | 47298 | 48068 | 3 | + | 771 | Beta-lactamase-like |
| CDS | contig00026 | 48154 | 48540 | 1 | + | 387 | Transcriptional regulator containing an amidase domain and an AraC-type DNA-binding HTH domain |
| CDS | contig00026 | 48577 | 49119 | 1 | + | 543 | Transcriptional regulator containing an amidase domain and an AraC-type DNA-binding HTH domain |
| CDS | contig00026 | 51630 | 49219 | -3 | - | 2412 | DNA gyrase subunit B (EC 5.99.1.3) |
| CDS | contig00026 | 52730 | 51627 | -2 | - | 1104 | DNA recombination and repair protein RecF |
| CDS | contig00026 | 53836 | 52733 | -1 | - | 1104 | DNA polymerase III beta subunit (EC 2.7.7.7) |
| CDS | contig00026 | 55290 | 53920 | -3 | - | 1371 | Chromosomal replication initiator protein DnaA |
| CDS | contig00026 | 55601 | 56359 | 2 | + | 759 | Amino acid ABC transporter, periplasmic amino acid-binding portion |
| CDS | contig00026 | 56360 | 57028 | 2 | + | 669 | Amino acid ABC transporter, permease protein |
| CDS | contig00026 | 57041 | 57775 | 2 | + | 735 | ABC-type polar amino acid transport system, ATPase component |
| CDS | contig00026 | 58045 | 58179 | 1 | + | 135 | LSU ribosomal protein L34p |
| CDS | contig00026 | 58332 | 58538 | 3 | + | 207 | Ribonuclease P protein component (EC 3.1.26.5) |
| CDS | contig00026 | 58759 | 60405 | 1 | + | 1647 | Inner membrane protein translocase component YidC, long form |
| CDS | contig00026 | 60467 | 61876 | 2 | + | 1410 | GTPase and tRNA-U34 5-formylation enzyme TrmE |
| CDS | contig00026 | 62039 | 62335 | 2 | + | 297 | Integrase |
| CDS | contig00026 | 63079 | 62351 | -1 | - | 729 | Mobile element protein |
| CDS | contig00026 | 63501 | 63208 | -3 | - | 294 | Mobile element protein |
| CDS | contig00026 | 64148 | 64402 | 2 | + | 255 | HipB protein |
| CDS | contig00026 | 64402 | 65721 | 1 | + | 1320 | HipA protein |
| CDS | contig00026 | 68122 | 66140 | -1 | - | 1983 | Ferric hydroxamate ABC transporter (TC 3.A.1.14.3), permease component FhuB |
| CDS | contig00026 | 69027 | 68119 | -3 | - | 909 | Ferric hydroxamate ABC transporter (TC 3.A.1.14.3), periplasmic substrate binding protein FhuD |
| CDS | contig00026 | 69817 | 69053 | -1 | - | 765 | Ferric hydroxamate ABC transporter (TC 3.A.1.14.3), ATP-binding protein FhuC |
| CDS | contig00026 | 71510 | 69864 | -2 | - | 1647 | Type I secretion system ATPase, LssB family LapB |
| CDS | contig00026 | 73679 | 71571 | -2 | - | 2109 | Ferric hydroxamate outer membrane receptor FhuA |
| CDS | contig00026 | 73883 | 74500 | 2 | + | 618 | Flavoprotein MioC |
| CDS | contig00026 | 75009 | 76898 | 3 | + | 1890 | tRNA uridine 5-carboxymethylaminomethyl modification enzyme GidA |
| CDS | contig00026 | 76910 | 77545 | 2 | + | 636 | rRNA small subunit 7-methylguanosine (m7G) methyltransferase GidB |
| CDS | contig00026 | 77573 | 78376 | 2 | + | 804 | Chromosome (plasmid) partitioning protein ParA |
| CDS | contig00026 | 78373 | 79281 | 1 | + | 909 | Chromosome (plasmid) partitioning protein ParB |
| CDS | contig00026 | 79487 | 79882 | 2 | + | 396 | FIG048548: ATP synthase protein I2 |
| CDS | contig00026 | 79891 | 80673 | 1 | + | 783 | ATP synthase F0 sector subunit a |
| CDS | contig00026 | 80732 | 80971 | 2 | + | 240 | ATP synthase F0 sector subunit c |
| CDS | contig00026 | 81023 | 81493 | 2 | + | 471 | ATP synthase F0 sector subunit b |
| CDS | contig00026 | 81507 | 82040 | 3 | + | 534 | ATP synthase delta chain (EC 3.6.3.14) |
| CDS | contig00026 | 82056 | 83597 | 3 | + | 1542 | ATP synthase alpha chain (EC 3.6.3.14) |
| CDS | contig00026 | 83640 | 84506 | 3 | + | 867 | ATP synthase gamma chain (EC 3.6.3.14) |
| CDS | contig00026 | 84540 | 85928 | 3 | + | 1389 | ATP synthase beta chain (EC 3.6.3.14) |
| CDS | contig00026 | 85947 | 86375 | 3 | + | 429 | ATP synthase epsilon chain (EC 3.6.3.14) |
| CDS | contig00026 | 86469 | 87830 | 3 | + | 1362 | N-acetylglucosamine-1-phosphate uridyltransferase (EC 2.7.7.23) / Glucosamine-1-phosphate N-acetyltransferase (EC 2.3.1.157) |
| CDS | contig00026 | 89273 | 87882 | -2 | - | 1392 | Inner membrane protein CreD |
| CDS | contig00026 | 90189 | 89395 | -3 | - | 795 | Beta-lactamase class D |
| CDS | contig00026 | 91737 | 90289 | -3 | - | 1449 | Two-component response regulator CreC |
| CDS | contig00026 | 92419 | 91745 | -1 | - | 675 | Two-component response regulator CreB |
| CDS | contig00026 | 92414 | 92545 | 2 | + | 132 | hypothetical protein |
| CDS | contig00026 | 92542 | 93315 | 1 | + | 774 | Transcriptional regulator of glmS gene, DeoR family |
| CDS | contig00026 | 93371 | 95203 | 2 | + | 1833 | Glucosamine--fructose-6-phosphate aminotransferase [isomerizing] (EC 2.6.1.16) |
| CDS | contig00026 | 96040 | 95264 | -1 | - | 777 | Transcriptional activator protein LuxR |
| CDS | contig00026 | 96219 | 97013 | 3 | + | 795 | TonB system biopolymer transport component; Chromosome segregation ATPase |
| CDS | contig00026 | 97010 | 98317 | 2 | + | 1308 | MotA/TolQ/ExbB proton channel family protein |
| CDS | contig00026 | 98314 | 98844 | 1 | + | 531 | Ferric siderophore transport system, biopolymer transport protein ExbB |
| CDS | contig00026 | 98841 | 99242 | 3 | + | 402 | Biopolymer transport protein ExbD/TolR |
| CDS | contig00026 | 99254 | 99901 | 2 | + | 648 | Ferric siderophore transport system, periplasmic binding protein TonB |
| CDS | contig00026 | 99904 | 101082 | 1 | + | 1179 | TPR domain protein, putative component of TonB system |
| CDS | contig00027 | 398 | 988 | 2 | + | 591 | TETRATRICOPEPTIDE REPEAT FAMILY PROTEIN |
| CDS | contig00027 | 1204 | 992 | -1 | - | 213 | hypothetical protein |
| CDS | contig00027 | 1530 | 3458 | 3 | + | 1929 | Threonyl-tRNA synthetase (EC 6.1.1.3) |
| CDS | contig00027 | 3603 | 4010 | 3 | + | 408 | Translation initiation factor 3 |
| CDS | contig00027 | 4097 | 4294 | 2 | + | 198 | LSU ribosomal protein L35p |
| CDS | contig00027 | 4310 | 4666 | 2 | + | 357 | LSU ribosomal protein L20p |
| CDS | contig00027 | 4973 | 5956 | 2 | + | 984 | Phenylalanyl-tRNA synthetase alpha chain (EC 6.1.1.20) |
| CDS | contig00027 | 5969 | 8356 | 2 | + | 2388 | Phenylalanyl-tRNA synthetase beta chain (EC 6.1.1.20) |
| CDS | contig00027 | 8360 | 8656 | 2 | + | 297 | Integration host factor alpha subunit |
| CDS | contig00027 | 8711 | 12634 | 2 | + | 3924 | histidine kinase/response regulator hybrid protein |
| CDS | contig00027 | 12636 | 13886 | 3 | + | 1251 | Response regulator |
| CDS | contig00027 | 13887 | 14501 | 3 | + | 615 | Acetyl-CoA synthetase (ADP-forming) alpha and beta chains, putative |
| CDS | contig00027 | 14574 | 15347 | 3 | + | 774 | NAD-dependent protein deacetylase of SIR2 family |
| CDS | contig00027 | 15454 | 15912 | 1 | + | 459 | hypothetical protein |
| CDS | contig00027 | 15909 | 16325 | 3 | + | 417 | GGDEF family protein |
| CDS | contig00027 | 16331 | 17557 | 2 | + | 1227 | GGDEF family protein |
| CDS | contig00027 | 18672 | 17647 | -3 | - | 1026 | Ribose operon repressor |
| CDS | contig00027 | 19604 | 18672 | -2 | - | 933 | Ribokinase (EC 2.7.1.15) |
| CDS | contig00027 | 20581 | 19703 | -1 | - | 879 | Ribose ABC transport system, periplasmic ribose-binding protein RbsB (TC 3.A.1.2.1) |
| CDS | contig00027 | 21577 | 20609 | -1 | - | 969 | Ribose ABC transport system, permease protein RbsC (TC 3.A.1.2.1) |
| CDS | contig00027 | 23103 | 21574 | -3 | - | 1530 | Ribose ABC transport system, ATP-binding protein RbsA (TC 3.A.1.2.1) |
| CDS | contig00027 | 23558 | 23139 | -2 | - | 420 | Ribose ABC transport system, high affinity permease RbsD (TC 3.A.1.2.1) |
| CDS | contig00027 | 24421 | 23804 | -1 | - | 618 | Glutathione S-transferase (EC 2.5.1.18) |
| CDS | contig00027 | 24679 | 25434 | 1 | + | 756 | Probable proline and glycine rich transmembrane protein gene in bax |
| CDS | contig00027 | 25496 | 26158 | 2 | + | 663 | Hypothetical protein, bax gene locus |
| CDS | contig00027 | 27138 | 26230 | -3 | - | 909 | tRNA(Cytosine32)-2-thiocytidine synthetase |
| CDS | contig00027 | 28153 | 27206 | -1 | - | 948 | Universal stress protein E |
| CDS | contig00027 | 29045 | 28290 | -2 | - | 756 | Fumarate and nitrate reduction regulatory protein |
| CDS | contig00027 | 29800 | 29114 | -1 | - | 687 | Heavy-metal-associated domain (N-terminus) and membrane-bounded cytochrome biogenesis cycZ-like domain, possible membrane copper tolerance protein |
| CDS | contig00027 | 29990 | 29784 | -2 | - | 207 | Type cbb3 cytochrome oxidase biogenesis protein CcoS, involved in heme b insertion |
| CDS | contig00027 | 32364 | 30073 | -3 | - | 2292 | Type cbb3 cytochrome oxidase biogenesis protein CcoI; Copper-translocating P-type ATPase (EC 3.6.3.4) |
| CDS | contig00027 | 32317 | 32556 | 1 | + | 240 | hypothetical protein |
| CDS | contig00027 | 33046 | 32558 | -1 | - | 489 | Putative analog of CcoH, COG3198 |
| CDS | contig00027 | 34149 | 33160 | -3 | - | 990 | Cytochrome c oxidase subunit CcoP (EC 1.9.3.1) |
| CDS | contig00027 | 34328 | 34149 | -2 | - | 180 | Cytochrome c oxidase subunit CcoQ (EC 1.9.3.1) |
| CDS | contig00027 | 34954 | 34340 | -1 | - | 615 | Cytochrome c oxidase subunit CcoO (EC 1.9.3.1) |
| CDS | contig00027 | 36397 | 34973 | -1 | - | 1425 | Cytochrome c oxidase subunit CcoN (EC 1.9.3.1) |
| CDS | contig00027 | 36732 | 36601 | -3 | - | 132 | hypothetical protein |
| CDS | contig00027 | 36734 | 37135 | 2 | + | 402 | hypothetical protein |
| CDS | contig00027 | 37244 | 37702 | 2 | + | 459 | Free methionine-(R)-sulfoxide reductase, contains GAF domain |
| CDS | contig00027 | 37804 | 38448 | 1 | + | 645 | ProQ: influences osmotic activation of compatible solute ProP |
| CDS | contig00027 | 38457 | 40472 | 3 | + | 2016 | Tail-specific protease precursor (EC 3.4.21.102) |
| CDS | contig00027 | 40602 | 43226 | 3 | + | 2625 | Membrane alanine aminopeptidase N (EC 3.4.11.2) |
| CDS | contig00027 | 43294 | 43515 | 1 | + | 222 | FIG00361373: hypothetical protein |
| CDS | contig00027 | 43790 | 48652 | 2 | + | 4863 | NAD-specific glutamate dehydrogenase (EC 1.4.1.2), large form |
| CDS | contig00027 | 48700 | 49710 | 1 | + | 1011 | Dihydroorotate dehydrogenase (EC 1.3.3.1) |
| CDS | contig00027 | 49796 | 50332 | 2 | + | 537 | FIG01199806: hypothetical protein |
| CDS | contig00027 | 50640 | 52811 | 3 | + | 2172 | 23S rRNA (guanine-N-2-) -methyltransferase rlmL EC 2.1.1.-) |
| CDS | contig00027 | 52811 | 53038 | 2 | + | 228 | Thiol-disulfide isomerase and thioredoxin |
| CDS | contig00027 | 53111 | 55021 | 2 | + | 1911 | ABC transporter ATP-binding protein uup |
| CDS | contig00027 | 55030 | 56679 | 1 | + | 1650 | FIG00361486: hypothetical protein |
| CDS | contig00027 | 56836 | 57015 | 1 | + | 180 | Ribosome modulation factor |
| CDS | contig00027 | 57656 | 57096 | -2 | - | 561 | 3-hydroxyacyl-[acyl-carrier-protein] dehydratase, FabA form (EC 4.2.1.59) |
| CDS | contig00027 | 59714 | 57735 | -2 | - | 1980 | ATP-dependent protease La (EC 3.4.21.53) Type II |
| CDS | contig00027 | 61190 | 59922 | -2 | - | 1269 | Site-specific recombinase, phage integrase family |
| CDS | contig00027 | 61928 | 62947 | 2 | + | 1020 | Site-specific recombinase, phage integrase family |
| CDS | contig00027 | 63753 | 63869 | 3 | + | 117 | hypothetical protein |
| CDS | contig00027 | 63962 | 64378 | 2 | + | 417 | FIG074102: hypothetical protein |
| CDS | contig00027 | 67209 | 65944 | -3 | - | 1266 | FIG014574: hypothetical protein |
| CDS | contig00027 | 69970 | 68060 | -1 | - | 1911 | Phage T7 exclusion protein |
| CDS | contig00027 | 73989 | 72544 | -3 | - | 1446 | DNA circulation protein, putative |
| CDS | contig00027 | 77255 | 74004 | -2 | - | 3252 | SMART domain SM00020; serine protease-like |
| CDS | contig00027 | 78406 | 77312 | -1 | - | 1095 | SMART domain SM00020; serine protease-like |
| CDS | contig00027 | 81845 | 78960 | -2 | - | 2886 | Mobile element protein |
| CDS | contig00027 | 81971 | 82585 | 2 | + | 615 | DNA-invertase |
| CDS | contig00028 | 1730 | 45 | -2 | - | 1686 | TrkA, Potassium channel-family protein |
| CDS | contig00028 | 2712 | 4724 | 3 | + | 2013 | Excinuclease ABC subunit B |
| CDS | contig00028 | 4721 | 4978 | 2 | + | 258 | FIG00362291: hypothetical protein |
| CDS | contig00028 | 5126 | 5788 | 2 | + | 663 | DedA protein |
| CDS | contig00028 | 6556 | 6035 | -1 | - | 522 | Periplasmic thiol:disulfide oxidoreductase DsbB, required for DsbA reoxidation |
| CDS | contig00028 | 8178 | 6643 | -3 | - | 1536 | Na+/H+ antiporter NhaB |
| CDS | contig00028 | 8551 | 9264 | 1 | + | 714 | Transcriptional regulator for fatty acid degradation FadR, GntR family |
| CDS | contig00028 | 10844 | 9324 | -2 | - | 1521 | FIG004684: SpoVR-like protein |
| CDS | contig00028 | 12126 | 10855 | -3 | - | 1272 | FIG002076: hypothetical protein |
| CDS | contig00028 | 14073 | 12151 | -3 | - | 1923 | Serine protein kinase (prkA protein), P-loop containing |
| CDS | contig00028 | 16569 | 14647 | -3 | - | 1923 | RecQ-like DNA helicase Pfl_0275 |
| CDS | contig00028 | 16902 | 18152 | 3 | + | 1251 | Sodium:dicarboxylate symporter |
| CDS | contig00028 | 20084 | 18888 | -2 | - | 1197 | Serine transporter |
| CDS | contig00028 | 20974 | 20402 | -1 | - | 573 | Hypothetical nudix hydrolase YeaB |
| CDS | contig00028 | 22382 | 21012 | -2 | - | 1371 | Para-aminobenzoate synthase, aminase component (EC 2.6.1.85) |
| CDS | contig00028 | 22565 | 22419 | -2 | - | 147 | hypothetical protein |
| CDS | contig00028 | 22593 | 24116 | 3 | + | 1524 | Fumarate hydratase class I, aerobic (EC 4.2.1.2) |
| CDS | contig00028 | 24413 | 24198 | -2 | - | 216 | Methyl-accepting chemotaxis protein I (serine chemoreceptor protein) |
| CDS | contig00028 | 24966 | 24487 | -3 | - | 480 | RecA/RadA recombinase |
| CDS | contig00028 | 25201 | 25398 | 1 | + | 198 | FIG00361720: hypothetical protein |
| CDS | contig00028 | 25827 | 25696 | -3 | - | 132 | hypothetical protein |
| CDS | contig00028 | 25792 | 28266 | 1 | + | 2475 | Glycogen phosphorylase (EC 2.4.1.1) |
| CDS | contig00028 | 28897 | 28331 | -1 | - | 567 | Ni,Fe-hydrogenase I cytochrome b subunit |
| CDS | contig00028 | 30487 | 29099 | -1 | - | 1389 | Di-and tricarboxylate transporter |
| CDS | contig00028 | 31082 | 30642 | -2 | - | 441 | Thioredoxin |
| CDS | contig00028 | 31236 | 34034 | 3 | + | 2799 | Sensory box histidine kinase/response regulator |
| CDS | contig00028 | 34135 | 35910 | 1 | + | 1776 | Lipid A export ATP-binding/permease protein MsbA |
| CDS | contig00028 | 36016 | 37767 | 1 | + | 1752 | Multidrug resistance-like ATP-binding protein mdlB |
| CDS | contig00028 | 38139 | 37822 | -3 | - | 318 | hypothetical protein |
| CDS | contig00028 | 39003 | 38647 | -3 | - | 357 | hypothetical protein |
| CDS | contig00028 | 39433 | 39906 | 1 | + | 474 | FIG00361496: hypothetical protein |
| CDS | contig00028 | 40296 | 40063 | -3 | - | 234 | FIG002958: hypothetical protein |
| CDS | contig00028 | 40866 | 40462 | -3 | - | 405 | MaoC family protein |
| CDS | contig00028 | 42650 | 40863 | -2 | - | 1788 | Polyhydroxyalkanoic acid synthase |
| CDS | contig00028 | 43099 | 42746 | -1 | - | 354 | FIG00362372: hypothetical protein |
| CDS | contig00028 | 43391 | 43954 | 2 | + | 564 | Uncharacterized low-complexity protein |
| CDS | contig00028 | 44047 | 44787 | 1 | + | 741 | Aeromonas caviae phaC PHA synthase,complete cds |
| CDS | contig00028 | 45299 | 46162 | 2 | + | 864 | Pyridoxal kinase (EC 2.7.1.35) |
| CDS | contig00028 | 46511 | 46203 | -2 | - | 309 | Antibiotic biosynthesis monooxygenase |
| CDS | contig00028 | 47122 | 46616 | -1 | - | 507 | PhnO protein |
| CDS | contig00028 | 47388 | 47224 | -3 | - | 165 | NTP pyrophosphohydrolases including oxidative damage repair enzymes |
| CDS | contig00028 | 48196 | 47480 | -1 | - | 717 | Adenylate cyclase (EC 4.6.1.1) |
| CDS | contig00028 | 48464 | 49309 | 2 | + | 846 | FIG00361752: hypothetical protein |
| CDS | contig00028 | 49510 | 49911 | 1 | + | 402 | N-acetylglutamate synthase (EC 2.3.1.1) |
| CDS | contig00028 | 49948 | 50385 | 1 | + | 438 | Histone acetyltransferase HPA2 and related acetyltransferases |
| CDS | contig00028 | 51844 | 50456 | -1 | - | 1389 | ATP-dependent RNA helicase RhlE |
| CDS | contig00028 | 52581 | 52105 | -3 | - | 477 | Glutathione peroxidase family protein |
| CDS | contig00028 | 52789 | 53397 | 1 | + | 609 | SM-20-related protein |
| CDS | contig00028 | 53598 | 54587 | 3 | + | 990 | D-alanine--D-alanine ligase (EC 6.3.2.4) |
| CDS | contig00028 | 54778 | 55527 | 1 | + | 750 | FIG074102: hypothetical protein |
| CDS | contig00028 | 55894 | 56673 | 1 | + | 780 | Putative membrane protein YfcA |
| CDS | contig00028 | 57658 | 56726 | -1 | - | 933 | Transcriptional regulator, LysR family |
| CDS | contig00028 | 58627 | 57710 | -1 | - | 918 | Permease of the drug/metabolite transporter (DMT) superfamily |
| CDS | contig00028 | 58794 | 59693 | 3 | + | 900 | Transcriptional regulator, AraC family |
| CDS | contig00028 | 59756 | 59878 | 2 | + | 123 | hypothetical protein |
| CDS | contig00028 | 59955 | 61589 | 3 | + | 1635 | Predicted ATP-dependent endonuclease of the OLD family, YbjD subgroup |
| CDS | contig00028 | 61696 | 62415 | 1 | + | 720 | hypothetical protein |
| CDS | contig00029 | 182 | 1627 | 2 | + | 1446 | Cytosol aminopeptidase PepA (EC 3.4.11.1) |
| CDS | contig00029 | 1661 | 2341 | 2 | + | 681 | 5&#39;-nucleotidase YjjG (EC 3.1.3.5) |
| CDS | contig00029 | 3668 | 2412 | -2 | - | 1257 | Catalyzes the cleavage of p-aminobenzoyl-glutamate to p-aminobenzoate and glutamate, subunit A |
| CDS | contig00029 | 4061 | 3933 | -2 | - | 129 | hypothetical protein |
| CDS | contig00029 | 4645 | 6453 | 1 | + | 1809 | possible site-specific recombinase |
| CDS | contig00029 | 7257 | 6931 | -3 | - | 327 | Phage DNA invertase |
| CDS | contig00029 | 9876 | 12332 | 3 | + | 2457 | Type I restriction-modification system, restriction subunit R (EC 3.1.21.3) |
| CDS | contig00029 | 12424 | 13890 | 1 | + | 1467 | Type I restriction-modification system, DNA-methyltransferase subunit M (EC 2.1.1.72) |
| CDS | contig00029 | 13893 | 15581 | 3 | + | 1689 | Type I restriction-modification system, specificity subunit S (EC 3.1.21.3) |
| CDS | contig00029 | 15581 | 16573 | 2 | + | 993 | UDP-N-acetylmuramoylalanyl-D-glutamate--2,6-diaminopimelate ligase (EC 6.3.2.13) |
| CDS | contig00029 | 17473 | 16994 | -1 | - | 480 | hypothetical protein |
| CDS | contig00029 | 18529 | 17474 | -1 | - | 1056 | Transcriptional regulator, XRE family |
| CDS | contig00030 | 537 | 1802 | 3 | + | 1266 | Cytosine deaminase (EC 3.5.4.1) |
| CDS | contig00030 | 1890 | 2267 | 3 | + | 378 | CrcB protein |
| CDS | contig00030 | 2333 | 3085 | 2 | + | 753 | Ubiquinone/menaquinone biosynthesis methyltransferase UbiE (EC 2.1.1.-) @ 2-heptaprenyl-1,4-naphthoquinone methyltransferase (EC 2.1.1.163) |
| CDS | contig00030 | 3094 | 3702 | 1 | + | 609 | Protein YigP (COG3165) clustered with ubiquinone biosynthetic genes |
| CDS | contig00030 | 3702 | 5342 | 3 | + | 1641 | Ubiquinone biosynthesis monooxygenase UbiB |
| CDS | contig00030 | 5382 | 5630 | 3 | + | 249 | Twin-arginine translocation protein TatA |
| CDS | contig00030 | 5634 | 6092 | 3 | + | 459 | Twin-arginine translocation protein TatB |
| CDS | contig00030 | 6089 | 6841 | 2 | + | 753 | Twin-arginine translocation protein TatC |
| CDS | contig00030 | 6953 | 7738 | 2 | + | 786 | Deoxyribonuclease TatD |
| CDS | contig00030 | 7742 | 8761 | 2 | + | 1020 | Porphobilinogen synthase (EC 4.2.1.24) |
| CDS | contig00030 | 9004 | 11025 | 1 | + | 2022 | Oligopeptidase A (EC 3.4.24.70) |
| CDS | contig00030 | 11260 | 13827 | 1 | + | 2568 | diguanylate cyclase/phosphodiesterase (GGDEF & EAL domains) with PAS/PAC sensor(s) |
| CDS | contig00030 | 13879 | 14637 | 1 | + | 759 | SAM-dependent methyltransferase (EC 2.1.1.-) |
| CDS | contig00030 | 14968 | 14708 | -1 | - | 261 | Rho-specific inhibitor of transcription termination (YaeO) |
| CDS | contig00030 | 16471 | 14981 | -1 | - | 1491 | Guanosine-5&#39;-triphosphate,3&#39;-diphosphate pyrophosphatase (EC 3.6.1.40) @ Exopolyphosphatase (EC 3.6.1.11) |
| CDS | contig00030 | 17735 | 16485 | -2 | - | 1251 | ATP-dependent RNA helicase RhlB |
| CDS | contig00030 | 17888 | 18214 | 2 | + | 327 | Thioredoxin |
| CDS | contig00030 | 18452 | 19687 | 2 | + | 1236 | Transcription termination factor Rho |
| CDS | contig00030 | 19794 | 20801 | 3 | + | 1008 | FIG01199582: hypothetical protein |
| CDS | contig00030 | 20835 | 22304 | 3 | + | 1470 | 3-polyprenyl-4-hydroxybenzoate carboxy-lyase (EC 4.1.1.-) |
| CDS | contig00030 | 22460 | 23158 | 2 | + | 699 | NAD(P)H-flavin reductase (EC 1.5.1.29) (EC 1.16.1.3) |
| CDS | contig00030 | 23555 | 23298 | -2 | - | 258 | FIG00362404: hypothetical protein |
| CDS | contig00030 | 23857 | 26274 | 1 | + | 2418 | putative lipase |
| CDS | contig00030 | 26686 | 26381 | -1 | - | 306 | hypothetical protein |
| CDS | contig00030 | 27549 | 26683 | -3 | - | 867 | GlpG protein (membrane protein of glp regulon) |
| CDS | contig00030 | 27869 | 27546 | -2 | - | 324 | Thiosulfate sulfurtransferase GlpE (EC 2.8.1.1) |
| CDS | contig00030 | 28033 | 28626 | 1 | + | 594 | Transporter, LysE family |
| CDS | contig00030 | 29450 | 28605 | -2 | - | 846 | Transcriptional regulator, AraC family |
| CDS | contig00030 | 30234 | 29533 | -3 | - | 702 | Branched-chain amino acid transport ATP-binding protein LivF (TC 3.A.1.4.1) |
| CDS | contig00030 | 31018 | 30245 | -1 | - | 774 | Branched-chain amino acid transport ATP-binding protein LivG (TC 3.A.1.4.1) |
| CDS | contig00030 | 32283 | 31015 | -3 | - | 1269 | Branched-chain amino acid transport system permease protein LivM (TC 3.A.1.4.1) |
| CDS | contig00030 | 33292 | 32366 | -1 | - | 927 | High-affinity branched-chain amino acid transport system permease protein LivH (TC 3.A.1.4.1) |
| CDS | contig00030 | 34480 | 33359 | -1 | - | 1122 | High-affinity leucine-specific transport system, periplasmic binding protein LivK (TC 3.A.1.4.1) |
| CDS | contig00030 | 34916 | 35920 | 2 | + | 1005 | Purine nucleotide synthesis repressor |
| CDS | contig00030 | 37530 | 36004 | -3 | - | 1527 | Anaerobic nitric oxide reductase transcription regulator NorR |
| CDS | contig00030 | 37701 | 39185 | 3 | + | 1485 | Anaerobic nitric oxide reductase flavorubredoxin |
| CDS | contig00030 | 39182 | 40348 | 2 | + | 1167 | Nitric oxide reductase FlRd-NAD(+) reductase (EC 1.18.1.-) |
| CDS | contig00030 | 40401 | 40739 | 3 | + | 339 | FIG074102: hypothetical protein |
| CDS | contig00030 | 40862 | 42079 | 2 | + | 1218 | Hypothetical iron-sulfur cluster binding protein YccM |
| CDS | contig00030 | 42223 | 42339 | 1 | + | 117 | hypothetical protein |
| CDS | contig00030 | 42543 | 44009 | 3 | + | 1467 | Tryptophanase (EC 4.1.99.1) |
| CDS | contig00030 | 44080 | 45294 | 1 | + | 1215 | Tryptophan-specific transport protein |
| CDS | contig00030 | 45446 | 45282 | -2 | - | 165 | hypothetical protein |
| CDS | contig00030 | 45555 | 46709 | 3 | + | 1155 | Tryptophan-specific transport protein |
| CDS | contig00030 | 47207 | 46803 | -2 | - | 405 | Protein ycgK precursor |
| CDS | contig00030 | 48319 | 47546 | -1 | - | 774 | hypothetical protein |
| CDS | contig00030 | 49249 | 48485 | -1 | - | 765 | FOG: EAL domain protein |
| CDS | contig00030 | 49202 | 49597 | 2 | + | 396 | hypothetical protein |
| CDS | contig00030 | 53200 | 51110 | -1 | - | 2091 | Glycyl-tRNA synthetase beta chain (EC 6.1.1.14) |
| CDS | contig00030 | 54133 | 53210 | -1 | - | 924 | Glycyl-tRNA synthetase alpha chain (EC 6.1.1.14) |
| CDS | contig00030 | 54321 | 54899 | 3 | + | 579 | DNA-3-methyladenine glycosylase (EC 3.2.2.20) |
| CDS | contig00030 | 55520 | 54954 | -2 | - | 567 | Isochorismatase (EC 3.3.2.1) |
| CDS | contig00030 | 55938 | 56180 | 3 | + | 243 | hypothetical protein |
| CDS | contig00030 | 56342 | 56998 | 2 | + | 657 | Outer membrane lipoprotein omp16 precursor |
| CDS | contig00030 | 57844 | 57497 | -1 | - | 348 | RidA/YER057c/UK114 superfamily, group 2, YoaB-like protein |
| CDS | contig00030 | 58108 | 57863 | -1 | - | 246 | tRNA 5-methylaminomethyl-2-thiouridine synthase TusA |
| CDS | contig00030 | 58221 | 58105 | -3 | - | 117 | tRNA 5-methylaminomethyl-2-thiouridine synthase TusA |
| CDS | contig00030 | 58613 | 58804 | 2 | + | 192 | FIG00361941: hypothetical protein |
| CDS | contig00030 | 60105 | 58849 | -3 | - | 1257 | ABC-type sugar transport system, periplasmic component |
| CDS | contig00030 | 61554 | 60109 | -3 | - | 1446 | Integral membrane sensor signal transduction histidine kinase (EC 2.7.13.3), glucose catabolism cluster |
| CDS | contig00030 | 62390 | 61668 | -2 | - | 723 | TorCAD operon transcriptional regulatory protein TorR |
| CDS | contig00030 | 63888 | 62725 | -3 | - | 1164 | 3-ketoacyl-CoA thiolase (EC 2.3.1.16) @ Acetyl-CoA acetyltransferase (EC 2.3.1.9) |
| CDS | contig00030 | 66057 | 63910 | -3 | - | 2148 | Enoyl-CoA hydratase (EC 4.2.1.17) / Delta(3)-cis-delta(2)-trans-enoyl-CoA isomerase (EC 5.3.3.8) / 3-hydroxyacyl-CoA dehydrogenase (EC 1.1.1.35) / 3-hydroxybutyryl-CoA epimerase (EC 5.1.2.3) |
| CDS | contig00030 | 68198 | 66261 | -2 | - | 1938 | Acetoacetyl-CoA synthetase (EC 6.2.1.16) |
| CDS | contig00030 | 68395 | 69717 | 1 | + | 1323 | Xaa-Pro dipeptidase PepQ (EC 3.4.13.9) |
| CDS | contig00030 | 69717 | 70334 | 3 | + | 618 | FIG000605: protein co-occurring with transport systems (COG1739) |
| CDS | contig00030 | 70371 | 71828 | 3 | + | 1458 | Potassium uptake protein TrkH |
| CDS | contig00030 | 71890 | 72360 | 1 | + | 471 | Protoporphyrinogen IX oxidase, oxygen-independent, HemG (EC 1.3.-.-) |
| CDS | contig00031 | 348 | 1349 | 3 | + | 1002 | Sulfate and thiosulfate binding protein CysP |
| CDS | contig00031 | 1389 | 1964 | 3 | + | 576 | FIG00974664: hypothetical protein |
| CDS | contig00031 | 2168 | 3073 | 2 | + | 906 | Sulfate transport system permease protein CysT |
| CDS | contig00031 | 3070 | 3963 | 1 | + | 894 | Sulfate transport system permease protein CysW |
| CDS | contig00031 | 3995 | 5056 | 2 | + | 1062 | Sulfate and thiosulfate import ATP-binding protein CysA (EC 3.6.3.25) |
| CDS | contig00031 | 5173 | 6120 | 1 | + | 948 | Alkanesulfonate utilization operon LysR-family regulator CbI |
| CDS | contig00031 | 7653 | 6232 | -3 | - | 1422 | Chitin binding protein |
| CDS | contig00031 | 7627 | 7863 | 1 | + | 237 | hypothetical protein |
| CDS | contig00031 | 9708 | 8029 | -3 | - | 1680 | Methyl-accepting chemotaxis protein |
| CDS | contig00031 | 10003 | 11853 | 1 | + | 1851 | probable peptidase VC1983 |
| CDS | contig00031 | 12323 | 12730 | 2 | + | 408 | ErfK/YbiS/YcfS/YnhG family protein |
| CDS | contig00031 | 12852 | 13565 | 3 | + | 714 | FIG006238: AzlC family protein |
| CDS | contig00031 | 13568 | 13903 | 2 | + | 336 | FIG00361879: hypothetical protein |
| CDS | contig00031 | 14026 | 15552 | 1 | + | 1527 | FIG00919702: hypothetical protein |
| CDS | contig00031 | 15560 | 16438 | 2 | + | 879 | radical activating enzyme |
| CDS | contig00031 | 16556 | 16879 | 2 | + | 324 | FIG00361563: hypothetical protein |
| CDS | contig00031 | 17828 | 16896 | -2 | - | 933 | LysR-family transcriptional regulator VCA0830 |
| CDS | contig00031 | 19083 | 17887 | -3 | - | 1197 | Aromatic-amino-acid aminotransferase (EC 2.6.1.57) |
| CDS | contig00031 | 19475 | 19600 | 2 | + | 126 | hypothetical protein |
| CDS | contig00031 | 19609 | 21123 | 1 | + | 1515 | FIG002337: predicted inner membrane protein |
| CDS | contig00031 | 21138 | 21362 | 3 | + | 225 | hypothetical protein |
| CDS | contig00031 | 21359 | 22021 | 2 | + | 663 | Cellulose synthase, putative |
| CDS | contig00031 | 22018 | 24585 | 1 | + | 2568 | Cellulose synthase catalytic subunit [UDP-forming] (EC 2.4.1.12) |
| CDS | contig00031 | 24582 | 26807 | 3 | + | 2226 | Cyclic di-GMP binding protein precursor |
| CDS | contig00031 | 26811 | 27920 | 3 | + | 1110 | Endoglucanase precursor (EC 3.2.1.4) |
| CDS | contig00031 | 27905 | 31345 | 2 | + | 3441 | Cellulose synthase operon protein C |
| CDS | contig00031 | 31649 | 31374 | -2 | - | 276 | probable alpha helix chain yaiN |
| CDS | contig00031 | 31721 | 32662 | 2 | + | 942 | Cobalt-zinc-cadmium resistance protein CzcD |
| CDS | contig00031 | 33082 | 32678 | -1 | - | 405 | FIG00361812: hypothetical protein |
| CDS | contig00031 | 33342 | 37400 | 3 | + | 4059 | Type II secretory pathway, pullulanase |
| CDS | contig00031 | 40017 | 37594 | -3 | - | 2424 | Lead, cadmium, zinc and mercury transporting ATPase (EC 3.6.3.3) (EC 3.6.3.5); Copper-translocating P-type ATPase (EC 3.6.3.4) |
| CDS | contig00031 | 40272 | 40772 | 3 | + | 501 | Thiol peroxidase, Tpx-type (EC 1.11.1.15) |
| CDS | contig00031 | 40919 | 42139 | 2 | + | 1221 | ATP-dependent RNA helicase VCA0990 |
| CDS | contig00031 | 43795 | 42236 | -1 | - | 1560 | Aerotaxis sensor receptor protein |
| CDS | contig00031 | 44247 | 43948 | -3 | - | 300 | hypothetical protein |
| CDS | contig00031 | 45962 | 44358 | -2 | - | 1605 | Periplasmic Murein Peptide-Binding Protein MppA |
| CDS | contig00031 | 46155 | 46844 | 3 | + | 690 | FIG00362035: hypothetical protein |
| CDS | contig00031 | 46930 | 47358 | 1 | + | 429 | FIG00361913: hypothetical protein |
| CDS | contig00031 | 48110 | 47361 | -2 | - | 750 | Enoyl-CoA hydratase (EC 4.2.1.17) |
| CDS | contig00031 | 48200 | 48640 | 2 | + | 441 | Copper metallochaperone, bacterial analog of Cox17 protein |
| CDS | contig00031 | 48733 | 49362 | 1 | + | 630 | FIG081498: hypothetical protein |
| CDS | contig00031 | 49381 | 50061 | 1 | + | 681 | putative short-chain dehydrogenase |
| CDS | contig00031 | 50883 | 50254 | -3 | - | 630 | DNA polymerase III alpha subunit (EC 2.7.7.7) |
| CDS | contig00031 | 52802 | 51078 | -2 | - | 1725 | Prolyl-tRNA synthetase (EC 6.1.1.15), bacterial type |
| CDS | contig00031 | 54949 | 52940 | -1 | - | 2010 | N-acetylglucosamine regulated methyl-accepting chemotaxis protein |
| CDS | contig00031 | 55182 | 55796 | 3 | + | 615 | Holliday junction DNA helicase RuvA |
| CDS | contig00031 | 55805 | 56815 | 2 | + | 1011 | Holliday junction DNA helicase RuvB |
| CDS | contig00031 | 57755 | 59311 | 2 | + | 1557 | Cytochrome d ubiquinol oxidase subunit I (EC 1.10.3.-) |
| CDS | contig00031 | 59324 | 60460 | 2 | + | 1137 | Cytochrome d ubiquinol oxidase subunit II (EC 1.10.3.-) |
| CDS | contig00031 | 60475 | 60591 | 1 | + | 117 | Cyd operon protein YbgT |
| CDS | contig00031 | 60578 | 60889 | 2 | + | 312 | Cyd operon protein YbgE |
| CDS | contig00031 | 61176 | 61583 | 3 | + | 408 | 4-hydroxybenzoyl-CoA thioesterase family active site |
| CDS | contig00031 | 61573 | 62259 | 1 | + | 687 | MotA/TolQ/ExbB proton channel family protein |
| CDS | contig00031 | 62259 | 62705 | 3 | + | 447 | Tol biopolymer transport system, TolR protein |
| CDS | contig00031 | 62711 | 63841 | 2 | + | 1131 | TolA protein |
| CDS | contig00031 | 63919 | 65244 | 1 | + | 1326 | tolB protein precursor, periplasmic protein involved in the tonb-independent uptake of group A colicins |
| CDS | contig00031 | 65293 | 65817 | 1 | + | 525 | 18K peptidoglycan-associated outer membrane lipoprotein; Peptidoglycan-associated lipoprotein precursor; Outer membrane protein P6; OmpA/MotB precursor |
| CDS | contig00031 | 65984 | 66784 | 2 | + | 801 | TPR repeat containing exported protein; Putative periplasmic protein contains a protein prenylyltransferase domain |
| CDS | contig00032 | 2178 | 1288 | -3 | - | 891 | DNA replication terminus site-binding protein |
| CDS | contig00032 | 3623 | 3360 | -2 | - | 264 | hypothetical protein |
| CDS | contig00032 | 3886 | 3656 | -1 | - | 231 | hypothetical protein |
| CDS | contig00032 | 4516 | 4232 | -1 | - | 285 | hypothetical protein |
| CDS | contig00032 | 4716 | 4534 | -3 | - | 183 | hypothetical protein |
| CDS | contig00032 | 5103 | 4738 | -3 | - | 366 | Phage protein |
| CDS | contig00032 | 5422 | 5153 | -1 | - | 270 | hypothetical protein |
| CDS | contig00032 | 5672 | 5484 | -2 | - | 189 | hypothetical protein |
| CDS | contig00032 | 6278 | 5718 | -2 | - | 561 | hypothetical protein |
| CDS | contig00032 | 6584 | 6291 | -2 | - | 294 | hypothetical protein |
| CDS | contig00032 | 7566 | 7321 | -3 | - | 246 | hypothetical protein |
| CDS | contig00032 | 9986 | 9324 | -2 | - | 663 | hypothetical protein |
| CDS | contig00032 | 10869 | 11006 | 3 | + | 138 | hypothetical protein |
| CDS | contig00032 | 12133 | 12537 | 1 | + | 405 | replication protein RepA |
| CDS | contig00032 | 13080 | 13367 | 3 | + | 288 | hypothetical protein |
| CDS | contig00032 | 15460 | 15332 | -1 | - | 129 | hypothetical protein |
| CDS | contig00032 | 18414 | 18193 | -3 | - | 222 | hypothetical protein |
| CDS | contig00032 | 20305 | 19937 | -1 | - | 369 | hypothetical protein |
| CDS | contig00032 | 21275 | 20556 | -2 | - | 720 | FIG01059005: hypothetical protein |
| CDS | contig00032 | 22216 | 21437 | -1 | - | 780 | hypothetical protein |
| CDS | contig00032 | 22788 | 22597 | -3 | - | 192 | hypothetical protein |
| CDS | contig00032 | 23308 | 22919 | -1 | - | 390 | hypothetical protein |
| CDS | contig00032 | 23909 | 23793 | -2 | - | 117 | hypothetical protein |
| CDS | contig00032 | 25209 | 25478 | 3 | + | 270 | HigB toxin protein |
| CDS | contig00032 | 28180 | 28512 | 1 | + | 333 | RelE/StbE replicon stabilization toxin |
| CDS | contig00032 | 28514 | 28798 | 2 | + | 285 | Transcriptional regulator, Cro/CI family |
| CDS | contig00032 | 30050 | 29844 | -2 | - | 207 | Cold shock domain family protein |
| CDS | contig00032 | 33754 | 30701 | -1 | - | 3054 | hypothetical protein |
| CDS | contig00032 | 34036 | 33815 | -1 | - | 222 | hypothetical protein |
| CDS | contig00032 | 37278 | 36235 | -3 | - | 1044 | hypothetical protein |
| CDS | contig00032 | 37578 | 38315 | 3 | + | 738 | hypothetical protein |
| CDS | contig00032 | 38529 | 39632 | 3 | + | 1104 | IncF plasmid conjugative transfer pilus assembly protein TraF |
| CDS | contig00032 | 39632 | 40966 | 2 | + | 1335 | putative conjugative transfer protein TraH |
| CDS | contig00032 | 41011 | 44151 | 1 | + | 3141 | IncF plasmid conjugative transfer protein TraG |
| CDS | contig00032 | 45966 | 44815 | -3 | - | 1152 | Error-prone, lesion bypass DNA polymerase V (UmuC) |
| CDS | contig00032 | 46337 | 46083 | -2 | - | 255 | Error-prone repair protein UmuD |
| CDS | contig00032 | 48352 | 47216 | -1 | - | 1137 | Mobile element protein |
| CDS | contig00032 | 50375 | 49989 | -2 | - | 387 | hypothetical protein |
| CDS | contig00032 | 51934 | 50516 | -1 | - | 1419 | Modification methylase ScrFIA (EC 2.1.1.37) |
| CDS | contig00032 | 53035 | 52520 | -1 | - | 516 | hypothetical protein |
| CDS | contig00032 | 54119 | 53049 | -2 | - | 1071 | hypothetical protein |
| CDS | contig00032 | 55864 | 54326 | -1 | - | 1539 | Putative peptidoglycan bound protein (LPXTG motif) Lmo1799 homolog |
| CDS | contig00032 | 55844 | 56227 | 2 | + | 384 | hypothetical protein |
| CDS | contig00032 | 57260 | 56298 | -2 | - | 963 | hypothetical protein |
| CDS | contig00032 | 58578 | 57523 | -3 | - | 1056 | Nitric oxide reductase activation protein NorQ |
| CDS | contig00032 | 59778 | 58690 | -3 | - | 1089 | DNA primase (EC 2.7.7.-), phage-associated |
| CDS | contig00032 | 59910 | 59791 | -3 | - | 120 | hypothetical protein |
| CDS | contig00033 | 519 | 46 | -3 | - | 474 | diguanylate cyclase/phosphodiesterase (GGDEF & EAL domains) with PAS/PAC sensor(s) |
| CDS | contig00033 | 2032 | 509 | -1 | - | 1524 | diguanylate cyclase/phosphodiesterase (GGDEF & EAL domains) with PAS/PAC sensor(s) |
| CDS | contig00033 | 2379 | 2203 | -3 | - | 177 | hypothetical protein |
| CDS | contig00033 | 2404 | 4548 | 1 | + | 2145 | Ferrichrome-iron receptor |
| CDS | contig00033 | 4611 | 5399 | 3 | + | 789 | Hypothetical protein in aerobactin uptake cluster |
| CDS | contig00033 | 6408 | 5473 | -3 | - | 936 | Transcriptional activator MetR |
| CDS | contig00033 | 6569 | 8833 | 2 | + | 2265 | 5-methyltetrahydropteroyltriglutamate--homocysteine methyltransferase (EC 2.1.1.14) |
| CDS | contig00033 | 10039 | 8927 | -1 | - | 1113 | Lipopolysaccharide heptosyltransferase I (EC 2.4.1.-) |
| CDS | contig00033 | 10217 | 11338 | 2 | + | 1122 | Probable transmembrane protein |
| CDS | contig00033 | 13313 | 11382 | -2 | - | 1932 | Exoenzymes regulatory protein AepA precursor |
| CDS | contig00033 | 13497 | 14195 | 3 | + | 699 | Transcriptional regulator, TetR family |
| CDS | contig00033 | 15503 | 14250 | -2 | - | 1254 | D-amino acid dehydrogenase small subunit (EC 1.4.99.1) |
| CDS | contig00033 | 17177 | 15657 | -2 | - | 1521 | FIG004436: Protein related to deoxyribodipyrimidine photolyase |
| CDS | contig00033 | 18401 | 17481 | -2 | - | 921 | Murein-DD-endopeptidase (EC 3.4.99.-) |
| CDS | contig00033 | 19942 | 18752 | -1 | - | 1191 | Cytochrome c-type heme lyase subunit nrfG, nitrite reductase complex assembly |
| CDS | contig00033 | 20538 | 19939 | -3 | - | 600 | Putative thiol:disulfide oxidoreductase, nitrite reductase complex assembly |
| CDS | contig00033 | 22496 | 20535 | -2 | - | 1962 | Cytochrome c-type heme lyase subunit nrfE, nitrite reductase complex assembly |
| CDS | contig00033 | 23623 | 22670 | -1 | - | 954 | NrfD protein |
| CDS | contig00033 | 24391 | 23711 | -1 | - | 681 | NrfC protein |
| CDS | contig00033 | 24981 | 24388 | -3 | - | 594 | Cytochrome c-type protein NrfB precursor |
| CDS | contig00033 | 26496 | 25033 | -3 | - | 1464 | Cytochrome c552 precursor (EC 1.7.2.2) |
| CDS | contig00033 | 29789 | 27195 | -2 | - | 2595 | hypothetical protein |
| CDS | contig00041 | 23 | 7852 | 2 | + | 7830 | Alkaline phosphatase (EC 3.1.3.1) |
| CDS | contig00041 | 9371 | 8169 | -2 | - | 1203 | Anaerobic glycerol-3-phosphate dehydrogenase subunit C (EC 1.1.5.3) |
| CDS | contig00041 | 10839 | 9562 | -3 | - | 1278 | Anaerobic glycerol-3-phosphate dehydrogenase subunit B (EC 1.1.5.3) |
| CDS | contig00041 | 12508 | 10829 | -1 | - | 1680 | Anaerobic glycerol-3-phosphate dehydrogenase subunit A (EC 1.1.5.3) |
| CDS | contig00041 | 13185 | 12730 | -3 | - | 456 | UPF0225 protein YchJ |
| CDS | contig00041 | 13314 | 14981 | 3 | + | 1668 | Methyl-accepting chemotaxis protein |
| CDS | contig00041 | 15098 | 16297 | 2 | + | 1200 | Mlc, transcriptional repressor of MalT (the transcriptional activator of maltose regulon) and manXYZ operon |
| CDS | contig00041 | 16294 | 17877 | 1 | + | 1584 | Sensor kinase CitA, DpiB (EC 2.7.3.-) |
| CDS | contig00041 | 17878 | 18543 | 1 | + | 666 | Fumarate respiration transcriptional regulator DcuR |
| CDS | contig00041 | 20210 | 18681 | -2 | - | 1530 | Tricarboxylate transport membrane protein TctA |
| CDS | contig00041 | 20681 | 20220 | -2 | - | 462 | Tricarboxylate transport protein TctB |
| CDS | contig00041 | 21704 | 20739 | -2 | - | 966 | Tricarboxylate transport protein TctC |
| CDS | contig00041 | 23396 | 21879 | -2 | - | 1518 | Transglycosylase, Slt family |
| CDS | contig00042 | 55 | 891 | 1 | + | 837 | Maltoporin (maltose/maltodextrin high-affinity receptor, phage lambda receptor protein) |
| CDS | contig00042 | 1095 | 1943 | 3 | + | 849 | Maltose operon periplasmic protein MalM |
| CDS | contig00042 | 2376 | 3809 | 3 | + | 1434 | PTS system, glucose-specific IIB component (EC 2.7.1.69) / PTS system, glucose-specific IIC component (EC 2.7.1.69) |
| CDS | contig00042 | 4356 | 3973 | -3 | - | 384 | UPF0325 protein YaeH |
| CDS | contig00042 | 5457 | 4630 | -3 | - | 828 | 2,3,4,5-tetrahydropyridine-2,6-dicarboxylate N-succinyltransferase (EC 2.3.1.117) |
| CDS | contig00042 | 8278 | 5636 | -1 | - | 2643 | [Protein-PII] uridylyltransferase (EC 2.7.7.59) |
| CDS | contig00042 | 9131 | 8343 | -2 | - | 789 | Methionine aminopeptidase (EC 3.4.11.18) |
| CDS | contig00042 | 9505 | 10218 | 1 | + | 714 | SSU ribosomal protein S2p (SAe) |
| CDS | contig00042 | 10323 | 11204 | 3 | + | 882 | Translation elongation factor Ts |
| CDS | contig00042 | 11276 | 12010 | 2 | + | 735 | Uridine monophosphate kinase (EC 2.7.4.22) |
| CDS | contig00042 | 12106 | 12663 | 1 | + | 558 | Ribosome recycling factor |
| CDS | contig00042 | 12736 | 13512 | 1 | + | 777 | Undecaprenyl diphosphate synthase (EC 2.5.1.31) |
| CDS | contig00042 | 13585 | 14397 | 1 | + | 813 | Phosphatidate cytidylyltransferase (EC 2.7.7.41) |
| CDS | contig00042 | 14465 | 15661 | 2 | + | 1197 | 1-deoxy-D-xylulose 5-phosphate reductoisomerase (EC 1.1.1.267) |
| CDS | contig00042 | 15738 | 17090 | 3 | + | 1353 | Membrane-associated zinc metalloprotease |
| CDS | contig00042 | 17125 | 19548 | 1 | + | 2424 | Outer membrane protein assembly factor YaeT precursor |
| CDS | contig00042 | 19695 | 20207 | 3 | + | 513 | Outer membrane protein H precursor |
| CDS | contig00042 | 20211 | 21230 | 3 | + | 1020 | UDP-3-O-[3-hydroxymyristoyl] glucosamine N-acyltransferase (EC 2.3.1.191) |
| CDS | contig00042 | 21294 | 21716 | 3 | + | 423 | 3-hydroxyacyl-[acyl-carrier-protein] dehydratase, FabZ form (EC 4.2.1.59) |
| CDS | contig00042 | 21719 | 22510 | 2 | + | 792 | Acyl-[acyl-carrier-protein]--UDP-N-acetylglucosamine O-acyltransferase (EC 2.3.1.129) |
| CDS | contig00042 | 22503 | 23642 | 3 | + | 1140 | Lipid-A-disaccharide synthase (EC 2.4.1.182) |
| CDS | contig00042 | 23721 | 24311 | 3 | + | 591 | Ribonuclease HII (EC 3.1.26.4) |
| CDS | contig00042 | 24359 | 27844 | 2 | + | 3486 | DNA polymerase III alpha subunit (EC 2.7.7.7) |
| CDS | contig00042 | 27861 | 28811 | 3 | + | 951 | Acetyl-coenzyme A carboxyl transferase alpha chain (EC 6.4.1.2) |
| CDS | contig00042 | 30284 | 28893 | -2 | - | 1392 | Multi antimicrobial extrusion protein (Na(+)/drug antiporter), MATE family of MDR efflux pumps |
| CDS | contig00042 | 31296 | 30391 | -3 | - | 906 | Transcriptional regulator |
| CDS | contig00042 | 31793 | 33085 | 2 | + | 1293 | Serine transporter |
| CDS | contig00042 | 33220 | 34491 | 1 | + | 1272 | Putative transport protein |
| CDS | contig00042 | 35071 | 34535 | -1 | - | 537 | Ribosomal-protein-alanine acetyltransferase (EC 2.3.1.128) |
| CDS | contig00042 | 36859 | 35144 | -1 | - | 1716 | Probable ABC transporter ATP binding protein |
| CDS | contig00042 | 37660 | 37223 | -1 | - | 438 | FIG00362025: hypothetical protein |
| CDS | contig00042 | 39803 | 37773 | -2 | - | 2031 | Peptidase, M13 family |
| CDS | contig00042 | 39933 | 41267 | 3 | + | 1335 | tRNA(Ile)-lysidine synthetase (EC 6.3.4.19) |
| CDS | contig00042 | 42194 | 41310 | -2 | - | 885 | diguanylate cyclase (GGDEF domain) with PAS/PAC sensor |
| CDS | contig00042 | 42430 | 44382 | 1 | + | 1953 | Glutathione-regulated potassium-efflux system protein KefB |
| CDS | contig00042 | 45570 | 44410 | -3 | - | 1161 | Patatin |
| CDS | contig00042 | 46551 | 45688 | -3 | - | 864 | Predicted hydrolase or acyltransferase |
| CDS | contig00042 | 47199 | 46618 | -3 | - | 582 | Transcriptional regulator, TetR family |
| CDS | contig00042 | 48225 | 47581 | -3 | - | 645 | Transcriptional regulator, LuxR family |
| CDS | contig00042 | 48384 | 49010 | 3 | + | 627 | Glutathione S-transferase family protein |
| CDS | contig00042 | 51197 | 49074 | -2 | - | 2124 | Periplasmic alpha-amylase (EC 3.2.1.1) |
| CDS | contig00042 | 52570 | 51404 | -1 | - | 1167 | Integral membrane protein |
| CDS | contig00042 | 52941 | 52570 | -3 | - | 372 | DNA for glycosyltransferase, lytic transglycosylase, dTDP-4-rhamnose reductase, complete cds |
| CDS | contig00042 | 53996 | 52938 | -2 | - | 1059 | Glycosyl transferase, family 2 |
| CDS | contig00042 | 54599 | 54054 | -2 | - | 546 | FIG00361866: hypothetical protein |
| CDS | contig00042 | 54909 | 55676 | 3 | + | 768 | Putative amino acid ABC transporter, periplasmic amino acid-binding protein |
| CDS | contig00042 | 55884 | 55699 | -3 | - | 186 | hypothetical protein |
| CDS | contig00042 | 55861 | 57249 | 1 | + | 1389 | FIG00361271: hypothetical protein |
| CDS | contig00043 | 854 | 1126 | 2 | + | 273 | hypothetical protein |
| CDS | contig00043 | 1657 | 1343 | -1 | - | 315 | Quaternary ammonium compound-resistance protein SugE |
| CDS | contig00043 | 1997 | 3109 | 2 | + | 1113 | Aspartate-semialdehyde dehydrogenase (EC 1.2.1.11) |
| CDS | contig00043 | 3444 | 3226 | -3 | - | 219 | Cold shock protein CspD |
| CDS | contig00043 | 3673 | 3990 | 1 | + | 318 | ATP-dependent Clp protease adaptor protein ClpS |
| CDS | contig00043 | 4051 | 6303 | 1 | + | 2253 | ATP-dependent Clp protease ATP-binding subunit ClpA |
| CDS | contig00043 | 6591 | 6373 | -3 | - | 219 | Translation initiation factor 1 |
| CDS | contig00043 | 7376 | 6660 | -2 | - | 717 | Leucyl/phenylalanyl-tRNA--protein transferase (EC 2.3.2.6) |
| CDS | contig00043 | 8080 | 7373 | -1 | - | 708 | Leucyl/phenylalanyl-tRNA--protein transferase (EC 2.3.2.6) |
| CDS | contig00043 | 8098 | 8568 | 1 | + | 471 | FIG106692: Outer membrane lipoprotein |
| CDS | contig00043 | 9893 | 8652 | -2 | - | 1242 | Response regulator |
| CDS | contig00043 | 11061 | 9952 | -3 | - | 1110 | Thioredoxin reductase (EC 1.8.1.9) |
| CDS | contig00043 | 12201 | 11086 | -3 | - | 1116 | Alanine dehydrogenase (EC 1.4.1.1) |
| CDS | contig00043 | 12352 | 12843 | 1 | + | 492 | Leucine-responsive regulatory protein, regulator for leucine (or lrp) regulon and high-affinity branched-chain amino acid transport system |
| CDS | contig00043 | 13033 | 15537 | 1 | + | 2505 | Cell division protein FtsK |
| CDS | contig00043 | 15603 | 16223 | 3 | + | 621 | Outer membrane lipoprotein carrier protein LolA |
| CDS | contig00043 | 16297 | 17628 | 1 | + | 1332 | FIG065221: Holliday junction DNA helicase |
| CDS | contig00043 | 17909 | 19198 | 2 | + | 1290 | Seryl-tRNA synthetase (EC 6.1.1.11) |
| CDS | contig00043 | 19374 | 19865 | 3 | + | 492 | Colicin V production protein |
| CDS | contig00043 | 19884 | 21404 | 3 | + | 1521 | Amidophosphoribosyltransferase (EC 2.4.2.14) |
| CDS | contig00043 | 22924 | 21494 | -1 | - | 1431 | 6-phospho-beta-glucosidase ascB (EC 3.2.1.86) |
| CDS | contig00043 | 24410 | 22956 | -2 | - | 1455 | PTS system, arbutin-, cellobiose-, and salicin-specific IIBC component (EC 2.7.1.69) |
| CDS | contig00043 | 24673 | 25674 | 1 | + | 1002 | AscBF operon repressor |
| CDS | contig00043 | 25768 | 26526 | 1 | + | 759 | tRNA-(ms[2]io[6]A)-hydroxylase (EC 1.-.-.-) |
| CDS | contig00043 | 27450 | 26665 | -3 | - | 786 | Peptide transport system ATP-binding protein SapF |
| CDS | contig00043 | 28531 | 27533 | -1 | - | 999 | Peptide transport system ATP-binding protein SapD |
| CDS | contig00043 | 29504 | 28611 | -2 | - | 894 | Peptide transport system permease protein SapC |
| CDS | contig00043 | 30553 | 29594 | -1 | - | 960 | Peptide transport system permease protein SapB |
| CDS | contig00043 | 32210 | 30615 | -2 | - | 1596 | Dipeptide-binding ABC transporter, periplasmic substrate-binding component (TC 3.A.1.5.2) |
| CDS | contig00043 | 33363 | 32347 | -3 | - | 1017 | Psp operon transcriptional activator |
| CDS | contig00043 | 33584 | 34264 | 2 | + | 681 | Phage shock protein A |
| CDS | contig00043 | 34268 | 34504 | 2 | + | 237 | Phage shock protein B |
| CDS | contig00043 | 34611 | 35015 | 3 | + | 405 | Phage shock protein C |
| CDS | contig00043 | 35088 | 36494 | 3 | + | 1407 | Conserved protein YcjX with nucleoside triphosphate hydrolase domain |
| CDS | contig00043 | 36544 | 37587 | 1 | + | 1044 | Membrane protein YcjF |
| CDS | contig00043 | 37754 | 38548 | 2 | + | 795 | Phenylalanine-4-hydroxylase (EC 1.14.16.1) |
| CDS | contig00043 | 38590 | 38928 | 1 | + | 339 | Pterin-4-alpha-carbinolamine dehydratase (EC 4.2.1.96) |
| CDS | contig00043 | 39071 | 40618 | 2 | + | 1548 | Transcriptional repressor protein TyrR |
| CDS | contig00043 | 40841 | 41359 | 2 | + | 519 | Uncharacterized protein YfiR precursor |
| CDS | contig00043 | 41356 | 42597 | 1 | + | 1242 | Inner membrane protein YfiN |
| CDS | contig00043 | 42631 | 43119 | 1 | + | 489 | Integral membrane protein YfiB |
| CDS | contig00043 | 43341 | 43153 | -3 | - | 189 | hypothetical protein |
| CDS | contig00043 | 43369 | 44751 | 1 | + | 1383 | Radical SAM family protein HutW, similar to coproporphyrinogen III oxidase, oxygen-independent, associated with heme uptake |
| CDS | contig00043 | 46223 | 44808 | -2 | - | 1416 | Exodeoxyribonuclease VII large subunit (EC 3.1.11.6) |
| CDS | contig00043 | 46137 | 46304 | 3 | + | 168 | hypothetical protein |
| CDS | contig00043 | 47187 | 46279 | -3 | - | 909 | Permease of the drug/metabolite transporter (DMT) superfamily |
| CDS | contig00043 | 47564 | 50770 | 2 | + | 3207 | Alpha-amylase precursor (EC 3.2.1.1) |
| CDS | contig00043 | 51570 | 52205 | 3 | + | 636 | Transporter |
| CDS | contig00043 | 52530 | 52366 | -3 | - | 165 | hypothetical protein |
| CDS | contig00043 | 52600 | 53934 | 1 | + | 1335 | Inosine-5&#39;-monophosphate dehydrogenase (EC 1.1.1.205) |
| CDS | contig00043 | 54019 | 55608 | 1 | + | 1590 | GMP synthase [glutamine-hydrolyzing] (EC 6.3.5.2) |
| CDS | contig00043 | 55927 | 56100 | 1 | + | 174 | hypothetical protein |
| CDS | contig00044 | 1728 | 25 | -3 | - | 1704 | N-carbamoylputrescine amidase (EC 3.5.1.53) |
| CDS | contig00044 | 2962 | 1865 | -1 | - | 1098 | Agmatine deiminase (EC 3.5.3.12) |
| CDS | contig00044 | 3108 | 4019 | 3 | + | 912 | Transcriptional regulator, LysR family |
| CDS | contig00044 | 4598 | 4933 | 2 | + | 336 | DNA methylase, putative |
| CDS | contig00044 | 5645 | 5079 | -2 | - | 567 | Transcriptional regulator, TetR family |
| CDS | contig00044 | 5813 | 6850 | 2 | + | 1038 | NADH-dependent flavin oxidoreductase, Oye family |
| CDS | contig00044 | 7044 | 7970 | 3 | + | 927 | Transcriptional regulator, MarR family / Aspartate N-acetyltransferase (EC 2.3.1.17) |
| CDS | contig00044 | 7967 | 8428 | 2 | + | 462 | PhnO protein |
| CDS | contig00044 | 9059 | 8610 | -2 | - | 450 | Histone acetyltransferase HPA2 and related acetyltransferases |
| CDS | contig00044 | 9471 | 9133 | -3 | - | 339 | FIG00362288: hypothetical protein |
| CDS | contig00044 | 11226 | 9640 | -3 | - | 1587 | Peptide chain release factor 3 |
| CDS | contig00044 | 13335 | 11473 | -3 | - | 1863 | GGDEF domain protein |
| CDS | contig00044 | 13531 | 14535 | 1 | + | 1005 | GGDEF domain protein |
| CDS | contig00044 | 16002 | 14548 | -3 | - | 1455 | Inner membrane protein |
| CDS | contig00044 | 17711 | 16098 | -2 | - | 1614 | Putative regulator protein |
| CDS | contig00044 | 17926 | 18357 | 1 | + | 432 | hypothetical protein |
| CDS | contig00044 | 18433 | 18894 | 1 | + | 462 | Ribosomal-protein-S18p-alanine acetyltransferase (EC 2.3.1.-) |
| CDS | contig00044 | 19509 | 18967 | -3 | - | 543 | hypothetical protein |
| CDS | contig00044 | 19690 | 21480 | 1 | + | 1791 | Long-chain-fatty-acid--CoA ligase (EC 6.2.1.3) |
| CDS | contig00044 | 21632 | 22363 | 2 | + | 732 | hypothetical protein |
| CDS | contig00044 | 23657 | 22443 | -2 | - | 1215 | FIG00361437: hypothetical protein |
| CDS | contig00044 | 24195 | 23887 | -3 | - | 309 | Uncharacterized protein YgeA of aspartate/glutamate/hydantoin racemase family |
| CDS | contig00044 | 24579 | 24247 | -3 | - | 333 | Uncharacterized protein YgeA of aspartate/glutamate/hydantoin racemase family |
| CDS | contig00044 | 25287 | 24697 | -3 | - | 591 | Transporter, LysE family |
| CDS | contig00044 | 26010 | 25390 | -3 | - | 621 | Transporter, LysE family |
| CDS | contig00044 | 26105 | 27481 | 2 | + | 1377 | Transcriptional regulator, GntR family domain / Aspartate aminotransferase (EC 2.6.1.1) |
| CDS | contig00044 | 27509 | 27973 | 2 | + | 465 | hypothetical protein |
| CDS | contig00044 | 29390 | 28077 | -2 | - | 1314 | Sodium-dependent transporter |
| CDS | contig00044 | 31137 | 29824 | -3 | - | 1314 | Sodium-dependent transporter |
| CDS | contig00044 | 32186 | 31347 | -2 | - | 840 | Isopenicillin N synthase |
| CDS | contig00044 | 32253 | 32411 | 3 | + | 159 | hypothetical protein |
| CDS | contig00044 | 32387 | 32950 | 2 | + | 564 | ATPase involved in DNA repair |
| CDS | contig00044 | 33826 | 33005 | -1 | - | 822 | FIG00361885: hypothetical protein |
| CDS | contig00044 | 35299 | 34061 | -1 | - | 1239 | Multidrug efflux transporter, MFS family |
| CDS | contig00044 | 35448 | 36071 | 3 | + | 624 | FIG00361977: hypothetical protein |
| CDS | contig00044 | 36381 | 36857 | 3 | + | 477 | Transcriptional regulator, AsnC family |
| CDS | contig00044 | 37311 | 36994 | -3 | - | 318 | FIG00361441: hypothetical protein |
| CDS | contig00044 | 37699 | 37460 | -1 | - | 240 | hypothetical protein |
| CDS | contig00044 | 38151 | 37726 | -3 | - | 426 | Organic hydroperoxide resistance transcriptional regulator |
| CDS | contig00044 | 38327 | 39331 | 2 | + | 1005 | Putative oxidoreductase YncB |
| CDS | contig00044 | 39510 | 39950 | 3 | + | 441 | Putative Mut family protein |
| CDS | contig00044 | 40124 | 41266 | 2 | + | 1143 | Chromate transport protein ChrA |
| CDS | contig00044 | 41404 | 42273 | 1 | + | 870 | Uncharacterized protein ygiV |
| CDS | contig00044 | 42729 | 42343 | -3 | - | 387 | Peptide methionine sulfoxide reductase MsrB (EC 1.8.4.12) |
| CDS | contig00044 | 44924 | 42786 | -2 | - | 2139 | Glycogen debranching enzyme (EC 3.2.1.-) |
| CDS | contig00044 | 47101 | 44924 | -1 | - | 2178 | 1,4-alpha-glucan (glycogen) branching enzyme, GH-13-type (EC 2.4.1.18) |
| CDS | contig00044 | 49373 | 47184 | -2 | - | 2190 | 4-alpha-glucanotransferase (amylomaltase) (EC 2.4.1.25) |
| CDS | contig00044 | 49720 | 50715 | 1 | + | 996 | NAD-dependent glyceraldehyde-3-phosphate dehydrogenase (EC 1.2.1.12) |
| CDS | contig00044 | 50821 | 51681 | 1 | + | 861 | Aldose 1-epimerase family protein YeaD |
| CDS | contig00044 | 52825 | 51752 | -1 | - | 1074 | Quinolinate synthetase (EC 2.5.1.72) |
| CDS | contig00044 | 52976 | 52839 | -2 | - | 138 | hypothetical protein |
| CDS | contig00044 | 52968 | 55073 | 3 | + | 2106 | Methyl-accepting chemotaxis transducer |
| CDS | contig00045 | 1303 | 47 | -1 | - | 1257 | NADP-dependent malic enzyme (EC 1.1.1.40) |
| CDS | contig00045 | 2916 | 1483 | -3 | - | 1434 | Predicted nucleoside-diphosphate-sugar epimerases |
| CDS | contig00045 | 3248 | 3033 | -2 | - | 216 | LSU ribosomal protein L31p @ LSU ribosomal protein L31p, zinc-dependent |
| CDS | contig00045 | 3429 | 5603 | 3 | + | 2175 | Helicase PriA essential for oriC/DnaA-independent DNA replication |
| CDS | contig00045 | 7893 | 6775 | -3 | - | 1119 | Outer membrane protein A precursor |
| CDS | contig00045 | 9686 | 8154 | -2 | - | 1533 | Threonine dehydratase biosynthetic (EC 4.3.1.19) |
| CDS | contig00045 | 11550 | 9751 | -3 | - | 1800 | Dihydroxy-acid dehydratase (EC 4.2.1.9) |
| CDS | contig00045 | 12683 | 11739 | -2 | - | 945 | Branched-chain amino acid aminotransferase (EC 2.6.1.42) |
| CDS | contig00045 | 12953 | 12696 | -2 | - | 258 | Acetolactate synthase small subunit (EC 2.2.1.6) |
| CDS | contig00045 | 14596 | 12950 | -1 | - | 1647 | Acetolactate synthase large subunit (EC 2.2.1.6) |
| CDS | contig00045 | 14597 | 14881 | 2 | + | 285 | hypothetical protein |
| CDS | contig00045 | 14981 | 16498 | 2 | + | 1518 | MG(2+) CHELATASE FAMILY PROTEIN / ComM-related protein |
| CDS | contig00045 | 16631 | 17530 | 2 | + | 900 | Acyltransferase family protein |
| CDS | contig00045 | 17538 | 18494 | 3 | + | 957 | 1-acyl-sn-glycerol-3-phosphate acyltransferase (EC 2.3.1.51) |
| CDS | contig00045 | 19643 | 19002 | -2 | - | 642 | Nudix-related transcriptional regulator NrtR |
| CDS | contig00045 | 19727 | 20362 | 2 | + | 636 | Nicotinamidase (EC 3.5.1.19) |
| CDS | contig00045 | 20464 | 21642 | 1 | + | 1179 | Nicotinate phosphoribosyltransferase (EC 2.4.2.11) |
| CDS | contig00045 | 22325 | 21717 | -2 | - | 609 | Periplasmic thiol:disulfide interchange protein DsbA |
| CDS | contig00045 | 23524 | 22535 | -1 | - | 990 | YihE protein, a ser/thr kinase implicated in LPS synthesis and Cpx signalling |
| CDS | contig00045 | 25047 | 23599 | -3 | - | 1449 | Type cbb3 cytochrome oxidase biogenesis protein CcoG, involved in Cu oxidation |
| CDS | contig00045 | 25508 | 25245 | -2 | - | 264 | Protein yihD |
| CDS | contig00045 | 25940 | 25539 | -2 | - | 402 | Phytochrome-like protein; Cph2 |
| CDS | contig00045 | 26691 | 25915 | -3 | - | 777 | Phytochrome-like protein; Cph2 |
| CDS | contig00045 | 27234 | 26845 | -3 | - | 390 | Cu(I)-responsive transcriptional regulator |
| CDS | contig00045 | 29660 | 27231 | -2 | - | 2430 | Lead, cadmium, zinc and mercury transporting ATPase (EC 3.6.3.3) (EC 3.6.3.5); Copper-translocating P-type ATPase (EC 3.6.3.4) |
| CDS | contig00045 | 30493 | 29840 | -1 | - | 654 | Orotate phosphoribosyltransferase (EC 2.4.2.10) |
| CDS | contig00045 | 31551 | 30835 | -3 | - | 717 | Ribonuclease PH (EC 2.7.7.56) |
| CDS | contig00045 | 31710 | 32561 | 3 | + | 852 | Protein YicC |
| CDS | contig00045 | 33623 | 32745 | -2 | - | 879 | Permease of the drug/metabolite transporter (DMT) superfamily |
| CDS | contig00045 | 35003 | 33735 | -2 | - | 1269 | 3-deoxy-D-manno-octulosonic-acid transferase (EC 2.-.-.-) |
| CDS | contig00045 | 36102 | 35005 | -3 | - | 1098 | ADP-heptose--lipooligosaccharide heptosyltransferase II (EC 2.4.1.-) |
| CDS | contig00045 | 36385 | 37218 | 1 | + | 834 | Lipopolysaccharide biosynthesis glycosyltransferase |
| CDS | contig00045 | 37514 | 37362 | -2 | - | 153 | hypothetical protein |
| CDS | contig00045 | 38602 | 37559 | -1 | - | 1044 | ADP-heptose--lipooligosaccharide heptosyltransferase II (EC 2.4.1.-) |
| CDS | contig00045 | 38824 | 39306 | 1 | + | 483 | Phosphopantetheine adenylyltransferase (EC 2.7.7.3) |
| CDS | contig00045 | 40252 | 39380 | -1 | - | 873 | putative virK protein |
| CDS | contig00045 | 41241 | 40429 | -3 | - | 813 | Formamidopyrimidine-DNA glycosylase (EC 3.2.2.23) |
| CDS | contig00045 | 41711 | 41256 | -2 | - | 456 | FIG00361539: hypothetical protein |
| CDS | contig00045 | 41969 | 41802 | -2 | - | 168 | LSU ribosomal protein L33p @ LSU ribosomal protein L33p, zinc-independent |
| CDS | contig00045 | 43028 | 42354 | -2 | - | 675 | DNA repair protein RadC |
| CDS | contig00045 | 43176 | 44378 | 3 | + | 1203 | Phosphopantothenoylcysteine decarboxylase (EC 4.1.1.36) / Phosphopantothenoylcysteine synthetase (EC 6.3.2.5) |
| CDS | contig00045 | 44455 | 44913 | 1 | + | 459 | Deoxyuridine 5&#39;-triphosphate nucleotidohydrolase (EC 3.6.1.23) |
| CDS | contig00045 | 44984 | 45580 | 2 | + | 597 | Transcriptional regulator SlmA, TetR family |
| CDS | contig00045 | 46053 | 45874 | -3 | - | 180 | hypothetical protein |
| CDS | contig00045 | 46087 | 46923 | 1 | + | 837 | Small-conductance mechanosensitive channel |
| CDS | contig00045 | 47251 | 47039 | -1 | - | 213 | FIG00362354: hypothetical protein |
| CDS | contig00045 | 47649 | 49298 | 3 | + | 1650 | Glucose-6-phosphate isomerase (EC 5.3.1.9) |
| CDS | contig00045 | 50943 | 49462 | -3 | - | 1482 | Ketol-acid reductoisomerase (EC 1.1.1.86) |
| CDS | contig00045 | 51137 | 52030 | 2 | + | 894 | HTH-type transcriptional regulator IlvY |
| CDS | contig00045 | 52138 | 52329 | 1 | + | 192 | hypothetical protein |
| CDS | contig00045 | 52420 | 54993 | 1 | + | 2574 | Protein acetyltransferase |
| CDS | contig00046 | 3599 | 3171 | -2 | - | 429 | Lactoylglutathione lyase and related lyases |
| CDS | contig00046 | 4572 | 3979 | -3 | - | 594 | lipoprotein, putative |
| CDS | contig00046 | 5051 | 4683 | -2 | - | 369 | Arginine/ornithine antiporter ArcD |
| CDS | contig00046 | 5232 | 5453 | 3 | + | 222 | conserved protein of unknown function |
| CDS | contig00046 | 6164 | 5568 | -2 | - | 597 | FMN-dependent NADH-azoreductase |
| CDS | contig00046 | 6274 | 7203 | 1 | + | 930 | Transcriptional regulator |
| CDS | contig00046 | 8108 | 7254 | -2 | - | 855 | Protein involved in catabolism of external DNA |
| CDS | contig00046 | 8563 | 8102 | -1 | - | 462 | hypothetical protein |
| CDS | contig00046 | 10466 | 8556 | -2 | - | 1911 | Glutathione-regulated potassium-efflux system ATP-binding protein |
| CDS | contig00046 | 10392 | 10526 | 3 | + | 135 | hypothetical protein |
| CDS | contig00046 | 10614 | 11024 | 3 | + | 411 | CBS domain protein |
| CDS | contig00046 | 11352 | 12452 | 3 | + | 1101 | outer membrane porin protein |
| CDS | contig00046 | 12788 | 13732 | 2 | + | 945 | Chemotaxis protein CheV (EC 2.7.3.-) |
| CDS | contig00046 | 14427 | 13819 | -3 | - | 609 | Arylesterase precursor (EC 3.1.1.2) |
| CDS | contig00046 | 14485 | 15195 | 1 | + | 711 | Predicted ABC-type transport system, ATPase component |
| CDS | contig00046 | 15200 | 17701 | 2 | + | 2502 | FIG00809136: hypothetical protein |
| CDS | contig00046 | 18649 | 17744 | -1 | - | 906 | Cell division inhibitor |
| CDS | contig00046 | 18895 | 20436 | 1 | + | 1542 | Rtn protein |
| CDS | contig00046 | 21681 | 20500 | -3 | - | 1182 | Aromatic-amino-acid aminotransferase (EC 2.6.1.57) |
| CDS | contig00046 | 22948 | 21881 | -1 | - | 1068 | Aminopeptidase Y (Arg, Lys, Leu preference) (EC 3.4.11.15) |
| CDS | contig00046 | 23209 | 23922 | 1 | + | 714 | tRNA (guanine46-N7-)-methyltransferase (EC 2.1.1.33) |
| CDS | contig00046 | 23949 | 24281 | 3 | + | 333 | FIG002060: uncharacterized protein YggL |
| CDS | contig00046 | 24378 | 25298 | 3 | + | 921 | Glutaminase (EC 3.5.1.2) |
| CDS | contig00046 | 25337 | 26620 | 2 | + | 1284 | Putative thiosulfate sulfurtransferase ynjE (EC 2.8.1.1) |
| CDS | contig00046 | 26868 | 26755 | -3 | - | 114 | hypothetical protein |
| CDS | contig00046 | 26867 | 27010 | 2 | + | 144 | Hypothetical lipoprotein ygdR precursor |
| CDS | contig00046 | 30626 | 27066 | -2 | - | 3561 | FIG00361300: hypothetical protein |
| CDS | contig00046 | 30630 | 30749 | 3 | + | 120 | hypothetical protein |
| CDS | contig00046 | 32001 | 30724 | -3 | - | 1278 | Proline iminopeptidase (EC 3.4.11.5) |
| CDS | contig00046 | 32238 | 32372 | 3 | + | 135 | hypothetical protein |
| CDS | contig00046 | 32529 | 32395 | -3 | - | 135 | hypothetical protein |
| CDS | contig00046 | 32624 | 32749 | 2 | + | 126 | hypothetical protein |
| CDS | contig00046 | 33352 | 33687 | 1 | + | 336 | Ribonuclease E inhibitor RraB |
| CDS | contig00046 | 35729 | 33756 | -2 | - | 1974 | GGDEF family protein |
| CDS | contig00046 | 35896 | 36390 | 1 | + | 495 | FIG00361799: hypothetical protein |
| CDS | contig00046 | 36891 | 36490 | -3 | - | 402 | FIG00361887: hypothetical protein |
| CDS | contig00046 | 37145 | 36894 | -2 | - | 252 | FIG00361611: hypothetical protein |
| CDS | contig00046 | 37398 | 37150 | -3 | - | 249 | FIG00361340: hypothetical protein |
| CDS | contig00046 | 38300 | 37395 | -2 | - | 906 | Phosphatidylserine decarboxylase (EC 4.1.1.65) |
| CDS | contig00046 | 39331 | 38297 | -1 | - | 1035 | Ribosome small subunit-stimulated GTPase EngC |
| CDS | contig00046 | 39459 | 39968 | 3 | + | 510 | 3&#39;-to-5&#39; oligoribonuclease (orn) |
| CDS | contig00047 | 954 | 745 | -3 | - | 210 | Cold shock protein CspA |
| CDS | contig00047 | 1774 | 1250 | -1 | - | 525 | hypothetical protein |
| CDS | contig00047 | 4111 | 2009 | -1 | - | 2103 | Methyl-accepting chemotaxis transducer |
| CDS | contig00047 | 4375 | 5790 | 1 | + | 1416 | Glutamyl-tRNA synthetase (EC 6.1.1.17) |
| CDS | contig00048 | 3639 | 1432 | -3 | - | 2208 | Extracellular and/or outer membrane deoxyribonuclease NucH/SO1066 |
| CDS | contig00048 | 6210 | 3943 | -3 | - | 2268 | putative ORF-3 protein |
| CDS | contig00049 | 65 | 979 | 2 | + | 915 | 1-aminocyclopropane-1-carboxylate deaminase (EC 3.5.99.7) |
| CDS | contig00049 | 2895 | 976 | -3 | - | 1920 | MSHA biogenesis protein MshH |
| CDS | contig00049 | 3469 | 4944 | 1 | + | 1476 | Rtn protein |
| CDS | contig00049 | 5096 | 6193 | 2 | + | 1098 | Ca2+/H+ antiporter |
| CDS | contig00049 | 7982 | 6450 | -2 | - | 1533 | Histidine ammonia-lyase (EC 4.3.1.3) |
| CDS | contig00049 | 9687 | 7984 | -3 | - | 1704 | Urocanate hydratase (EC 4.2.1.49) |
| CDS | contig00049 | 10671 | 9733 | -3 | - | 939 | Formiminoglutamase (EC 3.5.3.8) |
| CDS | contig00049 | 11899 | 10664 | -1 | - | 1236 | Imidazolonepropionase (EC 3.5.2.7) |
| CDS | contig00049 | 12760 | 12056 | -1 | - | 705 | Histidine utilization repressor |
| CDS | contig00049 | 13430 | 12771 | -2 | - | 660 | Carbonic anhydrase (EC 4.2.1.1) |
| CDS | contig00049 | 14412 | 13558 | -3 | - | 855 | RNA polymerase sigma factor RpoH |
| CDS | contig00049 | 15818 | 14865 | -2 | - | 954 | Cell division protein FtsX |
| CDS | contig00049 | 16486 | 15818 | -1 | - | 669 | Cell division transporter, ATP-binding protein FtsE (TC 3.A.5.1.1) |
| CDS | contig00049 | 18559 | 16709 | -1 | - | 1851 | Signal recognition particle receptor protein FtsY (=alpha subunit) (TC 3.A.5.1.1) |
| CDS | contig00049 | 18670 | 19299 | 1 | + | 630 | 16S rRNA (guanine(966)-N(2))-methyltransferase (EC 2.1.1.171) |
| CDS | contig00049 | 19408 | 19722 | 1 | + | 315 | FIG00362082: hypothetical protein |
| CDS | contig00049 | 20121 | 19756 | -3 | - | 366 | ATPase of the AAA+ class |
| CDS | contig00049 | 20761 | 20141 | -1 | - | 621 | Unsaturated fatty acid biosythesis repressor FabR, TetR family |
| CDS | contig00049 | 20911 | 22029 | 1 | + | 1119 | Fatty acid desaturase (EC 1.14.19.1); Delta-9 fatty acid desaturase (EC 1.14.19.1) |
| CDS | contig00049 | 23751 | 22498 | -3 | - | 1254 | Cardiolipin synthetase (EC 2.7.8.-) |
| CDS | contig00049 | 23827 | 23988 | 1 | + | 162 | hypothetical protein |
| CDS | contig00049 | 25415 | 23994 | -2 | - | 1422 | Transglycosylase, Slt family |
| CDS | contig00049 | 25934 | 25632 | -2 | - | 303 | FIG00361519: hypothetical protein |
| CDS | contig00049 | 26109 | 25945 | -3 | - | 165 | Twin-arginine translocation protein TatA |
| CDS | contig00049 | 26403 | 27380 | 3 | + | 978 | Transcriptional regulator, LysR family |
| CDS | contig00049 | 28040 | 27441 | -2 | - | 600 | FIG01056702: hypothetical protein |
| CDS | contig00049 | 28387 | 28142 | -1 | - | 246 | FIG00362006: hypothetical protein |
| CDS | contig00049 | 28595 | 29878 | 2 | + | 1284 | Sodium/glutamate symport protein |
| CDS | contig00049 | 30208 | 29894 | -1 | - | 315 | FIG00362154: hypothetical protein |
| CDS | contig00049 | 31272 | 30370 | -3 | - | 903 | Transcriptional regulator, LysR family |
| CDS | contig00049 | 31370 | 32422 | 2 | + | 1053 | Membrane fusion component of tripartite multidrug resistance system |
| CDS | contig00049 | 32458 | 33975 | 1 | + | 1518 | Inner membrane component of tripartite multidrug resistance system |
| CDS | contig00049 | 34662 | 34036 | -3 | - | 627 | Putative inner membrane protein |
| CDS | contig00049 | 34847 | 34993 | 2 | + | 147 | hypothetical protein |
| CDS | contig00049 | 37646 | 35082 | -2 | - | 2565 | TRAP-type uncharacterized transport system, fused permease component |
| CDS | contig00049 | 38714 | 37734 | -2 | - | 981 | TRAP transporter solute receptor, TAXI family precursor |
| CDS | contig00049 | 41418 | 39253 | -3 | - | 2166 | Protease II (EC 3.4.21.83) |
| CDS | contig00049 | 42138 | 41707 | -3 | - | 432 | CopG protein |
| CDS | contig00049 | 42507 | 42184 | -3 | - | 324 | FIG00361326: hypothetical protein |
| CDS | contig00049 | 43306 | 42584 | -1 | - | 723 | Short-chain dehydrogenase/reductase SDR |
| CDS | contig00049 | 43447 | 44289 | 1 | + | 843 | Hydrogen peroxide-inducible genes activator |
| CDS | contig00049 | 44349 | 44948 | 3 | + | 600 | Glutathione-regulated potassium-efflux system ancillary protein KefG |
| CDS | contig00049 | 45019 | 46875 | 1 | + | 1857 | Glutathione-regulated potassium-efflux system protein KefB |
| CDS | contig00049 | 46872 | 47258 | 3 | + | 387 | FIG002082: Protein SirB2 |
| CDS | contig00049 | 47544 | 48530 | 3 | + | 987 | Transcriptional factor MdcH |
| CDS | contig00050 | 438 | 596 | 3 | + | 159 | hypothetical protein |
| CDS | contig00050 | 864 | 989 | 3 | + | 126 | hypothetical protein |
| CDS | contig00050 | 2243 | 1035 | -2 | - | 1209 | Integrase |
| CDS | contig00050 | 5972 | 2859 | -2 | - | 3114 | RND multidrug efflux transporter; Acriflavin resistance protein |
| CDS | contig00050 | 7141 | 5987 | -1 | - | 1155 | RND efflux system, membrane fusion protein CmeA |
| CDS | contig00050 | 7300 | 8028 | 1 | + | 729 | Two-component response regulator |
| CDS | contig00050 | 8025 | 9104 | 3 | + | 1080 | Two-component hybrid sensor and regulator |
| CDS | contig00050 | 10884 | 9250 | -3 | - | 1635 | Sulfate permease |
| CDS | contig00050 | 11635 | 11060 | -1 | - | 576 | DJ-1/YajL/PfpI superfamily, includes chaperone protein YajL (former ThiJ), parkinsonism-associated protein DJ-1, peptidases PfpI, Hsp31 |
| CDS | contig00050 | 12613 | 11666 | -1 | - | 948 | 2-dehydropantoate 2-reductase (EC 1.1.1.169) |
| CDS | contig00050 | 12972 | 13454 | 3 | + | 483 | FIG001943: hypothetical protein YajQ |
| CDS | contig00050 | 14363 | 13557 | -2 | - | 807 | Outer membrane protein OmpK |
| CDS | contig00050 | 15923 | 14520 | -2 | - | 1404 | AmpG permease |
| CDS | contig00050 | 16338 | 16009 | -3 | - | 330 | hypothetical protein |
| CDS | contig00050 | 17004 | 16453 | -3 | - | 552 | Peptidyl-prolyl cis-trans isomerase PpiA precursor (EC 5.2.1.8) |
| CDS | contig00050 | 17594 | 17019 | -2 | - | 576 | Hypothetical lipoprotein YajG precursor |
| CDS | contig00050 | 18749 | 17616 | -2 | - | 1134 | 23S rRNA (guanine-N-2-) -methyltransferase rlmG (EC 2.1.1.-) |
| CDS | contig00050 | 18951 | 19265 | 3 | + | 315 | Cell division protein BolA |
| CDS | contig00050 | 19686 | 21029 | 3 | + | 1344 | Na(+)-translocating NADH-quinone reductase subunit A (EC 1.6.5.-) |
| CDS | contig00050 | 21033 | 22262 | 3 | + | 1230 | Na(+)-translocating NADH-quinone reductase subunit B (EC 1.6.5.-) |
| CDS | contig00050 | 22252 | 23040 | 1 | + | 789 | Na(+)-translocating NADH-quinone reductase subunit C (EC 1.6.5.-) |
| CDS | contig00050 | 23033 | 23665 | 2 | + | 633 | Na(+)-translocating NADH-quinone reductase subunit D (EC 1.6.5.-) |
| CDS | contig00050 | 23669 | 24265 | 2 | + | 597 | Na(+)-translocating NADH-quinone reductase subunit E (EC 1.6.5.-) |
| CDS | contig00050 | 24283 | 25506 | 1 | + | 1224 | Na(+)-translocating NADH-quinone reductase subunit F (EC 1.6.5.-) |
| CDS | contig00050 | 25621 | 26547 | 1 | + | 927 | Thiamin biosynthesis lipoprotein ApbE |
| CDS | contig00050 | 26559 | 26783 | 3 | + | 225 | Probable exported or periplasmic protein in ApbE locus |
| CDS | contig00050 | 27077 | 26865 | -2 | - | 213 | hypothetical protein |
| CDS | contig00050 | 27297 | 28349 | 3 | + | 1053 | DNA polymerase IV (EC 2.7.7.7) |
| CDS | contig00050 | 28417 | 30552 | 1 | + | 2136 | Methyl-accepting chemotaxis protein |
| CDS | contig00050 | 30839 | 31759 | 2 | + | 921 | Glycine cleavage system transcriptional activator GcvA |
| CDS | contig00050 | 31772 | 32407 | 2 | + | 636 | Predicted hydrolase of the alpha/beta-hydrolase fold |
| CDS | contig00050 | 32451 | 32837 | 3 | + | 387 | COG2363 |
| CDS | contig00050 | 32830 | 33924 | 1 | + | 1095 | LSU rRNA 2&#39;-O-methyl-C2498 methyltransferase RlmM |
| CDS | contig00050 | 34434 | 33991 | -3 | - | 444 | hypothetical protein |
| CDS | contig00050 | 35562 | 34555 | -3 | - | 1008 | Isocitrate dehydrogenase [NAD] (EC 1.1.1.41) |
| CDS | contig00050 | 36502 | 35705 | -1 | - | 798 | DNA polymerase I (EC 2.7.7.7) |
| CDS | contig00050 | 37983 | 36628 | -3 | - | 1356 | Decarboxylase family protein |
| CDS | contig00050 | 37972 | 38160 | 1 | + | 189 | hypothetical protein |
| CDS | contig00050 | 39734 | 38142 | -2 | - | 1593 | GGDEF domain family protein |
| CDS | contig00050 | 42099 | 39829 | -3 | - | 2271 | FIG00920775: hypothetical protein |
| CDS | contig00050 | 43064 | 42216 | -2 | - | 849 | NADPH dependent preQ0 reductase (EC 1.7.1.13) |
| CDS | contig00050 | 43170 | 43718 | 3 | + | 549 | Syd protein |
| CDS | contig00050 | 44590 | 43805 | -1 | - | 786 | Zn-ribbon-containing, possibly nucleic-acid-binding protein |
| CDS | contig00050 | 44905 | 44600 | -1 | - | 306 | hypothetical protein |
| CDS | contig00050 | 45323 | 45087 | -2 | - | 237 | hypothetical protein |
| CDS | contig00050 | 45679 | 45383 | -1 | - | 297 | FIG00361630: hypothetical protein |
| CDS | contig00050 | 46872 | 45748 | -3 | - | 1125 | FIG01199667: hypothetical protein |
| CDS | contig00050 | 46974 | 47309 | 3 | + | 336 | Hypothetical protein YqcC (clustered with tRNA pseudouridine synthase C) |
| CDS | contig00050 | 47306 | 48085 | 2 | + | 780 | tRNA pseudouridine synthase C (EC 4.2.1.70) |
| CDS | contig00050 | 48270 | 48710 | 3 | + | 441 | Hypothetical flavoprotein YqcA (clustered with tRNA pseudouridine synthase C) |
| CDS | contig00051 | 112 | 2001 | 1 | + | 1890 | Phage protein |
| CDS | contig00051 | 5978 | 2454 | -2 | - | 3525 | DNA primase (EC 2.7.7.-) |
| CDS | contig00051 | 6611 | 6354 | -2 | - | 258 | hypothetical protein |
| CDS | contig00051 | 7378 | 7860 | 1 | + | 483 | Phage protein |
| CDS | contig00051 | 8133 | 8519 | 3 | + | 387 | hypothetical protein |
| CDS | contig00051 | 8584 | 8784 | 1 | + | 201 | hypothetical protein |
| CDS | contig00051 | 8781 | 9068 | 3 | + | 288 | hypothetical protein |
| CDS | contig00051 | 9044 | 10039 | 2 | + | 996 | DNA recombination-dependent growth factor C |
| CDS | contig00051 | 10149 | 10265 | 3 | + | 117 | hypothetical protein |
| CDS | contig00051 | 10316 | 10471 | 2 | + | 156 | hypothetical protein |
| CDS | contig00051 | 10891 | 11946 | 1 | + | 1056 | Adenine-specific methyltransferase (EC 2.1.1.72) |
| CDS | contig00051 | 11973 | 12284 | 3 | + | 312 | hypothetical protein |
| CDS | contig00051 | 12308 | 12766 | 2 | + | 459 | FIG00641052: hypothetical protein |
| CDS | contig00051 | 14937 | 16718 | 3 | + | 1782 | Phage terminase, large subunit |
| CDS | contig00051 | 16756 | 16959 | 1 | + | 204 | hypothetical protein |
| CDS | contig00051 | 16956 | 18530 | 3 | + | 1575 | Phage portal protein |
| CDS | contig00051 | 18571 | 19842 | 1 | + | 1272 | Head-tail preconnector protein GP5 |
| CDS | contig00051 | 19845 | 20189 | 3 | + | 345 | hypothetical protein |
| CDS | contig00051 | 20189 | 21235 | 2 | + | 1047 | Phage major capsid protein #Fam0010 |
| CDS | contig00051 | 21293 | 21667 | 2 | + | 375 | hypothetical protein |
| CDS | contig00051 | 21720 | 22055 | 3 | + | 336 | hypothetical protein |
| CDS | contig00051 | 22069 | 22698 | 1 | + | 630 | hypothetical protein |
| CDS | contig00051 | 22707 | 23234 | 3 | + | 528 | Phage protein |
| CDS | contig00051 | 23249 | 23821 | 2 | + | 573 | Baseplate assembly protein V |
| CDS | contig00051 | 23832 | 24173 | 3 | + | 342 | Phage baseplate assembly protein |
| CDS | contig00051 | 24214 | 25176 | 1 | + | 963 | Phage-related baseplate assembly protein |
| CDS | contig00051 | 25508 | 25786 | 2 | + | 279 | Phage tail fibers |
| CDS | contig00051 | 25783 | 28302 | 1 | + | 2520 | Phage tail fiber protein |
| CDS | contig00051 | 28661 | 28828 | 2 | + | 168 | hypothetical protein |
| CDS | contig00051 | 28832 | 30283 | 2 | + | 1452 | Phage tail sheath monomer |
| CDS | contig00051 | 30283 | 30786 | 1 | + | 504 | Phage major tail tube protein |
| CDS | contig00051 | 30876 | 31472 | 3 | + | 597 | Phage protein |
| CDS | contig00051 | 31587 | 34382 | 3 | + | 2796 | Phage tail length tape-measure protein |
| CDS | contig00051 | 34430 | 34855 | 2 | + | 426 | hypothetical protein |
| CDS | contig00051 | 34852 | 35058 | 1 | + | 207 | hypothetical protein |
| CDS | contig00051 | 35106 | 36083 | 3 | + | 978 | Phage-related protein |
| CDS | contig00051 | 36137 | 36664 | 2 | + | 528 | hypothetical protein |
| CDS | contig00051 | 38101 | 36863 | -1 | - | 1239 | Mobile element protein |
| CDS | contig00051 | 38333 | 38103 | -2 | - | 231 | hypothetical protein |
| CDS | contig00051 | 39252 | 38635 | -3 | - | 618 | Exodeoxyribonuclease VIII (EC 3.1.11.-) |
| CDS | contig00051 | 39839 | 39249 | -2 | - | 591 | hypothetical protein |
| CDS | contig00051 | 41546 | 39882 | -2 | - | 1665 | C-5 cytosine-specific DNA methylase |
| CDS | contig00051 | 41815 | 41639 | -1 | - | 177 | hypothetical protein |
| CDS | contig00051 | 41967 | 41812 | -3 | - | 156 | hypothetical protein |
| CDS | contig00051 | 42328 | 42167 | -1 | - | 162 | hypothetical protein |
| CDS | contig00051 | 42486 | 42860 | 3 | + | 375 | hypothetical protein |
| CDS | contig00051 | 43294 | 43608 | 1 | + | 315 | Transcriptional activator protein LuxR |
| CDS | contig00051 | 43809 | 43630 | -3 | - | 180 | hypothetical protein |
| CDS | contig00051 | 44253 | 43891 | -3 | - | 363 | hypothetical protein |
| CDS | contig00051 | 44419 | 44264 | -1 | - | 156 | hypothetical protein |
| CDS | contig00051 | 45706 | 44480 | -1 | - | 1227 | Chromosome (plasmid) partitioning protein ParB |
| CDS | contig00051 | 46962 | 45790 | -3 | - | 1173 | Chromosome (plasmid) partitioning protein ParA |
| CDS | contig00052 | 552 | 220 | -3 | - | 333 | Uncharacterized conserved protein |
| CDS | contig00052 | 759 | 2117 | 3 | + | 1359 | Guanine-hypoxanthine permease |
| CDS | contig00052 | 2346 | 3416 | 3 | + | 1071 | Phosphoenolpyruvate-dihydroxyacetone phosphotransferase (EC 2.7.1.121), dihydroxyacetone binding subunit DhaK |
| CDS | contig00052 | 3426 | 4055 | 3 | + | 630 | Phosphoenolpyruvate-dihydroxyacetone phosphotransferase (EC 2.7.1.121), ADP-binding subunit DhaL |
| CDS | contig00052 | 4057 | 4773 | 1 | + | 717 | Phosphoenolpyruvate-dihydroxyacetone phosphotransferase (EC 2.7.1.121), subunit DhaM; DHA-specific IIA component / DHA-specific phosphocarrier protein HPr / DHA-specific EI component |
| CDS | contig00052 | 6771 | 4834 | -3 | - | 1938 | Phosphoenolpyruvate-dihydroxyacetone phosphotransferase operon regulatory protein DhaR |
| CDS | contig00052 | 7701 | 6808 | -3 | - | 894 | Glycine cleavage system transcriptional activator |
| CDS | contig00052 | 7842 | 8870 | 3 | + | 1029 | Agmatine deiminase (EC 3.5.3.12) |
| CDS | contig00052 | 9003 | 9902 | 3 | + | 900 | N-carbamoylputrescine amidase (3.5.1.53) |
| CDS | contig00052 | 9899 | 11008 | 2 | + | 1110 | Spermidine/putrescine-binding periplasmic protein |
| CDS | contig00052 | 11025 | 12146 | 3 | + | 1122 | Agmatine deiminase (EC 3.5.3.12) |
| CDS | contig00052 | 12941 | 12237 | -2 | - | 705 | Short-chain alcohol dehydrogenase associated with acetoin utilization |
| CDS | contig00052 | 14393 | 13308 | -2 | - | 1086 | Putative membrane protein |
| CDS | contig00052 | 15779 | 14403 | -2 | - | 1377 | Membrane-associated phospholipid phosphatase |
| CDS | contig00052 | 15952 | 16329 | 1 | + | 378 | hypothetical protein |
| CDS | contig00052 | 17816 | 16410 | -2 | - | 1407 | Phage tail length tape-measure protein 1 |
| CDS | contig00052 | 18036 | 18353 | 3 | + | 318 | hypothetical protein |
| CDS | contig00052 | 20821 | 19373 | -1 | - | 1449 | Catalase (EC 1.11.1.6) |
| CDS | contig00052 | 20974 | 21462 | 1 | + | 489 | GNAT family acetyltransferase YhhY |
| CDS | contig00052 | 21459 | 22316 | 3 | + | 858 | Transcriptional regulator, AraC family |
| CDS | contig00052 | 23285 | 22386 | -2 | - | 900 | Permease of the drug/metabolite transporter (DMT) superfamily |
| CDS | contig00052 | 23862 | 23395 | -3 | - | 468 | FIG00361313: hypothetical protein |
| CDS | contig00052 | 23994 | 25550 | 3 | + | 1557 | diguanylate cyclase (GGDEF domain) with PAS/PAC sensor |
| CDS | contig00052 | 26233 | 25574 | -1 | - | 660 | Phosphoserine phosphatase (EC 3.1.3.3) |
| CDS | contig00052 | 27306 | 26299 | -3 | - | 1008 | Ferric iron ABC transporter, ATP-binding protein |
| CDS | contig00052 | 28089 | 27313 | -3 | - | 777 | Thiamin ABC transporter, transmembrane component |
| CDS | contig00052 | 28978 | 28082 | -1 | - | 897 | ABC transporter permease protein |
| CDS | contig00052 | 29811 | 28978 | -3 | - | 834 | FIG01199621: hypothetical protein |
| CDS | contig00052 | 30871 | 29822 | -1 | - | 1050 | Ferric iron ABC transporter, iron-binding protein |
| CDS | contig00052 | 30927 | 31055 | 3 | + | 129 | hypothetical protein |
| CDS | contig00052 | 31052 | 31864 | 2 | + | 813 | 2-aminoethylphosphonate uptake and metabolism regulator |
| CDS | contig00052 | 32675 | 31911 | -2 | - | 765 | FIG00361869: hypothetical protein |
| CDS | contig00052 | 34033 | 33119 | -1 | - | 915 | LysR-family transcriptional regulator YhaJ |
| CDS | contig00052 | 34291 | 34623 | 1 | + | 333 | hypothetical protein |
| CDS | contig00052 | 34654 | 35340 | 1 | + | 687 | Protein yhhW |
| CDS | contig00052 | 35424 | 35816 | 3 | + | 393 | Inner membrane protein YqjF |
| CDS | contig00052 | 36049 | 39405 | 1 | + | 3357 | Exodeoxyribonuclease V gamma chain (EC 3.1.11.5) |
| CDS | contig00052 | 39405 | 43019 | 3 | + | 3615 | Exodeoxyribonuclease V beta chain (EC 3.1.11.5) |
| CDS | contig00052 | 43028 | 45082 | 2 | + | 2055 | Exodeoxyribonuclease V alpha chain (EC 3.1.11.5) |
| CDS | contig00053 | 1006 | 1923 | 1 | + | 918 | Mobile element protein |
| CDS | contig00053 | 4247 | 2625 | -2 | - | 1623 | 3-methylmercaptopropionyl-CoA ligase (DmdB) |
| CDS | contig00053 | 4571 | 5110 | 2 | + | 540 | FIG01200701: possible membrane protein |
| CDS | contig00053 | 8463 | 5266 | -3 | - | 3198 | Extracellular and/or outer membrane deoxyribonuclease NucH/SO1066 |
| CDS | contig00053 | 9038 | 10159 | 2 | + | 1122 | Putative periplasmic substrate-binding transport protein |
| CDS | contig00053 | 10261 | 11313 | 1 | + | 1053 | Iron(III) dicitrate transport system permease protein FecD (TC 3.A.1.14.1) |
| CDS | contig00053 | 11306 | 12076 | 2 | + | 771 | Iron(III) dicitrate transport ATP-binding protein FecE (TC 3.A.1.14.1) |
| CDS | contig00053 | 12132 | 13361 | 3 | + | 1230 | Probable conserved integral membrane transport protein |
| CDS | contig00053 | 14446 | 13562 | -1 | - | 885 | Arginine/ornithine antiporter ArcD |
| CDS | contig00053 | 15015 | 14458 | -3 | - | 558 | YaeQ protein |
| CDS | contig00053 | 15344 | 15204 | -2 | - | 141 | hypothetical protein |
| CDS | contig00053 | 17757 | 15607 | -3 | - | 2151 | Enoyl-CoA hydratase (EC 4.2.1.17) / Delta(3)-cis-delta(2)-trans-enoyl-CoA isomerase (EC 5.3.3.8) / 3-hydroxyacyl-CoA dehydrogenase (EC 1.1.1.35) / 3-hydroxybutyryl-CoA epimerase (EC 5.1.2.3) |
| CDS | contig00053 | 19064 | 17754 | -2 | - | 1311 | 3-ketoacyl-CoA thiolase (EC 2.3.1.16) @ Acetyl-CoA acetyltransferase (EC 2.3.1.9) |
| CDS | contig00053 | 19248 | 20198 | 3 | + | 951 | MoxR-like ATPase in aerotolerance operon |
| CDS | contig00053 | 20227 | 21117 | 1 | + | 891 | hypothetical protein PA3071 |
| CDS | contig00053 | 21102 | 21659 | 3 | + | 558 | hypothetical protein |
| CDS | contig00053 | 21656 | 22618 | 2 | + | 963 | BatA (Bacteroides aerotolerance operon) |
| CDS | contig00053 | 22640 | 24100 | 2 | + | 1461 | TPR domain protein in aerotolerance operon |
| CDS | contig00053 | 24097 | 25641 | 1 | + | 1545 | BatD |
| CDS | contig00053 | 25747 | 26262 | 1 | + | 516 | RNA polymerase sigma-70 factor, ECF subfamily |
| CDS | contig00053 | 26255 | 26998 | 2 | + | 744 | FIG00920272: hypothetical protein |
| CDS | contig00053 | 27407 | 28678 | 2 | + | 1272 | Long-chain fatty acid transport protein precursor |
| CDS | contig00053 | 28930 | 30186 | 1 | + | 1257 | Long-chain fatty acid transport protein |
| CDS | contig00053 | 30303 | 31544 | 3 | + | 1242 | Predicted signal transduction protein |
| CDS | contig00053 | 32772 | 31606 | -3 | - | 1167 | 3-ketoacyl-CoA thiolase [isoleucine degradation] (EC 2.3.1.16) |
| CDS | contig00053 | 32905 | 33303 | 1 | + | 399 | Predicted transcriptional regulator LiuR of leucine degradation pathway, MerR family |
| CDS | contig00053 | 34655 | 33411 | -2 | - | 1245 | Proton/glutamate symport protein @ Sodium/glutamate symport protein |
| CDS | contig00053 | 34924 | 35208 | 1 | + | 285 | Uncharacterized protein conserved in bacteria |
| CDS | contig00053 | 35365 | 36747 | 1 | + | 1383 | L-serine dehydratase (EC 4.3.1.17) |
| CDS | contig00053 | 37032 | 38402 | 3 | + | 1371 | L-serine dehydratase (EC 4.3.1.17) |
| CDS | contig00053 | 39502 | 38564 | -1 | - | 939 | Membrane-bound lytic murein transglycosylase B (EC 3.2.1.-) |
| CDS | contig00053 | 39818 | 39540 | -2 | - | 279 | Protein YcgL |
| CDS | contig00053 | 39925 | 40650 | 1 | + | 726 | Septum site-determining protein MinC |
| CDS | contig00053 | 40669 | 41481 | 1 | + | 813 | Septum site-determining protein MinD |
| CDS | contig00053 | 41484 | 41753 | 3 | + | 270 | Cell division topological specificity factor MinE |
| CDS | contig00053 | 41934 | 42260 | 3 | + | 327 | FIG00361526: hypothetical protein |
| CDS | contig00053 | 42245 | 43021 | 2 | + | 777 | Monofunctional biosynthetic peptidoglycan transglycosylase (EC 2.4.2.-) |
| CDS | contig00053 | 43306 | 43629 | 1 | + | 324 | putative cytoplasmic protein |
| CDS | contig00053 | 43986 | 43675 | -3 | - | 312 | FIG00361774: hypothetical protein |
| CDS | contig00053 | 44857 | 43988 | -1 | - | 870 | FIG00362319: hypothetical protein |
| CDS | contig00054 | 673 | 272 | -1 | - | 402 | DNA-binding protein H-NS |
| CDS | contig00054 | 1319 | 2818 | 2 | + | 1500 | Mobile element protein |
| CDS | contig00054 | 2805 | 3545 | 3 | + | 741 | Mobile element protein |
| CDS | contig00054 | 3807 | 4580 | 3 | + | 774 | Error-prone, lesion bypass DNA polymerase V (UmuC) |
| CDS | contig00054 | 4772 | 5968 | 2 | + | 1197 | DnaJ domain protein |
| CDS | contig00054 | 7591 | 6167 | -1 | - | 1425 | hypothetical protein |
| CDS | contig00054 | 8402 | 9151 | 2 | + | 750 | 27kDa outer membrane protein |
| CDS | contig00054 | 10351 | 9326 | -1 | - | 1026 | UDP-glucose 4-epimerase (EC 5.1.3.2) |
| CDS | contig00054 | 11456 | 10437 | -2 | - | 1020 | Flavodoxin reductases (ferredoxin-NADPH reductases) family 1 |
| CDS | contig00054 | 13206 | 11557 | -3 | - | 1650 | Hydroxylamine reductase (EC 1.7.-.-) |
| CDS | contig00054 | 13192 | 13311 | 1 | + | 120 | hypothetical protein |
| CDS | contig00054 | 13910 | 13443 | -2 | - | 468 | Non-specific DNA-binding protein Dps / Iron-binding ferritin-like antioxidant protein / Ferroxidase (EC 1.16.3.1) |
| CDS | contig00054 | 14584 | 14042 | -1 | - | 543 | FIG002577: Putative lipoprotein precursor |
| CDS | contig00054 | 15138 | 14596 | -3 | - | 543 | FIG026291: Hypothetical periplasmic protein |
| CDS | contig00054 | 15313 | 15095 | -1 | - | 219 | FIG00361487: hypothetical protein |
| CDS | contig00054 | 15789 | 15310 | -3 | - | 480 | FIG033155: Hypothetical protein |
| CDS | contig00054 | 17048 | 15792 | -2 | - | 1257 | S-adenosyl-L-methionine dependent methyltransferase, similar to cyclopropane-fatty-acyl-phospholipid synthase |
| CDS | contig00054 | 17895 | 17155 | -3 | - | 741 | FIG001571: Hypothetical protein |
| CDS | contig00054 | 19190 | 17931 | -2 | - | 1260 | COG2907: Amine oxidase, flavin-containing |
| CDS | contig00054 | 19921 | 19187 | -1 | - | 735 | Oxidoreductase, short-chain dehydrogenase/reductase family (EC 1.1.1.-) |
| CDS | contig00054 | 20346 | 19918 | -3 | - | 429 | FIG002994: Putative transcriptional regulator |
| CDS | contig00054 | 21772 | 20348 | -1 | - | 1425 | Deoxyribodipyrimidine photolyase (EC 4.1.99.3) |
| CDS | contig00054 | 22656 | 21769 | -3 | - | 888 | Transcriptional regulator, MerR family, associated with photolyase |
| CDS | contig00054 | 23740 | 22796 | -1 | - | 945 | COG1683: Uncharacterized conserved protein / FIG143828: Hypothetical protein YbgA |
| CDS | contig00054 | 24543 | 23914 | -3 | - | 630 | FIG00545237: hypothetical protein |
| CDS | contig00054 | 25276 | 24722 | -1 | - | 555 | hypothetical protein |
| CDS | contig00054 | 26195 | 25365 | -2 | - | 831 | Gll2047 protein |
| CDS | contig00054 | 26942 | 26343 | -2 | - | 600 | HAD superfamily hydrolase |
| CDS | contig00054 | 27278 | 27111 | -2 | - | 168 | hypothetical protein |
| CDS | contig00054 | 27306 | 28478 | 3 | + | 1173 | Chorismate mutase I (EC 5.4.99.5) / Prephenate dehydratase (EC 4.2.1.51) |
| CDS | contig00054 | 29485 | 29060 | -1 | - | 426 | Flagellar protein FlgP |
| CDS | contig00054 | 30194 | 29574 | -2 | - | 621 | Flagellar protein FlgO |
| CDS | contig00054 | 30459 | 31610 | 3 | + | 1152 | Flagellar protein FlgT |
| CDS | contig00054 | 31981 | 32931 | 1 | + | 951 | Mycobacteriophage Barnyard protein gp56 |
| CDS | contig00054 | 33026 | 34015 | 2 | + | 990 | Protein gp47, recombination-related [Bacteriophage A118] |
| CDS | contig00054 | 34012 | 35004 | 1 | + | 993 | FIG00965493: hypothetical protein |
| CDS | contig00054 | 35208 | 35005 | -3 | - | 204 | Transcriptional regulator, XRE family |
| CDS | contig00054 | 35517 | 36356 | 3 | + | 840 | hypothetical protein |
| CDS | contig00054 | 36666 | 36815 | 3 | + | 150 | hypothetical protein |
| CDS | contig00054 | 36999 | 36871 | -3 | - | 129 | hypothetical protein |
| CDS | contig00054 | 37547 | 37972 | 2 | + | 426 | hypothetical protein |
| CDS | contig00054 | 38195 | 38392 | 2 | + | 198 | hypothetical protein |
| CDS | contig00054 | 38385 | 38642 | 3 | + | 258 | hypothetical protein |
| CDS | contig00054 | 38754 | 39251 | 3 | + | 498 | DNA repair protein RadC |
| CDS | contig00054 | 39406 | 41412 | 1 | + | 2007 | FIG01059502: hypothetical protein |
| CDS | contig00054 | 41421 | 43277 | 3 | + | 1857 | FIG01059502: hypothetical protein |
| CDS | contig00055 | 828 | 478 | -3 | - | 351 | probable iron binding protein from the HesB_IscA_SufA family |
| CDS | contig00055 | 1610 | 918 | -2 | - | 693 | FIG00361456: hypothetical protein |
| CDS | contig00055 | 3092 | 1698 | -2 | - | 1395 | H(+)/Cl(-) exchange transporter ClcA |
| CDS | contig00055 | 3302 | 4588 | 2 | + | 1287 | Glutamate-1-semialdehyde aminotransferase (EC 5.4.3.8) |
| CDS | contig00055 | 4789 | 5601 | 1 | + | 813 | N-linked glycosylation glycosyltransferase PglG |
| CDS | contig00055 | 6550 | 5648 | -1 | - | 903 | Integral membrane protein |
| CDS | contig00055 | 7493 | 6810 | -2 | - | 684 | Putative membrane protein |
| CDS | contig00055 | 7693 | 8211 | 1 | + | 519 | hypothetical protein |
| CDS | contig00055 | 9042 | 8254 | -3 | - | 789 | Transcriptional regulator |
| CDS | contig00055 | 9292 | 9684 | 1 | + | 393 | Spermidine export protein MdtJ |
| CDS | contig00055 | 9672 | 9992 | 3 | + | 321 | Spermidine export protein MdtI |
| CDS | contig00055 | 10484 | 10083 | -2 | - | 402 | FIG00361475: hypothetical protein |
| CDS | contig00055 | 12848 | 10527 | -2 | - | 2322 | Multimodular transpeptidase-transglycosylase (EC 2.4.1.129) (EC 3.4.-.-) |
| CDS | contig00055 | 15467 | 12972 | -2 | - | 2496 | ATP-dependent helicase HrpB |
| CDS | contig00055 | 15610 | 16155 | 1 | + | 546 | 2&#39;-5&#39; RNA ligase |
| CDS | contig00055 | 16152 | 16895 | 3 | + | 744 | Sugar/maltose fermentation stimulation protein homolog |
| CDS | contig00055 | 17015 | 17464 | 2 | + | 450 | C4-type zinc finger protein, DksA/TraR family |
| CDS | contig00055 | 17574 | 18476 | 3 | + | 903 | glutamyl-Q-tRNA synthetase |
| CDS | contig00055 | 18649 | 20067 | 1 | + | 1419 | Poly(A) polymerase (EC 2.7.7.19) |
| CDS | contig00055 | 20078 | 20575 | 2 | + | 498 | 2-amino-4-hydroxy-6-hydroxymethyldihydropteridine pyrophosphokinase (EC 2.7.6.3) |
| CDS | contig00055 | 20596 | 21390 | 1 | + | 795 | 3-methyl-2-oxobutanoate hydroxymethyltransferase (EC 2.1.2.11) |
| CDS | contig00055 | 21403 | 22341 | 1 | + | 939 | Pantoate--beta-alanine ligase (EC 6.3.2.1) |
| CDS | contig00055 | 22362 | 22709 | 3 | + | 348 | Antibiotic biosynthesis monooxygenase |
| CDS | contig00055 | 23544 | 22771 | -3 | - | 774 | ABC-type multidrug transport system, permease component |
| CDS | contig00055 | 24449 | 23541 | -2 | - | 909 | Hypothetical ABC transporter ATP-binding protein yadG |
| CDS | contig00055 | 25101 | 24571 | -3 | - | 531 | Hypoxanthine-guanine phosphoribosyltransferase (EC 2.4.2.8) |
| CDS | contig00055 | 25369 | 25917 | 1 | + | 549 | Quorum-sensing regulator of virulence HapR |
| CDS | contig00055 | 26602 | 25991 | -1 | - | 612 | 3-polyprenyl-4-hydroxybenzoate carboxy-lyase UbiX (EC 4.1.1.-) |
| CDS | contig00055 | 27969 | 26599 | -3 | - | 1371 | UDP-N-acetylmuramate:L-alanyl-gamma-D-glutamyl-meso-diaminopimelate ligase (EC 6.3.2.-) |
| CDS | contig00055 | 28203 | 28733 | 3 | + | 531 | Inorganic pyrophosphatase (EC 3.6.1.1) |
| CDS | contig00055 | 29109 | 28759 | -3 | - | 351 | hypothetical protein |
| CDS | contig00055 | 29233 | 30111 | 1 | + | 879 | FIG00361597: hypothetical protein |
| CDS | contig00055 | 30317 | 30859 | 2 | + | 543 | Protein ytfJ precursor |
| CDS | contig00055 | 30930 | 31193 | 3 | + | 264 | hypothetical protein |
| CDS | contig00055 | 31230 | 31826 | 3 | + | 597 | Predicted zinc-binding protein |
| CDS | contig00055 | 31831 | 32517 | 1 | + | 687 | ABC-type antimicrobial peptide transport system, ATPase component |
| CDS | contig00055 | 32576 | 33823 | 2 | + | 1248 | ABC-type antimicrobial peptide transport system, permease component |
| CDS | contig00055 | 33855 | 34331 | 3 | + | 477 | FIG003461: hypothetical protein |
| CDS | contig00055 | 34452 | 35300 | 3 | + | 849 | FIG00919648: hypothetical protein |
| CDS | contig00055 | 35538 | 37166 | 3 | + | 1629 | Aerotaxis sensor receptor protein |
| CDS | contig00055 | 38245 | 39156 | 1 | + | 912 | Alpha/beta superfamily hydrolase |
| CDS | contig00056 | 2743 | 752 | -1 | - | 1992 | Transketolase (EC 2.2.1.1) |
| CDS | contig00056 | 2771 | 2983 | 2 | + | 213 | hypothetical protein |
| CDS | contig00056 | 3084 | 4235 | 3 | + | 1152 | S-adenosylmethionine synthetase (EC 2.5.1.6) |
| CDS | contig00056 | 4304 | 4843 | 2 | + | 540 | Protein sprT |
| CDS | contig00056 | 4917 | 5609 | 3 | + | 693 | Endonuclease I precursor (EC 3.1.21.1) @ Extracellular deoxyribonuclease Dns (EC 3.1.21.-) |
| CDS | contig00056 | 5739 | 6470 | 3 | + | 732 | Ribosomal RNA small subunit methyltransferase E (EC 2.1.1.-) |
| CDS | contig00056 | 6593 | 7543 | 2 | + | 951 | Glutathione synthetase (EC 6.3.2.3) |
| CDS | contig00056 | 7687 | 8241 | 1 | + | 555 | UPF0301 protein YqgE |
| CDS | contig00056 | 8326 | 8748 | 1 | + | 423 | Putative Holliday junction resolvase YggF |
| CDS | contig00056 | 9785 | 8745 | -2 | - | 1041 | Putative LysR-family transcriptional regulator YidZ |
| CDS | contig00056 | 10962 | 9796 | -3 | - | 1167 | Putative transport protein |
| CDS | contig00056 | 12406 | 11255 | -1 | - | 1152 | Beta-lactamase (EC 3.5.2.6) |
| CDS | contig00056 | 14016 | 12586 | -3 | - | 1431 | Sodium/alanine symporter |
| CDS | contig00056 | 14722 | 14597 | -1 | - | 126 | hypothetical protein |
| CDS | contig00056 | 14693 | 15691 | 2 | + | 999 | Transaldolase (EC 2.2.1.2) |
| CDS | contig00056 | 15872 | 16051 | 2 | + | 180 | FIG00361528: hypothetical protein |
| CDS | contig00056 | 16693 | 16148 | -1 | - | 546 | FIG01200137: hypothetical protein |
| CDS | contig00056 | 17534 | 16854 | -2 | - | 681 | Uracil-DNA glycosylase, family 1 |
| CDS | contig00056 | 18143 | 18523 | 2 | + | 381 | Pyruvate formate-lyase (EC 2.3.1.54) |
| CDS | contig00056 | 19886 | 18603 | -2 | - | 1284 | Aerobic C4-dicarboxylate transporter for fumarate, L-malate, D-malate, succunate |
| CDS | contig00056 | 21030 | 20176 | -3 | - | 855 | 2-Keto-3-deoxy-D-manno-octulosonate-8-phosphate synthase (EC 2.5.1.55) |
| CDS | contig00056 | 21842 | 21042 | -2 | - | 801 | FIG002708: Protein SirB1 |
| CDS | contig00056 | 22237 | 21854 | -1 | - | 384 | FIG002082: Protein sirB2 |
| CDS | contig00056 | 23113 | 22280 | -1 | - | 834 | Protein-N(5)-glutamine methyltransferase PrmC, methylates polypeptide chain release factors RF1 and RF2 |
| CDS | contig00056 | 24201 | 23113 | -3 | - | 1089 | Peptide chain release factor 1 |
| CDS | contig00056 | 25511 | 24252 | -2 | - | 1260 | Glutamyl-tRNA reductase (EC 1.2.1.70) |
| CDS | contig00056 | 25637 | 26215 | 2 | + | 579 | Outer membrane lipoprotein LolB precursor |
| CDS | contig00056 | 27387 | 28334 | 3 | + | 948 | Ribose-phosphate pyrophosphokinase (EC 2.7.6.1) |
| CDS | contig00056 | 28584 | 29180 | 3 | + | 597 | Peptidyl-tRNA hydrolase (EC 3.1.1.29) |
| CDS | contig00056 | 29312 | 30403 | 2 | + | 1092 | GTP-binding and nucleic acid-binding protein YchF |
| CDS | contig00056 | 33549 | 31717 | -3 | - | 1833 | Putative transport protein |
| CDS | contig00056 | 33820 | 34152 | 1 | + | 333 | RNA-binding domain protein |
| CDS | contig00056 | 34240 | 35160 | 1 | + | 921 | Lipase, GDXG family |
| CDS | contig00056 | 35222 | 35608 | 2 | + | 387 | Sll0939 protein |
| CDS | contig00056 | 37077 | 35713 | -3 | - | 1365 | multidrug transporter, putative |
| CDS | contig00056 | 37242 | 37868 | 3 | + | 627 | Putative phosphatase YqaB |
| CDS | contig00057 | 1133 | 96 | -2 | - | 1038 | FIG116849: hypothetical protein |
| CDS | contig00057 | 2683 | 1118 | -1 | - | 1566 | FIG131328: Predicted ATP-dependent endonuclease of the OLD family |
| CDS | contig00057 | 4095 | 3730 | -3 | - | 366 | hypothetical protein |
| CDS | contig00057 | 4123 | 5181 | 1 | + | 1059 | hypothetical protein |
| CDS | contig00057 | 5267 | 5554 | 2 | + | 288 | hypothetical protein |
| CDS | contig00057 | 5935 | 5657 | -1 | - | 279 | hypothetical protein |
| CDS | contig00057 | 6998 | 6216 | -2 | - | 783 | ABC-type amino acid transport, signal transduction systems, periplasmic component/domain |
| CDS | contig00057 | 7624 | 7130 | -1 | - | 495 | Putative membrane protein |
| CDS | contig00057 | 8628 | 7621 | -3 | - | 1008 | Putative membrane protein |
| CDS | contig00057 | 9559 | 8621 | -1 | - | 939 | Putative membrane protein |
| CDS | contig00057 | 10334 | 9777 | -2 | - | 558 | Probable transmembrane protein |
| CDS | contig00057 | 10917 | 10588 | -3 | - | 330 | Translation initiation factor SUI1-related protein |
| CDS | contig00057 | 11037 | 11264 | 3 | + | 228 | Ferrous iron transport protein A |
| CDS | contig00057 | 11261 | 13531 | 2 | + | 2271 | Ferrous iron transport protein B |
| CDS | contig00057 | 13578 | 13820 | 3 | + | 243 | Ferrous iron transport protein C |
| CDS | contig00057 | 15003 | 13882 | -3 | - | 1122 | Twitching motility protein PilT |
| CDS | contig00057 | 15431 | 15075 | -2 | - | 357 | FIG00361280: hypothetical protein |
| CDS | contig00057 | 15544 | 16275 | 1 | + | 732 | Bacterial extracellular solute-binding protein, family 3 |
| CDS | contig00057 | 16351 | 17358 | 1 | + | 1008 | Vitamin B12 ABC transporter, permease component BtuC |
| CDS | contig00057 | 17345 | 18118 | 2 | + | 774 | Vitamin B12 ABC transporter, ATPase component BtuD |
| CDS | contig00057 | 19394 | 18330 | -2 | - | 1065 | 2-keto-3-deoxy-D-arabino-heptulosonate-7-phosphate synthase I alpha (EC 2.5.1.54) |
| CDS | contig00057 | 19536 | 19916 | 3 | + | 381 | Diacylglycerol kinase (EC 2.7.1.107) |
| CDS | contig00057 | 21227 | 19995 | -2 | - | 1233 | D-3-phosphoglycerate dehydrogenase (EC 1.1.1.95) |
| CDS | contig00057 | 22146 | 21493 | -3 | - | 654 | Ribose 5-phosphate isomerase A (EC 5.3.1.6) |
| CDS | contig00057 | 22886 | 22290 | -2 | - | 597 | 5-formyltetrahydrofolate cyclo-ligase (EC 6.3.3.2) |
| CDS | contig00057 | 24337 | 22991 | -1 | - | 1347 | Methionine transporter MetT |
| CDS | contig00057 | 24648 | 25466 | 3 | + | 819 | 4-hydroxy-tetrahydrodipicolinate reductase (EC 1.17.1.8) |
| CDS | contig00057 | 26232 | 27263 | 3 | + | 1032 | Carbamoyl-phosphate synthase small chain (EC 6.3.5.5) |
| CDS | contig00057 | 27280 | 30504 | 1 | + | 3225 | Carbamoyl-phosphate synthase large chain (EC 6.3.5.5) |
| CDS | contig00057 | 31008 | 32666 | 3 | + | 1659 | 2-isopropylmalate synthase (EC 2.3.3.13) |
| CDS | contig00057 | 32911 | 33816 | 1 | + | 906 | diguanylate cyclase/phosphodiesterase (GGDEF & EAL domains) with PAS/PAC sensor(s) |
| CDS | contig00057 | 33962 | 35125 | 2 | + | 1164 | Putative outer membrane lipoprotein |
| CDS | contig00057 | 35731 | 35615 | -1 | - | 117 | hypothetical protein |
| CDS | contig00058 | 908 | 1993 | 2 | + | 1086 | dTDP-glucose 4,6-dehydratase (EC 4.2.1.46) |
| CDS | contig00058 | 1993 | 2880 | 1 | + | 888 | dTDP-4-dehydrorhamnose reductase (EC 1.1.1.133) |
| CDS | contig00058 | 2995 | 3873 | 1 | + | 879 | Glucose-1-phosphate thymidylyltransferase (EC 2.7.7.24) |
| CDS | contig00058 | 3923 | 4474 | 2 | + | 552 | dTDP-4-dehydrorhamnose 3,5-epimerase (EC 5.1.3.13) |
| CDS | contig00058 | 4476 | 5285 | 3 | + | 810 | O-antigen export system permease protein RfbD |
| CDS | contig00058 | 5269 | 6579 | 1 | + | 1311 | Teichoic acid export ATP-binding protein TagH (EC 3.6.3.40) |
| CDS | contig00058 | 6592 | 9294 | 1 | + | 2703 | Glycosyltransferase |
| CDS | contig00058 | 10369 | 11478 | 1 | + | 1110 | UDP-N-acetylglucosamine 2-epimerase (EC 5.1.3.14) |
| CDS | contig00058 | 11484 | 12560 | 3 | + | 1077 | Glycosyltransferase SypN |
| CDS | contig00058 | 13360 | 14310 | 1 | + | 951 | UDP-glucose 4-epimerase (EC 5.1.3.2) |
| CDS | contig00058 | 14464 | 15345 | 1 | + | 882 | Undecaprenyl-phosphate N-acetylglucosaminyl 1-phosphate transferase (EC 2.7.8.-) |
| CDS | contig00058 | 15933 | 17327 | 3 | + | 1395 | Nucleoside-diphosphate sugar epimerase/dehydratase |
| CDS | contig00058 | 17944 | 18876 | 1 | + | 933 | Undecaprenyl-phosphate N-acetylglucosaminyl 1-phosphate transferase (EC 2.7.8.-) |
| CDS | contig00058 | 19178 | 20425 | 2 | + | 1248 | Membrane protein involved in the export of O-antigen, teichoic acid lipoteichoic acids |
| CDS | contig00058 | 22578 | 23678 | 3 | + | 1101 | Poly(glycerol-phosphate) alpha-glucosyltransferase (EC 2.4.1.52) |
| CDS | contig00058 | 24914 | 25309 | 2 | + | 396 | Serine acetyltransferase (EC 2.3.1.30) |
| CDS | contig00058 | 25293 | 26477 | 3 | + | 1185 | Lipid carrier : UDP-N-acetylgalactosaminyltransferase (EC 2.4.1.-) / Alpha-1,3-N-acetylgalactosamine transferase PglA (EC 2.4.1.-); Putative glycosyltransferase |
| CDS | contig00058 | 26669 | 27784 | 2 | + | 1116 | Polysaccharide export lipoprotein Wza |
| CDS | contig00058 | 28020 | 28448 | 3 | + | 429 | Low molecular weight protein-tyrosine-phosphatase Wzb (EC 3.1.3.48) |
| CDS | contig00058 | 28511 | 30688 | 2 | + | 2178 | Tyrosine-protein kinase Wzc (EC 2.7.10.2) |
| CDS | contig00058 | 31285 | 30725 | -1 | - | 561 | Mobile element protein |
| CDS | contig00058 | 31272 | 33062 | 3 | + | 1791 | Putative outer membrane lipoprotein |
| CDS | contig00058 | 33201 | 34868 | 3 | + | 1668 | Lipid A core-O-antigen ligase |
| CDS | contig00059 | 317 | 180 | -2 | - | 138 | hypothetical protein |
| CDS | contig00059 | 613 | 494 | -1 | - | 120 | hypothetical protein |
| CDS | contig00059 | 1111 | 725 | -1 | - | 387 | Putative threonine efflux protein |
| CDS | contig00059 | 3012 | 2821 | -3 | - | 192 | hypothetical protein |
| CDS | contig00059 | 3730 | 3152 | -1 | - | 579 | Alkylphosphonate utilization operon protein PhnA |
| CDS | contig00059 | 3910 | 5103 | 1 | + | 1194 | SAM-dependent methyltransferases |
| CDS | contig00059 | 5252 | 6736 | 2 | + | 1485 | Chitinase (EC 3.2.1.14) |
| CDS | contig00059 | 6859 | 7224 | 1 | + | 366 | Glyoxalase family protein |
| CDS | contig00059 | 7432 | 7830 | 1 | + | 399 | Gfa-like protein |
| CDS | contig00059 | 7860 | 8087 | 3 | + | 228 | hypothetical protein |
| CDS | contig00059 | 8134 | 8544 | 1 | + | 411 | Bll8152 protein |
| CDS | contig00059 | 9119 | 8634 | -2 | - | 486 | FIG001674: hypothetical protein |
| CDS | contig00059 | 9212 | 10159 | 2 | + | 948 | Protein-N(5)-glutamine methyltransferase PrmB, methylates LSU ribosomal protein L3p |
| CDS | contig00059 | 10176 | 11264 | 3 | + | 1089 | Chorismate synthase (EC 4.2.3.5) |
| CDS | contig00059 | 12161 | 11352 | -2 | - | 810 | extracellular solute-binding protein, family 3 |
| CDS | contig00059 | 12467 | 13873 | 2 | + | 1407 | ATP-dependent RNA helicase RhlE |
| CDS | contig00059 | 14649 | 13960 | -3 | - | 690 | hypothetical protein |
| CDS | contig00059 | 15053 | 14649 | -2 | - | 405 | Uncharacterized protein y4oB |
| CDS | contig00059 | 15225 | 15091 | -3 | - | 135 | hypothetical protein |
| CDS | contig00059 | 16490 | 15297 | -2 | - | 1194 | Tyrosine-specific transport protein |
| CDS | contig00059 | 18111 | 17125 | -3 | - | 987 | Fructose repressor FruR, LacI family |
| CDS | contig00059 | 18419 | 19519 | 2 | + | 1101 | Fructose-specific phosphocarrier protein HPr (EC 2.7.1.69) / PTS system, fructose-specific IIA component (EC 2.7.1.69) |
| CDS | contig00059 | 19519 | 20508 | 1 | + | 990 | 1-phosphofructokinase (EC 2.7.1.56) |
| CDS | contig00059 | 20505 | 22229 | 3 | + | 1725 | PTS system, fructose-specific IIB component (EC 2.7.1.69) / PTS system, fructose-specific IIC component (EC 2.7.1.69) |
| CDS | contig00059 | 22563 | 22312 | -3 | - | 252 | Predicted Fe-S protein |
| CDS | contig00059 | 22735 | 23865 | 1 | + | 1131 | Membrane-bound lytic murein transglycosylase A precursor (EC 3.2.1.-) |
| CDS | contig00059 | 24319 | 23999 | -1 | - | 321 | Ferredoxin |
| CDS | contig00059 | 25450 | 24368 | -1 | - | 1083 | Ribonucleotide reductase of class Ia (aerobic), beta subunit (EC 1.17.4.1) |
| CDS | contig00059 | 27836 | 25581 | -2 | - | 2256 | Ribonucleotide reductase of class Ia (aerobic), alpha subunit (EC 1.17.4.1) |
| CDS | contig00059 | 27816 | 28010 | 3 | + | 195 | hypothetical protein |
| CDS | contig00059 | 29105 | 28437 | -2 | - | 669 | Similar to phosphoglycolate phosphatase, clustered with ubiquinone biosynthesis SAM-dependent O-methyltransferase |
| CDS | contig00059 | 29839 | 29123 | -1 | - | 717 | 3-demethylubiquinone-9 3-methyltransferase (EC 2.1.1.64) |
| CDS | contig00059 | 30039 | 32786 | 3 | + | 2748 | DNA gyrase subunit A (EC 5.99.1.3) |
| CDS | contig00059 | 33030 | 33161 | 3 | + | 132 | hypothetical protein |
| CDS | contig00059 | 33411 | 33271 | -3 | - | 141 | Radical SAM domain protein |
| CDS | contig00059 | 33951 | 33463 | -3 | - | 489 | Radical SAM domain protein |
| CDS | contig00060 | 903 | 1127 | 3 | + | 225 | hypothetical protein |
| CDS | contig00060 | 1137 | 1319 | 3 | + | 183 | hypothetical protein |
| CDS | contig00060 | 1751 | 1939 | 2 | + | 189 | Prevent host death protein, Phd antitoxin # A |
| CDS | contig00060 | 2918 | 4309 | 2 | + | 1392 | Mobile element protein |
| CDS | contig00060 | 5234 | 5103 | -2 | - | 132 | hypothetical protein |
| CDS | contig00060 | 5524 | 6288 | 1 | + | 765 | Mobile element protein |
| CDS | contig00060 | 6499 | 7074 | 1 | + | 576 | Mobile element protein |
| CDS | contig00060 | 7077 | 10043 | 3 | + | 2967 | Mobile element protein |
| CDS | contig00060 | 11584 | 11189 | -1 | - | 396 | hypothetical protein |
| CDS | contig00060 | 12611 | 12129 | -2 | - | 483 | hypothetical protein |
| CDS | contig00060 | 12804 | 14012 | 3 | + | 1209 | hypothetical protein |
| CDS | contig00060 | 14516 | 14034 | -2 | - | 483 | hypothetical protein |
| CDS | contig00060 | 15955 | 15578 | -1 | - | 378 | hypothetical protein |
| CDS | contig00060 | 18222 | 17125 | -3 | - | 1098 | hypothetical protein |
| CDS | contig00060 | 19382 | 18372 | -2 | - | 1011 | hypothetical protein |
| CDS | contig00060 | 21087 | 21518 | 3 | + | 432 | hypothetical protein |
| CDS | contig00060 | 22988 | 21594 | -2 | - | 1395 | FIG00784338: hypothetical protein |
| CDS | contig00060 | 24667 | 23249 | -1 | - | 1419 | FIG00784338: hypothetical protein |
| CDS | contig00060 | 26871 | 27410 | 3 | + | 540 | hypothetical protein |
| CDS | contig00060 | 28806 | 27688 | -3 | - | 1119 | Integrase-like protein |
| CDS | contig00060 | 29048 | 28803 | -2 | - | 246 | hypothetical protein |
| CDS | contig00060 | 29507 | 29346 | -2 | - | 162 | hypothetical protein |
| CDS | contig00060 | 30246 | 29839 | -3 | - | 408 | DNA-binding protein H-NS |
| CDS | contig00060 | 31667 | 30531 | -2 | - | 1137 | Mobile element protein |
| CDS | contig00061 | 18 | 905 | 3 | + | 888 | Mobile element protein |
| CDS | contig00061 | 2985 | 1195 | -3 | - | 1791 | ATP-dependent DNA helicase UvrD/PcrA |
| CDS | contig00061 | 2984 | 3964 | 2 | + | 981 | Endonuclease |
| CDS | contig00061 | 4269 | 4454 | 3 | + | 186 | hypothetical protein |
| CDS | contig00061 | 4728 | 4868 | 3 | + | 141 | hypothetical protein |
| CDS | contig00061 | 6134 | 5748 | -2 | - | 387 | hypothetical protein |
| CDS | contig00061 | 7270 | 6233 | -1 | - | 1038 | hypothetical protein |
| CDS | contig00061 | 8947 | 7919 | -1 | - | 1029 | Mobile element protein |
| CDS | contig00061 | 10062 | 9253 | -3 | - | 810 | Mobile element protein |
| CDS | contig00061 | 10075 | 10992 | 1 | + | 918 | Transposase |
| CDS | contig00061 | 11452 | 12465 | 1 | + | 1014 | Molybdenum cofactor biosynthesis protein MoaA |
| CDS | contig00062 | 102 | 1370 | 3 | + | 1269 | transposase, IS4 |
| CDS | contig00063 | 814 | 59 | -1 | - | 756 | Twin-arginine translocation protein TatC |
| CDS | contig00063 | 1236 | 811 | -3 | - | 426 | Twin-arginine translocation protein TatB |
| CDS | contig00063 | 1491 | 1240 | -3 | - | 252 | Twin-arginine translocation protein TatA |
| CDS | contig00063 | 4238 | 1791 | -2 | - | 2448 | Anaerobic dimethyl sulfoxide reductase chain A (EC 1.8.5.3) |
| CDS | contig00063 | 5222 | 4254 | -2 | - | 969 | Cytochrome c-type protein TorY |
| CDS | contig00063 | 5862 | 6998 | 3 | + | 1137 | Mobile element protein |
| CDS | contig00063 | 7324 | 7830 | 1 | + | 507 | Molybdenum cofactor biosynthesis protein MoaB |
| CDS | contig00063 | 7883 | 8377 | 2 | + | 495 | Molybdenum cofactor biosynthesis protein MoaC |
| CDS | contig00063 | 8374 | 8619 | 1 | + | 246 | Molybdenum cofactor biosynthesis protein MoaD |
| CDS | contig00063 | 8622 | 9101 | 3 | + | 480 | Molybdenum cofactor biosynthesis protein MoaE |
| CDS | contig00063 | 9289 | 10005 | 1 | + | 717 | Molybdenum transport system permease protein ModB (TC 3.A.1.8.1) |
| CDS | contig00063 | 10002 | 11057 | 3 | + | 1056 | Molybdenum transport ATP-binding protein ModC (TC 3.A.1.8.1) |
| CDS | contig00063 | 11887 | 11132 | -1 | - | 756 | Molybdopterin biosynthesis protein MoeB |
| CDS | contig00063 | 13116 | 11884 | -3 | - | 1233 | Molybdopterin biosynthesis protein MoeA |
| CDS | contig00063 | 14250 | 13405 | -3 | - | 846 | Anaerobic dimethyl sulfoxide reductase chain C (EC 1.8.5.3) |
| CDS | contig00063 | 14866 | 14252 | -1 | - | 615 | Anaerobic dimethyl sulfoxide reductase chain B (EC 1.8.5.3) |
| CDS | contig00063 | 17108 | 14877 | -2 | - | 2232 | Anaerobic dimethyl sulfoxide reductase chain A (EC 1.8.5.3) |
| CDS | contig00063 | 17103 | 17243 | 3 | + | 141 | hypothetical protein |
| CDS | contig00064 | 192 | 79 | -3 | - | 114 | hypothetical protein |
| CDS | contig00064 | 494 | 1690 | 2 | + | 1197 | NnrS protein involved in response to NO |
| CDS | contig00064 | 2263 | 1691 | -1 | - | 573 | Transcriptional regulator yidN, Cro/CI family |
| CDS | contig00064 | 2449 | 3528 | 1 | + | 1080 | Benzoate transport protein |
| CDS | contig00064 | 3676 | 4740 | 1 | + | 1065 | FIG00362058: hypothetical protein |
| CDS | contig00064 | 5700 | 4804 | -3 | - | 897 | 5,10-methylenetetrahydrofolate reductase (EC 1.5.1.20) |
| CDS | contig00064 | 6028 | 6819 | 1 | + | 792 | FIG01201438: hypothetical protein |
| CDS | contig00064 | 8343 | 6880 | -3 | - | 1464 | N-Acetyl-D-glucosamine ABC transport system, sugar-binding protein |
| CDS | contig00064 | 10331 | 8352 | -2 | - | 1980 | N-acetylglucosamine regulated methyl-accepting chemotaxis protein |
| CDS | contig00064 | 11142 | 10528 | -3 | - | 615 | Outer membrane protein W precursor |
| CDS | contig00064 | 11465 | 11590 | 2 | + | 126 | hypothetical protein |
| CDS | contig00064 | 14535 | 11707 | -3 | - | 2829 | Excinuclease ABC subunit A |
| CDS | contig00064 | 15266 | 14628 | -2 | - | 639 | Transcriptional regulator VpsT |
| CDS | contig00064 | 15785 | 16354 | 2 | + | 570 | Single-stranded DNA-binding protein |
| CDS | contig00064 | 16516 | 17286 | 1 | + | 771 | ABC-type amino acid transport/signal transduction systems, periplasmic component/domain |
| CDS | contig00064 | 17458 | 17333 | -1 | - | 126 | hypothetical protein |
| CDS | contig00064 | 17751 | 18035 | 3 | + | 285 | hypothetical protein |
| CDS | contig00064 | 18428 | 18637 | 2 | + | 210 | Cold shock protein CspG |
| CDS | contig00064 | 20200 | 18815 | -1 | - | 1386 | sensor histidine kinase |
| CDS | contig00064 | 20933 | 20208 | -2 | - | 726 | TorCAD operon transcriptional regulatory protein TorR |
| CDS | contig00064 | 21130 | 21486 | 1 | + | 357 | FIG00361378: hypothetical protein |
| CDS | contig00064 | 21974 | 23968 | 2 | + | 1995 | Methyl-accepting chemotaxis protein |
| CDS | contig00064 | 24444 | 24031 | -3 | - | 414 | FIG00362460: hypothetical protein |
| CDS | contig00064 | 25576 | 24575 | -1 | - | 1002 | diguanylate cyclase/phosphodiesterase (GGDEF & EAL domains) with PAS/PAC sensor(s) |
| CDS | contig00064 | 25963 | 26301 | 1 | + | 339 | Nitrogen regulatory protein P-II |
| CDS | contig00064 | 26327 | 27568 | 2 | + | 1242 | Ammonium transporter |
| CDS | contig00064 | 28560 | 27628 | -3 | - | 933 | Transcriptional regulator, AraC family |
| CDS | contig00064 | 28618 | 29016 | 1 | + | 399 | Possible glyoxylase family protein (Lactoylglutathione lyase) (EC 4.4.1.5) |
| CDS | contig00065 | 755 | 135 | -2 | - | 621 | FIG00657740: hypothetical protein |
| CDS | contig00065 | 1941 | 973 | -3 | - | 969 | Spermidine synthase (EC 2.5.1.16) |
| CDS | contig00065 | 2276 | 2926 | 2 | + | 651 | DedA protein |
| CDS | contig00065 | 3172 | 4467 | 1 | + | 1296 | ATP-dependent RNA helicase VVA0939 |
| CDS | contig00065 | 4583 | 5218 | 2 | + | 636 | FIG00361452: hypothetical protein |
| CDS | contig00065 | 5475 | 6512 | 3 | + | 1038 | Extracellular protease precursor (EC 3.4.24.-) |
| CDS | contig00065 | 6557 | 6988 | 2 | + | 432 | Extracellular protease precursor (EC 3.4.24.-) |
| CDS | contig00065 | 7179 | 6985 | -3 | - | 195 | hypothetical protein |
| CDS | contig00065 | 7241 | 7819 | 2 | + | 579 | O-methyltransferase family protein |
| CDS | contig00065 | 7882 | 8067 | 1 | + | 186 | hypothetical protein |
| CDS | contig00065 | 8500 | 9273 | 1 | + | 774 | Lysine-arginine-ornithine-binding periplasmic protein precursor (TC 3.A.1.3.1) |
| CDS | contig00065 | 9310 | 9987 | 1 | + | 678 | Cystine ABC transporter, permease protein |
| CDS | contig00065 | 9984 | 10745 | 3 | + | 762 | Cell division transporter, ATP-binding protein FtsE (TC 3.A.5.1.1) |
| CDS | contig00065 | 12246 | 10831 | -3 | - | 1416 | Glutamate synthase [NADPH] small chain (EC 1.4.1.13) |
| CDS | contig00065 | 16716 | 12259 | -3 | - | 4458 | Glutamate synthase [NADPH] large chain (EC 1.4.1.13) |
| CDS | contig00065 | 17330 | 18274 | 2 | + | 945 | COG1242: Predicted Fe-S oxidoreductase |
| CDS | contig00065 | 18664 | 18338 | -1 | - | 327 | hypothetical protein |
| CDS | contig00065 | 18644 | 20788 | 2 | + | 2145 | ABC transporter, transmembrane region:ABC transporter:Peptidase C39, bacteriocin processing |
| CDS | contig00065 | 20781 | 22196 | 3 | + | 1416 | Type I secretion system, membrane fusion protein LapC |
| CDS | contig00065 | 22232 | 23563 | 2 | + | 1332 | Type I secretion system, outer membrane component LapE |
| CDS | contig00065 | 23708 | 24421 | 2 | + | 714 | FIGfam010717 |
| CDS | contig00065 | 24425 | 26350 | 2 | + | 1926 | Membrane bound c-di-GMP receptor LapD |
| CDS | contig00066 | 1967 | 453 | -2 | - | 1515 | Thermostable carboxypeptidase 1 (EC 3.4.17.19) |
| CDS | contig00066 | 2619 | 2038 | -3 | - | 582 | FIG00361883: hypothetical protein |
| CDS | contig00066 | 2884 | 2735 | -1 | - | 150 | hypothetical protein |
| CDS | contig00066 | 3927 | 3040 | -3 | - | 888 | FIG00361813: hypothetical protein |
| CDS | contig00066 | 3998 | 4330 | 2 | + | 333 | FIG00362210: hypothetical protein |
| CDS | contig00066 | 5242 | 4412 | -1 | - | 831 | Putative NAD(P)-dependent oxidoreductase EC-YbbO |
| CDS | contig00066 | 6757 | 5357 | -1 | - | 1401 | N-acetylglucosamine-regulated outer membrane porin |
| CDS | contig00066 | 9376 | 7439 | -1 | - | 1938 | Methyl-accepting chemotaxis protein |
| CDS | contig00066 | 10326 | 9436 | -3 | - | 891 | Permease of the drug/metabolite transporter (DMT) superfamily |
| CDS | contig00066 | 11922 | 10417 | -3 | - | 1506 | Gluconate permease |
| CDS | contig00066 | 12869 | 11919 | -2 | - | 951 | Nucleoside-diphosphate-sugar epimerases |
| CDS | contig00066 | 13678 | 12905 | -1 | - | 774 | Hydroxypyruvate isomerase (EC 5.3.1.22) |
| CDS | contig00066 | 14307 | 13675 | -3 | - | 633 | Ribulose-5-phosphate 4-epimerase and related epimerases and aldolases |
| CDS | contig00066 | 15550 | 14300 | -1 | - | 1251 | FIG00641944: hypothetical protein |
| CDS | contig00066 | 16455 | 15547 | -3 | - | 909 | D-beta-hydroxybutyrate dehydrogenase (EC 1.1.1.30) |
| CDS | contig00066 | 16712 | 17506 | 2 | + | 795 | DeoR family transcriptional regulator probably related to glycerate, glycolaldehyde metabolism |
| CDS | contig00066 | 17707 | 17552 | -1 | - | 156 | hypothetical protein |
| CDS | contig00066 | 18782 | 17724 | -2 | - | 1059 | PQQ-dependent oxidoreductase, gdhB family |
| CDS | contig00066 | 19766 | 18942 | -2 | - | 825 | FIG00732265: hypothetical protein |
| CDS | contig00066 | 20228 | 21553 | 2 | + | 1326 | Integrase |
| CDS | contig00066 | 24401 | 25429 | 2 | + | 1029 | Type I restriction-modification system, specificity subunit S (EC 3.1.21.3) |
| CDS | contig00067 | 1534 | 452 | -1 | - | 1083 | ADP-heptose:LPS heptosyltransferase II |
| CDS | contig00067 | 3006 | 1750 | -3 | - | 1257 | FIG00732228: membrane protein |
| CDS | contig00067 | 3354 | 3067 | -3 | - | 288 | Putative sugar phosphotransferase component II B |
| CDS | contig00067 | 3830 | 3387 | -2 | - | 444 | PTS system, IIA component |
| CDS | contig00067 | 4083 | 5105 | 3 | + | 1023 | Transcriptional regulator, LacI family |
| CDS | contig00067 | 5371 | 5493 | 1 | + | 123 | hypothetical protein |
| CDS | contig00067 | 5662 | 6000 | 1 | + | 339 | FIG00361343: hypothetical protein |
| CDS | contig00067 | 6228 | 6058 | -3 | - | 171 | FIG00361822: hypothetical protein |
| CDS | contig00067 | 6716 | 6285 | -2 | - | 432 | Flagellar biosynthesis protein FliL |
| CDS | contig00067 | 6970 | 7413 | 1 | + | 444 | Chorismate--pyruvate lyase (EC 4.1.3.40) |
| CDS | contig00067 | 7410 | 8273 | 3 | + | 864 | 4-hydroxybenzoate polyprenyltransferase (EC 2.5.1.39) |
| CDS | contig00067 | 10760 | 8337 | -2 | - | 2424 | Glycerol-3-phosphate acyltransferase (EC 2.3.1.15) |
| CDS | contig00067 | 10965 | 11591 | 3 | + | 627 | SOS-response repressor and protease LexA (EC 3.4.21.88) |
| CDS | contig00067 | 12023 | 12232 | 2 | + | 210 | hypothetical protein |
| CDS | contig00067 | 12366 | 13049 | 3 | + | 684 | Cytochrome oxidase biogenesis protein Sco1/SenC/PrrC, putative copper metallochaperone |
| CDS | contig00067 | 13046 | 14434 | 2 | + | 1389 | DNA-damage-inducible protein F |
| CDS | contig00067 | 14930 | 14466 | -2 | - | 465 | tRNA (cytidine(34)-2&#39;-O)-methyltransferase (EC 2.1.1.207) |
| CDS | contig00067 | 16356 | 15001 | -3 | - | 1356 | Copper sensory histidine kinase CpxA |
| CDS | contig00067 | 17072 | 16353 | -2 | - | 720 | Copper-sensing two-component system response regulator CpxR |
| CDS | contig00067 | 17133 | 18044 | 3 | + | 912 | Cobalt-zinc-cadmium resistance protein |
| CDS | contig00067 | 18053 | 18832 | 2 | + | 780 | Serine acetyltransferase (EC 2.3.1.30) |
| CDS | contig00067 | 18854 | 20134 | 2 | + | 1281 | FIG00361618: hypothetical protein |
| CDS | contig00067 | 20350 | 20565 | 1 | + | 216 | FIG074102: hypothetical protein |
| CDS | contig00067 | 21288 | 20638 | -3 | - | 651 | Sigma cross-reacting protein 27A |
| CDS | contig00067 | 21690 | 24440 | 3 | + | 2751 | DNA polymerase I (EC 2.7.7.7) |
| CDS | contig00067 | 25084 | 24521 | -1 | - | 564 | carbonic anhydrase, family 3 |
| CDS | contig00068 | 148 | 354 | 1 | + | 207 | DNA repair protein RadC |
| CDS | contig00068 | 3636 | 577 | -3 | - | 3060 | helicase, putative |
| CDS | contig00068 | 5697 | 3637 | -3 | - | 2061 | FIG00964765: hypothetical protein |
| CDS | contig00068 | 6461 | 5694 | -2 | - | 768 | chemotaxis protein MotB-related protein |
| CDS | contig00068 | 8521 | 6461 | -1 | - | 2061 | Methyl-accepting chemotaxis protein |
| CDS | contig00068 | 9408 | 8695 | -3 | - | 714 | Putative predicted metal-dependent hydrolase |
| CDS | contig00068 | 12539 | 9408 | -2 | - | 3132 | Type I restriction-modification system, restriction subunit R (EC 3.1.21.3) |
| CDS | contig00068 | 13678 | 12614 | -1 | - | 1065 | ADP-ribose 1"-phosphate phophatase related protein |
| CDS | contig00068 | 14328 | 13675 | -3 | - | 654 | FIG00638839: hypothetical protein |
| CDS | contig00068 | 15924 | 14593 | -3 | - | 1332 | Type I restriction-modification system, specificity subunit S (EC 3.1.21.3) |
| CDS | contig00068 | 18371 | 15924 | -2 | - | 2448 | Type I restriction-modification system, DNA-methyltransferase subunit M (EC 2.1.1.72) |
| CDS | contig00068 | 19003 | 18557 | -1 | - | 447 | hypothetical protein |
| CDS | contig00068 | 19424 | 19897 | 2 | + | 474 | Error-prone repair protein UmuD |
| CDS | contig00068 | 19878 | 21137 | 3 | + | 1260 | Error-prone, lesion bypass DNA polymerase V (UmuC) |
| CDS | contig00068 | 21498 | 22151 | 3 | + | 654 | Alpha-amylase precursor (EC 3.2.1.1) (1,4-alpha-D-glucan glucanohydrolase) |
| CDS | contig00068 | 24434 | 22362 | -2 | - | 2073 | 3-phytase precursor (EC 3.1.3.8) |
| CDS | contig00069 | 273 | 905 | 3 | + | 633 | Threonine efflux protein |
| CDS | contig00069 | 1284 | 988 | -3 | - | 297 | DNA-binding protein Fis |
| CDS | contig00069 | 2275 | 1310 | -1 | - | 966 | tRNA dihydrouridine synthase B (EC 1.-.-.-) |
| CDS | contig00069 | 2566 | 3972 | 1 | + | 1407 | Outer membrane protein assembly factor YaeT precursor |
| CDS | contig00069 | 4054 | 3929 | -1 | - | 126 | hypothetical protein |
| CDS | contig00069 | 4046 | 5050 | 2 | + | 1005 | Membrane fusion component of tripartite multidrug resistance system |
| CDS | contig00069 | 5050 | 6186 | 1 | + | 1137 | ABC-type multidrug transport system, permease component |
| CDS | contig00069 | 6183 | 7376 | 3 | + | 1194 | ABC-type multidrug transport system, permease component |
| CDS | contig00069 | 8068 | 7433 | -1 | - | 636 | Flavin reductase (EC 1.5.1.30) |
| CDS | contig00069 | 9050 | 8196 | -2 | - | 855 | DNA replication protein DnaC |
| CDS | contig00069 | 9797 | 9123 | -2 | - | 675 | FIG00361637: hypothetical protein |
| CDS | contig00069 | 9765 | 9935 | 3 | + | 171 | hypothetical protein |
| CDS | contig00069 | 10100 | 11044 | 2 | + | 945 | Probable response regulator |
| CDS | contig00069 | 12416 | 11115 | -2 | - | 1302 | Di-/tripeptide transporter |
| CDS | contig00069 | 13637 | 12726 | -2 | - | 912 | UTP--glucose-1-phosphate uridylyltransferase (EC 2.7.7.9) |
| CDS | contig00069 | 14049 | 13732 | -3 | - | 318 | Competence protein ComEA |
| CDS | contig00069 | 14216 | 14986 | 2 | + | 771 | 3&#39;(2&#39;),5&#39;-bisphosphate nucleotidase (EC 3.1.3.7) |
| CDS | contig00069 | 15346 | 15050 | -1 | - | 297 | YciL protein |
| CDS | contig00069 | 15922 | 15371 | -1 | - | 552 | Intracellular septation protein IspA |
| CDS | contig00069 | 17532 | 16027 | -3 | - | 1506 | Glutamate decarboxylase, eukaryotic type (EC 4.1.1.15) |
| CDS | contig00069 | 17506 | 17682 | 1 | + | 177 | hypothetical protein |
| CDS | contig00069 | 17724 | 18356 | 3 | + | 633 | COG1272: Predicted membrane protein hemolysin III homolog |
| CDS | contig00069 | 18478 | 20139 | 1 | + | 1662 | Mediator of hyperadherence YidE |
| CDS | contig00069 | 20628 | 20350 | -3 | - | 279 | hypothetical protein |
| CDS | contig00069 | 22224 | 21046 | -3 | - | 1179 | hypothetical protein |
| CDS | contig00070 | 175 | 1245 | 1 | + | 1071 | hypothetical protein |
| CDS | contig00070 | 1208 | 1573 | 2 | + | 366 | hypothetical protein |
| CDS | contig00070 | 2126 | 1974 | -2 | - | 153 | hypothetical protein |
| CDS | contig00070 | 2289 | 2465 | 3 | + | 177 | hypothetical protein |
| CDS | contig00070 | 2715 | 3653 | 3 | + | 939 | Mobile element protein |
| CDS | contig00070 | 3696 | 3986 | 3 | + | 291 | FIG045511: hypothetical antitoxin (to FIG022160: hypothetical toxin) |
| CDS | contig00070 | 4311 | 4424 | 3 | + | 114 | hypothetical protein |
| CDS | contig00070 | 5348 | 4521 | -2 | - | 828 | Putative NAD(P)-dependent oxidoreductase EC-YbbO |
| CDS | contig00070 | 6811 | 5897 | -1 | - | 915 | 6-phosphofructokinase (EC 2.7.1.11) |
| CDS | contig00070 | 7767 | 7012 | -3 | - | 756 | Triosephosphate isomerase (EC 5.3.1.1) |
| CDS | contig00070 | 8688 | 7903 | -3 | - | 786 | Membrane Protein Functionally coupled to the MukBEF Chromosome Partitioning Mechanism |
| CDS | contig00070 | 8817 | 9593 | 3 | + | 777 | S-adenosylmethionine-dependent methyltransferase Functionally Coupled to the MukBEF Chromosome Partitioning Mechanism |
| CDS | contig00070 | 9708 | 11027 | 3 | + | 1320 | Chromosome partition protein MukF |
| CDS | contig00070 | 11035 | 11745 | 1 | + | 711 | Chromosome partition protein MukE |
| CDS | contig00070 | 11791 | 16218 | 1 | + | 4428 | Chromosome partition protein MukB |
| CDS | contig00070 | 16369 | 16755 | 1 | + | 387 | Rare lipoprotein A precursor |
| CDS | contig00070 | 17044 | 17517 | 1 | + | 474 | hypothetical protein |
| CDS | contig00070 | 17549 | 18019 | 2 | + | 471 | hypothetical protein |
| CDS | contig00070 | 18264 | 18593 | 3 | + | 330 | FIG00361636: hypothetical protein |
| CDS | contig00070 | 18772 | 18897 | 1 | + | 126 | hypothetical protein |
| CDS | contig00070 | 18936 | 19427 | 3 | + | 492 | Phosphohistidine phosphatase SixA |
| CDS | contig00070 | 19979 | 19500 | -2 | - | 480 | hypothetical protein |
| CDS | contig00071 | 21 | 4922 | 3 | + | 4902 | T1SS secreted agglutinin RTX |
| CDS | contig00071 | 6283 | 5090 | -1 | - | 1194 | MFS superfamily export protein YceL |
| CDS | contig00071 | 6492 | 7385 | 3 | + | 894 | Transcriptional regulator, LysR family |
| CDS | contig00071 | 9215 | 7443 | -2 | - | 1773 | Vibriolysin, extracellular zinc protease (EC 3.4.24.25) @ Pseudolysin, extracellular zinc protease (EC 3.4.24.26) |
| CDS | contig00071 | 9567 | 10121 | 3 | + | 555 | Ribosomal-protein-L7p-serine acetyltransferase |
| CDS | contig00071 | 10159 | 10713 | 1 | + | 555 | Ribosomal-protein-S5p-alanine acetyltransferase |
| CDS | contig00071 | 10888 | 11994 | 1 | + | 1107 | FIG01199554: hypothetical protein |
| CDS | contig00071 | 12304 | 12774 | 1 | + | 471 | Protein yjgK |
| CDS | contig00071 | 12977 | 12747 | -2 | - | 231 | hypothetical protein |
| CDS | contig00071 | 12993 | 14192 | 3 | + | 1200 | Acetate kinase (EC 2.7.2.1) |
| CDS | contig00071 | 14324 | 15565 | 2 | + | 1242 | Small-conductance mechanosensitive channel |
| CDS | contig00071 | 15729 | 15613 | -3 | - | 117 | hypothetical protein |
| CDS | contig00071 | 15751 | 16677 | 1 | + | 927 | L,D-transpeptidase YbiS |
| CDS | contig00071 | 16970 | 18460 | 2 | + | 1491 | Proline/sodium symporter PutP (TC 2.A.21.2.1) @ Propionate/sodium symporter |
| CDS | contig00071 | 19137 | 18532 | -3 | - | 606 | NAD(P)H dehydrogenase (quinone) |
| CDS | contig00071 | 19386 | 19192 | -3 | - | 195 | Transcriptional regulator, MerR family |
| CDS | contig00072 | 1348 | 302 | -1 | - | 1047 | Enoyl-[acyl-carrier-protein] reductase [FMN] (EC 1.3.1.9) |
| CDS | contig00072 | 2235 | 1552 | -3 | - | 684 | Ribonuclease precursor (EC 3.1.27.-) |
| CDS | contig00072 | 2415 | 2975 | 3 | + | 561 | Carbonic anhydrase, gamma class (EC 4.2.1.1) |
| CDS | contig00072 | 3221 | 3415 | 2 | + | 195 | hypothetical protein |
| CDS | contig00072 | 3417 | 3827 | 3 | + | 411 | hypothetical protein |
| CDS | contig00072 | 5254 | 3917 | -1 | - | 1338 | Putative two-component system sensor kinase |
| CDS | contig00072 | 5931 | 5251 | -3 | - | 681 | Transcriptional regulatory protein cpxR |
| CDS | contig00072 | 6080 | 7240 | 2 | + | 1161 | Macrolide-specific efflux protein MacA |
| CDS | contig00073 | 2122 | 227 | -1 | - | 1896 | Putative phosphatase |
| CDS | contig00073 | 3296 | 2388 | -2 | - | 909 | FIG00638797: hypothetical protein |
| CDS | contig00073 | 3447 | 3908 | 3 | + | 462 | hypothetical protein |
| CDS | contig00073 | 5061 | 3916 | -3 | - | 1146 | Putative drug efflux protein |
| CDS | contig00073 | 5389 | 6297 | 1 | + | 909 | regulatory protein, LysR:LysR, substrate-binding |
| CDS | contig00073 | 6387 | 7058 | 3 | + | 672 | Probable lipoprotein |
| CDS | contig00073 | 7070 | 7429 | 2 | + | 360 | Probable lipoprotein |
| CDS | contig00073 | 7426 | 8079 | 1 | + | 654 | Phosphonate ABC transporter phosphate-binding periplasmic component (TC 3.A.1.9.1) |
| CDS | contig00074 | 1677 | 1234 | -3 | - | 444 | Error-prone repair protein UmuD |
| CDS | contig00074 | 1794 | 1982 | 3 | + | 189 | hypothetical protein |
| CDS | contig00074 | 3881 | 4429 | 2 | + | 549 | FIG01203453: hypothetical protein |
| CDS | contig00074 | 4447 | 7920 | 1 | + | 3474 | FIG01059430: hypothetical protein |
| CDS | contig00074 | 8054 | 11494 | 2 | + | 3441 | Type II restriction enzyme, methylase subunits |
| CDS | contig00074 | 11505 | 12785 | 3 | + | 1281 | hypothetical protein |
| CDS | contig00074 | 13136 | 13390 | 2 | + | 255 | hypothetical protein |
| CDS | contig00074 | 13501 | 15477 | 1 | + | 1977 | FIG00971137: hypothetical protein |
[truncated: 18,940 more chars]
