# Supplementary material for: Genome-based insights into the resistome and mobilome of multidrug-resistant Aeromonas sp. ARM81 isolated from wastewater
Source: Arch Microbiol. 2016 Sep 2;199(1):177–83. doi: 10.1007/s00203-016-1285-6 (PMC5216076; doi:10.1007/s00203-016-1285-6)
Supplement: Supplementary file 3 — Supplementary material 3 (DOCX 33 kb) [file 203_2016_1285_MOESM3_ESM.docx]

Table S3. Genes and ncRNAs located within *Aeromonas* sp. ARM81 plasmids.

| **Genes and ncRNA located within plasmid pARM81P1** | | | | | | | |
| --- | --- | --- | --- | --- | --- | --- | --- |
| Gene/  ncRNA no. | Coding region (bp) | Orientation | Protein size (aa) | Possible function | Best pBLAST hits | | |
|  |  |  |  |  | Percentage identity (aa) | Organism | GenBank accession no. |
| 1 | 460-705 | ← | 81 | Initiator RepB protein | 91  (73/80) | *Aeromonas media* | WP_005325944 |
| 2 | 1395-1496 | → | - | RNAI | - | *-* | - |
| 3 | 1607-2890 | ← | 427 | Mobilization protein | 49  (207/425) | *Aeromonas simiae* | WP_052449398 |
| **Genes and ncRNA located within plasmid pARM81P2** | | | | | | | |
| Gene/  ncRNA no. | Coding region (bp) | Orientation | Protein size (aa) | Possible function | Best pBLAST hits | | |
|  |  |  |  |  | Percentage identity (aa) | Organism | GenBank accession no. |
| 1 | 273-375 | ← | - | RNAI | - | *-* | - |
| 2 | 1242-1481 | → | 79 | Initiator RepB protein | 95  (75/79) | *Aeromonas veronii* | WP_064336572 |
| 3 | 1620-1814 | ← | 64 | Hypothetical protein | 100  (56/56) | *Aeromonas caviae* | KMY27470 |
| 4 | 1800-1979 | → | 59 | Hypothetical protein | 98  (58/59) | uncultured bacterium | ALG87815 |
| 5 | 2039-3136 | → | 365 | Zinc transporter ZitB | 100  (365/365) | *Aeromonas caviae* | KMY27471 |
| 6 | 3180-3557 | → | 125 | Transcriptional regulator ArsR | 100  (125/125) | *Aeromonas caviae* | KMY27472 |
| 7 | 3661-4656 | ← | 331 | Hypothetical protein | 80  (259/325) | *Aeromonas veronii* | AMQ43325 |
| 8 | 4856-5281 | ← | 141 | Hypothetical protein | 65  (91/139) | *Enterobacter cloacae* | SAE82776 |
| **Genes and ncRNA located within plasmid pARM81P3** | | | | | | | |
| Gene/  ncRNA no. | Coding region (bp) | Orientation | Protein size (aa) | Possible function | Best pBLAST hits | | |
|  |  |  |  |  | Percentage identity (aa) | Organism | GenBank accession no. |
| 1 | 208-498 | → | 96 | Hypothetical protein | 99  (95/96) | *Aeromonas media* | WP_042650710 |
| 2 | 648-1592 | ← | 314 | Replicase | 100  (314/314) | *Aeromonas media* | WP_042650711 |
| 3 | 1607-1725 | ← | - | PK-repBA | - | - | - |
| 4 | 1802-2086 | ← | 94 | Toxin | 100  (94/94) | *Aeromonas media* | WP_042650712 |
| 5 | 2076-2315 | ← | 106 | Antitoxin | 100  (79/79) | *Escherichia coli* D6-117.29 | CDP79009 |
| 6 | 2464-3990 | ← | 508 | Glucosyl transferase | 38  (187/493) | *Enterobacter cloacae* | WP_049005608 |
| 7 | 4497-4703 | ← | 68 | Hypothetical protein | 100  (68/68) | *Aeromonas media* | WP_042650713 |
| 8 | 4719-4925 | ← | 68 | Hypothetical protein | 100  (68/68) | *Aeromonas media* | WP_042650714 |
| 9 | 4965-6578 | ← | 537 | Mobilization protein | 70  (381/551) | *Aeromonas salmonicida* | WP_005321947 |
| 10 | 6568-6981 | ← | 137 | Mobilization protein | 100  (137/137) | *Aeromonas media* | WP_042650709 |
| **Genes located within plasmid pARM81P4** | | | | | | | |
| Gene no. | Coding region (bp) | Orientation | Protein size (aa) | Possible function | Best pBLAST hits | | |
|  |  |  |  |  | Percentage identity (aa) | Organism | GenBank accession no. |
| 1 | 2787-3029 | ← | 80 | Hypothetical protein | 47  (37/78) | *Escherichia coli* MS 146-1 | EFK89037 |
| 2 | 3022-3294 | ← | 90 | Hypothetical protein | 80  (66/83) | *Raoultella ornithinolytica* | WP_064359009 |
| 3 | 3863-6046 | ← | 727 | Hypothetical bifunctional protein | 46  (238/523) | *Burkholderia* sp. LMG 29317 | SAL47300 |
| 4 | 6043-6222 | ← | 59 | Hypothetical protein | 47  (26/55) | *Aeromonas hydrophila* | WP_017408458 |
| 5 | 6492-6992 | ← | 66 | Hypothetical protein | 49  (32/65) | *Aeromonas sobria* | WP_042021560 |
| 6 | 6736-7599 | ← | 287 | Hypothetical protein | 45  (117/261) | *Klebsiella pneumoniae* | WP_064168307 |
